# Comparison of influencing factors on outcomes of single and multiple road traffic injuries: A regional study in Shanghai, China (2011-2014)

**DOI:** 10.1371/journal.pone.0176907

**Published:** 2017-05-11

**Authors:** Wenya Yu, Haiping Chen, Yipeng Lv, Qiangyu Deng, Peng Kang, Lulu Zhang

**Affiliations:** Department of Military Health Service Management, College of Military Health Service Management, Second Military Medical University, Shanghai, China; Beihang University, CHINA

## Abstract

**Introduction:**

To identify key intervention factors and reduce road traffic injury (RTI)-associated mortality, this study compared outcomes and influencing factors of single and multiple road traffic injuries (RTIs) in Shanghai.

**Methods:**

Based on the design of National Trauma Data Bank, this study collected demographic, injury, and outcome data from RTI patients treated at the four largest trauma centers in Shanghai from January 2011 to January 2015. Data were analyzed with descriptive statistics, univariate analysis, and hierarchical logistic regression analysis.

**Results:**

Among 2397 participants, 59.4% had a single injury, and 40.6% had multiple injuries. Most patients’ outcome was cure or improvement. For single-RTI patients, length of stay, body region, central nervous system injury, acute renal failure, multiple organ dysfunction syndrome, bacterial infection, and coma were significantly related to outcome. For multiple-RTI patients, age, admission pathway, prehospital time, length of stay, number of body regions, body region, injury condition, injury severity score, and coma were significantly related to outcome.

**Conclusions:**

Emergency rescue in road traffic accidents should focus on high-risk groups (the elderly), high-incidence body regions (head, thorax, pelvis) and number of injuries, injury condition (central nervous system injury, coma, complications, admission pathway), injury severity (critically injured patients), and time factors (particularly prehospital time).

## Introduction

According to the World Health Organization (WHO), approximately 1.24 million people globally die every year from road traffic injuries (RTIs)[1]. RTIs are the leading cause of death and disability among young people aged 15–29. Even in high-income countries where RTI mortality has continually declined, road traffic accidents remain a major cause of death and disability. This negative effect is especially great in low- and middle-income countries. According to WHO estimates, RTIs will become the fifth leading cause of death worldwide by 2030 if effective measures are not adopted in a timely manner[1]. In recent years, RTI mortality in China as steadily increased, rising from 8.65/100,000in 2010 to 8.7/100,000 in 2011and 9.04/100,000 in 2012[2–4].

The United Nations has supported in-depth studies on RTIs and called for all countries to take active and effective measures to intervene in the occurrence of RTIs and improve the technology and efficiency of prehospital and hospital first-aid treatment[5]. Both domestic and international studies have focused primarily on the severity and outcome of RTIs, generally through statistical analyses to explore influencing factors. Such factors include demographic characteristics (e.g., gender and age)[6], characteristics of road traffic accidents (e.g., time[7], geographic location[8], road condition[9], vehicle[10], and individual behaviors[11]), and clinical diagnosis and treatment (e.g., body region[12], clinical diagnosis[13], and first-aid time[14]). However, less than 50% of nations have carried out relevant research, and only 15% have developed comprehensive laws to address risk factors of RTIs. In addition, few studies have compared single and multiple RTIs. However, there are obvious differences in outcome, injury condition, and first-aid pattern between single- and multiple-RTI patients, and the mortality of multiple-RTI patients is higher. Therefore, to further clarify the specific body regions, injury condition, outcomes, and influencing factors of multiple-RTI patients, this study divided subjects into single- and multiple-RTI groups according to the number of injured body regions rather than the number of injuries. The outcomes and influencing factors of the two groups were considered separately. In addition, the classification of RTIs was based on the primary mechanism of trauma. For example, if a pedestrian suffered from skull fracture caused by a car accident and concussion due to ground contact, this person was considered a single-RTI patient because the primary mechanism was the road traffic accident, and the two injuries were in the same body region.

Studies on RTIs in developed countries have found that death due to RTI can be prevented[15, 16]. In recent years, China has also promoted RTI studies, but most have been regional studies[17, 18]. In this study, Shanghai was selected as the area of interest. Shanghai is the economic center of China, with more than 23 million residents, an advanced elevated road system, road traffic management system, and emergency rescue system[19]. However, due to the population density and complexity of the road traffic network, it is extremely challenging to guide the effective organization of emergency rescue with information on the occurrence and outcomes of RTIs[20]. Therefore, this study aimed to explore RTIs by analyzing outcomes of RTI patients and related influencing factors. These findings provide a theoretical basis for the emergency rescue of RTI patients in Shanghai, and serve as an example for other areas to develop practices to improve the efficiency of RTI rescue.

## Methods

### Study design

The United States established the National Trauma Data Bank (NTDB) in1982, which is the largest trauma registry system in the world. It provides extensive high-quality data for trauma research and includes 6 dimensions, namely, facility information, demographic information, injury characteristics, outcome information, regional analysis, and comparative analysis[21]. We devised a trauma system (e.g., research dimensions and indicators) based on the framework of the NTDB and national conditions in China.

Since the survey was only conducted in Shanghai, regional and comparative analyses were not taken into account. Because this study focused on patients, facility information was not included. Information on RTIs included 17 indicators consisting of demographic information, injury characteristics, and outcome information. Demographic information included gender, age, and marital status. Injury characteristics referred to body region, injury condition, injury mechanism, coma, and injury severity score. Outcome information consisted of admission pathway, prehospital time, length of stay (LOS), outcome, hospital complication, and comorbidity (diabetes, hypertension and osteoporosis).

### Data collection

Data collection was initiated in January 2011, and all data were derived from the 4 largest trauma centers in Shanghai. Each participating patient was informed of the purpose of this study and provided written consent before hospital admission. Information on these patients was extracted from the medical record systems of the 4 trauma centers (e.g., Burn Emergency Center, Orthopedic Trauma Emergency Center, Acute Trauma Center, and Trauma Emergency Center). This survey included all eligible medical records of patients who agreed to participate in the research. Data were collected according to International Classification of Diseases (ICD-10) and the guidelines of the Chinese Medical Record Registration System. Medical records were screened and exported from the system if they met the inclusion criteria. A preliminary survey was performed in a tertiary hospital in Shanghai, including 834 trauma patients, whose medical records were imported into the pre-designed trauma database. The preliminary survey results confirmed the rationality and feasibility of the database, and some unclear items were revised. Investigators were all trained and informed of the explicit survey aims and requirements. Meanwhile, to guarantee the accuracy and reliability of the data, we assigned a senior administrative staff member for the purpose of contact, and a doctor in each trauma center to guide us to make an accurate screen.

The response rate of the study was 82.9%, with 2890 RTI records were collected, and 2397 found to be complete and consistent with the inclusion criteria. Of these 2397 records, there were 1424 records of single-RTI patients (59.4%) (S1 Dataset) and 973 multiple-RTI patients (40.6%) (S2 Dataset).

### Data analysis

Two authors simultaneously recorded the data using EpiData software version 3.1, and the data were analyzed by using SPSS software version 21.0. Descriptive statistics were used to describe the frequency, percentage, standard deviation, and normal distribution of the data. According to the distribution and types of variables, different methods of univariate analysis were employed for the preliminary screening of factors influencing outcome. The Mann-Whitney U test was used to test the relationship between outcome and the dichotomous variables (e.g., gender, injury condition, affected body region of multiple-RTI patients, coma, complications, and comorbidity). The Kruskal-Wallis H test was used to test disordered multivariate variables (e.g., marital status, admission pathway, and affected body region of single-RTI patients). Spearman rank correlation test was used for ordered multivariate variables (e.g., age, prehospital time, LOS, number of affected body regions of multiple-RTI patients, Glasgow Coma Scale [GCS], and Abbreviated Injury Scale [AIS]/Injury Severity Score [ISS]). Furthermore, AIS or ISS is an international criterion reflecting a patient’s injury condition and has explicit and easy calculation rules. The AIS is a method proposed in 1971 to rate the severity of injuries caused by automotive crashes[22] and is appropriate for single-RTI patients. The AIS ranges from 1 (minor) to 6 (fatal). The ISS was developed based on the AIS and aimed at describing the severity of multiple injuries[23]. Each injured body region has an AIS value, and ISS is calculated by summing the three highest AIS values. The ISS ranges from 1 (minor) to 75 (fatal)[24]. Finally, hierarchical logistic regression analysis was used to further assess the influencing effect of the screened factors on RTI patient outcomes. All tests were two-sided, and P <0.05 was considered statistically significant.

### Ethics statement

The study was approved by the ethics committee of the Second Military Medical University. Only patients who provided written consent were included in the study. All participants were clearly aware of the purpose of the study, and they were all volunteers. We guaranteed the personal information security and privacy of all participants.

## Results

### Demographic characteristics and epidemiological information

#### Single-RTI patients

The outcomes of the 1,424 single-RTI patients were good: 90% were improved or cured, and only a small proportion died (2.04%). In the analysis of demographic information, male patients accounted for the majority. Most patients were over 44 years old, and those aged 55–64 formed the largest group. Nearly half of patients were injured in the extremities, followed by the head and spine. Fracture and joint injuries (FJI), skin and soft tissue injuries (SSTI), and central nervous system injuries (CNSI) were the most commonly diagnosed injuries. Only a small number of single-RTI patients were in a coma upon admission. The AIS scores of most single-RTI patients were ≤ 3 (not very serious), and the proportion of critical patients was small. Most patients were admitted from an emergency department, and 45.2% of patients were referred from other hospitals in Shanghai or surrounding areas. The average prehospital time was lengthy; only 27.9% of patients were admitted into a hospital in 3 hours. Most patients’ LOS was 8–14 days, followed by 4–7 days and 15–30 days. Complications consisted mainly of disturbance of consciousness (DC), bacterial infection (BI), and paraplegia. Among single-RTI patients, 17.6% had concomitant diabetes, hypertension, or osteoporosis (Table 1).

**Table 1:** Descriptive analysis and univariate analysis of single-RTI patients.

Characteristics	Total (%)	Dead	Invalid	Improved	Cured	P-value
**Total**	**1424**	**29**	**62**	**452**	**881**	
**Gender**						0.914
Male	915 (64.26)	17	39	295	564	
Female	509 (35.74)	12	23	157	317	
**Age**						0.760
0–14	27 (1.9)	1	4	4	18	
15–24	134 (9.41)	0	7	54	73	
25–34	176 (12.36)	2	8	56	110	
35–44	228 (16.01)	0	7	78	143	
45–54	318 (22.33)	8	17	92	201	
55–64	365 (25.63)	11	13	111	230	
≥65	176 (12.36)	7	6	57	106	
**Marital status**						0.017
Divorced/widowed	5 (0.35)	0	0	0	5	
Single	353 (24.79)	5	22	126	200	
Married	1066 (74.86)	24	40	326	676	
**Body region**						<0.001
Head	475 (33.36)	27	27	252	169	
Thorax	42 (2.95)	1	0	29	12	
Abdomen	17 (1.19)	1	1	8	7	
Spine	147 (10.32)	0	4	41	102	
Pelvis	43 (3.02)	0	3	14	26	
Extremity	700 (49.16)	0	27	108	565	
**Injury condition**						
FJI*(yes)	1070 (75.14)	16	47	295	712	<0.001
no	354 (24.86)	13	15	157	169	
SSTI* (yes)	738 (51.83)	24	27	290	397	<0.001
No	686 (48.17)	5	35	162	484	
Destruction (yes)	29 (2.04)	0	0	7	22	0.087
no	1395 (97.96)	29	62	445	859	
CNSI* (yes)	441 (30.97)	27	24	227	163	<0.001
no	983 (69.03)	2	38	225	718	
PC* (yes)	27 (1.90)	0	0	18	9	0.010
no	1397 (98.10)	29	62	434	872	
TH* (yes)	10 (0.70)	1	0	5	4	0.152
no	1414 (99.30)	28	62	447	877	
TOI* (yes)	18 (1.26)	1	1	11	5	0.003
no	1406 (98.74)	28	61	441	876	
**Coma (yes)**	131 (9.20)	17	9	69	36	<0.001
no	1293 (90.80)	12	53	383	845	
**AIS**						<0.001
1	94 (6.60)	0	2	40	52	
2	549 (38.55)	0	32	120	397	
3	676 (47.47)	16	19	246	395	
4	49 (3.44)	2	3	22	22	
5	52 (3.65)	11	6	21	14	
6	4 (0.28)	0	0	3	1	
**Admission pathway**						0.314
Non-local referral^†^	327 (22.96)	1	21	115	190	
Local referral^†^	316 (22.19)	6	14	100	196	
ED admission^†^	781 (54.85)	22	27	237	495	
**Prehospital time**						0.411
≤1h	23 (1.62)	2	1	11	9	
1-3h	232 (16.29)	13	10	62	147	
3-24h	599 (42.06)	10	18	182	389	
>24h	570 (40.03)	4	33	197	336	
**Length of stay**						<0.001
0-3d	106 (7.44)	11	29	37	29	
4-7d	361 (25.35)	9	22	126	204	
8-14d	470 (33.01)	3	7	143	317	
15-30d	346 (24.30)	4	3	106	233	
≥31d	141 (9.90)	2	1	40	98	
**Complications**						
HS^☨^ (yes)	23 (1.62)	3	1	7	12	0.178
No	1401 (98.38)	26	61	445	869	
DC^☨^ (yes)	349 (24.51)	26	19	169	135	<0.001
no	1075 (75.49)	3	43	283	746	
Apnea (yes)	2 (0.14)	2	0	0	0	0.005
no	1422 (99.86)	27	62	452	881	
CA^☨^ (yes)	2 (0.14)	1	0	0	1	0.391
no	1422 (99.86)	28	62	452	880	
CH^☨^ (yes)	14 (0.98)	5	2	2	5	0.001
no	1410 (99.02)	24	60	450	876	
Paraplegia (yes)	41 (2.88)	0	2	17	22	0.356
no	1383 (97.12)	29	60	435	859	
AF^☨^ (yes)	6 (0.42)	1	0	3	2	0.119
no	1418 (99.58)	28	62	449	879	
WED^☨^ (yes)	3 (0.21)	0	0	2	1	0.394
no	1421 (99.79)	29	62	450	880	
ARF^☨^ (yes)	5 (0.35)	3	0	0	2	0.048
no	1419 (99.65)	26	62	452	879	
MOF^☨^ (yes)	1 (0.07)	1	0	0	0	0.047
no	1423 (99.93)	28	62	452	881	
MODS^☨^ (yes)	8 (0.56)	5	1	1	1	<0.001
no	1416 (99.44)	24	61	451	880	
BI^☨^(yes)	60 (4.21)	9	2	32	17	<0.001
no	1364 (95.79)	20	60	420	864	
**Comorbidity**						
Diabetes (yes)	86 (6.04)	5	5	31	45	0.032
no	1338 (93.96)	24	57	421	836	
Hypertension (yes)	164 (11.52)	6	3	59	96	0.425
no	1260 (88.48)	23	59	393	785	
Osteoporosis (yes)	1338 (0.07)	24	57	421	836	0.440
no	1423 (99.93)	29	62	452	880	

* FJI: fracture and joint injuries; SSTI: skin and soft tissue injuries; CNSI: central nervous system injuries; PC: pulmonary contusion; TH: traumatic haemopneumothorax; TOI: traumatic organ injuries.

^†^ Non-local referral: referral from hospitals in other cities; Local referral: referral from hospitals in Shanghai; ED admission: emergency department admission.

^☨^ HS: hemorrhagic shock; DC: disturbance of consciousness; CA: cardiac arrest; CH: cerebral hernia; AF: ardent fever; WED: water and electrolyte disturbance; ARF: acute renal failure; MOF: multiple organ failure; MODS: multiple organ dysfunction syndrome; BI: bacterial infection.

To explore the influencing factors of patient outcomes, we conducted a univariate analysis to investigate the effect of demographic information, injury characteristics, and treatment information on patient outcomes. Before this analysis, we checked the distribution of independent variable via Shapiro-Wilk test. The statistical value of the Shapiro-Wilk test was 0.673 (P < 0.0001), indicating that the distribution was not normal. Thus, Mann-Whitney U test, Kruskal-Wallis H test and Spearman rank correlation test were chosen for the univariate analysis. The univariate analysis results indicated that many factors were significantly related to the outcomes of single-RTI patients, including marital status; LOS; body region; suffering from FJI, SSTI, CNSI, pulmonary contusion (PC), or traumatic organ injuries (TOI); injury accompanied by DC, apnea, cerebral hernia (CH), acute renal failure (ARF), multiple organ failure (MOF), multiple organ dysfunction syndrome (MODS), or BI; coma status; AIS; and concomitant diabetes (Table 1).

#### Multiple-RTI patients

The outcomes, demographic information, injury characteristics, and admission pathways of multiple-RTI patients were similar to those of single-RTI patients. Most patients were cured, but the mortality of multiple-RTI patients was higher than that of single-RTI patients. Patients who were male, aged 55–64, and married accounted for the largest proportion. Most patients were injured in 2 to 3 body regions. The most vulnerable body regions were the head, thorax, and extremities. The most common injury diagnoses were similar to those of single-RTI patients, namely, FJI, SSTI, and CNSI, and 7.7% of coma patients were in a coma upon transfer to hospitals. Most multiple-RTI patients were severely injured; more than half had an ISS ≥ 16. The timeliness of prehospital emergency rescue was unsatisfactory, as only approximately one-fifth of patients (20.45%) had a prehospital time less than 3 hours. More patients were admitted from the emergency department, but the proportion of patients referred from hospitals in other areas was higher than that in single-RTI patients. LOS of multiple-RTI patients was longer than that of single-RTI patients; the most common was 15–30 days, followed by 8–14 days. The most frequent complications were DC, MODS, hemorrhagic shock (HS), and BI, and 17.9% of patients were also suffering from hypertension or diabetes (Table 2).

**Table 2:** Descriptive analysis and univariate analysis for multiple-RTI patients.

Characteristics	Total (%)	Dead	Invalid	Improved	Cured	P-value
**Total**	**973**	**22**	**24**	**391**	**536**	
**Gender**						0.600
Male	669 (68.76)	17	20	265	367	
Female	304 (31.24)	5	4	126	169	
**Age**						0.013
0–14	19 (1.95)	0	0	11	8	
15–24	64 (6.58)	1	0	17	46	
25–34	118 (12.13)	1	1	56	60	
35–44	145 (14.90)	2	4	55	84	
45–54	238 (24.46)	2	7	95	134	
55–64	243 (24.97)	4	6	94	139	
≥65	146 (15.01)	12	6	63	65	
**Marital status**						0.360
Divorced/widowed	2 (0.21)	0	0	2	0	
Single	151 (15.52)	5	0	65	81	
Married	820 (84.28)	17	24	324	455	
**Number of body region**						0.015
2	511 (52.52)	13	13	186	299	
3	294 (30.22)	5	6	125	158	
4	113 (11.61)	1	4	52	56	
5	41 (4.21)	2	1	18	20	
6	13 (1.34)	0	0	10	3	
7	1 (0.10)	1	0	0	0	
**Body region**						
Head	677 (69.58)	19	17	301	340	<0.001
no	296 (30.42)	3	7	90	196	
Thorax	525 (53.96)	16	14	234	261	<0.001
no	448 (46.04)	6	10	157	275	
Abdomen	169 (17.37)	7	8	81	73	<0.001
no	804 (82.63)	15	16	310	463	
Upper extremity	422 (43.37)	7	9	157	249	0.025
no	551 (56.63)	15	15	234	287	
Lower extremity	414 (42.55)	7	8	149	250	0.003
no	559 (57.45)	15	16	242	286	
Spine	276 (28.37)	2	7	123	144	0.420
no	697 (71.63)	20	17	268	392	
Pelvis	163 (16.75)	4	2	60	97	0.199
no	810 (83.25)	18	22	331	439	
**Injury Condition**						
FJI*(yes)	904 (92.91)	21	22	353	508	0.020
no	69 (7.09)	1	2	38	28	
SSTI* (yes)	729 (74.92)	22	15	306	386	0.018
no	244 (25.08)	0	9	85	150	
Destruction (yes)	21 (2.16)	1	0	6	14	0.319
no	952 (97.84)	21	24	385	522	
CNSI* (yes)	419 (43.06)	17	12	215	175	<0.001
no	554 (56.94)	5	12	176	361	
PC* (yes)	292 (30.01)	10	6	137	139	0.002
no	681 (69.99)	12	18	254	397	
TH* (yes)	134 (13.77)	3	5	57	69	0.330
no	839 (86.23)	19	19	334	467	
TOI* (yes)	104 (10.69)	6	6	61	31	<0.001
no	869 (89.31)	16	18	330	505	
**Coma (yes)**	75 (7.71)	8	1	41	25	<0.001
no	898 (92.29)	14	23	350	511	
**ISS**						<0.001
1–8	150 (15.42)	0	4	43	103	
9–15	327 (33.61)	7	8	120	192	
16–24	268 (27.54)	6	3	110	149	
≥25	228 (23.43)	9	9	118	92	
**Admission Pathway**						0.001
Non-local referral^†^	295 (30.32)	1	14	144	136	
Local referral^†^	220 (22.61)	3	7	73	137	
ED admission^†^	458 (47.07)	18	3	174	263	
**Prehospital Time**						<0.001
≤1h	55 (5.65)	3	0	15	37	
1-3h	144 (14.80)	7	1	51	85	
3-24h	400 (41.11)	8	4	151	237	
>24h	374 (38.44)	4	19	174	177	
**Length of Stay**						<0.001
0-3d	43 (4.42)	12	8	14	9	
4-7d	152 (15.62)	2	6	72	72	
8-14d	266 (27.34)	2	6	103	155	
15-30d	320 (32.89)	5	3	144	168	
≥31d	192 (19.73)	1	1	58	132	
**Complications**						
HS^☨^ (yes)	83 (8.53)	7	4	31	41	0.079
no	890 (91.47)	15	20	360	495	
DC^☨^ (yes)	381 (39.16)	15	9	192	165	<0.001
no	592 (60.84)	7	15	199	371	
Apnea (yes)	8 (0.82)	3	1	4	0	<0.001
no	965 (99.18)	19	23	387	536	
CA^☨^ (yes)	8 (0.82)	3	2	3	0	<0.001
no	965 (99.18)	19	22	388	536	
CH^☨^ (yes)	2 (0.21)	1	0	1	0	0.038
no	971 (99.79)	21	24	390	536	
Paraplegia (yes)	56 (5.76)	0	1	28	27	0.430
no	917(84.24)	22	23	363	509	
AF^☨^ (yes)	13 (1.34)	1	0	7	5	0.213
no	960 (98.66)	21	24	384	531	
WED^☨^ (yes)	29 (2.98)	1	6	18	4	<0.001
no	944 (97.02)	21	18	373	532	
ABI^☨^ (yes)	6 (0.62)	1	1	3	1	0.016
no	967 (99.38)	21	23	388	535	
ARDS^☨^ (yes)	6 (0.62)	1	1	2	2	0.103
no	967 (99.38)	21	23	389	534	
ARF^☨^ (yes)	14 (1.44)	3	0	7	4	0.013
no	959 (98.56)	19	24	384	532	
MODS^☨^ (yes)	126 (12.95)	7	8	70	41	<0.001
no	847 (87.05)	15	16	321	495	
BI^☨^(yes)	83 (8.53)	7	4	31	41	<0.001
no	890 (91.47)	15	20	360	495	
**Comorbidity**						
Diabetes (yes)	66 (6.78)	2	2	23	39	0.594
no	907 (93.22)	20	22	368	497	
Hypertension (yes)	108 (11.10)	4	4	43	57	0.458
no	865 (88.90)	18	20	348	479	

* FJI: fracture and joint injuries; SSTI: skin and soft tissue injuries; CNSI: central nervous system injuries; PC: pulmonary contusion; TH: traumatic haemopneumothorax; TOI: traumatic organ injuries.

^†^ Non-local referral: referral from hospitals in other cities; Local referral: referral from hospitals in Shanghai; ED admission: emergency department admission.

^☨^ HS: hemorrhagic shock; DC: disturbance of consciousness; CA: cardiac arrest; CH: cerebral hernia; AF: ardent fever; WED: water and electrolyte disturbance; ABI: acid-Base imbalance; ARDS: acute respiratory distress syndrome; ARF: acute renal failure; MODS: multiple organ dysfunction syndrome; BI: bacterial infection.

The Shapiro-Wilk test for normal distribution revealed a statistical value of 0.691 (P < 0.0001), suggesting the distribution was not normal. Therefore, according to the univariate analysis, multiple-RTI patients’ outcome was significantly influenced by their age; admission pathway; prehospital time; LOS; number of affected body regions; injury to head, thorax, abdomen, or extremities; diagnosis of FJI, SSTI, CNSI, PC, or TOI; presence of concomitant DC, apnea, cardiac arrest (CA), CH, water and electrolyte disturbance (WED), acid-base imbalance (ABI), ARF, MODS, or BI; coma status; and ISS (Table 2).

### Logistic regression analysis of factors associated with outcomes of road traffic injuries

#### Single-RTI patients

Before logistic regression analysis, multicollinearity detection was conducted to check the reasonability of this analysis method via the tolerance (Tol) and the variance inflation factor (VIF). The values of Tol for different variables ranged from 0.471 to 0.876 (all values > 0.1), and the values of VIF ranged from 1.142 to 2.121 (all values < 10), indicating the absence of multicollinearity. According to hierarchical logistic regression analysis, LOS, body region, CNSI, ARF, MODS, BI, and coma status (all P < 0.05) had statistically significant effects on the outcome of single-RTI patients (Table 3).

**Table 3:** Hierarchical logistic regression analysis for single-RTI patients.

Parameter	Estimate	P-value	OR	95% Wald Confidence Limits	
				lower	upper
**x1 marital status (ref: married)**					
x1-1 divorced/widowed	13.322	0.979	1.638E-06	–	–
x1-2 single	-0.156	0.261	1.169	0.892	1.532
x2 LOS* **(ref: ≥31d)**					
x2-1 ≤3d	-2.611	<0.001	13.613	7.502	24.701
x2-2 4-7d	-0.719	0.006	2.052	1.231	3.423
x2-3 8-14d	-0.058	0.817	1.060	0.649	1.730
x2-4 15-30d	0.035	0.889	0.966	0.589	1.582
**x3 body region (ref: extremity)**					
x3-1 head	-1.271	<0.001	3.564	2.343	5.422
x3-2 thorax	-1.778	<0.001	5.918	2.518	13.909
x3-3 abdomen	-0.036	0.966	1.037	0.200	5.368
x3-4 spine	-0.047	0.859	1.048	0.625	1.758
x3-5 pelvis	-0.745	0.034	2.106	1.057	4.199
x4 FJI* **(ref: yes)**	0.201	0.205	0.818	0.599	1.117
x5 SSTI*(ref: yes)	0.274	0.072	0.760	0.563	1.026
x6 CNSI* **(ref: yes)**	0.618	0.003	0.539	0.359	0.810
x7 PC*(ref: yes)	0.163	0.756	0.850	0.304	2.377
x8 TOI* **(ref: yes)**	1.548	0.057	0.213	0.043	1.049
x9 DC* **(ref: yes)**	-0.138	0.453	1.148	0.800	1.646
**x10 apnea (ref: yes)**	15.868	0.971	1.284E-07	–	–
x11 CH* **(ref: yes)**	0.130	0.821	0.878	0.285	2.705
x12 ARF* **(ref: yes)**	2.144	0.020	0.117	0.019	0.710
x13 MOF* **(ref: yes)**	16.398	–	–	–	–
x14 MODS* **(ref: yes)**	2.983	<0.001	0.051	0.012	0.220
x15 BI* **(ref: yes)**	0.645	0.026	0.525	0.297	0.928
x16 AIS* **(ref: AIS = 6)**					
x16-1 AIS = 1	0.149	0.884	0.862	0.116	6.411
x16-2 AIS = 2	0.589	0.558	0.555	0.077	3.986
x16-3 AIS = 3	0.508	0.612	0.602	0.084	4.297
x16-4 AIS = 4	0.004	0.997	0.996	0.127	7.784
x16-5 AIS = 5	-0.360	0.727	1.433	0.190	10.834
**x17 diabetes (ref: yes)**	0.429	0.084	0.651	0.400	1.059
**x18 coma (ref: yes)**	0.638	0.003	0.528	0.347	0.805

* LOS: length of stay; FJI: fracture and joint injuries; SSTI: skin and soft tissue injuries; CNSI: central nervous system injuries; PC: pulmonary contusion; TOI: traumatic organ injuries; DC: disturbance of consciousness; CH: cerebral hernia; ARF: acute renal failure; MOF: multiple organ failure; MODS: multiple organ dysfunction syndrome; BI: bacterial infection; AIS: abbreviated injury scale.

In addition, the mortality rates of patients with different characteristics were compared on the basis of ORs and associated 95% confidence limits, with OR values > 1 indicating increased risk of mortality. Compared to patients whose LOS was more than 31 days, those with LOS less than 3 days (OR = 13.613) or of 4–7 days (OR = 2.052) had a higher risk of death. Patients injured in the head (OR = 3.564), thorax (OR = 5.918), or pelvis (OR = 2.106) were more likely to die in comparison to those with extremity injuries, and mortality was highest for thorax injuries. The risk of death was lower among patients who were not diagnosed with CNSI (OR = 0.539) and not experiencing ARF (OR = 0.117), MODS (OR = 0.051) and BI (OR = 0.525). Among these factors, MODS had the greatest effect on mortality, followed by ARF and BI. If a patient was not in a coma when he/she was admitted to a hospital, the risk of mortality decreased to nearly half of that among patients in a coma (OR = 0.528).

#### Multiple-RTI patients

Before logistic regression analysis, multicollinearity detection results indicated that the values of Tol for different variables ranged from 0.311 to 0.948 (all values > 0.1), and the values of VIF ranged from 1.034 to 3.219 (all values < 10), indicating that multicollinearity was not present. The logistic regression analysis revealed that age, admission pathway, prehospital time, LOS, number of affected body regions, body region, injury condition, complication, ISS, and coma status all significantly influenced multiple-RTI patient outcomes (all P < 0.05) (Table 4).

**Table 4:** Hierarchical logistic regression analysis for multiple-RTI patients.

Parameter	Estimate	P-value	OR	95% Wald Confidence Limits	
				lower	upper
**x1 age (ref: ≥65y)**					
x1-1 0-14y	-0.101	0.853	1.106	0.379	3.226
x1-2 15-24y	1.477	<0.001	0.228	0.110	0.473
x1-3 25-34y	0.570	0.034	0.566	0.334	0.956
x1-4 35-44y	0.515	0.045	0.598	0.361	0.989
x1-5 45-54y	0.694	0.003	0.500	0.318	0.786
x1-6 55-64y	0.672	0.003	0.511	0.327	0.798
x2 admission pathway (ref: ED admission*)					
x2-1 non-local referral*	0.390	0.108	0.677	0.421	1.088
x2-2 local referral*	0.742	<0.001	0.476	0.314	0.723
**x3 prehospital time (ref: >24h)**					
x3-1 ≤1h	1.066	0.007	0.344	0.158	0.751
x3-2 1-3h	0.524	0.064	0.592	0.340	1.031
x3-3 3-24h	0.318	0.124	0.728	0.485	1.092
**x4 LOS (ref: ≥31d)**					
x4-1 ≤3d	-3.667	<0.001	39.134	17.418	87.925
x4-2 4-7d	-1.333	<0.001	3.792	2.256	6.375
x4-3 8-14d	-0.683	0.004	1.980	1.244	3.150
x4-4 15-30d	-0.618	0.005	1.855	1.210	2.844
**x5 number of body region (ref: 7)**					
x5-1 2	20.643	<0.001	1.084E-09	2.632E-10	4.461E-09
x5-2 3	20.841	<0.001	8.889E-10	2.433E-10	3.247E-09
x5-3 4	20.925	<0.001	8.173E-10	2.322E-10	2.877E-09
x5-5 5	21.238	<0.001	5.977E-10	1.592E-10	2.244E-09
x5-6 6	20.354	–	–	–	–
**x6 head (ref: yes)**	0.133	0.569	0.875	0.555	1.382
**x7 thorax (ref: yes)**	0.545	0.010	0.580	0.383	0.877
**x8 abdomen (ref: yes)**	0.248	0.446	0.780	0.413	1.476
**x9 upper limb (ref: yes)**	0.026	0.888	0.974	0.679	1.397
**x10 lower limb (ref: yes)**	0.111	0.563	0.895	0.615	1.301
x11 FJI* **(ref: yes)**	-0.393	0.171	1.481	0.844	2.600
x12 SSTI* **(ref: yes)**	0.229	0.222	0.795	0.550	1.150
x13 CNSI* **(ref: yes)**	0.538	0.004	0.584	0.406	0.839
x14 PC* **(ref: yes)**	-0.180	0.392	1.197	0.792	1.810
x15 TOI* **(ref: yes)**	0.811	0.016	0.444	0.229	0.862
x16 DC* **(ref: yes)**	0.385	0.021	0.680	0.491	0.942
**x17 apnea (ref: yes)**	1.556	0.218	0.211	0.018	2.508
x18 CA* **(ref: yes)**	0.239	0.848	0.787	0.069	8.965
x19 CH* **(ref: yes)**	2.601	0.099	0.074	0.003	1.635
x20 WED* **(ref: yes)**	0.876	0.059	0.416	0.168	1.034
x21 ABI* **(ref: yes)**	-0.045	0.963	1.046	0.156	7.030
x22 ARF* **(ref: yes)**	0.623	0.281	0.536	0.173	1.665
x23 MODS* **(ref: yes)**	1.539	0.003	0.215	0.078	0.591
x24 BI* **(ref: yes)**	0.315	0.177	0.730	0.462	1.152
x25 ISS* **(ref: ≥25)**					
x25-1 1–8	0.985	0.001	0.373	0.204	0.684
x25-2 9–15	0.341	0.145	0.711	0.449	1.125
x25-1 16–24	0.220	0.299	0.803	0.530	1.216
**x26 coma (ref: yes)**	0.674	0.015	0.510	0.296	0.877

* ED admission: emergency department admission; non-local referral: referral from hospitals in other cities; local referral: referral from hospitals in Shanghai; FJI: fracture and joint injuries; SSTI: skin and soft tissue injuries; CNSI: central nervous system injuries; PC: pulmonary contusion; TOI: traumatic organ injuries; DC: disturbance of consciousness; CA: cardiac arrest; CH: cerebral hernia; WED: water and electrolyte disturbance; ABI: acid-Base imbalance; ARF: acute renal failure; MODS: multiple organ dysfunction syndrome; BI: bacterial infection; ISS: injury severity score.

The risk of mortality was also assessed on the basis of ORs and associated CIs. Compared to elderly patients (aged ≥ 65 years), patients aged 15–64 years old were less likely to die (all ORs ≤ 0.598). Among patients aged 15–64 years, the lowest mortality was among those aged 15–24 years (OR = 0.228), and the highest was among those aged 35–44 years (OR = 0.598). Patients who were referred from other hospitals in Shanghai had a lower mortality rate (OR = 0.476) than those admitted from the emergency department. Patients were less likely to die if they could be transported to a hospital within 1 hour (OR = 0.344) after road traffic accidents. LOS was a strong predictor of mortality, with shorter LOS associated with higher mortality (for example, patients with LOS ≤ 3d were 39.134 times more likely to die than those whose LOS was ≥ 31d). The probability of death was lower among patients with fewer affected body regions. Among body regions, only thorax injury was significantly associated with mortality; patients not injured in the thorax had a lower risk of death (OR = 0.580). Patients diagnosed with CNSI (OR = 0.584) or TOI (OR = 0.444) or accompanied with DC (OR = 0.680) or MODS (OR = 0.215) also had higher mortality. Compared to patients with ISS ≥ 25, patients with ISS of 1–8 had lower mortality (OR = 0.373). Patients were also less likely to die if they were not in a coma (OR = 0.510) when transferred to the hospital.

## Discussion

Based on statistical analyses, there were more single-RTI patients than multiple-RTI patients. Mortality among multiple-RTI patients was higher than that of single-RTI patients, and the influence of different factors on the risk of mortality in the two groups varied. Generally speaking, factors with an obvious effect on outcomes included injury characteristics and treatments. Among demographic data, only age had an influence on mortality.

Age was an important factor influencing outcomes among multiple-RTI patients. Outcomes of elderly patients were worse than those of younger patients, which can be explained by their longer response time, more fragile bodies, and weaker tolerance to injuries, thus leading to more severe injuries and a higher risk of death. In addition, as age increases, even a minor injury could be more likely to lead to complications such as BI and MOF, which have severe negative effects on patient outcomes[25]. Therefore, the emergency rescue of RTIs should pay special attention to the elderly, and timely and effective treatments should be administered according to their vital signs and injury characteristics[26–28].

With regard to injury characteristics, body region, injury diagnosis, coma status, and injury severity were major influencing factors of RTI patients’ outcomes. Body region was one of the most significant factors, and the extremities and head had the highest incidence of injury[29, 30]. The influence on mortality varied among different body regions. For single-RTI patients, the highest mortality was among patients injured in the head, thorax, and pelvis. Such patients were more likely to be in a coma and develop complications, and the injury condition was more complicated and severe, and always threatened patients’ lives[12, 13, 29]. For multiple-RTI patients, the more body regions were injured, the more severe the patients’ injury condition was likely to be. This result was supported by a study in Beijing, which suggested that medical staff should especially monitor the vital signs of patients with a greater number of injured body regions[31].

Head injuries are the main cause of death from road traffic accidents[32], and severe head injuries can significantly increase mortality[33], especially among children or teenagers. Studies also have found that taking measures to protect the head, such as wearing a helmet, could effectively reduce road traffic accident mortality[34]. In addition, if a multiple-RTI patient was injured in the thorax, mortality might be increased. The incidence of thorax injures followed those of extremity and head injuries. Twenty-five percent of RTI patients died due to thorax injuries, and another 25% of deaths were significantly related with thorax injuries[35]. Patients with severe thorax injuries generally suffered from breathing and circulatory dysfunction. Most deaths caused by thorax injures occurred at the scene or during transport, and less than 20% occurred in hospitals. Therefore, special attention should be paid to particularly high-risk symptoms during transport (e.g., airway obstruction, tension pneumothorax, and cardiac tamponade), and improving the accuracy of diagnosis of fatal thoracic injuries can substantially decrease mortality.

Pelvic injury was also a major cause of death in RTI patients. Pelvic injuries are always associated with skeletal injuries, soft tissue injuries, and massive hemorrhages, which often result in death. For example, mortality from pelvic ring high-energy fractures has reached 58.3%[36], suggesting the importance of accurate diagnosis and treatment decisions for the reduction of mortality. Finally, extremity injuries were the most common injuries in this study. Although mortality from extremity injuries was relatively low, the disability rate was extremely high. Thus, disabilities caused by extremity injuries can have a significant negative impact on patients, their families, and society as a whole.

RTIs often occur among young adults aged ≤ 40 years, and are the leading cause of death among those aged 15–24 years. They can reduce the quality of life of young adults and pose a threat to their mental health. In this regard, medical staff should show concern about the psychological problems of younger patients and make timely interventions[34]. For families and society overall, disabilities arising from RTIs can substantially reduce the labor force and increase economic burden[37]. Therefore, it is of great significance for individuals, families, and society to be aware of disabilities caused by RTIs.

In terms of injury diagnosis, RTI patients were often diagnosed with FJI, SSTI, and CNSI[12, 13, 18]. CNSI typically has a more significant influence on patient outcomes, and particularly leads to a higher risk of death[10, 38]. Coma status was also associated with higher mortality, which may be related to CNSI. Based on this finding, a rapid classification of RTI patients according to affected body regions should be implemented in emergency rescues. Patients with head injuries, diagnosed with CNSI, or in a coma should be carefully monitored and treated to limit greater damage from delays and misdiagnoses. Moreover, considering that many RTI patients were diagnosed with FJI and SSTI, fixation and hemostasis are particularly important in emergency rescues.

The severity of injuries was another important determinant of outcomes[39]. Single-RTI patients had less severe injuries than multiple-RTI patients. Further analysis of multiple-RTI patients suggested that those with more critical injury conditions were at higher risk of death. AIS and ISS are effective tools for the quick evaluation of injury severity. Thus, in administering first aid for RTIs (especially after massive road traffic accidents), medical staff should make good use of these tools and properly classify patients based on AIS and ISS, which would help to avoid medical delays among critically injured patients and select suitable medical treatments and healthcare institutions for transfer.

Treatment factors also affected patient outcomes. Although most patients were admitted into the hospitals through their emergency departments, this study involved some special circumstances. Since Shanghai possesses one of the most abundant and advanced health resources in China, many critically injured patients would be referred from other areas to Shanghai for better care. Thus, nearly one-third of patients in this study were referred from hospitals in other cities, whose injury conditions were generally more severe and critical. Therefore, mortality among these patients was higher. This phenomenon indicates that medical staff should concentrate more on patients referred from other cities to improve their likelihood of survival. Better communication between hospitals could provide medical staff with a more comprehensive understanding of injuries and compensate for the treatment delay owing to transfer. On the other hand, in low-income countries, about 60% of RTI deaths occur at the scene or during transportation, and 30% occur in hospitals[40]. Moreover, 52.2% of prehospital deaths occurred within 11–15 minutes, and 74.6% within 6–10 minutes[41]. Therefore, it would greatly decrease the rate of disability and death if prehospital emergency rescue capability could be substantially improved[42]. However, the prehospital time in China is far from satisfactory. Statistics indicate that the average prehospital time is too long, and that longer prehospital time can lead to a greater risk of death[43]. Therefore, it is critical to promote the efficiency of prehospital emergency rescue, including the improvement of emergency rescue systems and the training of medical staff, and greatly shortening prehospital time. Once patients were admitted to hospitals, LOS could affect their outcomes. Longer LOS could decrease mortality, which was associated with injury condition. Studies suggest that mortality peaked in the first week. During this period, patients’ injury condition was unstable, the success rate of rescue was not high, and the risk of death was high. In the progression of diseases, the injury condition typically stabilizes after the first week. Therefore, patients with longer LOS were generally in a stable state and had a lower risk of death[33].

Finally, epidemiological research on complications is not common, but complications were an important factor affecting outcomes in the present study. If patients demonstrated complications during the course of hospitalization, their mortality was increased. Specifically, complications such as DC, HS, BI, MODS, and paraplegia can increase mortality[16, 44], indicating the imperative of preventing various complications to improve RTI patients’ rate of survival and quality of life.

This study had two major limitations. First, this study focused on medical emergency rescue of RTIs, but the characteristics of road traffic accidents (e.g., road conditions, causes of accidents, time of accidents) also have a significant impact on patient outcomes. Therefore, characteristics of road traffic accidents should be added in further studies. Second, only the4largesttrauma centers in Shanghai were included in this study, and thus a small proportion of RTI patients in Shanghai were excluded. Thus, the number of participants should be expanded in future research.

This study analyzed outcomes and influencing factors of RTI and compared the similarities and differences between single- and multiple-RTI patients. Through statistical analysis and comparison, we assessed how emergency rescues can be conducted more effectively to significantly reduce mortality. Our findings suggest that the outcomes of single- and multiple-RTI patients were similar, but influencing factors varied. To effectively reduce the mortality of RTIs, emergency rescue should focus on high-risk groups (e.g., mortality of the elderly, disability of young adults), high-incidence body regions (e.g., head, thorax, pelvis) and number of injuries, injury condition (e.g., CNSI, coma, complications, admission pathway), injury severity (e.g., particularly critically injured patients) and time factors (e.g., particularly the prehospital time). By assessing these important factors, a more accurate classification of patients at the scene may be possible, with critically injured patients receiving more judicious health care, which could have a substantial impact on decreasing mortality.

## Supporting information

S1 Dataset Data used to analyze single road traffic injuries. (PDF)

S2 Dataset Data used to analyze multiple road traffic injuries. (PDF)
