# Supplementary material for: Comparison of influencing factors on outcomes of single and multiple road traffic injuries: A regional study in Shanghai, China (2011-2014)
Source: PLoS One. 2017 May 11;12(5):e0176907. doi: 10.1371/journal.pone.0176907 (PMC5426634; doi:10.1371/journal.pone.0176907)
Supplement: S1 Dataset — (PDF) [file pone.0176907.s001.pdf]

ID	Gender	Age	Marital status	Admission pathway	Prehospital time	Outcome
1	1	4	3	3	2	4
2	1	7	2	3	3	1
3	1	4	3	3	2	4
4	2	6	3	3	2	4
5	2	5	2	3	3	4
6	2	5	3	3	3	4
7	1	6	3	3	3	3
8	1	5	2	3	2	4
9	1	6	3	3	3	4
10	2	7	3	3	2	1
11	1	6	3	3	3	4
12	1	5	2	3	3	4
13	2	4	3	3	3	3
14	1	4	3	3	3	4
15	1	2	2	3	1	2
16	1	3	2	3	3	4
17	2	3	2	3	1	4
18	2	4	3	3	2	4
19	2	1	2	3	2	4
20	2	5	3	3	3	4
21	1	5	3	3	3	4
22	1	1	2	3	3	4
23	1	6	3	3	3	4
24	1	5	3	3	3	3
25	1	4	3	3	3	4
26	1	2	2	3	2	3
27	1	6	3	3	3	4
28	2	4	3	3	2	4
29	1	6	3	3	3	4
30	2	6	3	3	2	4
31	1	5	3	3	3	4
32	1	3	2	3	2	4
33	1	5	3	3	3	4
34	2	7	3	3	3	4
35	1	5	2	3	3	4
36	2	5	2	3	2	4
37	2	3	3	3	3	4
38	1	6	3	3	3	3
39	2	2	3	3	2	3
40	2	4	3	3	3	4
41	1	6	3	3	3	3
42	1	5	3	3	3	3
43	2	6	2	3	2	1
44	1	5	3	3	3	3
45	1	6	3	3	3	1
46	1	3	2	3	3	3
47	2	2	2	3	3	3

48	2	6	3	3	3	3
49	1	6	2	3	3	3
50	1	7	3	3	3	3
51	2	5	3	3	2	3
52	1	6	3	3	2	3
53	2	4	3	3	2	4
54	1	2	2	3	2	4
55	1	5	3	3	3	4
56	2	2	2	3	3	3
57	2	6	2	3	3	4
58	2	6	3	3	2	3
59	2	5	3	3	2	3
60	1	6	3	3	2	4
61	1	5	2	3	2	1
62	2	3	2	3	3	4
63	1	3	2	3	3	4
64	1	3	2	3	2	3
65	1	5	3	3	2	4
66	1	6	3	3	3	4
67	1	4	3	3	2	4
68	1	5	2	3	3	2
69	2	3	2	3	2	3
70	1	5	3	3	3	4
71	1	5	3	3	3	4
72	1	4	3	3	2	3
73	1	5	3	3	2	4
74	2	7	3	3	2	4
75	2	6	3	3	3	4
76	1	6	2	3	3	3
77	2	3	2	3	2	1
78	2	6	3	3	3	3
79	2	5	3	3	3	3
80	1	2	3	3	2	4
81	1	6	3	3	2	4
82	2	7	3	3	3	4
83	2	6	3	3	3	4
84	1	5	3	3	2	4
85	1	5	3	3	2	4
86	2	6	3	3	2	4
87	2	4	3	3	2	3
88	2	3	2	3	1	3
89	1	6	3	3	3	3
90	1	5	3	3	2	4
91	2	2	2	3	3	3
92	2	7	2	3	3	3
93	1	4	2	3	3	4
94	1	3	3	3	3	4
95	2	5	3	3	3	4

96	2	6	3	3	3	4
97	1	6	3	3	3	3
98	1	4	3	3	3	4
99	2	7	3	3	3	3
100	2	7	3	3	3	3
101	2	4	3	3	2	4
102	1	6	3	3	2	3
103	2	5	3	3	3	4
104	2	6	3	3	2	4
105	1	7	3	3	3	4
106	1	5	3	3	1	4
107	2	3	3	3	3	4
108	1	6	3	3	3	4
109	1	7	3	3	3	4
110	2	6	3	3	3	2
111	1	4	3	3	3	3
112	2	6	3	3	2	4
113	1	5	3	3	3	4
114	1	3	2	3	3	4
115	1	6	3	3	3	4
116	1	6	3	3	3	4
117	2	7	3	3	3	2
118	1	2	2	3	3	3
119	1	5	3	3	2	4
120	2	2	2	3	2	4
121	1	5	3	3	2	3
122	1	6	3	3	3	4
123	1	7	3	3	3	4
124	1	5	3	3	3	4
125	1	2	2	3	2	3
126	2	5	3	3	3	3
127	1	5	3	3	3	1
128	1	5	2	3	2	3
129	2	3	3	3	3	4
130	2	2	2	3	3	4
131	1	3	3	3	2	4
132	2	5	3	3	3	4
133	2	2	2	3	3	4
134	2	6	3	3	3	4
135	1	6	3	3	3	4
136	2	4	3	3	3	4
137	1	5	3	3	4	4
138	2	5	3	3	2	1
139	1	6	3	3	3	3
140	2	5	3	3	3	4
141	2	4	3	3	2	4
142	1	7	3	3	2	4
143	2	6	3	3	2	1

144	1	7	3	3	3	3
145	1	4	3	3	2	3
146	2	5	3	3	3	4
147	1	5	3	3	3	4
148	1	6	3	3	3	4
149	1	2	2	3	2	3
150	2	7	3	3	2	1
151	2	3	2	3	2	2
152	1	5	3	3	2	4
153	2	5	3	3	3	4
154	1	5	3	3	3	4
155	1	6	3	3	3	4
156	2	3	3	3	2	3
157	1	6	3	3	2	4
158	1	4	3	3	3	4
159	1	7	3	3	2	4
160	2	4	3	3	4	3
161	2	7	3	3	3	4
162	2	2	2	3	3	4
163	2	6	3	3	3	4
164	1	4	2	3	3	3
165	1	4	3	3	3	4
166	1	3	2	3	3	3
167	1	5	3	3	2	3
168	1	2	2	3	3	2
169	1	3	3	3	3	3
170	1	5	3	3	3	3
171	1	6	3	3	2	4
172	2	4	3	3	2	3
173	1	4	2	3	3	4
174	1	6	3	3	3	4
175	1	4	3	3	2	4
176	1	7	3	3	2	4
177	1	4	3	3	4	4
178	2	4	3	3	3	4
179	1	6	3	3	3	4
180	1	4	3	3	3	4
181	2	6	2	3	2	3
182	1	7	3	3	3	3
183	2	6	3	3	3	3
184	2	7	3	3	3	4
185	1	4	3	3	3	4
186	2	6	3	3	2	4
187	2	7	3	3	2	4
188	2	5	3	3	3	4
189	2	6	3	3	2	3
190	1	6	3	3	2	4
191	1	4	3	3	2	4

192	2	4	2	3	2	4
193	1	7	3	3	4	4
194	2	5	3	3	2	3
195	2	5	3	3	3	3
196	2	4	3	3	3	3
197	2	5	3	3	3	4
198	2	5	3	3	3	4
199	2	5	3	3	3	3
200	1	6	3	3	3	3
201	1	6	3	3	3	4
202	2	3	2	3	4	3
203	1	6	3	3	3	4
204	1	3	2	3	3	4
205	2	7	3	3	3	4
206	2	7	3	3	3	4
207	1	1	2	3	3	2
208	1	7	3	3	3	4
209	2	6	2	3	2	3
210	2	7	3	3	3	4
211	1	6	3	3	2	4
212	2	4	3	3	2	4
213	1	6	3	3	2	1
214	2	6	3	3	3	4
215	2	6	3	3	3	4
216	2	5	3	3	3	3
217	1	4	3	3	2	4
218	1	1	2	3	2	3
219	1	5	3	3	3	3
220	1	5	3	3	3	4
221	1	5	3	3	3	4
222	1	4	3	3	2	3
223	1	5	3	3	2	4
224	1	5	3	3	3	4
225	1	7	2	3	3	4
226	2	2	3	3	2	4
227	2	6	3	3	3	3
228	2	7	3	3	2	3
229	1	6	3	3	2	3
230	2	4	3	3	3	3
231	2	4	3	3	3	3
232	1	6	3	3	3	4
233	1	5	3	3	3	4
234	1	6	3	3	3	4
235	2	6	3	3	3	4
236	1	5	2	3	3	3
237	1	6	3	3	3	3
238	1	4	3	3	3	3
239	1	4	2	3	2	2

240	2	2	2	3	3	3
241	2	6	3	3	3	4
242	2	6	3	3	2	3
243	2	4	3	3	3	4
244	1	2	2	3	2	3
245	2	7	3	3	2	2
246	2	3	2	3	3	2
247	2	2	2	3	2	4
248	1	5	2	3	4	3
249	1	6	3	3	3	4
250	2	5	3	3	3	3
251	2	4	3	3	3	3
252	2	4	3	3	2	3
253	2	7	2	3	2	3
254	1	5	3	3	3	4
255	1	4	2	3	3	4
256	1	6	3	3	3	4
257	2	6	2	3	3	4
258	2	5	3	3	3	4
259	1	2	2	3	3	4
260	1	7	3	3	3	4
261	2	7	3	3	3	4
262	1	6	3	3	3	4
263	1	7	3	3	3	3
264	1	6	3	3	3	3
265	2	4	2	3	3	4
266	1	4	3	3	2	4
267	1	6	3	3	3	4
268	2	5	3	3	3	2
269	1	5	3	3	3	2
270	1	5	3	3	3	4
271	1	7	2	3	2	2
272	1	5	3	3	3	4
273	2	4	3	3	2	4
274	1	5	3	3	2	3
275	1	7	3	3	3	4
276	1	2	2	3	3	4
277	1	3	3	3	3	4
278	1	6	3	3	2	4
279	1	3	2	3	3	4
280	1	7	3	3	3	3
281	1	2	2	3	2	3
282	1	6	2	3	3	3
283	2	5	3	3	2	4
284	1	5	3	3	3	1
285	2	6	3	3	3	3
286	1	7	3	3	2	4
287	1	6	3	3	2	4

288	1	6	3	3	3	4
289	1	3	2	3	2	3
290	2	6	3	3	2	4
291	1	4	3	3	3	4
292	2	4	3	3	3	3
293	2	6	3	3	2	3
294	2	6	3	3	3	3
295	2	6	3	3	2	3
296	2	5	3	3	3	3
297	2	5	3	3	2	4
298	1	6	3	3	3	3
299	1	7	3	3	3	1
300	1	4	3	3	4	4
301	2	7	3	3	3	1
302	1	4	3	3	3	3
303	2	2	2	3	3	3
304	2	6	2	3	3	4
305	2	2	2	3	1	3
306	2	5	3	3	3	4
307	1	5	3	3	2	3
308	2	6	3	3	3	4
309	1	5	3	3	3	4
310	2	5	3	3	3	4
311	2	2	2	3	3	3
312	2	6	3	3	2	4
313	2	6	2	3	3	4
314	1	2	2	3	4	3
315	2	6	3	3	2	3
316	1	6	3	3	3	3
317	2	5	2	3	3	3
318	2	7	3	3	2	3
319	2	6	2	3	3	4
320	2	7	3	3	2	1
321	1	2	2	3	3	3
322	1	6	2	3	2	4
323	1	5	3	3	2	4
324	1	4	3	3	2	2
325	2	3	3	3	3	4
326	1	2	2	3	3	3
327	1	7	3	3	2	4
328	1	6	3	3	3	4
329	2	5	2	3	3	4
330	2	7	3	3	3	3
331	2	2	2	3	3	3
332	2	5	3	3	2	3
333	1	5	3	3	3	3
334	1	4	3	3	4	3
335	1	6	3	3	3	4

336	1	6	3	3	3	4
337	1	3	3	3	2	4
338	1	4	2	3	3	2
339	1	5	3	3	2	4
340	1	3	3	3	3	4
341	1	7	3	3	2	4
342	1	3	2	3	3	4
343	1	4	3	3	2	4
344	2	5	3	3	4	4
345	1	5	3	3	2	4
346	2	4	2	3	3	3
347	1	6	2	3	3	3
348	2	5	2	3	2	4
349	1	6	3	3	3	4
350	1	7	2	3	3	4
351	1	6	3	3	3	4
352	1	5	2	3	3	4
353	1	6	3	3	3	4
354	1	6	3	3	2	4
355	2	2	2	3	3	4
356	1	6	3	3	3	4
357	2	7	3	3	3	4
358	2	7	3	3	3	4
359	1	2	2	3	3	4
360	1	5	3	3	3	4
361	2	5	2	3	2	4
362	2	7	3	3	2	4
363	1	4	3	3	3	4
364	1	7	3	3	2	4
365	2	7	3	3	2	4
366	1	6	3	3	3	4
367	1	6	3	3	3	4
368	1	6	3	3	2	4
369	2	4	3	3	3	3
370	1	6	3	3	3	4
371	1	6	3	3	3	4
372	1	6	3	3	3	4
373	2	5	3	3	3	4
374	1	6	3	3	4	3
375	1	3	3	3	4	3
376	2	6	3	3	4	4
377	1	6	3	3	4	2
378	1	5	3	3	4	1
379	1	4	3	3	4	4
380	1	3	3	3	4	3
381	1	4	3	3	4	3
382	2	6	3	3	4	3
383	1	6	3	3	4	3

384	2	6	3	3	4	3
385	1	7	3	3	4	3
386	2	7	3	3	4	3
387	1	5	3	3	4	3
388	1	2	2	3	4	4
389	1	6	3	3	4	4
390	1	6	3	3	4	1
391	1	5	3	3	4	4
392	1	5	3	3	4	4
393	1	3	3	3	4	3
394	2	7	3	3	4	3
395	2	6	3	3	4	3
396	1	5	3	3	4	4
397	2	6	3	3	4	4
398	1	2	2	3	4	3
399	2	6	3	3	4	3
400	1	7	3	3	4	3
401	1	5	3	3	4	4
402	1	2	3	3	4	3
403	1	3	3	3	4	2
404	1	7	3	3	4	4
405	2	5	3	3	4	4
406	1	3	3	3	4	4
407	1	2	2	3	4	4
408	1	3	3	3	4	3
409	2	6	3	3	4	3
410	1	5	3	3	4	4
411	1	6	3	3	4	4
412	1	6	3	3	4	4
413	1	5	3	3	4	4
414	1	6	3	3	4	4
415	1	3	3	3	4	4
416	1	4	3	3	4	3
417	1	6	3	3	4	4
418	2	7	3	3	4	4
419	2	4	3	3	3	3
420	1	6	3	3	3	3
421	2	7	3	3	3	4
422	2	6	3	3	3	4
423	2	4	3	3	2	3
424	1	5	3	3	3	4
425	1	6	3	3	2	4
426	1	6	3	3	2	4
427	1	3	3	3	2	4
428	1	6	3	3	2	1
429	1	7	3	3	3	4
430	1	7	3	3	3	3
431	1	4	3	3	2	4

432	1	6	3	3	3	3
433	2	3	3	3	2	3
434	1	5	3	3	3	3
435	1	6	3	3	3	3
436	2	7	3	3	3	4
437	1	6	3	3	2	3
438	1	6	3	3	3	4
439	2	5	3	3	3	4
440	1	5	3	3	2	2
441	1	6	3	3	3	4
442	1	5	3	3	1	3
443	1	7	3	3	3	3
444	1	6	3	3	3	3
445	2	7	3	3	3	4
446	1	6	3	3	1	3
447	1	5	3	3	3	4
448	2	5	3	3	3	4
449	1	1	2	3	1	3
450	2	5	3	3	3	4
451	1	6	3	3	1	4
452	1	3	3	3	3	4
453	1	4	2	3	3	4
454	1	5	3	3	3	4
455	1	7	3	3	2	4
456	2	5	3	3	3	4
457	1	4	3	3	3	4
458	2	5	3	3	3	4
459	2	6	3	3	2	4
460	1	4	3	3	3	3
461	1	4	3	3	3	4
462	1	6	3	3	3	4
463	1	5	3	3	3	4
464	1	6	3	3	3	4
465	1	7	3	3	3	4
466	1	6	3	3	2	4
467	1	3	2	3	2	4
468	1	4	3	3	4	3
469	1	3	2	3	4	4
470	1	4	3	3	4	4
471	1	4	3	3	4	3
472	2	2	2	3	4	2
473	2	5	3	3	4	3
474	1	2	2	3	4	4
475	1	5	3	3	4	4
476	2	7	3	3	4	3
477	1	2	2	3	4	3
478	2	4	3	3	4	4
479	1	5	3	3	4	2

480	1	4	3	3	4	4
481	1	6	3	3	4	3
482	1	7	3	3	4	3
483	1	5	3	3	4	4
484	1	5	3	3	4	4
485	2	7	3	3	4	4
486	2	2	2	3	4	3
487	1	4	2	3	4	4
488	1	3	3	3	4	4
489	1	3	3	3	4	4
490	1	6	3	3	4	2
491	2	3	2	3	4	4
492	2	7	3	3	4	4
493	1	5	3	3	4	4
494	1	5	3	3	4	4
495	2	2	2	3	4	3
496	1	7	3	3	4	3
497	2	6	3	3	4	3
498	1	5	3	3	4	4
499	1	6	2	3	4	4
500	2	3	3	3	4	3
501	1	4	3	3	4	3
502	1	6	3	3	4	4
503	1	6	3	3	4	4
504	2	6	3	3	4	4
505	1	6	3	3	4	4
506	1	3	3	3	4	4
507	2	7	3	3	4	4
508	2	5	3	3	4	4
509	1	3	2	3	4	4
510	1	3	3	3	4	3
511	2	4	3	3	4	4
512	1	7	3	3	4	3
513	1	5	3	3	4	4
514	2	3	2	2	3	4
515	1	6	3	2	3	4
516	1	5	3	2	3	4
517	2	2	2	2	3	4
518	1	4	3	2	2	4
519	1	6	3	2	3	3
520	1	6	3	2	3	4
521	1	5	3	2	3	4
522	1	6	3	2	3	4
523	2	6	3	2	3	3
524	2	6	2	2	2	4
525	2	6	3	2	3	4
526	2	3	3	2	3	2
527	2	6	3	2	4	4

528	2	7	3	2	3	3
529	2	2	3	2	3	4
530	1	6	3	2	2	3
531	1	5	3	2	4	3
532	1	6	3	2	4	4
533	2	1	2	2	3	4
534	1	6	3	2	4	3
535	1	4	3	2	4	4
536	1	6	3	2	3	3
537	2	5	3	2	3	4
538	2	5	3	2	4	3
539	2	6	3	2	3	4
540	2	4	3	2	3	4
541	1	6	3	2	3	4
542	2	4	3	2	3	4
543	1	7	3	2	3	3
544	2	6	3	2	3	3
545	1	7	2	2	2	4
546	2	6	3	2	3	4
547	1	7	3	2	3	3
548	1	6	3	2	2	1
549	1	3	3	2	3	3
550	1	6	3	2	4	4
551	2	2	3	2	3	4
552	1	6	3	2	4	3
553	1	6	3	2	3	3
554	1	4	3	2	4	3
555	2	6	3	2	3	4
556	1	5	2	2	3	4
557	2	6	3	2	4	1
558	1	6	3	2	4	3
559	2	7	3	2	4	3
560	2	5	3	2	4	4
561	1	6	3	2	4	3
562	2	5	3	2	4	4
563	1	3	3	2	4	4
564	2	6	3	2	4	3
565	2	6	3	2	4	4
566	1	6	3	2	4	4
567	2	2	2	2	4	4
568	2	5	3	2	4	4
569	1	5	3	2	4	3
570	2	5	3	2	4	2
571	1	7	3	2	4	3
572	1	6	3	2	4	4
573	1	6	3	2	4	3
574	1	1	2	2	4	4
575	2	3	3	2	4	3

576	2	6	3	2	4	3
577	2	5	3	2	4	4
578	1	6	3	2	4	4
579	2	6	3	2	4	4
580	1	3	3	2	4	4
581	2	3	3	2	2	4
582	1	4	3	2	3	4
583	1	5	3	2	1	1
584	2	4	3	2	3	4
585	1	5	3	2	3	4
586	2	5	3	2	3	3
587	1	4	3	2	3	4
588	2	7	3	2	3	4
589	1	3	2	2	3	2
590	2	5	3	2	3	4
591	1	3	3	2	3	4
592	2	6	3	2	3	4
593	1	6	3	2	3	4
594	1	6	3	2	3	4
595	1	4	3	2	3	4
596	1	7	3	2	3	4
597	2	5	3	2	3	4
598	1	6	3	2	3	3
599	1	6	3	2	3	4
600	1	2	3	2	3	4
601	1	6	3	2	3	3
602	1	7	3	2	3	3
603	1	4	3	2	3	4
604	1	6	3	2	3	4
605	2	7	3	2	3	4
606	1	4	3	2	3	4
607	1	6	3	2	3	3
608	1	3	3	2	2	4
609	2	6	3	2	3	4
610	2	4	3	2	4	4
611	2	3	2	2	4	4
612	1	5	3	2	4	2
613	1	5	3	2	4	4
614	1	3	3	2	4	4
615	1	7	3	2	4	3
616	2	5	3	2	4	4
617	1	7	3	2	4	4
618	2	6	3	2	4	3
619	1	4	3	2	4	3
620	1	5	2	2	4	3
621	2	4	3	2	4	3
622	1	4	3	2	4	4
623	1	3	2	2	4	4

624	2	5	3	2	4	3
625	2	2	2	2	4	3
626	1	6	3	2	4	3
627	2	2	2	2	4	3
628	1	4	2	2	4	3
629	1	7	3	2	4	3
630	1	7	3	2	4	4
631	1	6	3	2	4	4
632	2	4	3	2	4	4
633	1	6	3	2	4	4
634	2	7	3	2	4	3
635	1	5	3	2	4	3
636	2	3	3	2	4	4
637	2	7	3	2	4	4
638	2	4	3	2	4	2
639	1	5	3	2	4	4
640	1	6	3	2	4	4
641	1	2	2	2	4	4
642	1	4	3	2	4	4
643	1	4	3	2	4	4
644	2	6	3	2	4	4
645	1	5	3	2	4	4
646	1	6	3	2	4	4
647	1	3	3	2	4	3
648	1	7	3	2	4	4
649	1	6	3	2	4	4
650	1	2	2	2	4	4
651	1	6	3	2	4	4
652	2	6	3	2	4	4
653	2	6	3	2	4	4
654	1	3	3	2	4	4
655	1	3	3	2	4	3
656	2	3	3	2	4	4
657	2	5	3	2	4	4
658	2	4	3	2	4	4
659	1	2	2	2	4	4
660	2	6	3	2	4	2
661	2	6	3	2	4	4
662	1	6	3	2	4	2
663	1	2	3	2	4	4
664	2	7	3	2	4	4
665	1	5	3	2	4	3
666	1	3	3	2	4	4
667	1	3	3	2	4	3
668	1	7	3	2	4	3
669	1	5	3	2	4	4
670	2	6	3	2	4	4
671	1	6	3	2	4	4

672	2	6	3	2	4	3
673	1	7	3	2	4	4
674	1	5	3	2	4	3
675	1	7	3	2	4	4
676	1	6	3	2	4	4
677	1	7	3	2	4	4
678	1	6	3	2	4	4
679	1	2	2	2	4	3
680	1	4	3	2	4	4
681	1	5	3	2	4	4
682	1	7	3	2	4	4
683	2	6	3	2	4	4
684	2	3	3	2	4	3
685	2	5	3	2	4	4
686	1	6	3	2	4	4
687	1	5	3	2	4	4
688	1	5	3	1	3	3
689	1	3	2	1	3	4
690	1	6	3	1	4	2
691	1	4	3	1	4	4
692	1	7	3	1	4	2
693	2	5	3	1	3	4
694	1	3	3	1	3	4
695	2	4	3	1	3	4
696	1	4	3	1	3	3
697	2	4	3	1	2	4
698	1	3	2	1	3	4
699	2	2	2	1	4	4
700	2	5	3	1	4	3
701	1	3	3	1	4	4
702	1	4	3	1	3	4
703	1	5	3	1	3	4
704	1	5	3	1	3	4
705	2	6	3	1	3	3
706	2	3	3	1	4	4
707	2	4	3	1	4	4
708	1	5	2	1	4	4
709	1	5	3	1	4	2
710	1	6	2	1	4	4
711	2	5	3	1	4	4
712	1	7	3	1	4	3
713	2	6	2	1	4	3
714	2	1	2	1	4	4
715	2	5	3	1	4	4
716	1	6	3	1	4	4
717	1	4	3	1	4	3
718	1	5	3	1	4	4
719	1	4	3	1	4	3

720	2	2	2	1	4	4
721	1	6	3	1	4	4
722	2	1	2	1	4	2
723	1	4	3	1	4	3
724	2	4	3	1	4	3
725	2	4	3	1	4	4
726	1	6	3	1	4	4
727	2	4	3	1	4	4
728	1	5	3	1	4	3
729	1	3	2	1	4	3
730	1	5	3	1	4	3
731	1	6	3	1	4	2
732	1	5	3	1	4	3
733	2	5	3	1	4	3
734	1	4	3	1	4	3
735	1	3	3	1	4	4
736	1	4	3	1	4	3
737	1	7	3	1	4	4
738	2	1	2	1	4	4
739	2	6	3	1	4	3
740	1	6	3	1	4	4
741	1	4	3	1	4	3
742	1	5	3	1	4	3
743	1	6	3	1	4	3
744	1	5	2	1	4	3
745	2	5	2	1	4	4
746	2	6	3	1	4	3
747	2	5	3	1	4	3
748	1	7	2	1	4	4
749	1	4	3	1	4	3
750	1	2	2	1	4	2
751	1	3	2	1	4	4
752	2	7	3	1	4	3
753	1	5	3	1	4	4
754	1	7	3	1	4	4
755	2	6	3	1	4	4
756	1	3	2	1	4	3
757	1	6	3	1	4	3
758	2	5	3	1	4	4
759	2	3	3	1	4	4
760	2	4	3	1	4	2
761	1	5	3	1	4	4
762	1	6	3	1	4	4
763	1	3	3	1	4	4
764	1	5	3	1	4	4
765	1	3	3	1	4	3
766	2	6	3	1	4	4
767	1	3	3	1	4	3

768	1	6	3	1	4	4
769	1	7	3	1	4	3
770	1	5	3	1	4	3
771	1	5	3	1	4	4
772	2	5	3	1	4	4
773	1	2	2	1	4	2
774	2	6	3	1	4	4
775	2	7	3	1	4	4
776	1	3	3	1	4	4
777	1	6	3	1	4	4
778	1	2	2	1	4	3
779	1	4	3	1	4	4
780	2	4	3	1	4	4
781	1	6	3	1	4	4
782	1	6	3	1	4	3
783	2	6	3	1	4	4
784	1	6	3	1	4	4
785	1	1	2	1	4	2
786	2	5	3	1	4	4
787	1	4	3	1	4	3
788	2	5	3	1	4	4
789	1	5	3	1	4	4
790	1	4	3	1	4	4
791	1	3	3	1	4	4
792	1	6	3	1	4	4
793	2	5	3	1	4	4
794	1	5	3	1	4	4
795	2	6	3	1	4	4
796	2	4	3	1	4	4
797	2	6	3	1	4	4
798	2	6	3	1	4	4
799	2	2	2	1	4	4
800	2	3	3	1	4	4
801	2	4	3	1	4	4
802	2	5	3	1	4	3
803	1	3	3	1	4	3
804	2	4	3	1	4	4
805	1	6	3	1	4	4
806	1	5	3	1	4	3
807	1	6	3	1	4	4
808	2	4	3	1	4	2
809	1	6	3	1	4	2
810	2	5	3	1	4	4
811	2	1	2	1	4	4
812	1	4	3	1	4	4
813	2	6	3	1	4	2
814	2	4	3	1	4	3
815	2	5	3	1	4	4

816	1	5	3	1	4	3
817	2	6	3	1	4	3
818	1	3	3	1	4	4
819	1	4	3	1	4	3
820	2	6	3	1	4	2
821	1	3	2	1	4	4
822	1	6	3	1	4	3
823	1	6	3	1	4	4
824	1	3	3	1	4	3
825	1	4	3	1	4	4
826	1	5	3	1	4	4
827	1	3	3	1	4	3
828	1	5	3	1	4	4
829	2	4	3	1	4	3
830	1	6	3	1	4	3
831	2	6	3	1	4	3
832	1	3	3	1	4	4
833	1	6	3	1	4	4
834	2	2	2	1	4	4
835	1	4	3	1	4	4
836	2	2	2	1	4	4
837	2	6	3	1	4	4
838	1	5	3	1	4	4
839	1	6	3	1	4	4
840	1	5	3	1	4	3
841	1	3	3	1	4	3
842	2	5	3	1	4	4
843	1	2	2	1	4	4
844	1	7	3	1	4	4
845	2	6	3	1	4	4
846	1	2	2	1	4	4
847	2	1	2	1	4	4
848	1	2	2	1	4	4
849	1	5	3	1	4	4
850	2	2	2	1	4	4
851	2	5	3	1	4	4
852	2	4	3	1	4	2
853	2	2	2	1	3	3
854	1	2	2	1	3	3
855	1	4	3	1	3	3
856	1	2	2	1	3	4
857	1	2	2	1	3	4
858	1	3	3	1	3	4
859	2	3	3	1	3	3
860	1	6	3	1	3	4
861	2	7	3	1	3	4
862	1	2	2	1	3	4
863	1	3	2	1	3	4

864	1	5	2	1	3	4
865	1	7	3	1	3	4
866	2	4	3	1	3	4
867	2	5	3	1	3	4
868	1	1	2	1	3	4
869	1	2	3	1	3	4
870	2	4	3	1	3	4
871	1	1	2	1	3	3
872	1	6	3	1	3	3
873	1	4	3	1	4	3
874	1	5	3	1	4	4
875	1	3	2	1	4	3
876	1	3	3	1	4	3
877	1	2	2	1	4	4
878	1	3	3	1	4	3
879	1	6	3	1	4	4
880	1	4	3	1	4	4
881	1	3	3	1	4	4
882	1	7	3	1	4	4
883	1	6	3	1	4	4
884	1	5	3	1	4	3
885	1	6	3	1	4	3
886	1	5	3	1	4	4
887	1	4	3	1	4	3
888	1	5	3	1	4	4
889	2	4	3	1	4	4
890	1	3	3	1	4	4
891	1	4	3	1	4	3
892	1	3	3	1	4	2
893	1	6	3	1	4	4
894	1	4	3	1	4	3
895	2	5	3	1	4	3
896	1	4	3	1	4	4
897	1	2	2	1	4	3
898	1	3	3	1	4	4
899	1	5	3	1	4	3
900	2	5	3	1	4	4
901	1	3	3	1	4	2
902	2	5	3	1	4	4
903	2	2	2	1	4	3
904	1	6	3	1	4	3
905	1	2	2	1	4	4
906	1	4	3	1	4	4
907	1	6	3	1	4	2
908	1	3	3	1	4	4
909	1	2	2	1	4	2
910	1	1	2	1	4	4
911	1	7	3	1	4	4

912	1	7	3	1	4	3
913	1	5	3	1	4	3
914	2	7	2	1	4	4
915	1	7	2	1	4	4
916	1	6	2	1	4	4
917	1	3	2	1	4	4
918	1	3	2	1	4	3
919	2	7	2	1	4	3
920	1	7	2	1	4	3
921	1	4	2	1	4	4
922	2	3	2	1	4	3
923	1	6	2	1	4	3
924	1	4	2	1	4	4
925	1	7	2	1	4	4
926	2	3	2	1	4	2
927	1	7	2	1	4	3
928	1	6	2	1	4	4
929	1	6	2	1	4	4
930	2	5	2	1	4	4
931	2	6	2	1	4	4
932	2	7	2	1	4	3
933	1	5	2	1	4	3
934	1	6	2	1	4	4
935	2	7	2	1	4	3
936	1	4	2	1	4	3
937	2	4	2	1	4	3
938	1	3	2	1	4	3
939	1	2	2	1	4	4
940	1	6	2	1	4	3
941	1	5	2	1	4	3
942	1	3	2	1	4	4
943	1	6	2	1	4	4
944	2	3	2	1	4	4
945	2	7	2	1	4	4
946	1	4	3	1	4	4
947	1	6	3	1	4	4
948	2	6	3	1	4	3
949	1	3	3	1	4	3
950	2	6	3	1	4	4
951	1	2	2	1	4	3
952	2	3	2	1	4	4
953	2	4	3	1	4	4
954	2	3	3	1	4	4
955	1	1	2	1	4	4
956	1	7	3	1	4	3
957	1	2	2	1	4	4
958	1	7	3	1	4	3
959	1	5	3	1	4	3

960	2	3	3	1	4	4
961	2	3	3	1	4	3
962	2	2	2	1	4	3
963	1	2	2	1	4	4
964	2	3	3	1	4	4
965	2	4	3	1	4	4
966	1	1	2	1	4	4
967	1	5	3	1	4	4
968	2	1	2	1	4	4
969	1	4	3	1	4	4
970	1	5	3	1	4	4
971	2	1	2	1	4	3
972	2	5	3	1	4	3
973	2	3	2	1	4	4
974	1	5	3	1	4	4
975	1	1	2	1	4	4
976	1	4	3	1	4	4
977	1	6	3	1	4	4
978	2	6	3	2	3	3
979	1	5	3	2	2	2
980	1	5	3	2	3	3
981	2	5	3	2	3	4
982	1	5	3	2	2	4
983	2	4	3	2	3	4
984	2	3	3	2	3	4
985	1	2	2	2	3	4
986	2	3	2	2	3	3
987	2	5	3	2	3	4
988	1	4	3	2	3	4
989	1	4	3	2	3	4
990	1	4	3	2	3	3
991	1	4	3	2	3	3
992	1	7	3	2	3	4
993	1	5	3	2	3	4
994	2	7	3	2	3	3
995	1	7	3	2	3	4
996	1	4	3	2	3	4
997	1	7	3	2	2	2
998	1	6	3	2	3	4
999	2	7	3	2	2	4
1000	2	6	3	2	3	3
1001	1	3	3	2	3	1
1002	1	5	3	2	3	3
1003	1	4	2	2	3	4
1004	2	5	3	2	2	4
1005	1	4	3	2	3	3
1006	1	5	3	2	3	3
1007	1	6	3	2	3	4

1008	1	6	3	2	3	3
1009	1	4	3	2	3	4
1010	2	4	3	2	3	3
1011	1	5	3	2	3	4
1012	2	6	3	2	2	1
1013	1	6	3	2	3	3
1014	1	2	3	2	3	2
1015	1	3	3	2	3	3
1016	1	5	3	2	3	4
1017	1	3	2	2	3	4
1018	1	7	2	2	3	4
1019	1	5	2	2	2	3
1020	1	7	2	2	3	3
1021	1	7	2	2	2	3
1022	2	5	2	2	3	3
1023	1	1	2	2	2	4
1024	1	6	2	2	3	4
1025	1	6	2	2	3	4
1026	2	7	2	2	3	2
1027	1	4	2	2	3	3
1028	1	1	2	2	3	1
1029	1	5	2	2	3	4
1030	1	7	2	2	2	4
1031	2	3	2	2	3	4
1032	1	5	2	2	3	3
1033	1	5	2	2	3	4
1034	1	4	2	2	2	3
1035	2	5	3	2	3	4
1036	1	6	3	2	3	4
1037	2	5	3	2	3	4
1038	1	5	3	2	3	3
1039	2	5	3	2	3	4
1040	1	4	3	2	3	3
1041	1	4	3	2	3	4
1042	1	1	2	2	3	4
1043	1	6	3	2	2	3
1044	2	4	3	2	3	4
1045	2	4	3	2	3	4
1046	2	4	3	2	3	3
1047	1	6	3	2	3	4
1048	2	3	1	2	3	4
1049	1	3	3	2	3	4
1050	2	5	3	2	4	4
1051	1	6	3	2	4	4
1052	1	3	3	2	4	4
1053	1	5	3	2	4	4
1054	1	6	3	2	4	4
1055	1	5	3	2	4	3

1056	2	5	3	2	4	4
1057	1	6	3	2	4	4
1058	2	7	3	2	4	4
1059	1	6	3	2	4	3
1060	2	3	3	2	4	4
1061	2	5	3	2	4	3
1062	1	6	3	2	4	4
1063	1	4	3	2	4	4
1064	1	2	2	2	4	4
1065	1	6	3	2	4	2
1066	1	6	3	2	4	4
1067	1	5	2	2	4	4
1068	2	7	2	2	4	3
1069	2	6	2	2	4	3
1070	1	6	2	2	4	3
1071	1	7	2	2	4	3
1072	1	5	2	2	4	4
1073	1	2	2	2	4	4
1074	1	5	2	2	4	4
1075	1	6	2	2	4	4
1076	1	6	2	2	4	2
1077	1	7	2	2	4	4
1078	1	1	2	2	4	4
1079	1	4	2	2	4	3
1080	2	4	2	2	4	4
1081	1	2	2	2	4	4
1082	2	5	2	2	4	2
1083	1	7	2	2	4	3
1084	2	2	2	2	4	4
1085	2	5	2	2	4	3
1086	2	5	2	2	4	3
1087	1	2	2	2	4	4
1088	2	4	3	2	4	4
1089	2	6	3	2	4	4
1090	2	5	3	2	4	4
1091	1	2	2	2	4	4
1092	2	4	3	2	4	4
1093	2	5	3	2	4	4
1094	2	6	3	2	4	4
1095	2	4	2	3	3	4
1096	1	6	3	3	2	3
1097	1	6	3	3	3	4
1098	1	5	3	3	3	3
1099	2	3	3	3	2	4
1100	1	4	3	3	3	4
1101	2	5	3	3	2	3
1102	2	7	3	3	2	4
1103	1	5	3	3	3	1

1104	1	5	3	3	2	4
1105	1	6	3	3	3	4
1106	1	6	3	3	1	1
1107	2	5	3	3	1	3
1108	2	7	3	3	3	1
1109	2	6	3	3	3	3
1110	1	2	2	3	3	3
1111	1	5	3	3	3	3
1112	2	7	3	3	3	3
1113	2	1	2	3	3	2
1114	1	6	3	3	3	3
1115	2	2	2	3	3	4
1116	2	6	3	3	2	1
1117	1	6	3	3	3	4
1118	2	5	3	3	3	4
1119	2	3	2	3	1	3
1120	1	5	3	3	2	4
1121	1	6	3	3	3	3
1122	1	4	3	3	3	4
1123	1	5	3	3	3	3
1124	1	7	3	3	3	4
1125	1	7	2	3	3	3
1126	2	4	2	3	3	3
1127	1	5	2	3	3	2
1128	1	6	2	3	3	4
1129	1	7	2	3	2	3
1130	2	7	2	3	3	4
1131	1	2	2	3	3	4
1132	1	5	2	3	2	4
1133	1	6	2	3	3	4
1134	1	5	2	3	3	4
1135	1	3	2	3	2	4
1136	2	4	2	3	3	4
1137	2	7	2	3	3	4
1138	2	7	2	3	3	4
1139	1	4	2	3	3	4
1140	2	7	2	3	3	4
1141	2	5	2	3	3	4
1142	1	2	2	3	3	3
1143	1	4	2	3	3	4
1144	1	7	2	3	3	3
1145	1	3	2	3	3	3
1146	1	6	2	3	2	4
1147	1	6	2	3	3	4
1148	1	5	2	3	2	3
1149	2	4	2	3	2	4
1150	1	6	2	3	2	4
1151	1	7	2	3	3	4

1152	2	5	2	3	3	3
1153	2	7	2	3	3	4
1154	1	6	2	3	3	4
1155	1	2	2	3	3	4
1156	2	5	3	3	2	3
1157	1	4	3	3	3	4
1158	1	4	3	3	2	4
1159	1	5	3	3	3	4
1160	2	3	3	3	3	4
1161	2	2	2	3	3	4
1162	2	6	3	3	3	4
1163	2	4	3	3	3	4
1164	1	7	3	3	3	4
1165	1	5	3	3	2	4
1166	1	6	3	3	2	4
1167	1	3	3	3	3	4
1168	1	6	3	3	3	4
1169	1	6	3	3	3	4
1170	1	5	3	3	3	4
1171	1	6	3	3	3	3
1172	1	5	3	3	3	4
1173	1	6	3	3	2	3
1174	2	4	3	3	3	4
1175	1	5	3	3	3	4
1176	2	6	3	3	2	4
1177	1	3	3	3	2	4
1178	1	4	3	3	3	4
1179	2	5	3	3	3	4
1180	1	4	3	3	2	4
1181	1	6	3	3	3	4
1182	1	7	3	3	3	4
1183	1	6	3	3	1	4
1184	2	6	3	3	3	4
1185	1	5	3	3	3	4
1186	2	7	3	3	3	4
1187	1	6	3	3	3	4
1188	2	6	3	3	3	4
1189	2	6	3	3	3	4
1190	1	2	2	3	2	4
1191	1	5	3	3	3	4
1192	2	6	3	3	1	4
1193	2	5	3	3	3	4
1194	2	5	3	3	3	4
1195	1	3	3	3	2	4
1196	1	4	3	3	3	4
1197	1	3	3	3	2	3
1198	1	6	3	3	3	4
1199	1	6	3	3	3	3

1200	2	7	3	3	3	4
1201	1	4	3	3	2	4
1202	1	6	3	3	2	4
1203	1	5	3	3	2	4
1204	1	1	2	3	2	4
1205	2	2	2	3	3	4
1206	2	5	3	3	3	4
1207	2	4	3	3	2	4
1208	1	4	3	3	3	4
1209	1	2	2	3	3	4
1210	1	6	3	3	3	4
1211	1	4	3	3	3	4
1212	1	4	3	3	3	3
1213	1	5	3	3	2	3
1214	1	3	3	3	3	4
1215	1	6	3	3	2	4
1216	1	7	3	3	3	4
1217	2	5	3	3	3	4
1218	2	3	3	3	3	4
1219	1	4	3	3	3	3
1220	1	7	3	3	3	4
1221	1	5	3	3	1	3
1222	1	6	3	3	2	4
1223	2	4	3	3	3	3
1224	2	6	3	3	3	4
1225	1	7	3	3	2	4
1226	2	4	3	3	2	3
1227	1	5	3	3	2	3
1228	2	4	3	3	3	4
1229	1	2	2	3	3	4
1230	1	5	3	3	2	4
1231	1	4	3	3	3	3
1232	1	7	3	3	2	4
1233	1	3	3	3	3	4
1234	2	2	2	3	3	4
1235	1	3	2	3	2	3
1236	1	6	3	3	2	4
1237	2	4	3	3	2	4
1238	2	6	3	3	3	4
1239	1	2	3	3	3	4
1240	1	5	3	3	3	4
1241	2	2	2	3	3	4
1242	1	6	3	3	2	4
1243	2	4	3	3	3	4
1244	2	5	3	3	3	4
1245	1	6	3	3	3	4
1246	2	5	3	3	2	4
1247	2	4	3	3	3	3

1248	2	4	3	3	1	4
1249	1	2	2	3	2	4
1250	1	5	3	3	3	4
1251	1	3	3	3	2	4
1252	1	5	3	3	3	4
1253	2	3	1	3	2	4
1254	1	6	3	3	2	4
1255	1	5	3	3	1	4
1256	1	7	3	3	3	4
1257	1	3	3	3	3	3
1258	1	4	3	3	3	4
1259	1	6	1	3	3	4
1260	1	4	3	3	3	4
1261	1	2	2	3	3	3
1262	1	5	3	3	2	4
1263	1	5	3	3	2	3
1264	1	6	3	3	3	4
1265	1	2	2	3	2	4
1266	1	3	3	3	3	4
1267	2	4	3	3	3	4
1268	2	3	2	3	3	4
1269	1	6	3	3	3	4
1270	1	3	3	3	3	4
1271	2	7	3	3	3	4
1272	1	4	1	3	2	4
1273	1	7	3	3	3	4
1274	1	3	3	3	2	4
1275	2	5	3	3	3	4
1276	2	2	3	3	2	4
1277	1	5	3	3	3	4
1278	1	6	3	3	2	4
1279	1	6	3	3	3	3
1280	1	2	2	3	3	4
1281	2	4	3	3	2	4
1282	1	7	3	3	3	3
1283	1	5	3	3	2	4
1284	2	6	3	3	3	4
1285	1	4	3	3	3	3
1286	1	6	3	3	3	4
1287	2	3	3	3	3	3
1288	1	6	3	3	3	4
1289	1	6	3	3	3	3
1290	1	5	3	3	3	4
1291	1	4	3	3	4	4
1292	1	7	2	3	4	4
1293	1	3	2	3	4	4
1294	2	4	3	3	4	4
1295	2	7	3	3	4	4

1296	2	6	3	3	4	4
1297	2	5	3	3	4	4
1298	1	7	3	3	4	4
1299	1	7	3	3	4	4
1300	1	3	2	3	4	4
1301	1	7	3	3	4	4
1302	1	4	3	3	4	4
1303	1	2	2	3	4	4
1304	2	6	3	3	4	3
1305	1	4	3	3	4	4
1306	2	6	3	3	4	4
1307	1	7	3	3	4	4
1308	1	2	2	3	4	4
1309	1	7	3	3	4	3
1310	1	5	3	3	3	4
1311	2	5	3	2	3	3
1312	2	3	2	3	1	4
1313	1	6	3	3	2	4
1314	1	2	2	3	3	3
1315	1	6	3	3	3	4
1316	1	6	3	3	3	3
1317	1	5	3	2	3	3
1318	1	2	2	3	3	3
1319	2	2	2	3	1	3
1320	1	5	3	1	3	3
1321	2	6	1	3	3	4
1322	1	5	3	3	3	4
1323	1	6	3	3	3	3
1324	1	7	3	3	2	4
1325	1	4	3	3	3	3
1326	1	3	3	3	1	3
1327	2	5	3	1	3	3
1328	1	4	3	3	3	3
1329	1	6	3	3	3	3
1330	1	5	3	3	1	4
1331	2	5	3	3	2	4
1332	1	7	3	3	2	4
1333	1	7	3	3	2	4
1334	1	5	3	2	3	4
1335	1	3	2	3	2	4
1336	1	3	3	3	3	4
1337	1	7	3	3	3	4
1338	2	3	2	3	2	4
1339	1	4	3	1	3	4
1340	2	7	3	3	3	4
1341	1	6	3	3	4	3
1342	2	7	3	3	4	3
1343	1	6	3	3	4	3

1344	2	2	2	1	4	3
1345	1	3	3	1	4	4
1346	1	2	2	1	4	3
1347	1	2	2	1	4	3
1348	1	2	2	1	4	4
1349	1	6	3	1	4	3
1350	1	2	2	1	4	4
1351	2	3	3	3	2	4
1352	1	4	3	3	3	3
1353	1	5	3	1	3	2
1354	1	7	3	3	1	3
1355	1	2	2	1	3	3
1356	2	6	3	2	3	4
1357	1	5	3	3	2	2
1358	2	5	3	3	2	2
1359	1	4	3	2	3	3
1360	2	6	3	3	3	3
1361	1	2	2	1	3	3
1362	1	5	3	1	4	1
1363	2	4	3	1	4	4
1364	1	5	3	1	4	3
1365	1	3	3	2	4	3
1366	1	5	3	1	4	2
1367	1	4	3	1	4	4
1368	1	4	3	1	4	4
1369	1	2	2	1	4	3
1370	1	4	3	2	4	3
1371	2	6	3	1	4	3
1372	1	5	3	3	3	3
1373	1	4	3	1	3	4
1374	1	3	3	2	2	4
1375	1	7	3	2	3	4
1376	1	3	3	2	3	3
1377	1	6	3	3	3	3
1378	2	7	3	2	3	4
1379	1	6	3	3	3	3
1380	2	5	3	1	4	4
1381	1	3	3	1	4	4
1382	1	2	2	2	4	4
1383	1	3	3	1	4	4
1384	2	5	3	3	3	3
1385	2	4	3	3	2	4
1386	2	3	3	3	2	4
1387	2	4	3	3	3	3
1388	2	3	3	3	2	3
1389	2	6	3	3	3	4
1390	2	3	2	2	2	4
1391	1	5	3	3	3	4

1392	1	2	2	1	3	4
1393	2	6	3	1	4	4
1394	1	2	2	1	4	4
1395	1	2	2	1	3	3
1396	1	2	2	2	2	3
1397	1	2	2	2	3	3
1398	1	5	3	2	3	3
1399	1	4	3	3	2	4
1400	1	2	2	1	3	4
1401	1	4	3	2	3	3
1402	1	3	2	3	3	4
1403	1	5	3	3	3	2
1404	1	2	2	3	3	3
1405	1	3	3	3	3	3
1406	1	3	3	2	3	4
1407	1	2	2	1	4	3
1408	1	2	2	1	4	3
1409	1	3	2	1	4	3
1410	1	3	2	1	4	3
1411	1	3	2	2	4	3
1412	1	3	3	2	3	3
1413	1	2	2	1	3	3
1414	2	5	3	3	3	2
1415	1	5	3	2	3	4
1416	2	2	2	3	3	3
1417	1	3	3	2	3	4
1418	1	4	3	2	3	4
1419	1	2	2	3	3	4
1420	1	4	3	1	4	3
1421	1	5	3	2	4	3
1422	2	2	2	3	4	3
1423	1	3	2	2	4	4
1424	2	4	3	1	4	3

LOS	Body region	FJI	SSTI	Destruction	CNSI	PC	TH	TOI
3	6	1	0	0	0	0	0	0
2	1	1	1	0	1	0	0	0
4	6	1	1	0	0	0	0	0
4	6	1	0	0	0	0	0	0
5	6	1	1	0	0	0	0	0
3	6	1	0	0	0	0	0	0
2	1	1	1	0	0	0	0	0
4	6	1	0	0	0	0	0	0
2	6	1	0	0	0	0	0	0
1	1	0	1	0	1	0	0	0
3	6	1	0	0	0	0	0	0
5	6	1	1	0	0	0	0	0
4	6	1	1	0	0	0	0	0
4	6	1	0	0	0	0	0	0
1	6	1	0	0	0	0	0	0
3	6	1	0	0	0	0	0	0
4	6	1	1	0	0	0	0	0
2	6	1	0	0	0	0	0	0
4	6	1	1	0	0	0	0	0
2	6	1	0	0	0	0	0	0
4	6	1	0	0	0	0	0	0
3	6	1	1	0	0	0	0	0
5	6	1	0	0	0	0	0	0
2	4	0	1	0	0	0	0	0
4	6	1	0	0	0	0	0	0
2	6	1	0	0	0	0	0	0
4	6	1	0	0	0	0	0	0
4	6	1	0	0	0	0	0	0
4	6	1	0	0	0	0	0	0
2	6	1	1	0	0	0	0	0
4	6	1	0	0	0	0	0	0
3	6	1	1	0	0	0	0	0
4	6	1	1	0	0	0	0	0
4	6	1	1	0	0	0	0	0
4	6	1	0	0	0	0	0	0
4	6	1	1	0	0	0	0	0
2	1	0	1	0	1	0	0	0
4	1	1	0	0	1	0	0	0
3	1	1	0	0	1	0	0	0
4	1	1	1	0	1	0	0	0
3	1	0	0	0	1	0	0	0
4	1	1	0	0	1	0	0	0
4	1	1	1	0	1	0	0	0
3	1	0	1	0	0	0	0	0
1	1	0	0	0	1	0	0	0
5	1	1	1	0	1	0	0	0
2	1	1	0	0	1	0	0	0

3	1	0	1	0	1	0	0	0
3	1	0	1	0	1	0	0	0
2	1	1	1	0	1	0	0	0
2	1	0	1	0	1	0	0	0
1	1	0	1	0	0	0	0	0
4	6	1	0	0	0	0	0	0
5	2	1	1	0	1	0	0	0
3	3	0	0	0	0	0	0	1
3	1	0	1	0	1	0	0	0
5	1	1	1	0	0	0	0	0
3	2	1	0	0	1	1	0	0
2	1	0	1	0	0	0	0	0
4	1	1	1	0	1	0	0	0
2	1	1	0	0	1	0	0	0
4	6	1	0	0	0	0	0	0
4	6	1	0	0	0	0	0	0
1	6	1	1	0	0	0	0	0
2	6	1	0	0	0	0	0	0
4	6	1	0	0	0	0	0	0
2	1	1	1	0	1	0	0	0
3	1	0	1	0	1	0	0	0
2	1	0	1	0	1	0	0	0
4	1	1	1	0	1	0	0	0
3	6	1	0	0	0	0	0	0
2	6	1	0	0	0	0	0	0
4	6	1	1	0	0	0	0	0
4	6	1	0	0	0	0	0	0
3	6	1	0	0	0	0	0	0
3	1	0	0	0	1	0	0	0
1	1	1	0	0	1	0	0	0
2	1	1	1	0	1	0	0	0
3	1	1	1	0	0	0	0	0
3	1	1	1	0	1	0	0	0
3	1	0	1	0	1	0	0	0
4	6	1	0	0	0	0	0	0
2	6	1	0	0	0	0	0	0
4	6	1	0	0	0	0	0	0
3	6	1	0	0	0	0	0	0
5	6	1	1	1	0	0	0	0
4	6	1	0	0	0	0	0	0
4	1	0	1	0	1	0	0	0
4	1	1	1	0	0	0	0	0
4	1	1	0	0	1	0	0	0
3	1	1	1	0	1	0	0	0
3	1	0	1	0	1	0	0	0
3	6	1	0	0	0	0	0	0
5	6	1	0	0	0	0	0	0
3	6	1	0	0	0	0	0	0

4	6	1	0	0	0	0	0	0
2	6	1	0	0	0	0	0	0
4	6	1	0	0	0	0	0	0
5	6	1	1	0	0	0	0	0
3	6	1	0	0	0	0	0	0
3	6	1	0	0	0	0	0	0
2	4	1	0	0	0	0	0	0
4	6	1	0	0	0	0	0	0
4	6	1	1	0	0	0	0	0
5	6	1	0	0	0	0	0	0
4	6	1	0	0	0	0	0	0
4	6	1	0	0	0	0	0	0
3	6	1	0	0	0	0	0	0
2	6	1	0	0	0	0	0	0
2	6	1	0	0	0	0	0	0
2	6	1	0	0	0	0	0	0
3	6	1	0	0	0	0	0	0
3	6	1	0	0	0	0	0	0
3	1	1	0	0	1	0	0	0
4	6	1	0	0	0	0	0	0
4	6	1	1	0	0	0	0	0
2	6	1	0	0	0	0	0	0
2	6	1	1	0	0	0	0	0
3	6	1	0	0	0	0	0	0
4	5	1	0	0	0	0	0	0
3	6	1	0	0	0	0	0	0
3	6	1	0	0	0	0	0	0
3	6	1	0	0	0	0	0	0
3	6	1	0	0	0	0	0	0
3	1	1	1	0	1	0	0	0
3	1	0	0	0	1	0	0	0
3	1	0	1	0	1	0	0	0
4	1	0	1	0	1	0	0	0
2	6	1	0	0	0	0	0	0
3	6	1	1	0	0	0	0	0
3	6	1	0	0	0	0	0	0
2	6	1	0	0	0	0	0	0
2	6	1	0	0	0	0	0	0
4	6	1	0	0	0	0	0	0
3	6	1	0	0	0	0	0	0
3	6	0	1	0	0	0	0	0
4	6	0	1	0	0	0	0	0
1	1	1	1	0	1	0	0	0
1	6	1	0	0	0	0	0	0
3	6	1	1	0	0	0	0	0
2	6	0	1	0	0	0	0	0
3	6	1	0	0	0	0	0	0
1	3	0	1	0	0	0	0	1

2	2	1	0	0	0	1	0	0
2	2	1	0	0	0	0	1	0
1	6	1	0	0	0	0	0	0
3	6	1	1	0	0	0	0	0
3	6	1	0	0	0	0	0	0
3	1	1	0	0	1	0	0	0
2	1	0	1	0	1	0	0	0
2	6	1	0	0	0	0	0	0
3	6	1	0	0	0	0	0	0
4	5	1	0	0	0	0	0	0
2	6	1	0	0	0	0	0	0
3	6	1	0	0	0	0	0	0
3	6	1	1	0	0	0	0	0
2	6	1	0	0	0	0	0	0
3	6	1	0	0	0	0	0	0
4	2	1	0	0	0	1	0	0
2	2	1	0	0	0	1	0	0
4	6	1	0	0	0	0	0	0
3	5	1	0	0	0	0	0	0
3	6	1	0	0	0	0	0	0
2	2	1	0	0	0	1	0	0
3	1	0	1	0	1	0	0	0
3	1	1	1	0	1	0	0	0
3	1	1	1	0	1	0	0	0
2	1	0	1	0	1	0	0	0
2	1	0	0	0	1	0	0	0
3	1	0	1	0	1	0	0	0
4	6	1	0	0	0	0	0	0
1	6	1	0	0	0	0	0	0
4	6	1	1	0	0	0	0	0
3	6	1	0	0	0	0	0	0
3	6	1	0	0	0	0	0	0
4	6	1	0	0	0	0	0	0
3	6	1	0	0	0	0	0	0
3	6	1	0	0	0	0	0	0
3	6	1	0	0	0	0	0	0
3	6	1	0	0	0	0	0	0
3	2	1	0	0	0	1	0	0
2	2	1	0	0	0	1	0	0
2	2	1	0	0	0	0	0	0
4	6	1	0	0	0	0	0	0
1	6	1	1	0	0	0	0	0
3	6	1	0	0	0	0	0	0
4	6	1	0	0	0	0	0	0
2	6	0	1	0	0	0	0	0
1	6	1	0	0	0	0	0	0
4	6	1	1	0	0	0	0	0
3	6	1	1	0	0	0	0	0

3	6	1	0	0	0	0	0	0
4	6	1	0	0	0	0	0	0
3	2	1	0	0	0	0	0	0
4	2	1	0	0	0	0	0	0
4	6	0	0	1	0	0	0	0
3	6	1	0	0	0	0	0	0
4	4	1	0	0	0	0	0	0
1	4	1	0	0	0	0	0	0
3	2	1	0	0	0	1	0	0
3	2	1	0	0	0	0	1	0
3	2	1	0	0	0	1	0	0
3	6	1	0	0	0	0	0	0
3	6	1	0	0	0	0	0	0
4	6	1	0	0	0	0	0	0
4	1	1	0	0	0	0	0	0
1	6	1	0	0	0	0	0	0
4	6	1	0	0	0	0	0	0
1	6	1	0	0	0	0	0	0
3	6	1	0	0	0	0	0	0
4	6	1	0	0	0	0	0	0
4	5	1	0	0	0	0	0	0
2	1	0	1	0	1	0	0	0
4	6	1	0	0	0	0	0	0
2	4	1	0	0	0	0	0	0
3	4	1	0	0	0	0	0	0
3	6	1	0	0	0	0	0	0
4	6	0	1	0	0	0	0	0
2	1	1	1	0	1	0	0	0
2	6	1	0	0	0	0	0	0
3	6	1	0	0	0	0	0	0
2	6	1	0	0	0	0	0	0
2	1	1	0	0	1	0	0	0
1	1	1	1	0	0	0	0	0
4	1	1	1	0	1	0	0	0
4	1	0	0	0	1	0	0	0
4	1	0	1	0	1	0	0	0
3	1	0	1	0	0	0	0	0
4	1	1	1	0	0	0	0	0
4	1	1	1	0	1	0	0	0
4	1	1	1	0	1	0	0	0
1	1	0	1	0	0	0	0	0
4	5	0	1	0	0	0	0	0
2	6	1	0	0	0	0	0	0
4	6	1	0	0	0	0	0	0
4	1	0	1	0	1	0	0	0
4	1	1	1	0	1	0	0	0
5	1	1	1	0	1	0	0	0
2	6	1	0	0	0	0	0	0

3	1	1	1	0	1	0	0	0
3	1	1	1	0	1	0	0	0
3	1	0	1	0	1	0	0	0
4	1	1	1	0	1	0	0	0
3	1	1	1	0	1	0	0	0
2	1	0	1	0	1	0	0	0
1	6	1	0	0	0	0	0	0
3	6	1	0	0	0	0	0	0
2	1	0	1	0	1	0	0	0
2	1	0	1	0	1	0	0	0
3	1	0	1	0	1	0	0	0
1	1	0	0	0	1	0	0	0
3	1	1	1	0	1	0	0	0
3	1	0	1	0	1	0	0	0
3	6	1	0	0	0	0	0	0
4	6	1	0	0	0	0	0	0
3	6	1	1	0	0	0	0	0
3	6	1	0	0	0	0	0	0
2	6	1	0	0	0	0	0	0
4	6	1	0	0	0	0	0	0
3	6	1	0	0	0	0	0	0
4	6	1	0	0	0	0	0	0
4	6	1	1	0	0	0	0	0
3	6	0	1	0	0	0	0	0
2	6	1	1	0	0	0	0	0
4	6	1	1	0	0	0	0	0
3	6	1	0	0	0	0	0	0
4	6	1	0	0	0	0	0	0
2	5	1	0	0	0	0	0	0
1	6	1	0	0	0	0	0	0
4	6	1	0	0	0	0	0	0
2	6	1	0	0	0	0	0	0
3	6	1	0	0	0	0	0	0
3	1	0	1	0	1	0	0	0
4	1	1	0	0	0	0	0	0
2	1	0	1	0	1	0	0	0
2	1	1	0	0	0	0	0	0
2	1	0	1	0	0	0	0	0
5	2	1	1	0	1	0	0	0
3	3	0	1	0	0	0	0	1
2	6	1	1	0	0	0	0	0
2	5	0	1	0	0	0	0	0
4	1	1	1	0	1	0	0	0
4	1	0	1	0	1	0	0	0
5	1	0	1	0	1	0	0	0
4	1	1	1	0	1	0	0	0
4	1	0	1	0	1	0	0	0
3	6	1	0	0	0	0	0	0

3	6	1	0	0	0	0	0	0
1	1	0	1	0	0	0	0	0
4	4	1	0	0	0	0	0	0
4	4	1	0	0	0	0	0	0
2	1	0	1	0	1	0	0	0
2	1	0	0	0	1	0	0	0
4	1	1	1	0	1	0	0	0
3	1	0	1	0	0	0	0	0
4	1	1	1	0	1	0	0	0
3	6	1	0	0	0	0	0	0
3	1	0	1	0	1	0	0	0
1	1	1	1	0	1	0	0	0
3	1	1	0	0	0	0	0	0
1	2	1	0	0	0	0	1	0
3	5	1	0	0	0	1	0	1
5	6	1	1	0	1	0	0	0
3	6	1	0	0	0	0	0	0
3	1	0	1	0	1	0	0	0
3	1	0	1	0	0	0	0	0
3	1	1	0	0	1	0	0	0
3	6	1	0	0	0	0	0	0
3	4	0	0	0	1	0	0	0
2	6	1	0	0	0	0	0	0
2	1	1	1	0	0	0	0	0
5	1	1	1	0	0	0	0	0
4	6	1	1	0	0	0	0	0
2	5	0	1	0	0	0	0	0
3	1	0	1	0	0	0	0	0
3	1	1	1	0	1	0	0	0
2	1	0	1	0	0	0	0	0
4	1	1	1	0	0	0	0	0
4	6	1	0	0	0	0	0	0
2	1	0	1	0	1	0	0	0
2	2	0	0	0	0	1	0	0
2	1	0	1	0	0	0	0	0
3	1	0	1	0	0	0	0	0
2	1	1	1	0	1	0	0	0
3	1	1	1	0	1	0	0	0
4	6	0	1	0	1	0	0	0
4	6	1	0	0	0	0	0	0
3	6	1	0	0	0	0	0	0
3	6	1	0	0	0	0	0	0
2	6	1	1	0	0	0	0	0
2	1	1	1	0	0	0	0	0
3	1	1	1	0	1	0	0	0
2	1	0	1	0	1	0	0	0
3	1	1	1	0	0	0	0	0
3	2	1	0	0	0	1	0	0

5	4	1	0	0	0	0	0	0
3	6	1	0	0	0	0	0	0
1	4	1	0	0	0	0	0	0
4	6	1	0	0	0	0	0	0
4	6	1	0	0	0	0	0	0
5	6	1	0	0	0	0	0	0
3	6	1	0	0	0	0	0	0
4	6	1	1	0	0	0	0	0
2	6	0	1	0	0	0	0	0
3	1	1	1	0	1	0	0	0
3	1	0	1	0	1	0	0	0
3	1	1	1	0	1	0	0	0
4	6	1	0	0	0	0	0	0
1	6	1	0	0	0	0	0	0
4	6	1	0	0	0	0	0	0
2	6	1	0	0	0	0	0	0
4	6	1	0	0	0	0	0	0
3	6	1	0	0	0	0	0	0
2	6	1	0	0	0	0	0	0
3	6	1	0	0	0	0	0	0
4	6	1	0	0	0	0	0	0
3	6	1	0	0	0	0	0	0
4	6	1	1	0	0	0	0	0
4	6	1	0	0	0	0	0	0
4	4	1	0	0	0	0	0	0
3	6	1	0	0	0	0	0	0
4	6	1	1	0	0	0	0	0
4	6	1	0	0	0	0	0	0
4	6	1	0	0	0	0	0	0
4	6	1	0	0	0	0	0	0
2	6	1	0	0	0	0	0	0
3	6	1	0	0	0	0	0	0
3	6	1	0	0	0	0	0	0
3	5	1	0	0	0	0	0	0
4	4	1	0	0	0	0	0	0
2	6	1	0	0	0	0	0	0
4	6	1	0	0	0	0	0	0
3	6	1	0	0	0	0	0	0
3	1	1	1	0	0	0	0	0
1	1	1	1	0	0	0	0	0
4	6	1	0	0	0	0	0	0
2	1	1	0	0	0	0	0	0
2	1	0	0	0	1	0	0	0
4	6	1	1	0	0	0	0	0
1	1	0	1	0	0	0	0	0
3	1	1	1	0	1	0	0	0
3	1	0	1	0	1	0	0	0
1	1	0	1	0	1	0	0	0

2	1	1	1	0	1	0	0	0
4	1	1	0	0	1	0	0	0
3	1	1	1	0	1	0	0	0
3	1	1	0	0	1	0	0	0
4	6	1	0	0	0	0	0	0
2	4	0	0	0	1	0	0	0
1	1	1	1	0	1	0	0	0
3	6	1	1	0	0	0	0	0
3	6	1	0	0	0	0	0	0
1	1	1	1	0	1	0	0	0
1	6	1	0	0	0	0	0	0
2	1	0	1	0	1	0	0	0
2	1	1	1	0	1	0	0	0
3	2	1	0	0	0	1	1	0
2	2	1	0	0	0	1	1	0
5	1	0	1	0	1	0	0	0
2	1	0	1	0	1	0	0	0
5	1	1	1	0	0	0	0	0
1	1	0	1	0	1	0	0	0
3	1	1	1	0	1	0	0	0
3	6	1	0	0	0	0	0	0
5	1	0	0	0	1	0	0	0
3	6	1	0	0	0	0	0	0
2	6	1	0	0	0	0	0	0
2	4	1	0	0	0	0	0	0
4	6	1	0	0	0	0	0	0
4	6	1	0	0	0	0	0	0
2	6	1	0	0	0	0	0	0
3	6	1	0	0	0	0	0	0
2	6	1	0	0	0	0	0	0
3	6	1	0	0	0	0	0	0
2	6	1	0	0	0	0	0	0
3	4	0	0	0	1	0	0	0
2	6	1	0	0	0	0	0	0
4	6	1	0	0	0	0	0	0
2	1	1	0	0	0	0	0	0
3	1	0	1	0	1	0	0	0
3	6	1	0	0	0	0	0	0
3	6	1	0	0	0	0	0	0
2	6	1	0	0	0	0	0	0
3	6	1	0	0	0	0	0	0
4	6	1	0	0	0	0	0	0
2	6	1	0	0	0	0	0	0
3	6	1	0	0	0	0	0	0
4	1	1	1	0	1	0	0	0
2	1	1	0	0	0	0	0	0
2	6	1	0	0	0	0	0	0
4	6	1	0	0	0	0	0	0

2	1	0	1	0	0	0	0	0
3	1	1	0	0	1	0	0	0
3	1	1	1	0	1	0	0	0
1	6	1	0	0	0	0	0	0
3	6	1	0	0	0	0	0	0
2	5	1	0	0	0	0	0	0
3	1	0	1	0	1	0	0	0
4	6	1	0	0	0	0	0	0
2	3	0	0	0	0	0	0	1
2	6	1	0	0	0	0	0	0
2	1	1	1	0	1	0	0	0
5	1	1	1	0	1	0	0	0
3	1	1	1	0	1	0	0	0
3	6	1	0	0	0	0	0	0
3	1	1	1	0	1	0	0	0
3	1	0	1	0	1	0	0	0
3	6	1	1	0	0	0	0	0
3	6	1	1	0	0	0	0	0
3	6	1	0	0	0	0	0	0
3	1	1	1	0	1	0	0	0
3	6	1	1	0	0	0	0	0
3	6	1	0	0	0	0	0	0
3	6	1	0	0	0	0	0	0
3	1	0	0	0	1	0	0	0
1	6	0	1	0	0	0	0	0
4	1	1	1	0	1	0	0	0
4	6	0	1	0	0	0	0	0
3	6	1	1	0	0	0	0	0
1	1	0	1	0	1	0	0	0
4	6	1	0	0	0	0	0	0
4	6	1	1	0	0	0	0	0
1	6	0	1	0	0	0	0	0
4	6	1	1	0	0	0	0	0
3	6	1	0	0	0	0	0	0
4	6	1	0	0	0	0	0	0
3	6	1	0	0	0	0	0	0
4	3	0	0	0	0	0	0	1
3	6	0	1	0	0	0	0	0
3	6	0	1	0	0	0	0	0
1	2	0	1	0	0	0	0	0
1	6	1	1	0	0	0	0	0
2	1	0	1	0	0	0	0	0
3	5	0	1	0	0	0	0	0
2	5	0	1	0	0	0	0	0
3	2	0	1	0	0	0	0	0
1	1	0	0	0	1	0	0	0
4	6	1	1	0	0	0	0	0
1	6	1	0	0	0	0	0	0

3	6	0	1	0	0	0	0	0
2	1	0	1	0	0	0	0	0
4	6	0	0	0	0	0	0	0
3	6	1	1	0	0	0	0	0
2	6	1	0	0	0	0	0	0
3	6	1	0	0	0	0	0	0
2	6	0	1	0	0	0	0	0
4	6	1	1	0	0	0	0	0
3	6	1	0	0	0	0	0	0
2	6	0	1	0	0	0	0	0
1	6	0	1	0	0	0	0	0
2	6	1	1	0	0	0	0	0
2	4	1	0	0	0	0	0	0
2	6	0	1	0	0	0	0	0
3	6	0	1	0	0	0	0	0
2	1	0	0	0	1	0	0	0
1	6	0	1	0	0	0	0	0
2	6	1	1	0	0	0	0	0
3	6	0	1	0	0	0	0	0
3	6	1	0	0	0	0	0	0
1	6	1	0	0	0	0	0	0
2	5	1	0	0	1	0	0	0
2	4	0	0	0	1	0	0	0
2	6	1	0	0	0	0	0	0
2	6	1	0	0	0	0	0	0
2	6	1	0	0	0	0	0	0
1	6	0	1	0	0	0	0	0
1	6	0	1	0	0	0	0	0
1	6	0	1	0	0	0	0	0
1	6	0	1	0	0	0	0	0
1	6	0	1	0	0	0	0	0
3	6	1	0	0	0	0	0	0
2	1	0	1	0	1	0	0	0
3	6	1	0	0	0	0	0	0
4	6	1	1	0	0	0	0	0
2	6	1	0	0	0	0	0	0
4	6	1	0	0	0	0	0	0
2	1	1	1	0	1	0	0	0
4	6	1	0	0	0	0	0	0
4	1	1	1	0	1	0	0	0
4	2	0	1	0	1	1	0	0
3	6	1	0	0	0	0	0	0
2	6	1	0	0	0	0	0	0
4	1	1	1	0	0	0	0	0
4	6	1	0	0	0	0	0	0
3	6	1	0	0	0	0	0	0
2	6	1	0	0	0	0	0	0
4	6	1	1	0	0	0	0	0

5	5	1	1	0	0	0	0	0
4	6	1	0	0	0	0	0	0
3	1	0	1	0	1	0	0	0
5	3	0	0	0	0	0	0	1
4	6	1	0	0	0	0	0	0
4	1	1	1	0	0	0	0	0
3	1	1	0	0	1	0	0	0
2	6	1	0	0	0	0	0	0
2	2	1	0	0	0	0	0	0
4	1	1	1	0	1	0	0	0
3	4	0	0	0	1	0	0	0
4	6	1	0	0	0	0	0	0
3	6	1	1	0	0	0	0	0
3	6	1	0	0	0	0	0	0
4	6	1	0	0	0	0	0	0
2	1	0	0	0	1	0	0	0
4	1	0	1	0	1	0	0	0
3	6	1	0	0	0	0	0	0
3	6	1	0	0	0	0	0	0
2	1	0	1	0	1	0	0	0
3	1	1	1	0	1	0	0	0
3	1	1	1	0	1	0	0	0
2	6	1	0	0	0	0	0	0
1	1	1	1	0	0	0	0	0
3	2	1	0	0	0	0	1	0
2	1	1	0	0	1	0	0	0
2	1	1	0	0	1	0	0	0
3	6	1	0	0	0	0	0	0
2	6	1	1	0	0	0	0	0
1	1	1	1	0	1	0	0	0
5	1	1	1	0	1	0	0	0
3	1	1	1	0	1	0	0	0
3	1	0	1	0	1	0	0	0
3	1	1	1	0	1	0	0	0
4	1	0	1	0	1	0	0	0
4	1	0	0	0	1	0	0	0
4	1	1	0	0	1	0	0	0
4	6	0	1	0	0	0	0	0
4	1	0	1	0	1	0	0	0
3	1	1	1	0	1	0	0	0
2	1	1	1	0	1	0	0	0
4	1	0	1	0	1	0	0	0
1	6	1	1	0	0	0	0	0
2	1	1	1	0	1	0	0	0
3	6	1	0	0	0	0	0	0
5	1	0	1	0	1	0	0	0
3	6	1	1	0	0	0	0	0
3	1	1	1	0	1	0	0	0

2	4	1	0	0	0	0	0	0
3	6	1	0	0	0	0	0	0
2	6	1	1	0	0	0	0	0
3	4	1	0	0	0	0	0	0
3	4	1	0	0	0	0	0	0
4	1	0	1	0	1	0	0	0
4	1	1	1	0	1	0	0	0
1	1	0	1	0	1	0	0	0
2	4	1	0	0	0	0	0	0
2	6	1	0	0	0	0	0	0
3	4	0	0	0	1	0	0	0
3	6	1	0	0	0	0	0	0
2	4	1	0	0	0	0	0	0
2	6	1	0	0	0	0	0	0
4	6	1	1	0	0	0	0	0
3	6	1	0	0	0	0	0	0
2	4	1	0	0	0	0	0	0
3	4	1	1	0	0	0	0	0
4	1	1	1	0	1	0	0	0
4	1	1	1	0	1	0	0	0
3	6	1	0	0	0	0	0	0
3	6	1	0	0	0	0	0	0
2	5	0	1	0	1	0	0	0
2	6	1	0	0	0	0	0	0
3	6	1	0	0	0	0	0	0
3	4	1	0	0	1	0	0	0
3	1	0	1	0	1	0	0	0
4	1	1	1	0	1	0	0	0
2	4	1	0	0	0	0	0	0
4	6	1	0	0	0	0	0	0
2	6	1	0	0	0	0	0	0
4	6	1	1	0	0	0	0	0
3	6	1	0	0	0	0	0	0
2	6	1	0	0	0	0	0	0
2	1	0	1	0	1	0	0	0
3	6	0	1	0	0	0	0	0
3	6	1	0	0	0	0	0	0
3	6	0	1	0	0	0	0	0
2	6	1	0	0	0	0	0	0
3	2	1	0	0	0	1	0	0
3	2	1	0	0	0	1	1	0
4	2	1	1	0	1	1	0	0
4	1	0	1	0	1	0	0	0
3	2	0	0	0	0	0	1	0
3	1	0	1	0	1	0	0	0
4	1	1	1	0	0	0	0	0
4	6	0	1	0	0	0	0	0
2	1	1	0	0	0	0	0	0

4	1	1	1	0	1	0	0	0
5	1	0	1	0	1	0	0	0
4	1	0	1	0	0	0	0	0
4	1	1	1	0	0	0	0	0
5	1	0	1	0	1	0	0	0
2	2	1	0	0	0	1	0	0
3	6	1	0	0	0	0	0	0
3	4	1	0	0	1	0	0	0
2	6	1	0	0	0	0	0	0
3	1	0	0	0	1	0	0	0
3	4	1	0	0	1	0	0	0
1	1	0	0	0	1	0	0	0
4	4	1	0	0	1	0	0	0
2	1	1	1	0	0	0	0	0
2	1	1	1	0	0	0	0	0
1	4	1	0	0	0	0	0	0
2	4	0	0	0	1	0	0	0
2	1	1	1	0	1	0	0	0
2	4	0	0	0	1	0	0	0
4	1	1	1	0	0	0	0	0
2	6	1	0	0	0	0	0	0
2	4	0	0	0	1	0	0	0
3	6	1	1	0	0	0	0	0
4	4	1	0	0	0	0	0	0
3	6	0	1	0	0	0	0	0
2	4	1	0	0	0	0	0	0
3	5	1	1	0	0	0	0	0
3	6	1	0	0	0	0	0	0
2	4	1	0	0	0	0	0	0
2	4	1	0	0	0	0	0	0
2	1	1	0	0	0	0	0	0
2	4	1	0	0	0	0	0	0
3	1	1	1	0	1	0	0	0
2	4	1	0	0	0	0	0	0
2	6	1	0	0	0	0	0	0
3	4	1	1	0	0	0	0	0
3	6	1	1	0	0	0	0	0
1	6	0	1	0	0	0	0	0
2	6	1	0	0	0	0	0	0
2	1	1	1	0	0	0	0	0
3	6	1	0	0	0	0	0	0
1	4	1	0	0	0	0	0	0
3	5	1	0	0	0	0	0	0
2	1	1	1	0	1	0	0	0
3	6	1	0	0	0	0	0	0
2	4	1	0	0	0	0	0	0
2	4	1	0	0	0	0	0	0
2	6	1	0	0	0	0	0	0

2	4	1	0	0	0	0	0	0
3	6	1	0	0	0	0	0	0
2	6	1	1	0	0	0	0	0
3	1	0	1	0	1	0	0	0
4	4	1	0	0	0	0	0	0
2	4	0	0	0	1	0	0	0
3	6	1	0	0	0	0	0	0
3	3	0	1	0	0	0	0	1
2	4	1	0	0	0	0	0	0
2	4	1	0	0	0	0	0	0
3	4	1	0	0	0	0	0	0
3	4	0	0	0	1	0	0	0
3	1	1	1	0	0	0	0	0
3	5	1	0	0	0	0	0	0
2	5	1	1	0	1	0	0	0
4	6	1	1	0	0	0	0	0
3	1	0	1	0	1	0	0	0
3	6	1	0	0	0	0	0	0
1	1	0	1	0	1	0	0	0
4	4	1	1	0	1	0	0	0
2	1	0	1	0	1	0	0	0
3	6	1	1	0	0	0	0	0
3	6	1	0	0	0	0	0	0
3	5	1	1	0	0	0	0	0
3	1	0	1	0	1	0	0	0
4	5	1	0	0	0	0	0	0
3	6	1	0	0	0	0	0	0
3	6	1	1	0	0	0	0	0
4	1	0	0	0	1	0	0	0
2	6	1	0	0	0	0	0	0
3	1	1	0	0	0	0	0	0
2	1	1	0	0	0	0	0	0
2	4	1	1	0	0	0	0	0
4	1	0	1	0	1	0	0	0
3	6	0	1	0	0	0	0	0
2	4	0	1	0	0	0	0	0
4	6	1	1	0	0	0	0	0
1	6	1	0	0	0	0	0	0
3	1	0	1	0	1	0	0	0
3	6	0	1	0	0	0	0	0
4	2	1	0	0	1	1	0	0
2	3	0	0	0	0	0	0	1
2	6	0	1	0	0	0	0	0
3	4	1	0	0	0	0	0	0
2	1	0	0	0	1	0	0	0
4	1	0	1	0	0	0	0	0
3	6	1	0	0	0	0	0	0
1	6	1	0	0	0	0	0	0

4	6	1	0	0	0	0	0	0
4	6	1	1	0	0	0	0	0
1	5	1	0	0	0	0	0	0
4	4	1	0	0	1	0	0	0
4	2	1	0	0	0	1	1	0
4	5	1	0	0	0	0	0	0
3	1	1	1	0	0	0	0	0
4	5	1	0	0	0	0	0	0
4	4	0	0	0	1	0	0	0
4	1	1	0	0	0	0	0	0
4	3	0	0	0	0	0	0	1
1	6	1	0	0	0	0	0	0
4	1	0	1	0	0	0	0	0
4	1	0	1	0	1	0	0	0
2	1	1	0	0	1	0	0	0
3	1	0	1	0	0	0	0	0
2	2	1	1	0	0	1	0	0
5	1	1	0	0	1	0	0	0
4	1	1	1	0	1	0	0	0
2	2	1	0	0	0	0	0	0
2	6	1	0	0	0	0	0	0
4	1	0	1	0	1	0	0	0
4	1	0	1	0	1	0	0	0
2	2	1	0	0	0	1	0	0
3	1	0	1	0	1	0	0	0
3	4	0	0	0	1	0	0	0
5	1	1	1	0	1	0	0	0
4	1	0	1	0	1	0	0	0
3	4	0	0	0	1	0	0	0
3	2	1	0	0	0	0	0	0
5	1	0	1	0	1	0	0	0
4	6	1	0	0	0	0	0	0
4	1	1	1	0	1	0	0	0
4	5	1	0	0	0	0	0	0
2	6	1	0	0	0	0	0	0
2	6	1	0	0	0	0	0	0
4	5	0	1	0	0	0	0	0
3	1	1	1	0	1	0	0	0
3	6	1	0	0	0	0	0	0
3	6	1	0	0	0	0	0	0
3	6	1	0	0	0	0	0	0
2	4	1	0	0	1	0	0	0
3	4	1	0	0	1	0	0	0
2	4	1	0	0	0	0	0	0
4	6	0	1	0	0	0	0	0
2	4	1	0	0	0	0	0	0
2	5	0	1	0	1	0	0	0
2	4	1	0	0	0	0	0	0

2	6	1	0	0	0	0	0	0
4	6	0	1	0	0	0	0	0
2	1	1	0	0	0	0	0	0
1	4	0	0	0	1	0	0	0
2	1	1	0	0	0	0	0	0
1	1	1	1	0	1	0	0	0
3	6	1	0	0	0	0	0	0
3	6	1	0	0	0	0	0	0
3	5	1	1	0	0	0	0	0
3	6	1	0	0	0	0	0	0
3	1	0	1	0	1	0	0	0
2	4	0	0	0	1	0	0	0
3	1	1	1	0	0	0	0	0
4	4	1	0	0	1	0	0	0
3	4	0	0	0	1	0	0	0
5	6	0	1	0	0	0	0	0
3	6	1	1	0	0	0	0	0
2	1	1	0	0	0	0	0	0
2	4	0	0	0	1	0	0	0
4	4	0	0	1	0	0	0	0
2	1	1	0	0	0	0	0	0
2	6	1	0	0	0	0	0	0
2	4	1	0	0	0	0	0	0
2	1	1	0	0	0	0	0	0
4	1	0	1	0	1	0	0	0
5	6	1	1	0	0	0	0	0
3	4	1	0	0	1	0	0	0
2	4	1	0	0	0	0	0	0
3	1	1	0	0	0	0	0	0
3	4	1	0	0	0	0	0	0
3	6	1	0	0	0	0	0	0
2	6	1	0	0	0	0	0	0
2	6	1	0	0	0	0	0	0
2	1	1	0	0	0	0	0	0
1	4	1	0	0	0	0	0	0
2	1	1	0	0	1	0	0	0
2	4	0	0	0	1	0	0	0
2	4	1	0	0	0	0	0	0
2	4	0	0	0	1	0	0	0
1	4	0	0	0	1	0	0	0
2	1	1	1	0	0	0	0	0
1	4	1	0	0	1	0	0	0
4	6	1	1	0	0	0	0	0
3	6	1	0	0	0	0	0	0
3	1	0	1	0	1	0	0	0
1	1	0	0	0	1	0	0	0
3	1	1	0	0	1	0	0	0
4	6	1	1	0	0	0	0	0

2	1	1	1	0	1	0	0	0
2	1	0	1	0	1	0	0	0
3	1	1	0	0	0	0	0	0
2	1	0	0	0	1	0	0	0
1	1	1	1	0	1	0	0	0
2	4	1	0	0	0	0	0	0
2	1	1	1	0	0	0	0	0
2	4	1	0	0	0	0	0	0
3	4	0	0	0	1	0	0	0
3	6	1	0	0	0	0	0	0
3	6	1	0	0	0	0	0	0
5	6	1	1	1	0	0	0	0
3	6	1	1	0	0	0	0	0
1	6	1	1	0	0	0	0	0
3	1	1	1	0	1	0	0	0
4	1	1	1	0	1	0	0	0
2	1	1	0	0	0	0	0	0
2	4	1	0	0	0	0	0	0
2	6	1	0	0	0	0	0	0
2	1	1	1	0	1	0	0	0
2	6	1	1	0	0	0	0	0
2	4	1	0	0	0	0	0	0
1	4	1	0	0	0	0	0	0
2	1	1	1	0	0	0	0	0
4	6	1	1	0	0	0	0	0
1	4	1	0	0	1	0	0	0
3	4	1	0	0	1	0	0	0
3	6	1	1	0	0	0	0	0
3	4	0	0	0	1	0	0	0
3	4	1	0	0	0	0	0	0
2	4	1	0	0	0	0	0	0
4	6	0	1	0	0	0	0	0
1	6	0	1	0	0	0	0	0
2	6	1	0	0	0	0	0	0
4	4	1	0	0	1	0	0	0
3	5	1	0	0	0	0	0	0
4	6	1	0	0	0	0	0	0
3	2	0	0	0	0	0	0	1
3	6	1	1	0	0	0	0	0
2	6	1	0	0	0	0	0	0
4	1	0	1	0	1	0	0	0
3	1	1	1	0	0	0	0	0
3	1	0	1	0	1	0	0	0
3	5	1	0	0	0	0	0	0
2	1	1	1	0	1	0	0	0
2	1	0	0	0	1	0	0	0
2	4	1	0	0	0	0	0	0
3	6	1	0	0	0	0	0	0

3	1	1	0	0	1	0	0	0
4	6	1	1	0	0	0	0	0
3	6	1	0	0	0	0	0	0
2	6	1	0	0	0	0	0	0
5	6	0	1	0	0	0	0	0
5	6	0	1	0	0	0	0	0
5	6	1	1	1	0	0	0	0
2	6	0	1	0	0	0	0	0
5	6	1	1	0	0	0	0	0
4	1	0	1	0	1	0	0	0
2	4	0	0	0	1	0	0	0
5	3	0	1	0	1	1	0	0
4	1	1	1	0	1	0	0	0
3	1	1	1	0	0	0	0	0
4	5	1	1	0	0	0	0	0
3	2	1	0	0	0	0	0	0
3	4	1	0	0	0	0	0	0
2	4	1	0	0	1	0	0	0
3	4	1	0	0	0	0	0	0
2	5	0	1	0	1	0	0	0
3	6	1	0	0	0	0	0	0
3	5	0	0	0	0	0	0	1
2	4	0	0	0	1	0	0	0
2	4	0	0	0	1	0	0	0
5	1	1	1	0	0	0	0	0
2	4	0	0	0	1	0	0	0
4	4	1	0	0	0	0	0	0
5	1	0	1	0	1	0	0	0
1	1	1	0	0	0	0	0	0
2	4	0	0	0	1	0	0	0
2	4	1	0	0	1	0	0	0
3	4	0	0	0	1	0	0	0
3	4	0	0	0	1	0	0	0
5	1	0	1	0	1	0	0	0
3	1	1	0	0	0	0	0	0
5	1	1	1	0	1	0	0	0
3	5	1	1	0	1	0	0	0
3	1	1	1	0	1	0	0	0
2	4	1	0	0	1	0	0	0
3	5	1	1	0	0	0	0	0
3	4	1	0	0	1	0	0	0
4	1	1	0	0	1	0	0	0
4	1	1	1	0	0	0	0	0
1	6	1	0	0	0	0	0	0
4	1	1	1	0	0	0	0	0
2	6	1	0	0	0	0	0	0
4	6	1	1	0	0	0	0	0
2	4	1	0	0	0	0	0	0

3	4	1	0	0	1	0	0	0
4	6	1	1	0	0	0	0	0
3	6	1	0	0	0	0	0	0
3	1	1	1	0	0	0	0	0
2	4	1	0	0	0	0	0	0
2	1	1	1	0	1	0	0	0
3	1	1	1	0	1	0	0	0
2	4	1	0	0	0	0	0	0
1	1	0	1	0	0	0	0	0
2	6	0	1	0	0	0	0	0
3	1	1	1	0	0	0	0	0
3	6	1	0	0	0	0	0	0
3	4	1	0	0	0	0	0	0
2	6	0	1	0	0	0	0	0
1	1	0	1	0	1	0	0	0
2	1	1	1	0	1	0	0	0
2	1	1	1	0	1	0	0	0
4	1	1	1	1	1	1	0	0
2	1	0	1	0	1	0	0	0
2	4	1	0	0	0	0	0	0
2	1	1	0	0	0	0	0	0
2	1	1	0	0	1	0	0	0
4	4	1	0	0	0	0	0	0
2	4	1	0	0	0	0	0	0
2	4	1	0	0	0	0	0	1
5	1	0	1	0	1	0	0	0
3	1	0	1	0	1	0	0	0
2	1	1	0	0	0	0	0	0
2	1	1	1	0	1	0	0	0
3	1	1	1	0	0	0	0	0
4	5	1	0	0	0	0	0	0
5	1	0	1	0	1	0	0	0
2	6	0	1	0	0	0	0	0
2	6	1	0	0	0	0	0	0
3	6	1	0	0	0	0	0	0
2	6	0	1	0	0	0	0	0
2	6	1	0	0	0	0	0	0
3	6	1	1	0	0	0	0	0
3	6	1	0	0	0	0	0	0
5	6	1	1	0	0	0	0	0
2	6	1	0	0	0	0	0	0
5	6	1	1	0	0	0	0	0
5	6	1	1	1	0	0	0	0
5	6	1	1	0	0	0	0	0
5	6	1	1	0	0	0	0	0
5	6	1	1	1	0	0	0	0
3	6	0	1	0	0	0	0	0
4	6	0	1	0	0	0	0	0

5	6	1	1	0	0	0	0	0
5	6	1	1	0	0	0	0	0
5	6	1	1	0	0	0	0	0
5	6	0	1	0	0	0	0	0
5	6	1	1	0	0	0	0	0
5	6	1	1	0	0	0	0	0
5	6	1	1	0	0	0	0	0
4	6	1	1	0	0	0	0	0
5	6	1	1	0	0	0	0	0
5	6	1	1	0	0	1	0	0
4	6	1	1	0	0	0	0	0
4	6	0	1	0	0	0	0	0
3	6	1	1	0	0	0	0	0
5	6	1	1	0	0	0	0	0
5	6	1	0	0	0	0	0	0
5	6	1	1	1	0	0	0	0
3	6	1	1	0	0	0	0	0
5	6	0	1	0	0	0	0	0
2	1	1	1	0	0	0	0	0
2	1	1	1	0	1	0	0	0
2	1	0	1	0	1	0	0	0
3	6	1	0	0	0	0	0	0
4	1	1	1	0	1	0	0	0
4	1	0	1	0	1	0	0	0
4	1	0	1	0	1	0	0	0
4	1	1	1	0	1	0	0	0
4	6	1	1	0	0	0	0	0
4	3	1	1	0	1	0	0	0
4	1	0	1	0	1	0	0	0
4	6	1	1	0	0	0	0	0
4	6	1	1	0	0	0	0	0
2	6	1	0	0	0	0	0	0
3	1	0	1	0	1	0	0	0
1	6	1	0	0	0	0	0	0
3	6	1	1	0	0	0	0	0
2	4	1	0	0	0	0	0	0
3	4	1	0	0	1	0	0	0
1	1	0	1	0	1	0	0	0
4	1	0	1	0	1	0	0	0
3	6	1	0	0	0	0	0	0
2	1	1	0	0	0	0	0	0
2	1	1	1	0	1	0	0	0
2	4	1	0	0	0	0	0	0
3	1	1	1	0	1	0	0	0
3	1	1	1	0	1	0	0	0
1	1	1	1	0	1	0	0	0
3	1	0	1	0	1	0	0	0
5	1	1	1	0	1	0	0	0

4	1	0	1	0	1	0	0	0
1	1	1	1	0	1	0	0	0
4	6	1	0	0	0	0	0	0
2	6	1	0	0	0	0	0	0
2	1	0	1	0	1	0	0	0
2	4	1	0	0	1	0	0	0
1	1	0	1	0	1	0	0	0
2	1	0	0	0	1	0	0	0
4	1	0	1	0	1	0	0	0
2	4	1	0	0	1	0	0	0
3	5	1	0	0	0	0	0	0
1	1	0	1	0	1	0	0	0
3	6	1	0	0	0	0	0	0
2	1	0	0	0	1	0	0	0
4	1	1	1	0	1	0	0	0
4	6	1	1	0	0	0	0	0
3	6	1	0	0	0	0	0	0
2	4	1	0	0	0	0	0	0
1	5	1	0	0	0	0	0	0
4	1	0	1	0	1	0	0	0
4	1	0	1	0	1	0	0	0
4	1	1	1	0	1	0	0	0
3	1	0	1	0	1	0	0	0
2	6	1	0	0	0	0	0	0
3	6	1	0	0	0	0	0	0
4	3	1	1	0	1	0	0	0
4	1	0	1	0	1	0	0	0
2	4	1	0	0	0	0	0	0
2	6	1	0	0	0	0	0	0
3	6	1	0	0	0	0	0	0
1	6	1	0	0	0	0	0	0
5	6	1	1	0	0	0	0	0
4	6	1	1	1	0	0	0	0
5	6	1	1	0	0	0	0	0
5	6	1	1	0	0	0	0	0
4	6	1	1	0	0	0	0	0
4	6	0	1	0	0	0	0	0
4	6	1	1	0	0	0	0	0
1	6	0	1	0	0	0	0	0
4	6	0	1	1	0	0	0	0
5	6	1	1	0	0	0	0	0
5	6	1	1	1	0	0	0	0
3	4	1	0	0	0	0	0	0
3	4	1	0	0	1	0	0	0
4	1	1	0	0	0	0	0	0
2	4	1	0	0	1	0	0	0
3	1	0	1	0	1	0	0	0
2	6	1	0	0	0	0	0	0

3	4	1	0	0	0	0	0	0
3	6	1	0	0	0	0	0	0
3	4	1	0	0	0	0	0	0
2	5	0	1	0	1	0	0	0
3	4	1	0	0	0	0	0	0
2	4	1	0	0	0	0	0	0
3	4	1	0	0	0	0	0	0
2	4	0	0	0	1	0	0	0
4	1	1	1	0	0	0	0	0
1	4	0	0	0	1	0	0	0
3	4	0	0	0	1	0	0	0
3	4	1	0	0	0	0	0	0
3	1	1	1	0	1	0	0	0
2	6	1	0	0	0	0	0	0
2	4	1	1	0	0	0	0	0
3	2	1	0	0	0	0	0	0
2	6	1	0	0	0	0	0	0
2	4	1	0	0	0	0	0	0
2	1	0	0	0	1	0	0	0
3	1	0	1	0	1	0	0	0
1	4	1	0	0	0	0	0	0
2	1	1	0	0	0	0	0	0
3	6	1	0	0	0	0	0	0
3	6	1	0	0	0	0	0	0
2	1	0	0	0	1	0	0	0
1	6	0	1	0	0	0	0	0
2	6	0	1	0	0	0	0	0
4	1	0	0	0	1	0	0	0
3	6	0	1	0	0	0	0	0
3	1	0	0	0	1	0	0	0
3	6	0	1	0	0	0	0	0
1	6	1	1	0	0	0	0	0
4	5	1	0	0	0	0	0	0
3	6	1	0	0	0	0	0	0
2	6	1	1	0	0	0	0	0
4	6	1	0	0	0	0	0	0
4	6	1	1	0	0	0	0	0
5	6	1	1	0	0	0	0	0
5	6	1	1	0	0	0	0	0
3	6	1	1	0	0	0	0	0
2	1	0	1	0	0	0	0	0
4	6	1	1	0	0	0	0	0
5	1	1	1	0	1	0	0	0
3	1	0	1	0	0	0	0	0
3	6	1	0	0	0	0	0	0
4	1	1	1	0	0	0	0	0
3	6	1	0	0	0	0	0	0
1	1	0	1	0	1	0	0	0

3	1	1	0	0	0	0	0	0
3	6	1	0	0	0	0	0	0
2	1	1	1	0	1	0	0	0
2	1	1	1	0	1	0	0	0
4	1	1	1	0	1	0	0	0
3	6	0	1	0	0	0	0	0
4	3	0	0	0	0	0	0	1
4	6	1	1	0	0	0	0	0
3	1	1	1	0	0	0	0	0
1	6	1	0	0	0	0	0	0
4	1	0	1	0	1	0	0	0
4	3	0	0	0	0	0	0	1
3	1	1	1	0	1	0	0	0
2	2	1	1	0	0	0	1	0
2	1	1	1	0	1	0	0	0
4	1	0	1	0	1	0	0	0
4	2	1	0	0	0	0	0	0
2	4	0	0	0	1	0	0	0
2	4	1	1	0	0	0	0	0
3	4	0	1	0	1	0	0	0
3	6	1	0	0	0	0	0	0
3	6	1	0	0	0	0	0	0
5	1	1	1	0	1	0	0	0
1	6	1	0	0	0	0	0	0
3	1	0	1	0	1	0	0	0
5	1	0	1	0	1	0	0	0
4	1	0	1	0	1	0	0	0
3	6	1	0	0	0	0	0	0
3	6	1	0	0	0	0	0	0
4	6	1	1	0	0	0	0	0
4	6	1	0	0	0	0	0	0
4	6	1	0	0	0	0	0	0
2	1	0	1	0	1	0	0	0
2	6	1	0	0	0	0	0	0
3	1	1	1	0	1	0	0	0
3	1	0	1	0	1	0	0	0
1	6	1	0	0	0	0	0	0
2	1	0	1	0	1	0	0	0
3	1	1	0	0	1	0	0	0
3	1	0	1	0	1	0	0	0
2	1	1	1	0	1	0	0	0
3	1	1	1	0	1	0	0	0
3	6	1	1	0	0	0	0	0
3	6	1	1	0	0	0	0	0
3	6	1	1	0	0	0	0	0
3	1	0	1	0	1	0	0	0
5	6	1	1	0	0	0	0	0
4	6	1	1	0	0	0	0	0

4	1	0	1	0	1	0	0	0
4	6	1	0	0	0	0	0	0
3	6	1	0	0	0	0	0	0
4	6	1	1	0	0	0	0	0
2	6	1	0	0	0	0	0	0
3	6	1	0	0	0	0	0	0
3	6	1	0	0	0	0	0	0
2	6	1	0	0	0	0	0	0
2	6	1	0	0	0	0	0	0
2	6	1	0	0	0	0	0	0
2	6	1	1	0	0	0	0	0
3	6	0	1	0	0	0	0	0
4	6	1	0	0	0	0	0	0
2	6	1	0	0	0	0	0	0
3	6	1	0	0	0	0	0	0
2	6	1	0	0	0	0	0	0
3	4	1	0	0	0	0	0	0
2	6	1	1	0	0	0	0	0
3	6	1	1	0	0	0	0	0
2	6	1	0	0	0	0	0	0
3	6	1	0	0	0	0	0	0
3	6	1	0	0	0	0	0	0
2	6	1	1	0	0	0	0	0
2	6	1	0	0	0	0	0	0
2	6	1	0	0	0	0	0	0
3	6	1	0	0	0	0	0	0
2	6	1	0	0	0	0	0	0
4	6	1	1	0	0	0	0	0
1	6	1	0	0	0	0	0	0
3	6	1	0	0	0	0	0	0
2	6	1	0	0	0	0	0	0
3	6	1	0	0	0	0	0	0
3	6	1	0	0	0	0	0	0
3	6	1	0	0	0	0	0	0
3	6	1	0	0	0	0	0	0
2	6	1	0	0	0	0	0	0
2	6	1	0	0	0	0	0	0
3	6	1	0	0	0	0	0	0
3	5	1	0	0	0	0	0	0
1	6	1	0	0	0	0	0	0
3	6	1	0	0	0	0	0	0
3	6	1	0	0	0	0	0	0
2	6	1	0	0	0	0	0	0
3	6	1	0	0	0	0	0	0
2	6	1	0	0	0	0	0	0
2	6	1	0	0	0	0	0	0
4	6	1	0	0	0	0	0	0
4	6	1	0	0	0	0	0	0

3	6	1	0	0	0	0	0	0
2	6	1	0	0	0	0	0	0
4	5	1	0	0	0	0	0	0
5	6	1	1	0	0	0	0	0
5	6	1	1	1	0	0	0	0
4	6	1	1	1	0	0	0	0
5	6	1	1	0	0	0	0	0
5	6	1	1	0	0	0	0	0
5	6	1	1	1	0	1	0	0
3	6	0	1	0	0	0	0	0
5	6	1	1	0	0	0	0	0
5	6	1	1	0	0	0	0	0
4	6	1	1	0	0	0	0	0
5	6	0	1	0	0	0	0	0
5	6	1	1	0	0	0	0	0
5	6	0	0	1	0	0	0	0
1	6	1	1	0	0	0	0	0
4	6	0	1	0	0	0	0	0
5	6	1	1	0	0	0	0	0
2	6	1	0	0	0	0	0	0
4	6	0	1	0	0	0	0	0
4	6	1	1	0	0	0	0	0
5	3	0	1	0	0	0	0	1
5	6	1	1	0	0	0	0	0
4	6	0	1	0	0	0	0	0
4	6	0	0	1	0	0	0	0
5	6	1	1	0	0	0	0	0
3	6	1	1	0	0	0	0	0
4	6	0	1	0	0	0	0	0
5	6	1	1	0	0	0	0	0
3	6	1	0	0	0	0	0	0
5	6	1	1	0	0	0	0	0
4	6	0	1	0	0	0	0	0
5	6	1	1	0	0	0	0	0
5	6	1	1	1	0	0	0	0
5	6	1	0	0	0	0	0	0
5	6	0	1	0	0	0	0	0
3	6	0	1	0	0	0	0	0
5	6	1	1	0	0	0	0	0
4	6	1	1	0	0	0	0	0
5	6	1	0	0	0	0	0	0
5	6	1	1	0	0	0	0	0
4	6	0	1	0	0	0	0	0
5	6	0	1	0	0	0	0	0
5	6	1	1	1	0	0	0	0
5	6	1	1	0	0	0	0	0
4	6	0	1	0	0	0	0	0
5	6	1	1	0	0	0	0	0

5	6	1	1	1	0	0	0	0
5	6	1	1	0	0	0	0	0
5	6	1	1	1	0	0	0	0
5	6	1	0	1	0	0	0	0
3	6	0	1	0	0	0	0	0
5	6	1	1	0	0	0	0	0
5	6	1	1	0	0	0	0	0
5	6	1	1	0	0	0	0	0
4	6	0	1	0	0	0	0	0
4	6	1	1	0	0	0	0	0
5	6	1	1	0	0	0	0	0
5	6	0	1	0	0	0	0	0
5	6	1	1	0	0	0	0	0
4	6	0	1	0	0	0	0	0
5	6	1	1	0	0	0	0	0
4	6	1	1	0	0	0	0	0
4	6	1	1	0	0	0	0	0
3	6	0	1	0	0	0	0	0
4	6	1	1	0	0	0	0	0
2	6	1	0	0	0	0	0	0
3	6	1	1	0	0	0	0	0
5	6	1	1	0	0	0	0	0
5	6	0	1	0	0	0	0	0
5	6	1	1	0	0	0	0	0
3	1	0	1	0	0	0	0	0
4	6	0	1	0	0	0	0	0
5	6	1	1	0	0	0	0	0
5	6	1	1	0	0	0	0	0
3	6	0	1	0	0	0	0	0
5	6	1	1	0	0	0	0	0
5	6	0	1	0	0	0	0	0
5	6	1	1	0	0	0	0	0
3	6	0	1	0	0	0	0	0
5	6	1	1	0	0	0	0	0
2	6	1	1	0	0	0	0	0
5	6	1	0	1	0	0	0	0
5	6	1	1	1	0	0	0	0
5	6	1	0	1	0	0	0	0
5	6	1	1	1	0	0	0	0
4	1	1	1	0	0	0	0	0
3	6	0	1	0	0	0	0	0
4	6	1	1	0	0	0	0	0
4	6	1	1	0	0	0	0	0
2	1	0	1	0	0	0	0	0
3	1	1	1	0	1	0	0	0
4	1	1	1	0	1	0	0	0
3	6	1	1	0	0	0	0	0
1	6	1	0	0	0	0	0	0

2	6	1	0	0	0	0	0	0
3	6	1	0	0	0	0	0	0
3	6	1	0	0	0	0	0	0
2	6	1	0	0	0	0	0	0
2	6	1	0	0	0	0	0	0
2	6	1	0	0	0	0	0	0
4	6	0	1	0	0	0	0	0
4	6	0	1	0	0	0	0	0
4	6	1	0	0	0	0	0	0
5	6	1	1	0	0	0	0	0
4	6	1	1	0	0	0	0	0
3	6	1	1	0	0	0	0	0
3	6	1	1	0	0	0	0	0
4	6	0	1	0	0	0	0	0
3	1	0	1	0	1	0	0	0
4	1	1	1	0	0	0	0	0
4	6	1	1	0	0	0	0	0
2	1	1	1	0	1	0	0	0
3	1	0	1	0	1	0	0	0
4	6	1	1	0	0	0	0	0
2	1	1	1	0	0	0	0	0
2	1	1	1	0	1	0	0	0
3	1	1	1	0	1	0	0	0
3	1	1	1	0	1	0	0	0
4	1	1	1	0	0	0	0	0
3	1	0	1	0	1	0	0	0
4	1	1	1	0	1	0	0	0
1	1	1	1	0	0	0	0	0
2	1	0	1	0	1	0	0	0
3	1	1	1	0	1	0	0	0
1	1	1	1	0	1	0	0	0
2	1	0	0	0	1	0	0	0
3	1	1	0	0	0	0	0	0
4	3	0	1	0	0	0	0	1
4	6	1	1	0	0	0	0	0
1	6	1	1	0	0	0	0	0
3	6	1	1	0	0	0	0	0
4	1	1	0	0	1	0	0	0
4	6	1	1	0	0	0	0	0
3	6	1	1	0	0	0	0	0
3	6	1	1	0	0	0	0	0
2	4	1	1	0	0	0	0	0
3	6	1	1	0	0	0	0	0
3	6	1	1	0	0	0	0	0
3	6	1	0	0	0	0	0	0
3	1	1	1	0	1	0	0	0
2	1	1	1	0	0	0	0	0
3	1	0	1	0	0	0	0	0

2	1	0	1	0	1	0	0	0
4	1	1	1	0	1	0	0	0
3	1	1	1	0	1	0	0	0
4	1	1	1	0	1	0	0	0
3	1	1	1	0	0	0	0	0
4	4	0	0	0	1	0	0	0
5	6	1	1	0	0	0	0	0
4	1	1	1	0	1	0	0	0
3	1	1	1	0	1	0	0	0
4	1	1	1	0	1	0	0	0
5	1	1	1	0	1	0	0	0
2	1	1	0	0	1	0	0	0
2	1	1	1	0	0	0	0	0
3	1	1	1	0	1	0	0	0
4	1	1	1	0	1	0	0	0
3	1	0	1	0	1	0	0	0
3	1	1	1	0	1	0	0	0
4	1	1	1	0	1	0	0	0
5	1	1	1	0	1	0	0	0
4	1	1	1	0	1	0	0	0
5	1	1	1	0	0	0	0	0
3	1	1	1	0	1	0	0	0
1	1	1	0	0	1	0	0	0
2	1	0	0	0	1	0	0	0
3	6	1	1	0	0	0	0	0
4	1	1	1	0	1	0	0	0
4	1	1	1	0	1	0	0	0
2	1	0	1	0	0	0	0	0
3	1	1	1	0	1	0	0	0
3	1	1	1	0	0	0	0	0
4	1	1	1	0	1	0	0	0
5	1	1	1	0	1	0	0	0
5	1	1	1	0	1	0	0	0
2	1	1	1	0	1	0	0	0
4	1	1	1	0	1	0	0	0
3	1	1	1	0	1	0	0	0
3	1	1	1	0	1	0	0	0
5	1	1	1	0	0	0	0	0
3	1	1	1	0	1	0	0	0
5	6	1	1	0	0	0	0	0
4	6	1	1	1	0	0	0	0
5	6	1	1	1	0	0	0	0
3	6	0	1	0	0	0	0	0
5	6	1	1	1	0	0	0	0
5	6	1	1	0	0	0	0	0
5	6	1	1	0	0	0	0	0
3	1	0	1	0	0	0	0	0
4	6	1	1	0	0	0	0	0

4	6	1	1	0	0	0	0	0
5	6	1	1	0	0	0	0	0
4	1	0	1	0	1	0	0	0
4	1	1	1	0	1	0	0	0
4	1	1	1	0	1	0	0	0
4	1	1	1	0	1	0	0	0
3	1	1	1	0	1	0	0	0
3	1	1	1	0	1	0	0	0
3	1	1	1	0	1	0	0	0
3	1	1	1	0	1	0	0	0
1	1	1	1	0	0	0	0	0
1	1	1	1	0	1	0	0	0
2	1	1	1	0	1	0	0	0
4	1	1	1	0	1	0	0	0
4	3	0	1	0	0	0	0	1
4	1	1	1	0	1	0	0	0
3	1	1	0	0	1	0	0	0
3	1	1	1	0	1	0	0	0
2	1	1	1	0	1	0	0	0
3	1	1	1	0	1	0	0	0
1	1	1	1	0	1	0	0	0
3	1	1	1	0	1	0	0	0
2	1	0	1	0	1	0	0	0
2	6	1	1	0	0	0	0	0
1	1	0	1	0	1	0	0	0
3	1	1	1	0	1	0	0	0
3	1	1	1	0	1	0	0	0
5	6	1	1	0	0	0	0	0
3	1	1	1	0	1	0	0	0
3	4	1	1	0	0	0	0	0
2	1	0	1	0	1	0	0	0
2	1	1	1	0	1	0	0	0
4	1	1	1	0	1	0	0	0

HS	DC	Apnea	CA	CH	Paraplegia	AF	WED	ARF
0	0	0	0	0	0	0	0	0
0	0	0	0	0	0	0	0	0
0	0	0	0	0	0	0	0	0
0	0	0	0	0	0	0	0	0
0	0	0	0	0	0	0	0	0
0	0	0	0	0	0	0	0	0
0	0	0	0	0	0	0	0	0
0	0	0	0	0	0	0	0	0
0	0	0	0	0	0	0	0	0
0	1	0	0	0	0	0	0	0
0	0	0	0	0	0	0	0	0
0	0	0	0	0	0	0	0	0
0	1	0	0	0	0	0	0	0
0	0	0	0	0	0	0	0	0
0	0	0	0	0	0	0	0	0
0	0	0	0	0	0	0	0	0
0	0	0	0	0	0	0	0	0
0	0	0	0	0	0	0	0	0
0	0	0	0	0	0	0	0	0
0	0	0	0	0	0	0	0	0
0	0	0	0	0	0	0	0	0
0	0	0	0	0	0	0	0	0
0	0	0	0	0	0	0	0	0
0	0	0	0	0	0	0	0	0
0	0	0	0	0	0	0	0	0
0	0	0	0	0	0	0	0	0
0	0	0	0	0	0	0	0	0
0	0	0	0	0	0	0	0	0
0	0	0	0	0	0	0	0	0
0	0	0	0	0	0	0	0	0
0	0	0	0	0	0	0	0	0
0	0	0	0	0	0	0	0	0
0	0	0	0	0	0	0	0	0
0	0	0	0	0	0	0	0	0
0	0	0	0	0	0	0	0	0
0	0	0	0	0	0	0	0	0
0	1	0	0	0	0	0	0	0
0	1	0	0	0	0	0	0	0
0	1	0	0	0	0	0	0	0
0	1	0	0	0	0	0	0	0
0	1	0	0	0	0	0	0	0
0	0	0	0	0	0	0	0	0
0	1	0	0	0	0	0	0	0
0	1	0	0	0	0	0	0	0
0	1	0	0	0	0	0	0	0
0	1	0	0	0	0	0	0	0
0	0	0	0	0	0	0	0	0

0	0	0	0	0	0	0	0	0
0	1	0	0	0	0	0	0	0
0	1	0	0	0	0	0	0	0
0	1	0	0	0	0	0	0	0
0	1	0	0	0	0	0	0	0
0	0	0	0	0	0	0	0	0
0	1	0	0	1	0	0	0	0
0	0	0	0	0	0	0	0	0
0	1	0	0	0	0	0	0	0
0	1	0	0	0	0	0	0	0
0	0	0	0	0	0	0	0	0
0	1	0	0	0	0	0	0	0
0	1	0	0	0	0	0	0	0
0	1	0	0	1	0	0	0	0
0	0	0	0	0	0	0	0	0
0	0	0	0	0	0	0	0	0
0	0	0	0	0	0	0	0	0
0	0	0	0	0	0	0	0	0
0	0	0	0	0	0	0	0	0
0	1	0	0	0	0	0	0	0
0	1	0	0	0	0	0	0	0
0	1	0	0	0	0	0	0	0
0	1	0	0	0	0	0	0	0
0	0	0	0	0	0	0	0	0
0	0	0	0	0	0	0	0	0
0	0	0	0	0	0	0	0	0
0	0	0	0	0	0	0	0	0
0	0	0	0	0	0	0	0	0
0	0	0	0	0	0	0	0	0
0	1	1	0	1	0	0	0	0
0	0	0	0	0	0	0	0	0
0	0	0	0	0	0	0	0	0
0	1	0	0	0	0	0	0	0
0	1	0	0	0	0	0	0	0
0	0	0	0	0	0	0	0	0
0	0	0	0	0	0	0	0	0
0	0	0	0	0	0	0	0	0
0	0	0	0	0	0	0	0	0
0	0	0	0	0	0	0	0	0
0	0	0	0	0	0	0	0	0
0	0	0	0	0	0	0	0	0
0	0	0	0	0	0	0	0	0
0	1	0	0	0	0	0	0	0
0	1	0	0	0	0	0	0	0
0	0	0	0	0	0	0	0	0
0	0	0	0	0	0	0	0	0
0	0	0	0	0	0	0	0	0
0	0	0	0	0	0	0	0	0

0	0	0	0	0	0	0	0	0
0	0	0	0	0	0	0	0	0
0	0	0	0	0	0	0	0	0
0	0	0	0	0	0	0	0	0
0	0	0	0	0	0	0	0	0
0	0	0	0	0	0	0	0	0
0	0	0	0	0	0	0	0	0
0	0	0	0	0	0	0	0	0
1	0	0	0	0	0	0	0	0
0	0	0	0	0	0	0	0	0
0	0	0	0	0	0	0	0	0
0	0	0	0	0	0	0	0	0
0	0	0	0	0	0	0	0	0
0	1	0	0	0	0	0	0	0
0	0	0	0	0	0	0	0	0
0	0	0	0	0	0	0	0	0
0	0	0	0	0	0	0	0	0
0	0	0	0	0	0	0	0	0
0	0	0	0	0	0	0	0	0
0	0	0	0	0	0	0	0	0
0	0	0	0	0	0	0	0	0
0	0	0	0	0	0	0	0	0
0	0	0	0	0	0	0	0	0
0	0	0	0	0	0	0	0	0
0	0	0	0	0	0	0	0	0
0	0	0	0	0	0	0	0	0
0	0	0	0	0	0	0	0	0
0	0	0	0	0	0	0	0	0
0	0	0	0	0	0	0	0	0
0	1	0	0	0	0	0	0	0
0	0	0	0	0	0	0	0	0
0	1	0	0	0	0	0	0	0
0	1	0	0	0	0	0	0	0
0	0	0	0	0	0	0	0	0
0	0	0	0	0	0	0	0	0
0	0	0	0	0	0	0	0	0
0	0	0	0	0	0	0	0	0
0	0	0	0	0	0	0	0	0
0	0	0	0	0	0	0	0	0
0	0	0	0	0	0	0	0	0
0	0	0	0	0	0	0	0	0
0	0	0	0	0	0	0	0	0
0	1	0	0	0	0	0	0	0
0	0	0	0	0	0	0	0	0
0	0	0	0	0	0	0	0	0
0	0	0	0	0	0	0	0	0
0	0	0	0	0	0	0	0	0
1	1	0	0	0	0	0	0	0

0	1	0	0	0	0	0	0	0
0	0	0	0	0	0	0	0	0
0	0	0	0	0	0	0	0	0
0	1	0	0	0	0	0	0	0
0	0	0	0	0	0	0	0	0
0	1	0	0	0	0	0	0	0
0	1	0	0	0	0	0	0	0
0	0	0	0	0	0	0	0	0
0	0	0	0	0	0	0	0	0
0	0	0	0	0	0	0	0	0
0	0	0	0	0	0	0	0	0
0	0	0	0	0	0	0	0	0
0	0	0	0	0	0	0	0	0
0	0	0	0	0	0	0	0	0
0	0	0	0	0	0	0	0	0
0	0	0	0	0	0	0	0	0
0	0	0	0	0	0	0	0	0
0	0	0	0	0	0	0	0	0
0	0	0	0	0	0	0	0	0
0	0	0	0	0	0	0	0	0
0	0	0	0	0	0	0	0	0
0	1	0	0	0	0	0	0	0
0	1	0	0	0	0	0	0	0
0	1	0	0	0	0	0	0	0
0	0	0	0	0	0	0	0	0
0	0	0	0	0	0	0	0	0
0	0	0	0	0	0	0	0	0
0	0	0	0	0	0	0	0	0
0	0	0	0	0	0	0	0	0
0	0	0	0	0	0	0	0	0
0	0	0	0	0	0	0	0	0
0	0	0	0	0	0	0	0	0
0	0	0	0	0	0	0	0	0
0	0	0	0	0	0	0	0	0
0	0	0	0	0	0	0	0	0
0	0	0	0	0	0	0	0	0
0	0	0	0	0	0	0	0	0
0	0	0	0	0	0	0	0	0
0	0	0	0	0	0	0	0	0
0	0	0	0	0	0	0	0	0
0	0	0	0	0	0	0	0	0
0	0	0	0	0	0	0	0	0
0	0	0	0	0	0	0	0	0
0	0	0	0	0	0	0	0	0
0	0	0	0	0	0	0	0	0
0	0	0	0	0	0	0	0	0
0	0	0	0	0	0	0	0	0
0	0	0	0	0	0	0	0	0

0	0	0	0	0	0	0	0	0
0	0	0	0	0	0	0	0	0
0	0	0	0	0	0	0	0	0
0	0	0	0	0	0	0	0	0
1	0	0	0	0	0	0	0	0
0	0	0	0	0	0	0	0	0
0	0	0	0	0	0	0	0	0
0	0	0	0	0	0	0	0	0
0	0	0	0	0	0	0	0	0
0	1	0	0	0	0	0	0	0
0	0	0	0	0	0	0	0	0
0	0	0	0	0	0	0	0	0
0	0	0	0	0	0	0	0	0
0	0	0	0	0	0	0	0	0
0	0	0	0	0	0	0	0	0
0	0	0	0	0	0	0	0	0
0	0	0	0	0	0	0	0	0
0	0	0	0	0	0	0	0	0
0	0	0	0	0	0	0	0	0
0	0	0	0	0	0	0	0	0
0	0	0	0	0	0	0	0	0
0	0	0	0	0	0	0	0	0
0	0	0	0	0	0	0	0	0
0	0	0	0	0	0	0	0	0
0	0	0	0	0	0	0	0	0
0	0	0	0	0	0	0	0	0
0	0	0	0	0	0	0	0	0
0	0	0	0	0	0	0	0	0
0	0	0	0	0	0	0	0	0
0	0	0	0	0	0	0	0	0
0	0	0	0	0	0	0	0	0
0	0	0	0	0	0	0	0	0
0	0	0	0	0	0	0	0	0
0	1	0	0	0	0	0	0	0
0	1	0	0	0	0	0	0	0
0	1	0	0	0	0	0	0	0
0	0	0	0	0	0	0	0	0
0	0	0	0	0	0	0	0	0
0	1	0	0	0	0	0	0	0
0	1	0	0	0	0	0	0	0
0	0	0	0	0	0	0	0	0
0	0	0	0	0	0	0	0	0
0	0	0	0	0	0	0	0	0
0	0	0	0	0	0	0	0	0
0	1	0	0	0	0	0	0	0
0	0	0	0	0	0	0	0	0
0	1	0	0	0	0	0	0	0
0	0	0	0	0	0	0	0	0

0	1	0	0	0	0	0	0	0
0	1	0	0	0	0	0	0	0
0	1	0	0	0	0	0	0	0
0	1	0	0	0	0	0	0	0
0	1	0	0	0	0	0	0	0
0	0	0	0	0	0	0	0	0
0	0	0	0	0	0	0	0	0
0	0	0	0	0	0	0	0	0
0	1	0	0	0	0	0	0	0
0	1	0	0	0	0	0	0	0
0	0	0	0	0	0	0	0	0
0	0	0	0	0	0	0	0	0
0	1	0	0	0	0	0	0	0
0	1	0	0	0	0	0	0	0
0	0	0	0	0	0	0	0	0
0	0	0	0	0	0	0	0	0
0	0	0	0	0	0	0	0	0
0	0	0	0	0	0	0	0	0
0	0	0	0	0	0	0	0	0
0	0	0	0	0	0	0	0	0
0	0	0	0	0	0	0	0	0
0	0	0	0	0	0	0	0	0
0	0	0	0	0	0	0	0	0
0	0	0	0	0	0	0	0	0
0	0	0	0	0	0	0	0	0
0	0	0	0	0	0	0	0	0
0	0	0	0	0	0	0	0	0
0	0	0	0	0	0	0	0	0
0	0	0	0	0	0	0	0	0
0	0	0	0	0	0	0	0	0
0	0	0	0	0	0	0	0	0
0	0	0	0	0	0	0	0	0
0	0	0	0	0	0	0	0	0
0	1	0	0	0	0	0	0	0
0	0	0	0	0	0	0	0	0
0	0	0	0	0	0	0	0	0
0	0	0	0	0	0	0	0	0
0	0	0	0	0	0	0	0	0
0	1	0	0	0	0	0	0	0
0	0	0	0	0	0	0	0	0
0	0	0	0	0	0	0	0	0
0	0	0	0	0	0	0	0	0
0	1	0	0	0	0	0	0	0
0	1	0	0	0	0	0	0	0
0	1	0	0	0	0	0	0	0
0	1	0	0	0	0	0	0	0
0	1	0	0	0	0	0	0	0
0	0	0	0	0	0	0	0	0

0	0	0	0	0	0	0	0	0
0	0	0	0	0	0	0	0	0
0	0	0	0	0	0	0	0	0
0	0	0	0	0	0	0	0	0
0	1	0	0	0	0	0	0	0
0	0	0	0	0	0	0	0	0
0	0	0	0	0	0	0	0	0
0	0	0	0	0	0	0	0	0
0	1	0	0	0	0	0	0	0
0	0	0	0	0	0	0	0	0
0	1	0	0	0	0	0	0	0
0	1	0	0	0	0	0	0	0
0	0	0	0	0	0	0	0	0
1	0	0	0	0	0	0	0	0
0	0	0	0	0	0	0	0	0
0	1	0	0	0	0	0	0	0
0	0	0	0	0	0	0	0	0
0	1	0	0	0	0	0	0	0
0	1	0	0	0	0	0	0	0
0	0	0	0	0	0	0	0	0
0	0	0	0	0	0	0	0	0
0	0	0	0	0	0	0	0	0
0	0	0	0	0	0	0	0	0
0	0	0	0	0	0	0	0	0
0	1	0	0	0	0	0	0	0
0	0	0	0	0	0	0	0	0
0	0	0	0	0	0	0	0	0
0	1	0	0	0	0	0	0	0
0	1	0	0	0	0	0	0	0
0	0	0	0	0	0	0	0	0
0	0	0	0	0	0	0	0	0
0	0	0	0	0	0	0	0	0
0	1	0	0	0	0	0	0	0
0	0	0	0	0	0	0	0	0
0	0	0	0	0	0	0	0	0
0	0	0	0	0	0	0	0	0
0	1	0	0	0	0	0	0	0
0	0	0	0	0	0	0	0	0
1	1	0	0	0	0	0	0	0
0	0	0	0	0	0	0	0	0
0	0	0	0	0	0	0	0	0
0	0	0	0	0	0	0	0	0
0	0	0	0	0	0	0	0	0
0	1	0	0	0	0	0	0	0
0	1	0	0	0	0	0	0	0
0	1	0	0	0	0	0	0	0
0	0	0	0	0	0	0	0	0
0	0	0	0	0	0	0	0	0

0	0	0	0	0	0	0	0	0
0	0	0	0	0	0	0	0	0
0	0	0	0	0	0	0	0	0
0	0	0	0	0	0	0	0	0
0	0	0	0	0	0	0	0	0
0	0	0	0	0	0	0	0	0
0	0	0	0	0	0	0	0	0
0	0	0	0	0	0	0	0	0
0	0	0	0	0	0	0	0	0
0	1	0	0	0	0	0	0	0
0	0	0	0	0	0	0	0	0
0	1	0	0	0	0	0	0	0
0	0	0	0	0	0	0	0	0
0	0	0	0	0	0	0	0	0
0	0	0	0	0	0	0	0	0
0	0	0	0	0	0	0	0	0
0	0	0	0	0	0	0	0	0
0	0	0	0	0	0	0	0	0
0	0	0	0	0	0	0	0	0
0	0	0	0	0	0	0	0	0
0	0	0	0	0	0	0	0	0
0	0	0	0	0	0	0	0	0
0	0	0	0	0	0	0	0	0
0	0	0	0	0	0	0	0	0
0	0	0	0	0	0	0	0	0
0	0	0	0	0	0	0	0	0
0	0	0	0	0	0	0	0	0
0	0	0	0	0	0	0	0	0
0	0	0	0	0	0	0	0	0
0	0	0	0	0	0	0	0	0
0	0	0	0	0	0	0	0	0
0	0	0	0	0	0	0	0	0
0	0	0	0	0	0	0	0	0
0	0	0	0	0	0	0	0	0
0	0	0	0	0	0	0	0	0
0	0	0	0	0	0	0	0	0
0	0	0	0	0	0	0	0	0
0	0	0	0	0	0	0	0	0
0	1	0	0	0	0	0	0	0
0	1	0	0	0	0	0	0	0
0	0	0	0	0	0	0	0	0
0	1	0	0	0	0	0	0	0
0	1	0	0	1	0	0	0	0
0	0	0	0	0	0	0	0	0
0	0	0	0	0	0	0	0	0
0	1	0	0	0	0	0	0	0
0	0	0	0	0	0	0	0	0
0	1	0	0	0	0	0	0	0

0	1	0	0	0	0	0	0	0
0	1	0	0	0	0	0	0	0
0	0	0	0	0	0	0	0	0
0	1	0	0	0	0	0	0	0
0	0	0	0	0	0	0	0	0
0	0	0	0	0	0	0	0	0
0	1	0	0	0	0	0	0	0
0	0	0	0	0	0	0	0	0
0	0	0	0	0	0	0	0	0
0	1	0	0	0	0	0	0	0
0	0	0	0	0	0	0	0	0
0	0	0	0	0	0	0	0	0
0	0	0	0	0	0	0	0	0
0	0	0	0	0	0	0	0	0
0	0	0	0	0	0	0	0	0
0	1	0	0	1	0	0	0	0
0	0	0	0	0	0	0	0	0
0	1	0	0	0	0	0	0	0
0	1	0	0	0	0	0	0	0
0	1	0	0	0	0	0	0	0
0	0	0	0	0	0	0	0	0
0	0	0	0	0	0	0	0	0
0	0	0	0	0	0	0	0	0
0	0	0	0	0	0	0	0	0
0	1	0	0	0	1	0	0	0
0	0	0	0	0	0	0	0	0
0	0	0	0	0	0	0	0	0
0	0	0	0	0	0	0	0	0
0	0	0	0	0	0	0	0	0
0	0	0	0	0	0	0	0	0
0	0	0	0	0	0	0	0	0
0	0	0	0	0	0	0	0	0
0	0	0	0	0	1	0	0	0
0	0	0	0	0	0	0	0	0
0	0	0	0	0	0	0	0	0
0	0	0	0	0	0	0	0	0
0	0	0	0	0	0	0	0	0
0	0	0	0	0	0	0	0	0
0	0	0	0	0	0	0	0	0
0	0	0	0	0	0	0	0	0
0	0	0	0	0	0	0	0	0
0	0	0	0	0	0	0	0	0
0	0	0	0	0	0	0	0	0
0	0	0	0	0	0	0	0	0
0	1	0	0	1	0	0	0	0
0	0	0	0	0	0	0	0	0
0	0	0	0	0	0	0	0	0
0	0	0	0	0	0	0	0	0

0	0	0	0	0	0	0	0	0
0	1	0	0	0	0	0	0	0
0	0	0	0	0	0	0	0	0
0	0	0	0	0	0	0	0	0
0	0	0	0	0	0	0	0	0
0	0	0	0	0	0	0	0	0
0	1	0	0	0	0	0	0	0
0	0	0	0	0	0	0	0	0
0	0	0	0	0	0	0	0	0
0	0	0	0	0	0	0	0	0
0	1	0	0	0	0	0	0	0
0	0	0	0	0	0	0	0	0
0	1	0	0	0	0	0	0	0
0	0	0	0	0	0	0	0	0
0	0	0	0	0	0	0	0	0
0	1	0	0	0	0	0	0	0
0	0	0	0	0	0	0	0	0
0	0	0	0	0	0	0	0	0
0	0	0	0	0	0	0	0	0
0	1	0	0	0	0	0	0	0
0	0	0	0	0	0	0	0	0
0	0	0	0	0	0	0	0	0
0	0	0	0	0	0	0	0	0
0	1	0	1	0	0	1	0	1
0	0	0	0	0	0	0	0	0
0	1	0	0	0	0	0	0	0
0	0	0	0	0	0	0	0	0
0	0	0	0	0	0	0	0	0
0	0	0	0	0	0	0	0	0
0	0	0	0	0	0	0	0	0
0	0	0	0	0	0	0	0	0
0	0	0	0	0	0	0	0	0
0	0	0	0	0	0	0	0	0
0	0	0	0	0	0	0	0	0
0	0	0	0	0	0	0	0	0
0	0	0	0	0	0	0	0	0
0	0	0	0	0	0	0	0	0
0	0	0	0	0	0	0	0	0
0	0	0	0	0	0	0	0	0
0	0	0	0	0	0	0	0	0
0	0	0	0	0	0	0	0	0
0	0	0	0	0	0	0	0	0
0	0	0	0	0	0	0	0	0
0	0	0	0	0	0	0	0	0
0	0	0	0	0	0	0	0	0
0	0	0	0	0	0	0	0	0
0	0	0	0	0	0	0	0	0
0	0	0	0	0	0	0	0	0

0	0	0	0	0	0	0	0	0
0	0	0	0	0	0	0	0	0
0	1	0	0	0	0	0	0	0
0	0	0	0	0	0	0	0	0
0	0	0	0	0	0	0	0	0
0	0	0	0	0	0	0	0	0
0	0	0	0	0	0	0	0	0
0	0	0	0	0	0	0	0	0
0	0	0	0	0	0	0	0	0
0	0	0	0	0	0	0	0	0
0	0	0	0	0	0	0	0	0
0	0	0	0	0	0	0	0	0
0	0	0	0	0	0	0	0	0
0	0	0	0	0	0	0	0	0
0	0	0	0	0	0	0	0	0
0	1	0	0	0	0	0	0	0
0	0	0	0	0	0	0	0	0
0	0	0	0	0	0	0	0	0
0	0	0	0	0	0	0	0	0
0	0	0	0	0	0	0	0	0
0	0	0	0	0	0	0	0	0
0	0	0	0	0	1	0	0	0
0	0	0	0	0	0	0	0	0
0	0	0	0	0	0	0	0	0
0	0	0	0	0	0	0	0	0
0	0	0	0	0	0	0	0	0
0	0	0	0	0	0	0	0	0
0	0	0	0	0	0	0	0	0
0	0	0	0	0	0	0	0	0
0	0	0	0	0	0	0	0	0
0	0	0	0	0	0	0	0	0
0	0	0	0	0	0	0	0	0
0	0	0	0	0	0	0	0	0
0	0	0	0	0	0	0	0	0
0	0	0	0	0	0	0	0	0
0	0	0	0	0	0	0	0	0
0	0	0	0	0	0	0	0	0
0	0	0	0	0	0	0	0	0
0	0	0	0	0	0	0	0	0
0	0	0	0	0	0	0	0	0
0	1	0	0	0	0	0	0	0
0	0	0	0	0	0	0	0	0
0	0	0	0	0	0	0	0	0
0	0	0	0	0	0	0	0	0
0	0	0	0	0	0	0	0	0
0	0	0	0	0	0	0	0	0
0	0	0	0	0	0	0	0	0
0	0	0	0	0	0	0	0	0

0	0	0	0	0	0	0	0	0
0	0	0	0	0	0	0	0	0
0	1	0	0	0	0	0	0	0
0	0	0	0	0	0	0	0	0
0	0	0	0	0	0	0	0	0
0	1	0	0	0	0	0	0	0
0	1	0	0	0	0	0	0	0
0	0	0	0	0	0	0	0	0
0	0	0	0	0	0	0	0	0
0	0	0	0	0	0	0	0	0
0	1	0	0	0	0	0	0	0
0	0	0	0	0	0	0	0	0
0	0	0	0	0	0	0	0	0
0	0	0	0	0	0	0	0	0
0	0	0	0	0	0	0	0	0
0	1	0	0	0	0	0	0	0
0	1	0	0	0	0	0	0	0
0	0	0	0	0	0	0	0	0
0	0	0	0	0	0	0	0	0
0	0	0	0	0	0	0	0	0
0	1	0	0	0	0	0	0	0
0	0	0	0	0	0	0	0	0
0	0	0	0	0	0	0	0	0
0	0	0	0	0	0	0	0	0
0	0	0	0	0	0	0	0	0
0	1	0	0	0	0	0	0	0
0	1	0	0	0	0	0	0	0
0	0	0	0	0	0	0	0	0
0	0	0	0	0	0	0	0	0
0	1	0	0	1	0	0	0	1
0	1	0	0	0	0	0	0	0
0	1	0	0	0	0	0	0	0
0	1	0	0	1	0	0	0	0
0	1	0	0	0	0	0	0	0
0	1	0	0	1	0	0	0	0
0	1	0	0	0	0	0	0	0
0	1	0	0	0	0	0	0	0
0	0	0	0	0	0	0	0	0
0	0	0	0	0	0	0	0	0
0	1	0	0	0	0	0	0	0
0	1	0	0	0	0	0	0	0
0	0	0	0	0	0	0	0	0
0	0	0	0	0	0	0	0	0
0	1	0	0	0	0	0	0	0
0	0	0	0	0	0	0	0	0
0	1	0	0	0	0	0	0	0
0	0	0	0	0	0	0	0	0
0	1	0	0	0	0	0	0	0

0	0	0	0	0	0	0	0	0
0	0	0	0	0	0	0	0	0
0	0	0	0	0	0	0	0	0
0	0	0	0	0	0	0	0	0
0	1	0	0	0	0	0	0	0
0	1	0	0	1	0	0	0	0
0	1	0	0	0	0	0	0	0
0	1	0	0	0	0	0	0	0
0	1	0	0	0	0	0	0	0
0	0	0	0	0	0	0	0	0
0	0	0	0	0	0	0	0	0
0	0	0	0	0	0	0	0	0
0	0	0	0	0	0	0	0	0
0	0	0	0	0	0	0	0	0
0	0	0	0	0	0	0	0	0
0	0	0	0	0	0	0	0	0
0	0	0	0	0	0	0	0	0
0	1	0	0	0	0	0	0	0
0	0	0	0	0	0	0	0	0
0	1	0	0	0	0	0	0	0
0	0	0	0	0	0	0	0	0
0	0	0	0	0	0	0	0	0
0	0	0	0	0	0	0	0	0
0	0	0	0	0	0	0	0	0
0	0	0	0	0	0	0	0	0
0	0	0	0	0	1	0	0	0
0	0	0	0	0	0	0	0	0
0	1	0	0	0	0	0	0	0
0	0	0	0	0	0	0	0	0
0	0	0	0	0	0	0	0	0
0	0	0	0	0	0	0	0	0
0	0	0	0	0	0	0	0	0
0	0	0	0	0	0	0	0	0
0	0	0	0	0	0	0	0	0
0	0	0	0	0	0	0	0	0
0	0	0	0	0	0	0	0	0
0	0	0	0	0	0	0	0	0
0	0	0	0	0	0	0	0	0
0	0	0	0	0	0	0	0	0
0	0	0	0	0	0	0	0	0
0	0	0	0	0	0	0	0	0
0	1	0	0	0	0	0	0	0
0	1	0	0	0	0	0	0	0
0	0	0	0	0	0	1	0	0
0	0	0	0	0	0	0	0	0
0	0	0	0	0	0	0	0	0
0	0	0	0	0	0	0	0	0
0	0	0	0	0	0	0	0	0

0	1	0	0	0	0	0	0	0
0	1	0	0	0	0	0	0	0
0	0	0	0	0	0	0	0	0
0	1	0	0	0	0	0	0	0
0	1	0	0	0	0	0	0	0
0	1	0	0	0	0	0	0	0
0	0	0	0	0	0	0	0	0
0	0	0	0	0	0	0	0	0
0	0	0	0	0	0	0	0	0
0	0	0	0	0	0	0	0	0
0	0	0	0	0	0	0	0	0
0	1	0	0	0	0	0	0	0
0	0	0	0	0	0	0	0	0
0	0	0	0	0	0	0	0	0
0	0	0	0	0	0	0	0	0
0	0	0	0	0	0	0	0	0
0	0	0	0	0	0	0	0	0
0	1	0	0	0	0	0	0	0
0	0	0	0	0	1	0	0	0
0	0	0	0	0	0	0	0	0
0	0	0	0	0	0	0	0	0
0	0	0	0	0	1	0	0	0
0	0	0	0	0	0	0	0	0
0	0	0	0	0	0	0	0	0
0	0	0	0	0	0	0	0	0
0	0	0	0	0	0	0	0	0
0	0	0	0	0	0	0	0	0
0	0	0	0	0	0	0	0	0
0	0	0	0	0	0	0	0	0
0	0	0	0	0	0	0	0	0
0	0	0	0	0	0	0	0	0
0	0	0	0	0	1	0	0	0
0	1	0	0	0	0	0	0	0
0	0	0	0	0	0	0	0	0
0	0	0	0	0	0	0	0	0
0	0	0	0	0	0	0	0	0
0	0	0	0	0	0	0	0	0
0	0	0	0	0	0	0	0	0
0	0	0	0	0	0	0	0	0
0	0	0	0	0	0	0	0	0
0	0	0	0	0	0	0	0	0
0	0	0	0	0	0	0	0	0
0	1	0	0	0	0	0	0	0
0	0	0	0	0	0	0	0	0
0	0	0	0	0	0	0	0	0
0	0	0	0	0	0	0	0	0
0	0	0	0	0	0	0	0	0
0	0	0	0	0	0	0	0	0

0	0	0	0	0	0	0	0	0
0	0	0	0	0	0	0	0	0
0	0	0	0	0	0	0	0	0
0	1	0	0	0	0	0	0	0
0	0	0	0	0	0	0	0	0
0	1	0	0	0	0	0	0	0
0	0	0	0	0	0	0	0	0
0	0	0	0	0	0	0	0	0
0	0	0	0	0	0	0	0	0
0	0	0	0	0	0	0	0	0
0	0	0	0	0	0	0	0	0
0	0	0	0	0	1	0	0	0
0	1	0	0	0	0	0	1	0
0	0	0	0	0	0	0	0	0
0	1	0	0	0	0	0	0	0
0	0	0	0	0	0	0	0	0
0	1	0	0	0	0	0	0	0
0	0	0	0	0	0	0	0	0
0	0	0	0	1	0	0	0	0
0	0	0	0	0	1	0	0	0
0	1	0	0	1	0	0	0	0
0	0	0	0	0	0	0	0	0
0	0	0	0	0	0	0	0	0
0	0	0	0	0	0	0	0	0
0	0	0	0	0	0	0	0	0
0	0	0	0	0	0	0	0	0
0	0	0	0	0	0	0	0	0
0	0	0	0	0	0	0	0	0
0	0	0	0	0	0	0	0	0
0	0	0	0	0	0	0	0	0
0	0	0	0	0	0	0	0	0
0	0	0	0	0	0	0	0	0
0	0	0	0	0	0	0	0	0
0	1	0	0	0	0	0	0	0
0	0	0	0	0	0	0	0	0
0	0	0	0	0	0	0	0	0
0	0	0	0	0	0	0	0	0
0	0	0	0	0	0	0	0	0
0	1	0	0	0	0	0	0	0
0	0	0	0	0	0	0	0	0
0	1	0	0	0	0	0	0	0
0	0	0	0	0	0	0	0	0
0	0	0	0	0	0	0	0	0
0	0	0	0	0	0	0	0	0
0	1	0	0	0	0	0	0	0
0	0	0	0	0	0	0	0	0
0	0	0	0	0	0	0	0	0
0	0	0	0	0	0	0	0	0

0	0	0	0	0	0	0	0	0
0	0	0	0	0	0	0	0	0
0	0	0	0	0	0	0	0	0
0	0	0	0	0	0	0	0	0
0	0	0	0	0	0	0	0	0
0	0	0	0	0	0	0	0	0
0	0	0	0	0	0	0	0	0
0	0	0	0	0	0	0	0	0
0	0	0	0	0	0	0	0	0
0	1	0	0	0	0	0	0	0
1	0	0	0	0	0	0	0	0
0	0	0	0	0	0	0	0	0
0	0	0	0	0	0	0	0	0
0	1	0	0	0	0	0	0	0
0	1	0	0	0	0	0	0	0
0	0	0	0	0	0	0	0	0
0	0	0	0	0	0	0	0	0
0	1	0	0	0	0	0	0	0
0	1	0	0	0	0	0	0	0
0	0	0	0	0	0	0	0	0
0	0	0	0	0	0	0	0	0
0	1	0	0	0	0	0	0	0
0	1	0	0	0	0	0	0	0
0	0	0	0	0	0	0	0	0
0	0	0	0	0	0	0	0	0
0	0	0	0	0	0	0	0	0
0	1	0	0	0	0	0	0	0
0	1	0	0	0	0	0	0	0
0	0	0	0	0	0	0	0	0
0	0	0	0	0	0	0	0	0
0	1	0	0	0	0	0	0	0
0	0	0	0	0	0	0	0	0
0	0	0	0	0	0	0	0	0
0	0	0	0	0	0	0	0	0
0	0	0	0	0	0	0	0	0
0	0	0	0	0	0	0	0	0
0	0	0	0	0	0	0	0	0
0	1	0	0	0	0	0	0	0
0	0	0	0	0	0	0	0	0
0	0	0	0	0	0	0	0	0
0	0	0	0	0	0	0	0	0
0	0	0	0	0	0	0	0	0
0	1	0	0	0	1	0	0	0
0	0	0	0	0	0	0	0	0
0	1	0	0	0	0	0	0	0
0	0	0	0	0	1	0	0	0
0	0	0	0	0	0	0	0	0
0	0	0	0	0	0	0	0	0

0	0	0	0	0	0	0	0	0
0	0	0	0	0	0	0	0	0
0	0	0	0	0	0	0	0	0
0	0	0	0	0	1	0	0	0
0	0	0	0	0	0	0	0	0
1	1	0	0	0	0	0	0	0
0	0	0	0	0	0	0	0	0
0	0	0	0	0	0	0	0	0
0	0	0	0	0	0	0	0	0
0	0	0	0	0	0	0	0	0
0	1	0	0	0	0	0	0	0
0	0	0	0	0	1	0	0	0
0	0	0	0	0	0	0	0	0
0	1	0	0	0	1	0	1	0
0	0	0	0	0	1	0	0	0
0	0	0	0	0	0	0	0	0
0	0	0	0	0	0	0	0	0
0	0	0	0	0	0	0	0	0
0	1	0	0	0	0	0	0	0
0	0	0	0	0	1	0	0	0
0	0	0	0	0	0	0	0	0
0	0	0	0	0	0	0	0	0
0	0	0	0	0	0	0	0	0
0	1	0	0	0	0	0	0	0
0	0	0	0	0	0	0	0	0
0	0	0	0	0	0	0	0	0
0	0	0	0	0	0	0	0	0
0	0	0	0	0	0	0	0	0
0	0	0	0	0	0	0	0	0
0	1	0	0	0	0	0	0	0
0	0	0	0	0	0	0	0	0
0	0	0	0	0	0	0	0	0
0	0	0	0	0	0	0	0	0
0	1	0	0	0	0	0	0	0
0	0	0	0	0	0	0	0	0
0	1	0	0	0	0	0	0	0
0	0	0	0	0	0	0	0	0
0	0	0	0	0	0	0	0	0
0	0	0	0	0	1	0	0	0
0	0	0	0	0	1	0	0	0
0	0	0	0	0	0	0	0	0
0	1	0	0	0	1	0	0	0
0	0	0	0	0	0	0	0	0
0	0	0	0	0	0	0	0	0
0	0	0	0	0	0	0	0	0
0	1	0	0	0	0	0	0	0
0	0	0	0	0	0	0	0	0
0	0	0	0	0	0	0	0	0

0	0	0	0	0	0	0	0	0
0	0	0	0	0	0	0	0	0
0	1	0	0	0	0	0	0	0
0	1	0	0	0	0	0	0	0
0	0	0	0	0	0	0	0	0
0	0	0	0	0	0	0	0	0
0	0	0	0	0	0	0	0	0
0	0	0	0	0	0	0	0	0
0	0	0	0	0	1	0	0	0
0	0	0	0	0	0	0	0	0
0	0	0	0	0	0	0	0	0
0	0	0	0	0	0	0	0	0
0	0	0	0	0	0	0	0	0
0	0	0	0	0	0	0	0	0
0	1	0	0	0	0	0	0	0
0	1	0	0	0	0	0	0	0
0	0	0	0	0	0	0	0	0
0	0	0	0	0	0	0	0	0
0	0	0	0	0	0	0	0	0
0	1	0	0	0	0	0	0	0
0	0	0	0	0	0	0	0	0
0	0	0	0	0	0	0	0	0
0	0	0	0	0	0	0	0	0
0	0	0	0	0	0	0	0	0
0	0	0	0	0	0	0	0	0
0	1	0	0	0	1	0	0	0
0	0	0	0	0	0	0	0	0
0	0	0	0	0	0	0	0	0
0	0	0	0	0	0	0	0	0
0	0	0	0	0	0	0	0	0
0	0	0	0	0	0	0	0	0
0	0	0	0	0	0	0	0	0
0	0	0	0	0	0	0	0	0
0	0	0	0	0	0	0	0	0
0	0	0	0	0	0	0	0	0
0	0	0	0	0	0	0	0	0
0	0	0	0	0	0	0	0	0
0	0	0	0	0	0	0	0	0
0	0	0	0	0	1	0	0	0
0	0	0	0	0	0	0	0	0
0	0	0	0	0	0	0	0	0
0	1	0	0	0	0	0	0	0
0	1	0	0	0	0	0	0	0
0	0	0	0	0	0	0	0	0
0	1	0	0	0	0	0	0	0
0	0	0	0	0	1	0	0	0
0	0	0	0	0	0	0	0	0
0	0	0	0	0	0	0	0	0

0	1	0	0	0	0	0	0	0
0	0	0	0	0	0	0	0	0
0	0	0	0	0	0	0	0	0
0	0	0	0	0	0	0	0	0
0	0	0	0	0	1	0	0	0
0	0	0	0	0	0	0	0	0
0	0	0	0	0	0	0	0	0
0	0	0	0	0	0	0	0	0
0	0	0	0	0	0	0	0	0
0	1	0	0	0	0	0	0	0
0	0	0	0	0	0	0	0	0
0	1	0	0	0	0	0	0	0
0	1	0	0	0	0	0	0	0
0	1	0	0	0	0	0	0	0
0	0	0	0	0	0	0	0	0
0	0	0	0	0	0	0	0	0
0	1	0	0	0	1	0	0	0
0	1	0	0	0	1	0	0	0
0	0	0	0	0	0	0	0	0
0	0	0	0	0	1	0	0	0
0	0	0	0	0	0	0	0	0
0	0	0	0	0	0	1	1	0
0	0	0	0	0	0	0	0	0
0	0	0	0	0	0	0	0	0
0	0	0	0	0	0	0	0	0
0	0	0	0	0	0	0	0	0
0	1	0	0	0	1	0	0	0
0	1	0	0	0	0	0	0	0
0	0	0	0	0	0	0	0	0
0	1	0	0	0	1	0	0	0
0	0	0	0	0	0	0	0	0
0	0	0	0	0	0	0	0	0
0	0	0	0	0	0	0	0	0
0	1	0	0	0	0	0	0	0
0	0	0	0	0	1	0	0	0
0	1	0	0	0	0	0	0	0
0	1	0	0	0	1	0	0	0
0	1	0	0	0	0	0	0	0
0	0	0	0	0	1	0	0	0
0	0	0	0	0	0	0	0	0
0	1	0	0	0	0	0	0	0
0	0	0	0	0	0	0	0	0
0	0	0	0	0	0	0	0	0
0	0	0	0	0	0	0	0	0
0	0	0	0	0	0	0	0	0
0	0	0	0	0	0	0	0	0
0	0	0	0	0	0	0	0	0
0	0	0	0	0	0	0	0	0

0	0	0	0	0	0	0	0	0
0	0	0	0	0	0	0	0	0
0	0	0	0	0	0	0	0	0
0	0	0	0	0	0	0	0	0
0	0	0	0	0	0	0	0	0
0	0	0	0	0	0	0	0	0
0	1	0	0	0	0	0	0	0
0	0	0	0	0	0	0	0	0
0	1	0	0	0	0	0	0	0
0	0	0	0	0	0	0	0	0
0	1	0	0	0	0	0	0	0
0	0	0	0	0	0	0	0	0
0	0	0	0	0	0	0	0	0
0	0	0	0	0	0	0	0	0
0	1	0	0	0	0	0	0	0
0	1	0	0	0	0	0	0	0
0	0	0	0	0	0	0	0	0
0	1	0	0	0	0	0	0	0
0	1	0	0	0	0	0	0	0
0	0	0	0	0	0	0	0	0
0	0	0	0	0	0	0	0	0
0	0	0	0	0	0	0	0	0
0	0	0	0	0	1	0	0	1
0	1	0	0	0	1	0	0	0
0	0	0	0	0	0	0	0	0
0	1	0	0	0	0	0	0	0
0	1	0	0	0	0	0	0	0
0	1	0	0	0	0	0	0	0
0	1	0	0	0	0	0	0	0
0	0	0	0	0	0	0	0	0
0	0	0	0	0	0	0	0	0
1	1	0	0	0	0	0	0	0
0	0	0	0	0	0	0	0	0
0	0	0	0	0	0	0	0	0
0	0	0	0	0	0	0	0	0
0	0	0	0	0	0	0	0	0
0	0	0	0	0	0	0	0	0
0	0	0	0	0	0	0	0	0
0	0	0	0	0	0	0	0	0
0	0	0	0	0	0	0	0	0
0	0	0	0	0	0	0	0	0
0	0	0	0	0	0	0	0	0
1	0	0	0	0	0	1	0	0
0	0	0	0	0	0	0	0	0
1	0	0	0	0	0	0	0	0
0	0	0	0	0	0	0	0	0
0	0	0	0	0	0	0	0	0
0	0	0	0	0	0	0	0	0

0	0	0	0	0	0	0	0	0
0	0	0	0	0	0	0	0	0
0	0	0	0	0	0	0	0	0
0	0	0	0	0	0	0	0	0
0	0	0	0	0	0	0	0	0
0	0	0	0	0	0	0	0	0
0	0	0	0	0	0	0	0	0
0	0	0	0	0	0	0	0	0
0	0	0	0	0	0	0	0	0
0	0	0	0	0	0	0	0	0
0	1	0	0	0	0	0	0	0
0	0	0	0	0	0	0	0	0
0	0	0	0	0	0	0	0	0
0	0	0	0	0	0	0	0	0
0	1	0	0	0	0	0	0	0
0	0	0	0	0	0	0	0	0
0	0	0	0	0	0	0	0	0
0	0	0	0	0	0	0	0	0
0	1	0	0	0	0	0	0	0
0	1	0	0	0	0	0	0	0
0	0	0	0	0	0	0	0	0
0	0	0	0	0	0	0	0	0
0	1	0	0	0	0	0	0	0
0	1	0	0	0	0	0	0	0
0	1	0	0	0	0	0	0	0
0	1	0	0	0	0	0	0	0
0	1	0	0	0	0	0	0	0
0	1	0	0	0	0	0	0	0
0	1	0	0	0	0	0	0	0
1	0	0	0	0	0	0	0	0
0	0	0	0	0	0	0	0	0
0	0	0	0	0	0	0	0	0
0	1	0	0	0	0	0	0	0
0	0	0	0	0	0	0	0	0
0	0	0	0	0	0	0	0	0
0	0	0	0	0	0	0	0	0
0	0	0	0	0	0	0	0	0
0	0	0	0	0	0	0	0	0
0	1	0	0	1	0	0	0	0
0	0	0	0	0	0	0	0	0
0	1	0	0	0	0	0	0	0
0	1	1	1	0	0	0	0	0
0	0	0	0	0	1	0	0	0
0	1	0	0	0	0	0	0	0
0	1	0	0	0	0	0	0	0
0	0	0	0	0	0	0	0	0
0	1	0	0	0	0	0	0	0
0	1	0	0	0	0	0	0	0

0	1	0	0	0	0	0	0	0
0	1	0	0	0	0	0	0	0
0	0	0	0	0	0	0	0	0
0	0	0	0	0	0	0	0	0
0	1	0	0	0	0	0	0	1
0	0	0	0	0	1	0	0	0
0	1	0	0	0	0	0	0	0
0	1	0	0	0	0	0	0	0
0	1	0	0	0	0	0	0	0
0	0	0	0	0	1	0	0	0
0	0	0	0	0	0	0	0	0
0	1	0	0	0	0	0	0	0
0	0	0	0	0	0	0	0	0
0	1	0	0	0	0	0	0	0
0	0	0	0	0	0	0	0	0
0	0	0	0	0	0	0	0	0
0	0	0	0	0	0	0	0	0
0	0	0	0	0	0	0	0	0
0	0	0	0	0	0	0	0	0
0	1	0	0	0	0	0	0	0
0	1	0	0	0	0	1	0	1
0	1	0	0	0	0	0	0	0
0	1	0	0	0	0	0	0	0
0	0	0	0	0	0	0	0	0
0	0	0	0	0	0	0	0	0
0	1	0	0	0	0	0	0	0
0	0	0	0	0	0	0	0	0
0	0	0	0	0	0	0	0	0
0	0	0	0	0	0	0	0	0
0	0	0	0	0	0	0	0	0
0	0	0	0	0	0	0	0	0
1	0	0	0	0	0	0	0	0
0	0	0	0	0	0	0	0	0
0	0	0	0	0	0	0	0	0
0	0	0	0	0	0	0	0	0
0	0	0	0	0	0	0	0	0
0	0	0	0	0	0	0	0	0
0	0	0	0	0	0	0	0	0
0	0	0	0	0	0	0	0	0
0	0	0	0	0	0	0	0	0
1	1	0	0	0	0	0	0	0
0	0	0	0	0	0	0	0	0
0	0	0	0	0	0	0	0	0
0	0	0	0	0	0	0	0	0
0	0	0	0	0	0	0	0	0
0	0	0	0	0	0	0	0	0
0	1	0	0	0	0	0	0	0
0	0	0	0	0	0	0	0	0

0	0	0	0	0	0	0	0	0
0	0	0	0	0	0	0	0	0
0	0	0	0	0	0	0	0	0
0	0	0	0	0	0	0	0	0
0	0	0	0	0	0	0	0	0
0	0	0	0	0	0	0	0	0
0	0	0	0	0	0	0	0	0
0	1	0	0	0	1	0	0	0
0	1	0	0	0	0	0	0	0
0	0	0	0	0	1	0	0	0
0	0	0	0	0	0	0	0	0
0	0	0	0	0	0	0	0	0
0	1	0	0	0	0	0	0	0
0	0	0	0	0	0	0	0	0
0	0	0	0	0	0	0	0	0
0	0	0	0	0	0	0	0	0
0	0	0	0	0	0	0	0	0
0	0	0	0	0	0	0	0	0
0	0	0	0	0	0	0	0	0
0	1	0	0	0	0	0	0	0
0	0	0	0	0	0	0	0	0
0	0	0	0	0	0	0	0	0
0	0	0	0	0	0	0	0	0
0	0	0	0	0	0	0	0	0
0	1	0	0	0	0	0	0	0
0	0	0	0	0	0	0	0	0
0	0	0	0	0	0	0	0	0
0	0	0	0	0	0	0	0	0
0	0	0	0	0	0	0	0	0
0	1	0	0	0	0	0	0	0
0	0	0	0	0	0	0	0	0
0	0	0	0	0	0	0	0	0
0	0	0	0	0	0	0	0	0
0	0	0	0	0	0	0	0	0
0	0	0	0	0	0	0	0	0
0	0	0	0	0	0	0	0	0
0	0	0	0	0	0	0	0	0
0	0	0	0	0	0	0	0	0
0	0	0	0	0	0	0	0	0
0	0	0	0	0	0	0	0	0
0	1	0	0	0	0	0	0	0
0	0	0	0	0	0	0	0	0
0	1	0	0	0	0	0	0	0
0	1	0	0	0	0	0	0	0
0	0	0	0	0	0	0	0	0
0	0	0	0	0	0	0	0	0
0	0	0	0	0	0	0	0	0
0	1	0	0	0	0	0	0	0

0	0	0	0	0	0	0	0	0
0	0	0	0	0	0	0	0	0
0	1	0	0	0	0	0	0	0
0	1	0	0	0	0	0	0	0
0	1	0	0	0	0	0	0	0
0	0	0	0	0	0	0	0	0
0	0	0	0	0	0	0	0	0
0	0	0	0	0	0	0	0	0
0	0	0	0	0	0	0	0	0
0	0	0	0	0	0	0	0	0
0	0	0	0	0	0	0	0	0
1	1	0	0	0	0	0	0	0
0	1	0	0	0	0	0	0	0
0	0	0	0	0	0	0	0	0
0	1	0	0	0	0	0	0	0
0	1	0	0	0	0	0	0	0
0	0	0	0	0	0	0	0	0
0	0	0	0	0	0	0	0	0
0	0	0	0	0	0	0	0	0
0	0	0	0	0	0	0	0	0
0	0	0	0	0	0	0	0	0
0	0	0	0	0	0	0	0	0
0	1	0	0	0	0	0	0	0
0	0	0	0	0	0	0	0	0
0	1	0	0	0	0	0	0	0
0	1	0	0	0	0	0	0	0
0	1	0	0	0	0	0	0	0
0	0	0	0	0	0	0	0	0
0	0	0	0	0	0	0	0	0
0	0	0	0	0	0	0	0	0
0	0	0	0	0	0	0	0	0
0	0	0	0	0	0	0	0	0
0	1	0	0	0	0	0	0	0
0	0	0	0	0	0	0	0	0
0	1	0	0	0	0	0	0	0
0	1	0	0	0	0	0	0	0
0	0	0	0	0	0	0	0	0
0	0	0	0	0	0	0	0	0
0	0	0	0	0	0	0	0	0
0	1	0	0	0	0	0	0	0
0	0	0	0	0	0	0	0	0
0	1	0	0	0	0	0	0	0
0	0	0	0	0	0	0	0	0
0	0	0	0	0	0	0	0	0
0	0	0	0	0	0	0	0	0
0	1	0	0	0	0	0	0	0
0	0	0	0	0	0	0	0	0
0	0	0	0	0	0	0	0	0

0	1	0	0	0	0	0	0	0
0	0	0	0	0	0	0	0	0
0	0	0	0	0	0	0	0	0
0	0	0	0	0	0	0	0	0
0	0	0	0	0	0	0	0	0
0	0	0	0	0	0	0	0	0
0	0	0	0	0	0	0	0	0
0	0	0	0	0	0	0	0	0
0	0	0	0	0	0	0	0	0
0	0	0	0	0	0	0	0	0
0	0	0	0	0	0	0	0	0
0	0	0	0	0	0	0	0	0
0	0	0	0	0	0	0	0	0
0	0	0	0	0	0	0	0	0
0	0	0	0	0	0	0	0	0
0	0	0	0	0	0	0	0	0
0	0	0	0	0	0	0	0	0
0	0	0	0	0	0	0	0	0
0	0	0	0	0	0	0	0	0
0	0	0	0	0	0	0	0	0
0	0	0	0	0	0	0	0	0
0	0	0	0	0	0	0	0	0
0	0	0	0	0	0	0	0	0
0	0	0	0	0	0	0	0	0
0	0	0	0	0	0	0	0	0
0	0	0	0	0	0	0	0	0
0	0	0	0	0	0	0	0	0
0	0	0	0	0	0	0	0	0
0	0	0	0	0	0	0	0	0
0	0	0	0	0	0	0	0	0
0	0	0	0	0	0	0	0	0
0	0	0	0	0	0	0	0	0
0	0	0	0	0	0	0	0	0
0	0	0	0	0	0	0	0	0
0	0	0	0	0	0	0	0	0
0	0	0	0	0	0	0	0	0
0	0	0	0	0	0	0	0	0
0	0	0	0	0	0	0	0	0
0	1	0	0	0	0	0	0	0
0	0	0	0	0	0	0	0	0
0	0	0	0	0	0	0	0	0
0	0	0	0	0	0	0	0	0
0	0	0	0	0	0	0	0	0
0	0	0	0	0	0	0	0	0
0	0	0	0	0	0	0	0	0
0	0	0	0	0	0	0	0	0
0	0	0	0	0	0	0	0	0
0	0	0	0	0	0	0	0	0

0	0	0	0	0	0	0	0	0
0	0	0	0	0	0	0	0	0
0	0	0	0	0	0	0	0	0
1	0	0	0	0	0	0	0	0
0	0	0	0	0	0	0	0	0
0	0	0	0	0	0	0	0	0
0	0	0	0	0	0	0	0	0
0	0	0	0	0	0	0	0	0
1	1	0	0	0	0	0	0	0
0	0	0	0	0	0	0	0	0
0	0	0	0	0	0	0	0	0
0	0	0	0	0	0	0	0	0
0	0	0	0	0	0	0	0	0
0	0	0	0	0	0	0	0	0
0	0	0	0	0	0	0	0	0
0	0	0	0	0	0	0	0	0
0	0	0	0	0	0	0	0	0
0	0	0	0	0	0	0	0	0
0	0	0	0	0	0	0	0	0
0	0	0	0	0	0	0	0	0
0	0	0	0	0	0	0	0	0
1	0	0	0	0	0	0	0	0
0	0	0	0	0	0	0	0	0
0	0	0	0	0	0	0	0	0
0	0	0	0	0	0	0	0	0
0	0	0	0	0	0	0	0	0
0	0	0	0	0	0	0	0	0
0	0	0	0	0	0	0	0	0
0	0	0	0	0	0	0	0	0
0	0	0	0	0	0	0	0	0
0	0	0	0	0	0	0	0	0
1	0	0	0	0	0	0	0	0
0	0	0	0	0	0	0	0	0
0	0	0	0	0	0	0	0	0
0	0	0	0	0	0	0	0	0
0	0	0	0	0	0	0	0	0
0	0	0	0	0	0	0	0	0
0	0	0	0	0	0	0	0	0
0	0	0	0	0	0	0	0	0
0	0	0	0	0	0	0	0	0
0	0	0	0	0	0	0	0	0
0	0	0	0	0	0	0	0	0
0	0	0	0	0	0	0	0	0
1	0	0	0	0	0	0	0	0
0	0	0	0	0	0	0	0	0
0	0	0	0	0	0	0	0	0
0	0	0	0	0	0	0	0	0
0	0	0	0	0	0	0	0	0

0	0	0	0	0	0	0	0	0
1	0	0	0	0	0	0	0	0
0	0	0	0	0	0	0	0	0
0	0	0	0	0	0	0	0	0
0	0	0	0	0	0	0	0	0
0	0	0	0	0	0	0	0	0
0	0	0	0	0	0	0	0	0
0	0	0	0	0	0	0	0	0
0	0	0	0	0	0	0	0	0
0	0	0	0	0	0	0	0	0
0	0	0	0	0	0	0	0	0
0	0	0	0	0	0	0	0	0
0	0	0	0	0	0	0	0	0
0	0	0	0	0	0	0	0	0
0	0	0	0	0	0	0	0	0
0	0	0	0	0	0	0	0	0
0	0	0	0	0	0	0	0	0
0	0	0	0	0	0	0	0	0
0	0	0	0	0	0	0	0	0
0	0	0	0	0	0	0	0	0
0	0	0	0	0	0	0	0	0
0	0	0	0	0	0	0	0	0
0	0	0	0	0	0	0	0	0
0	0	0	0	0	0	0	0	0
0	0	0	0	0	0	0	0	0
0	0	0	0	0	0	0	0	0
0	0	0	0	0	0	0	0	0
0	0	0	0	0	0	0	0	0
0	0	0	0	0	0	0	0	0
0	0	0	0	0	0	0	0	0
0	0	0	0	0	0	0	0	0
0	0	0	0	0	0	0	0	0
0	0	0	0	0	0	0	0	0
0	0	0	0	0	0	0	0	0
0	0	0	0	0	0	0	0	0
0	0	0	0	0	0	0	0	0
0	0	0	0	0	0	0	0	0
0	0	0	0	0	0	0	0	0
0	0	0	0	0	0	0	0	0
0	0	0	0	0	0	0	0	0
0	0	0	0	0	0	0	0	0
0	0	0	0	0	0	0	0	0
0	0	0	0	0	0	0	0	0
0	1	0	0	0	0	0	0	0
0	0	0	0	0	0	0	0	0
0	0	0	0	0	0	0	0	0
0	0	0	0	0	0	0	0	0
0	0	0	0	0	0	0	0	0

0	0	0	0	0	0	0	0	0
0	0	0	0	0	0	0	0	0
0	0	0	0	0	0	0	0	0
0	0	0	0	0	0	0	0	0
0	0	0	0	0	0	0	0	0
0	0	0	0	0	0	0	0	0
0	0	0	0	0	0	0	0	0
0	0	0	0	0	0	0	0	0
0	0	0	0	0	0	0	0	0
0	0	0	0	0	0	0	0	0
0	0	0	0	0	0	0	0	0
0	0	0	0	0	0	0	0	0
0	0	0	0	0	0	0	0	0
0	0	0	0	0	0	0	0	0
0	0	0	0	0	0	0	0	0
0	0	0	0	0	0	0	0	0
0	0	0	0	0	0	0	0	0
0	0	0	0	0	0	0	0	0
0	0	0	0	0	0	0	0	0
0	0	0	0	0	0	0	0	0
0	0	0	0	0	0	0	0	0
0	1	0	0	0	0	0	0	0
0	1	0	0	0	0	0	0	0
0	1	0	0	0	0	0	0	0
0	0	0	0	0	0	0	0	0
0	1	0	0	0	0	0	0	0
0	1	0	0	0	0	0	0	0
0	0	0	0	0	0	0	0	0
0	0	0	0	0	0	0	0	0
0	1	0	0	0	0	0	0	0
0	1	0	0	0	0	0	0	0
0	1	0	0	0	0	0	0	0
0	1	0	0	0	0	0	0	0
0	0	0	0	0	0	0	0	0
0	0	0	0	0	0	0	0	0
0	0	0	0	0	0	0	0	0
0	0	0	0	0	0	0	0	0
0	1	0	0	0	0	0	0	0
0	0	0	0	0	0	0	0	0
0	0	0	0	0	0	0	0	0
0	0	0	0	0	0	0	0	0
0	0	0	0	0	0	0	0	0
0	0	0	0	0	0	0	0	0
0	0	0	0	0	0	0	0	0
0	0	0	0	0	0	0	0	0
0	0	0	0	0	0	0	0	0
0	0	0	0	0	0	0	0	0
0	1	0	0	0	0	0	0	0

0	1	0	0	0	0	0	0	0
0	0	0	0	0	0	0	0	0
0	1	0	0	0	0	0	0	0
0	1	0	0	0	0	0	0	0
0	0	0	0	0	0	0	0	0
0	0	0	0	0	1	0	0	0
0	0	0	0	0	0	0	0	0
0	1	0	0	0	0	0	0	0
0	0	0	0	0	0	0	0	0
0	1	0	0	0	0	0	0	0
0	1	0	0	0	0	1	0	0
0	1	0	0	0	0	0	0	0
0	0	0	0	0	0	0	0	0
0	1	0	0	0	0	0	0	0
0	1	0	0	0	0	0	0	0
0	0	0	0	0	0	0	0	0
0	1	0	0	0	0	0	0	0
0	1	0	0	0	0	0	0	0
1	1	0	0	0	0	0	0	0
0	1	0	0	0	0	0	0	0
0	0	0	0	0	0	0	0	0
0	1	0	0	0	0	0	0	0
0	1	0	0	0	0	0	0	0
0	1	0	0	0	0	0	0	0
0	0	0	0	0	0	0	0	0
0	1	0	0	1	0	0	0	0
0	1	0	0	0	0	0	0	0
0	0	0	0	0	0	0	0	0
0	1	0	0	0	0	0	0	0
0	1	0	0	0	0	0	0	0
0	1	0	0	0	0	0	0	0
0	1	0	0	0	0	0	0	0
0	1	0	0	0	0	0	0	0
0	1	0	0	0	0	0	0	0
0	1	0	0	0	0	0	0	0
0	1	0	0	0	0	0	0	0
0	1	0	0	0	0	0	0	0
0	0	0	0	0	0	0	0	0
0	1	0	0	0	0	0	0	0
0	1	0	0	0	0	0	0	0
1	0	0	0	0	0	0	0	0
0	0	0	0	0	0	0	0	0
0	0	0	0	0	0	0	0	0
0	0	0	0	0	0	0	0	0
0	0	0	0	0	0	0	0	0
0	0	0	0	0	0	0	0	0
0	1	0	0	0	0	0	0	0
0	0	0	0	0	0	0	0	0

0	0	0	0	0	0	0	0	0
0	0	0	0	0	0	0	0	0
0	0	0	0	0	0	0	0	0
0	1	0	0	0	0	0	0	0
0	1	0	0	0	0	0	0	0
0	1	0	0	0	0	0	0	0
0	0	0	0	0	0	0	0	0
0	1	0	0	0	0	0	0	0
0	1	0	0	0	0	0	0	0
0	0	0	0	0	0	0	0	0
0	1	0	0	0	0	0	0	0
0	1	0	0	0	0	0	0	0
0	0	0	0	0	0	0	0	0
0	1	0	0	0	0	0	0	0
0	0	0	0	0	0	0	0	0
0	1	0	0	0	0	0	0	0
0	1	0	0	0	0	0	0	0
0	1	0	0	0	0	0	0	0
0	1	0	0	0	0	0	0	0
0	1	0	0	0	0	0	0	0
0	1	0	0	0	0	0	0	0
0	0	0	0	0	0	0	0	0
0	1	0	0	0	0	0	0	0
0	0	0	0	0	0	0	0	0
0	0	0	0	0	0	0	0	0
0	0	0	0	0	0	0	0	0
0	0	0	0	0	0	0	0	0
1	0	0	0	0	0	0	0	0
0	1	0	0	0	0	0	0	0
0	0	0	0	0	1	0	0	0
0	1	0	0	0	0	0	0	0
0	1	0	0	0	0	0	0	0
0	1	0	0	0	0	0	0	0

MOF	MODS	BI	AIS	Diabetes	Hypertension	Osteoporosis	Coma
0	0	0	2	0	0	0	0
0	0	0	4	0	0	0	0
0	0	0	3	0	0	0	0
0	0	0	3	0	1	0	0
0	0	0	2	0	0	0	0
0	0	0	2	0	0	0	0
0	0	0	2	0	1	0	1
0	0	0	3	0	0	0	0
0	0	0	2	0	0	0	0
0	0	0	3	0	0	0	1
0	0	0	3	0	0	0	0
0	0	0	2	0	0	0	0
0	0	0	3	0	0	0	1
0	0	0	3	0	0	0	0
0	0	0	2	0	0	0	0
0	0	0	2	0	0	0	0
0	0	0	2	0	0	0	1
0	0	0	2	0	0	0	0
0	0	0	2	0	0	0	0
0	0	0	2	0	0	0	0
0	0	0	2	0	0	0	0
0	0	0	3	0	0	0	0
0	0	0	3	0	1	0	0
0	0	0	2	0	0	0	0
0	0	0	3	0	0	0	0
0	0	0	2	0	0	0	0
0	0	0	2	0	0	0	0
0	0	0	2	0	0	0	0
0	0	0	2	0	0	0	0
0	0	0	2	0	0	0	0
0	0	0	2	0	0	0	0
0	0	0	2	0	0	0	0
0	0	0	2	0	0	0	0
0	0	0	2	0	0	0	0
0	0	0	3	0	0	0	0
0	0	0	3	0	0	0	0
0	0	0	2	0	0	0	0
0	0	0	3	0	0	0	0
0	0	0	3	0	0	0	1
0	0	0	3	0	0	0	0
0	0	0	2	0	1	0	1
0	0	0	3	0	0	0	1
0	0	0	3	0	0	0	1
0	0	0	2	0	0	0	0
0	0	0	3	0	0	0	0
0	0	0	3	0	0	0	1
0	0	0	3	0	0	0	0

0	0	0	2	0	0	0	1
0	0	0	2	0	0	0	0
0	0	0	3	0	1	0	0
0	0	0	3	0	0	0	1
0	0	0	1	0	0	0	1
0	0	0	2	0	0	0	0
0	0	1	3	0	0	0	0
0	0	0	5	0	0	0	0
0	0	0	5	0	0	0	1
0	0	0	3	0	0	0	0
0	0	0	2	0	0	0	0
0	0	0	3	0	0	0	0
0	0	0	3	0	0	0	1
0	0	0	3	0	0	0	1
0	0	0	2	0	0	0	0
0	0	0	3	0	0	0	0
0	0	0	2	0	0	0	0
0	0	0	2	0	0	0	0
0	0	0	2	0	0	0	0
0	0	0	3	0	0	0	1
0	0	0	3	0	0	0	1
0	0	0	2	0	0	0	1
0	0	0	3	0	0	0	0
0	0	0	2	0	0	0	0
0	0	0	2	0	0	0	0
0	0	0	3	0	0	0	0
0	0	0	2	0	0	0	0
0	0	0	2	0	0	0	0
0	0	0	3	0	0	0	0
0	0	0	5	0	0	0	1
0	0	0	2	0	0	0	1
0	0	0	3	0	0	0	1
0	0	0	3	0	0	0	0
0	0	0	3	0	1	0	0
0	0	0	3	0	0	0	0
0	0	0	2	0	0	0	0
0	0	0	2	0	1	0	0
0	0	0	3	0	0	0	0
0	0	0	3	0	0	0	0
0	0	0	2	0	0	0	0
0	0	0	5	0	0	0	0
0	0	0	3	1	0	0	1
0	0	0	3	0	0	0	1
0	0	0	3	0	0	0	0
0	0	0	2	1	0	0	0
0	0	0	2	0	0	0	0
0	0	0	3	0	0	0	0
0	0	0	2	0	0	0	0

0	0	0	2	0	0	0	0
0	0	0	1	0	0	0	0
0	0	0	2	1	0	0	0
0	0	0	3	1	1	0	0
0	0	0	3	0	0	0	0
0	0	0	2	0	0	0	0
0	0	0	3	0	0	0	0
0	0	0	3	0	0	0	0
0	0	0	3	0	0	0	0
0	0	0	2	1	0	0	0
0	0	0	2	0	0	0	0
0	0	0	3	0	0	0	0
0	0	0	2	0	0	0	0
0	0	0	2	0	0	0	0
0	0	0	2	0	0	0	0
0	0	0	2	0	0	0	0
0	0	0	2	0	0	0	0
0	0	0	2	0	0	0	0
0	0	0	3	0	0	0	0
0	0	0	2	0	0	0	0
0	0	0	3	0	0	0	0
0	0	0	3	1	1	0	0
0	0	0	2	0	0	0	0
0	0	0	3	0	1	0	0
0	0	0	3	0	0	0	0
0	0	0	3	0	0	0	0
0	0	0	2	0	0	0	0
0	0	0	3	0	0	0	0
0	0	0	2	0	0	0	0
0	0	0	3	0	0	0	0
0	0	0	3	0	1	0	1
0	0	1	5	0	0	0	1
0	0	0	3	0	1	0	0
0	0	0	2	0	0	0	0
0	0	0	2	0	0	0	0
0	0	0	2	0	0	0	0
0	0	0	2	0	0	0	0
0	0	0	2	0	0	0	0
0	0	0	2	0	0	0	0
0	0	0	2	0	0	0	0
0	0	0	2	0	0	0	0
0	0	0	2	0	0	0	0
0	0	0	3	1	0	0	0
0	0	0	3	0	1	0	0
0	0	0	2	0	0	0	0
0	0	0	1	0	0	0	0
0	0	0	2	0	1	0	0
0	1	0	5	1	1	0	0

0	0	0	3	0	0	0	0
0	0	0	3	0	0	0	0
0	0	0	2	0	0	0	0
0	0	0	2	0	0	0	0
0	0	0	2	0	0	0	0
0	0	0	3	0	0	0	1
1	0	0	4	0	1	0	0
0	0	0	2	0	0	0	0
0	0	0	2	0	0	0	0
0	0	0	3	0	0	0	0
0	0	0	2	0	0	0	0
0	0	0	3	0	0	0	0
0	0	0	2	0	0	0	0
0	0	0	2	0	0	0	0
0	0	0	2	0	0	0	0
0	0	0	3	0	0	0	0
0	0	0	3	0	0	0	0
0	0	0	3	0	0	0	0
0	0	0	3	0	0	0	0
0	0	0	2	0	0	0	0
0	0	0	3	0	1	0	0
0	0	0	2	0	0	0	0
0	0	0	3	0	0	0	1
0	0	0	3	0	0	0	1
0	0	0	4	0	0	0	0
0	0	0	2	0	0	0	0
0	0	0	3	0	0	0	1
0	0	0	2	0	0	0	0
0	0	0	2	0	0	0	0
0	0	0	2	0	0	0	0
0	0	0	2	1	0	0	0
0	0	0	2	0	0	0	0
0	0	0	3	0	0	0	0
0	0	0	2	0	0	0	0
0	0	0	2	0	0	0	0
0	0	0	2	0	0	0	0
0	0	0	2	0	0	0	0
0	0	0	3	0	0	0	0
0	0	0	3	0	0	0	0
0	0	0	1	0	1	0	0
0	0	0	3	0	0	0	0
0	0	0	2	0	1	0	0
0	0	0	3	0	0	0	0
0	0	0	2	0	0	0	0
0	0	0	1	0	0	0	0
0	0	0	2	0	0	0	0
0	0	0	3	0	0	0	0
0	0	0	2	0	0	0	0

0	0	0	3	0	0	0	0
0	0	0	3	0	0	0	0
0	0	0	3	0	0	0	0
0	0	0	1	0	0	0	0
0	0	1	3	0	0	0	0
0	0	0	2	0	0	0	0
0	0	0	3	0	0	0	0
0	0	0	3	0	0	0	0
0	0	0	3	0	0	0	0
0	0	0	3	0	0	0	0
0	0	0	3	0	0	0	0
0	0	0	2	0	0	0	0
0	0	0	2	0	0	0	0
0	0	0	3	0	0	0	0
0	0	0	3	0	0	0	0
0	0	0	2	0	0	0	0
0	0	0	3	0	0	0	0
0	0	0	2	0	0	0	0
0	0	0	3	0	0	0	0
0	0	0	2	0	1	0	0
0	0	0	2	0	0	0	0
0	0	1	3	0	0	0	1
0	0	0	2	0	0	0	0
0	0	0	3	0	0	0	0
0	0	0	3	0	0	0	0
0	0	0	2	0	0	0	0
0	0	0	2	0	0	0	0
0	0	0	1	0	0	0	0
0	0	0	2	0	0	0	0
0	0	0	2	0	0	0	0
0	0	0	2	0	0	0	0
0	0	0	3	0	0	0	1
0	0	0	1	0	0	0	0
0	0	0	3	0	0	0	1
0	0	0	3	0	0	0	1
0	0	0	2	0	0	0	0
0	0	1	3	1	1	0	1
0	0	0	3	0	0	0	0
0	0	0	3	0	0	0	0
0	0	0	3	0	0	0	0
0	0	0	1	0	0	0	0
0	0	0	1	0	0	0	0
0	0	0	2	0	0	0	0
0	0	0	2	1	1	0	0
0	0	0	2	0	0	0	0
0	0	0	3	0	1	0	0
0	0	0	3	0	0	0	0
0	0	0	2	0	0	0	0

0	0	0	3	0	0	0	0
0	0	0	3	0	0	0	0
0	0	0	2	0	0	0	0
0	0	0	3	0	0	0	0
0	0	0	3	0	0	0	0
0	0	0	3	0	0	0	0
0	0	0	3	0	0	0	0
0	0	0	3	0	0	0	0
0	0	0	2	0	0	0	0
0	0	0	2	0	0	0	0
0	0	0	2	0	1	0	0
0	0	0	2	0	0	0	0
0	0	0	3	0	0	0	0
0	0	0	2	0	0	0	0
0	0	0	2	0	0	0	0
0	0	0	2	0	0	0	0
0	0	0	2	0	0	0	0
0	0	0	3	0	0	0	0
0	0	0	2	0	0	0	0
0	0	0	2	0	0	0	0
0	0	0	3	0	0	0	0
0	0	0	3	0	0	0	0
0	0	0	2	0	0	0	0
0	0	0	1	0	1	0	0
0	0	0	2	0	0	0	0
0	0	0	2	0	0	0	0
0	0	0	2	0	0	0	0
0	0	0	3	0	0	0	0
0	0	0	2	0	0	0	0
0	0	0	1	0	0	0	0
0	0	0	3	0	0	0	0
0	0	0	3	0	0	0	0
0	0	0	2	1	1	0	0
0	0	0	2	0	0	0	0
0	0	0	3	0	0	0	0
0	0	0	2	1	0	0	0
0	0	0	2	0	0	0	0
0	0	0	1	0	0	0	0
0	0	1	3	0	0	0	0
0	0	1	4	0	0	0	0
0	0	0	2	0	0	0	0
0	0	0	2	0	0	0	0
0	0	0	3	0	0	0	1
0	0	0	3	0	0	0	0
0	1	1	3	1	1	0	1
0	0	0	3	0	0	0	0
0	0	0	4	0	0	0	0
0	0	0	2	0	0	0	0

0	0	0	3	0	0	0	0
0	0	0	1	0	0	0	0
0	0	0	3	0	0	0	0
0	0	0	3	0	0	0	0
0	0	0	2	0	0	0	0
0	0	0	2	1	1	0	0
0	0	0	3	0	0	0	0
0	0	0	3	1	1	0	0
0	0	0	3	0	0	0	0
0	0	0	3	0	0	0	0
0	0	0	2	0	1	0	0
0	0	1	5	0	0	0	1
0	0	0	1	0	0	0	0
0	0	0	3	1	1	0	0
0	0	0	3	1	0	0	0
0	0	0	3	0	0	0	1
0	0	0	2	1	0	0	0
0	0	0	2	0	0	0	1
0	0	0	1	0	0	0	0
0	0	0	3	0	0	0	1
0	0	0	2	0	1	0	0
0	0	0	3	0	0	0	0
0	0	0	2	0	0	0	0
0	0	0	3	0	0	0	0
0	0	1	2	0	0	0	0
0	0	0	2	0	0	0	0
0	0	0	1	0	0	0	0
0	0	0	3	0	0	0	0
0	0	0	3	0	0	0	0
0	0	0	3	0	0	0	0
0	0	0	3	0	0	0	1
0	0	0	2	0	0	0	0
0	0	1	5	0	0	0	1
0	0	0	3	0	0	0	0
0	0	0	2	0	0	0	0
0	0	0	1	0	0	0	0
0	0	0	3	0	0	0	1
0	0	0	3	0	0	0	0
0	0	1	3	0	0	0	1
0	0	0	2	0	0	0	0
0	0	0	2	0	0	0	0
0	0	0	2	0	0	0	0
0	0	0	2	0	0	0	0
0	0	0	3	0	0	0	0
0	0	0	3	0	0	0	0
0	0	0	2	0	0	0	0
0	0	0	3	1	0	0	0
0	0	0	3	0	1	0	0

0	0	0	3	0	0	0	0
0	0	0	2	0	0	0	0
0	0	0	3	0	0	0	0
0	0	0	2	0	0	0	0
0	0	0	2	0	0	0	0
0	0	0	2	0	0	0	0
0	0	0	2	0	0	0	0
0	0	0	2	0	0	0	0
0	0	0	2	0	0	0	0
0	0	0	3	0	0	0	0
0	0	0	2	0	0	0	0
0	0	0	3	0	0	0	0
0	0	0	2	0	0	0	0
0	0	0	2	0	0	0	0
0	0	0	2	1	0	0	0
0	0	0	2	0	0	0	0
0	0	0	3	0	0	0	0
0	0	0	3	0	0	0	0
0	0	0	2	0	0	0	0
0	0	0	2	0	0	0	0
0	0	0	2	0	0	0	0
0	0	0	2	0	0	0	0
0	0	0	2	0	0	0	0
0	0	0	3	0	0	0	0
0	0	0	3	0	0	0	0
0	0	0	2	0	0	0	0
0	0	0	2	0	0	0	0
0	0	0	2	0	0	0	0
0	0	0	2	0	1	0	0
0	0	0	2	0	0	0	0
0	0	0	2	0	0	0	0
0	0	0	2	0	0	0	0
0	0	0	2	0	0	0	0
0	0	0	3	0	0	0	0
0	0	0	3	0	0	0	0
0	0	0	2	0	0	0	0
0	0	0	3	1	0	0	0
0	0	0	2	0	1	0	0
0	0	0	2	1	0	0	0
0	0	0	3	0	0	0	0
0	0	0	3	0	0	0	0
0	0	0	3	1	0	0	0
0	1	0	5	0	0	0	0
0	0	0	3	0	1	0	0
0	0	0	2	0	0	0	0
0	0	0	3	0	0	0	0
0	0	0	2	0	0	0	1
0	0	0	3	0	0	0	0

0	0	0	4	0	0	0	0
0	0	0	3	0	0	0	1
0	0	1	3	0	0	0	0
0	0	0	3	0	0	0	0
0	0	0	2	0	0	0	0
0	0	0	3	0	0	0	0
0	0	0	5	0	0	0	0
0	0	0	2	0	0	0	0
0	0	0	2	0	0	0	0
0	0	0	3	0	0	0	1
0	0	0	2	0	0	0	0
0	0	0	3	0	1	0	0
0	0	0	3	0	0	0	0
0	0	0	3	1	0	0	0
0	0	0	3	0	0	0	0
0	0	1	5	0	0	0	1
0	0	1	3	0	1	0	0
0	0	0	3	0	0	0	1
0	0	0	2	0	0	0	0
0	0	0	3	0	0	0	0
0	0	0	3	0	0	0	0
0	0	0	2	0	0	0	0
0	0	0	2	0	0	0	0
0	0	0	2	0	0	0	0
0	0	0	4	0	0	0	0
0	0	0	2	0	0	0	0
0	0	0	2	0	0	0	0
0	0	0	2	0	0	0	0
0	0	0	2	0	0	0	0
0	0	0	2	1	0	0	0
0	0	0	2	0	0	0	0
0	0	0	2	0	0	0	0
0	0	0	4	0	0	0	0
0	0	0	2	0	0	0	0
0	0	0	2	0	1	0	0
0	0	0	3	0	0	0	0
0	0	0	3	0	0	0	0
0	0	0	3	0	0	0	0
0	0	0	2	0	1	0	0
0	0	0	2	0	0	0	0
0	0	0	2	0	0	0	0
0	0	0	2	1	1	0	0
0	0	0	3	0	0	0	0
0	0	0	2	0	0	0	0
0	0	1	5	0	0	0	1
0	0	0	3	0	0	0	0
0	0	0	2	0	1	0	0
0	0	0	2	0	0	0	0

0	0	0	1	1	1	0	0
0	0	0	3	0	0	0	1
0	0	0	3	0	0	0	0
0	0	0	3	0	0	0	0
0	0	0	2	1	1	0	0
0	0	0	2	0	0	0	0
0	0	0	2	1	1	0	0
0	0	0	2	1	0	0	0
0	0	0	2	0	0	0	0
0	0	0	2	0	0	0	0
0	0	0	3	0	0	0	0
0	0	0	3	0	1	0	0
0	0	1	3	0	1	0	1
0	0	0	2	0	0	0	0
0	0	0	3	0	0	0	0
0	0	0	3	0	0	0	1
0	0	0	2	0	0	0	0
0	0	0	3	0	0	0	0
0	0	0	2	0	0	0	0
0	0	0	3	0	0	0	0
0	0	0	3	0	0	0	0
0	0	0	2	0	0	0	0
0	0	0	2	0	0	0	0
0	0	1	3	1	1	0	0
0	0	0	2	0	0	0	0
0	0	0	3	0	0	0	1
0	0	0	1	0	0	0	0
0	0	0	3	0	0	0	0
0	0	0	3	0	0	0	1
0	0	0	3	0	0	0	0
0	0	0	3	1	0	0	0
0	0	0	2	0	0	0	0
0	0	0	2	0	0	0	0
0	0	0	3	0	0	0	0
0	0	0	2	1	1	0	0
0	0	0	2	0	0	0	0
0	0	0	2	0	0	0	0
0	0	0	2	0	0	0	0
0	0	0	2	0	0	0	0
0	0	0	2	0	0	0	0
0	0	0	2	0	0	0	0
0	0	0	1	1	0	0	0
0	0	0	1	0	0	0	0
0	0	0	2	0	0	0	0
0	0	1	1	1	1	0	0
0	0	0	3	0	0	0	0
0	0	0	2	0	0	0	0
0	0	0	2	0	0	0	0

0	0	0	1	0	0	0	0
0	0	0	1	1	1	0	0
0	0	0	3	0	1	0	0
0	0	0	2	0	0	0	0
0	0	0	2	0	0	0	0
0	0	0	3	0	1	0	0
0	0	0	1	0	0	0	0
0	0	0	2	0	0	0	0
0	0	0	2	0	0	0	0
0	0	0	2	0	0	0	0
0	0	0	2	0	0	0	0
0	0	0	3	0	0	0	0
0	0	0	3	0	0	0	0
0	0	0	2	0	1	0	0
0	0	0	2	0	0	0	0
0	0	0	2	0	0	0	1
0	0	0	1	0	0	0	0
0	0	0	2	0	0	0	0
0	0	0	2	0	0	0	0
0	0	0	2	0	0	0	0
0	0	0	2	0	0	0	0
0	0	0	4	0	0	0	0
0	0	0	3	1	0	0	0
0	0	0	2	0	1	0	0
0	0	0	2	0	0	0	0
0	0	0	2	0	0	0	0
0	0	0	2	0	0	0	0
0	0	0	2	0	0	0	0
0	0	0	2	0	0	0	0
0	0	0	2	0	0	0	0
0	0	0	2	0	0	0	0
0	0	0	2	0	0	0	0
0	0	0	3	1	0	0	0
0	0	0	2	0	0	0	0
0	0	0	3	0	0	0	0
0	0	0	3	0	0	0	0
0	0	0	2	0	0	0	0
0	0	0	3	0	0	0	0
0	0	0	2	0	0	0	0
0	0	0	3	0	0	0	1
0	0	0	3	0	0	0	1
0	0	0	2	0	1	0	0
0	0	0	2	0	0	0	0
0	0	0	3	0	0	0	1
0	0	0	2	0	0	0	0
0	0	0	2	0	0	0	0
0	0	0	2	0	0	0	0
0	0	0	2	0	0	0	0

0	0	0	3	1	1	0	0
0	0	0	3	0	0	0	0
0	0	0	2	0	0	0	1
0	0	0	2	0	0	0	0
0	0	0	2	0	0	0	0
0	0	0	2	0	0	0	0
0	0	0	4	0	0	0	0
0	0	0	2	0	0	0	0
0	0	0	1	1	0	0	0
0	0	0	3	0	0	0	0
0	0	0	3	0	0	0	0
0	0	0	3	0	0	0	0
0	0	0	2	0	0	0	0
0	0	0	2	0	0	0	0
0	0	0	2	0	0	0	0
0	0	0	2	0	1	0	0
0	0	0	3	0	0	0	0
0	0	0	2	0	0	0	0
0	0	0	2	0	0	0	0
0	0	0	2	0	1	0	0
0	0	1	3	0	0	0	1
0	0	0	3	0	0	0	0
0	0	0	2	0	0	0	0
0	0	0	3	0	0	0	1
0	0	0	3	0	0	0	0
0	0	0	3	0	0	0	1
0	0	0	2	0	0	0	1
0	0	0	2	0	0	0	0
0	0	0	2	0	0	0	0
0	0	0	3	1	1	0	1
0	0	1	5	0	0	0	1
0	0	0	3	0	1	0	1
0	0	0	3	0	0	0	1
0	0	0	3	0	0	0	1
0	0	0	3	0	0	0	1
0	0	0	3	0	0	0	1
0	0	0	4	1	0	0	1
0	0	0	1	0	1	0	0
0	0	1	3	0	0	0	0
0	0	0	3	0	0	0	0
0	0	0	3	0	0	0	0
0	0	0	3	0	0	0	0
0	0	0	2	0	0	0	0
0	0	0	3	0	0	0	1
0	0	0	2	0	0	0	0
0	0	0	2	1	1	0	1
0	0	0	3	0	0	0	0
0	0	0	3	0	0	0	0

0	0	0	3	0	0	0	0
0	0	0	2	0	0	0	0
0	0	0	3	1	0	0	0
0	0	0	3	0	0	0	0
0	0	0	3	0	0	0	0
0	0	0	3	0	0	0	0
0	0	0	3	0	0	0	0
0	0	0	3	0	1	0	1
0	0	0	3	0	0	0	0
0	0	0	2	0	0	0	0
0	0	0	3	0	0	0	0
0	0	0	2	0	0	0	0
0	0	0	3	0	0	0	0
0	0	0	1	0	0	0	0
0	0	0	2	0	1	0	0
0	0	0	1	0	0	0	0
0	0	0	3	0	0	0	0
0	0	0	3	0	0	0	0
0	0	0	3	0	1	0	0
0	0	0	3	0	0	0	1
0	0	0	3	0	1	0	0
0	0	0	2	0	0	0	0
0	0	0	4	0	1	0	0
0	0	0	2	0	1	0	0
0	0	0	3	0	0	0	0
0	0	0	4	0	1	0	0
0	0	0	3	0	0	0	0
0	0	0	5	0	0	0	1
0	0	0	3	0	0	0	0
0	0	0	2	1	1	0	0
0	0	0	2	0	0	0	0
0	0	0	3	0	0	0	0
0	0	0	3	0	0	0	0
0	0	0	2	0	1	0	0
0	0	0	3	0	0	0	0
0	0	0	2	0	0	0	0
0	0	0	2	0	0	0	0
0	0	0	2	0	0	0	0
0	0	0	2	0	0	0	0
0	0	0	3	0	0	0	0
0	0	0	3	0	0	0	0
0	0	0	3	0	1	0	0
0	0	0	2	0	1	0	0
0	0	0	3	0	0	0	0
0	0	0	3	0	0	0	0
0	0	0	3	0	0	0	0
0	0	0	2	0	0	0	0
0	0	0	1	0	0	0	0

0	0	0	5	0	0	0	1
0	0	0	2	0	0	0	0
0	0	0	1	0	0	0	0
0	0	1	3	0	0	0	1
0	0	1	3	1	0	0	1
0	0	0	3	1	0	0	0
0	0	0	3	0	0	0	0
0	0	0	3	0	1	0	0
0	0	0	1	0	0	0	0
0	0	0	3	0	0	0	0
0	0	0	3	0	1	0	0
0	0	0	3	0	0	0	0
0	0	0	3	0	0	0	0
0	0	0	2	0	1	0	0
0	0	0	2	0	0	0	0
0	0	0	3	0	0	0	0
0	0	0	4	0	0	0	0
0	0	1	3	0	0	0	1
0	0	0	4	0	0	0	0
0	0	0	2	0	0	0	0
0	0	0	2	0	0	0	0
0	0	0	4	0	0	0	0
0	0	0	3	0	0	0	0
0	0	0	2	0	0	0	0
0	0	0	2	0	0	0	0
0	0	0	3	0	0	0	0
0	0	0	3	1	0	0	0
0	0	0	3	0	1	0	0
0	0	0	3	0	0	0	0
0	0	0	3	0	1	0	0
0	0	0	3	0	0	0	0
0	0	0	4	0	0	0	0
0	0	0	3	0	0	0	0
0	0	0	3	0	0	0	0
0	0	0	2	0	0	0	0
0	0	0	3	0	0	0	0
0	0	0	2	0	0	0	0
0	0	0	2	0	0	0	0
0	0	0	2	0	0	0	0
0	0	0	2	0	0	0	0
0	0	0	3	0	0	0	0
0	0	0	3	0	0	0	0
0	0	0	3	0	0	0	0
0	0	0	3	0	0	0	0
0	0	0	3	1	1	0	0
0	0	0	3	0	0	0	0
0	0	0	3	0	0	0	0
0	0	0	3	0	0	0	0

0	0	0	3	0	0	0	0
0	0	0	3	0	0	0	0
0	0	0	3	0	0	0	0
0	0	0	3	0	0	0	0
0	0	0	3	0	0	0	0
0	0	0	3	0	1	0	0
0	0	0	2	0	0	0	0
0	0	1	2	0	0	0	0
0	0	0	3	1	0	0	0
0	0	0	3	0	0	0	0
0	0	0	3	0	1	0	0
0	0	0	4	0	0	0	0
0	0	0	3	0	0	0	0
0	0	0	3	0	0	0	0
0	0	0	3	0	0	0	0
0	0	0	2	0	0	0	0
0	0	0	3	0	0	0	1
0	0	0	3	0	0	0	0
0	0	0	3	0	0	0	0
0	0	0	5	0	0	0	0
0	0	0	5	0	0	0	1
0	0	0	2	1	1	0	0
0	0	0	2	0	0	0	0
0	0	0	3	0	0	0	0
0	0	0	1	0	0	0	0
0	0	0	2	0	0	0	0
0	0	0	2	0	0	0	0
0	0	0	2	0	0	0	0
0	0	0	5	0	0	0	0
0	0	0	3	0	0	0	0
0	0	0	3	0	0	0	0
0	0	0	3	0	0	0	0
0	0	0	3	0	0	0	0
0	0	0	2	0	1	0	0
0	0	0	2	0	0	0	0
0	0	0	2	0	0	0	0
0	0	0	3	0	0	0	0
0	0	0	2	0	0	0	0
0	0	0	2	0	0	0	1
0	0	0	2	0	0	0	0
0	0	0	4	0	0	0	1
0	0	0	5	0	0	0	0
0	0	0	2	0	0	0	0
0	0	0	3	0	0	0	0
0	0	0	3	0	0	0	1
0	0	0	1	0	0	0	0
0	0	0	2	1	0	0	0
0	0	0	2	0	0	0	0

0	0	0	2	0	0	0	0
0	0	0	2	0	0	0	0
0	0	0	2	0	0	0	0
0	0	1	3	0	0	0	0
0	0	0	3	0	0	0	0
0	0	0	3	0	0	0	0
0	0	0	2	0	0	0	0
0	0	0	2	0	0	0	0
0	0	0	3	0	0	0	0
0	0	0	2	0	0	0	0
0	0	0	5	0	0	0	0
0	0	0	2	1	0	0	0
0	0	0	2	0	0	0	0
0	0	0	1	0	0	0	0
0	0	0	3	0	0	0	0
0	0	0	2	0	0	0	0
0	0	0	3	0	0	0	0
0	1	1	3	0	0	0	0
0	0	0	2	0	0	0	0
0	0	0	1	0	0	0	0
0	0	0	2	0	0	0	0
0	0	1	3	0	0	0	0
0	0	1	2	0	0	0	1
0	0	0	3	0	1	0	0
0	0	0	4	0	0	0	0
0	0	0	3	0	0	0	0
0	0	1	3	0	0	0	0
0	0	0	3	0	0	0	1
0	0	0	3	0	0	0	0
0	0	0	2	0	0	0	0
0	0	1	5	0	0	0	1
0	0	0	2	0	0	0	0
0	0	0	3	0	1	0	0
0	0	0	2	0	0	0	0
0	0	0	2	1	1	0	0
0	0	0	2	0	0	0	0
0	0	0	1	0	0	0	0
0	0	1	3	0	0	0	1
0	0	0	2	0	0	0	0
0	0	0	2	0	0	0	0
0	0	0	3	0	1	0	0
0	0	0	3	1	0	0	0
0	0	0	5	0	0	0	0
0	0	0	3	0	0	0	0
0	0	0	2	0	0	0	0
0	0	0	5	0	0	0	0
0	0	0	3	0	0	0	0
0	0	0	3	0	0	0	0

0	0	0	3	0	0	0	0
0	0	1	1	1	1	0	0
0	0	0	3	0	0	0	0
0	0	0	4	0	0	0	0
0	0	0	3	0	0	0	0
0	0	1	5	0	0	0	0
0	0	0	2	1	0	0	0
0	0	0	3	0	0	0	0
0	0	0	3	0	0	0	0
0	0	0	2	0	0	0	0
0	0	0	5	0	0	0	0
0	0	0	4	0	1	0	0
0	0	0	2	0	0	0	0
0	0	1	4	0	0	0	0
0	0	0	4	0	0	0	0
0	0	0	1	0	0	0	0
0	0	0	3	0	0	0	0
0	0	0	2	0	0	0	0
0	0	0	3	0	0	0	0
0	0	0	4	0	0	0	0
0	0	0	3	0	0	0	0
0	0	0	2	0	0	0	0
0	0	0	3	0	0	0	0
0	0	0	3	0	0	0	0
0	0	1	3	0	0	0	0
0	0	0	2	0	0	0	0
0	0	0	3	0	0	0	0
0	0	0	3	0	0	0	0
0	0	0	3	0	0	0	0
0	0	0	3	0	0	0	0
0	0	0	3	0	1	0	0
0	0	0	2	0	0	0	0
0	0	0	2	0	0	0	0
0	0	0	3	0	0	0	0
0	0	0	3	0	0	0	0
0	0	0	2	0	0	0	0
0	0	0	3	0	0	0	0
0	0	0	3	0	1	0	0
0	0	0	4	0	0	0	0
0	0	0	4	0	0	0	0
0	0	0	2	0	0	0	0
0	0	0	4	0	0	0	0
0	0	0	2	0	0	0	0
0	0	0	2	0	0	0	0
0	0	0	3	0	0	0	0
0	0	0	3	1	1	0	1
0	0	1	3	0	0	0	0
0	0	0	3	0	0	0	0

0	0	1	4	0	0	0	0
0	0	0	2	1	0	0	0
0	0	0	3	0	0	0	0
0	0	0	3	0	0	0	0
0	0	0	2	0	0	0	0
0	0	0	3	0	0	0	0
0	0	0	1	0	0	0	0
0	0	0	3	0	0	0	0
0	0	0	5	0	0	0	0
0	0	0	2	0	1	0	0
0	0	0	3	0	0	0	0
0	0	0	3	0	0	0	0
0	0	0	3	0	0	0	0
0	0	0	2	0	0	0	0
0	0	1	3	0	1	0	0
0	0	0	3	0	1	0	0
0	0	0	3	0	0	0	0
0	0	0	3	0	0	0	0
0	0	0	2	0	0	0	0
0	0	0	3	0	0	0	0
0	0	0	3	0	0	0	0
0	0	0	3	0	1	0	0
0	0	0	3	0	1	0	0
0	0	0	2	0	0	0	0
0	0	0	2	0	0	0	0
0	0	0	5	0	0	0	0
0	0	0	3	0	0	0	0
0	0	0	2	0	0	0	0
0	0	0	3	0	0	0	0
0	0	0	3	0	0	0	0
0	0	0	3	0	0	0	0
0	0	0	1	0	0	0	0
0	0	0	2	0	0	0	0
0	0	0	2	0	0	0	0
0	0	0	3	0	0	0	0
0	0	0	2	0	0	0	0
0	0	0	2	0	0	0	0
0	0	0	5	0	0	0	0
0	0	0	3	0	0	0	0
0	0	0	2	0	0	0	0
0	0	0	3	0	0	0	0
0	0	0	3	0	0	0	0
0	0	0	3	0	0	0	1
0	0	0	2	0	0	0	0
0	0	0	3	0	0	0	0
0	0	0	5	0	0	0	0
0	0	0	3	0	0	0	0
0	0	0	2	0	0	0	0

0	0	0	3	0	0	0	1
0	0	0	2	1	0	0	0
0	0	0	3	0	0	0	0
0	0	0	2	0	0	0	0
0	0	0	3	0	0	0	0
0	0	0	2	0	0	0	0
0	0	0	3	0	0	0	0
0	0	0	1	0	0	0	0
0	0	0	3	0	0	0	0
0	0	0	5	0	0	0	0
0	0	0	3	0	0	0	0
0	0	0	5	0	0	0	1
0	0	0	5	0	0	0	1
0	0	0	2	0	0	0	0
0	0	0	3	0	0	0	0
0	0	0	1	0	0	0	0
0	0	0	3	0	0	0	0
0	0	0	4	0	0	0	0
0	0	0	3	0	0	0	0
0	0	0	4	0	0	0	0
0	0	0	3	0	0	0	0
0	1	1	4	0	0	0	0
0	0	0	4	0	0	0	0
0	0	0	4	0	0	0	0
0	0	1	1	0	0	0	0
0	0	0	3	0	0	0	0
0	0	0	5	1	0	0	0
0	0	0	3	0	0	0	1
0	0	0	2	0	0	0	0
0	0	0	4	0	0	0	0
0	0	0	3	0	0	0	0
0	0	0	3	0	0	0	0
0	0	0	3	0	0	0	0
0	0	1	3	0	0	0	1
0	0	0	4	0	0	0	0
0	0	0	3	0	0	0	1
0	0	0	4	0	0	0	0
0	0	0	2	0	0	0	0
0	0	0	5	0	0	0	0
0	0	0	3	0	0	0	0
0	0	1	4	0	0	0	0
0	0	0	2	0	0	0	0
0	0	0	3	0	0	0	0
0	0	0	2	0	0	0	0
0	0	0	2	0	0	0	0
0	0	0	2	0	0	0	0
0	0	0	2	0	0	0	0
0	0	0	3	0	0	0	0

0	0	0	3	0	1	0	0
0	0	0	3	0	0	0	0
0	0	0	3	0	1	0	0
0	0	0	2	0	0	0	0
0	0	0	3	0	0	0	0
0	0	0	3	0	0	0	0
0	0	0	3	0	0	0	0
0	0	0	3	0	0	0	0
0	0	0	1	0	0	0	0
0	0	0	2	0	0	0	0
0	0	0	1	0	0	0	0
0	0	0	2	0	0	0	0
0	0	0	3	0	0	0	0
0	0	0	2	0	0	0	0
0	0	0	5	0	0	0	0
0	0	0	3	0	0	0	0
0	0	0	3	0	0	0	0
0	0	0	2	0	0	0	0
0	0	0	1	0	0	0	1
0	0	0	3	0	1	0	0
0	0	0	3	0	0	0	0
0	0	0	2	0	0	0	0
0	0	1	4	0	0	0	0
0	0	0	4	0	0	0	0
0	0	0	2	0	0	0	0
0	0	1	3	0	0	0	0
0	0	0	3	0	0	0	0
0	0	0	2	0	0	0	0
0	0	0	3	0	1	0	0
0	0	0	1	1	1	0	0
0	0	0	2	0	0	0	0
0	0	1	5	0	0	0	0
0	0	0	2	0	0	0	0
0	0	0	3	1	0	0	0
0	0	0	2	0	0	0	0
0	0	0	2	0	0	0	0
0	0	0	3	0	0	0	0
0	0	0	3	0	0	0	0
0	0	0	2	0	1	0	0
0	0	0	3	0	0	0	0
0	0	0	2	0	0	0	0
0	0	0	3	0	0	0	0
0	0	0	4	0	0	0	0
0	0	0	2	0	0	0	0
0	0	0	3	0	1	0	0
0	0	0	3	0	0	0	0
0	0	0	1	0	0	0	0
0	0	0	1	0	0	0	0

0	0	0	2	0	0	0	0
0	0	0	2	0	0	0	0
0	0	0	2	0	0	0	0
0	0	0	2	0	0	0	0
0	0	0	3	0	0	0	0
0	0	0	2	0	0	0	0
0	0	0	3	0	0	0	0
0	0	0	2	0	0	0	0
0	0	0	2	0	0	0	0
0	0	0	2	0	0	0	0
0	0	0	2	0	0	0	0
0	0	0	2	0	0	0	0
0	0	0	2	0	0	0	0
0	0	0	2	0	0	0	0
0	0	0	2	0	0	0	0
0	0	0	4	0	0	0	0
0	0	0	2	0	0	0	0
0	0	0	2	0	1	0	0
0	0	0	1	0	1	0	0
0	1	0	5	1	0	0	1
0	0	0	3	0	0	0	0
0	0	0	2	0	0	0	0
0	0	0	3	0	0	0	0
0	0	0	3	0	0	0	0
0	0	0	3	0	0	0	1
0	0	0	5	0	0	0	1
0	0	0	3	0	0	0	0
0	0	0	1	0	0	0	1
0	0	0	2	0	0	0	0
0	0	0	3	0	0	0	0
0	0	0	2	0	0	0	0
0	0	0	1	0	0	0	0
0	0	0	3	0	0	0	1
0	0	0	2	0	0	0	0
0	0	0	3	0	0	0	0
0	0	0	3	0	0	0	0
0	0	0	3	0	0	0	0
0	0	0	3	0	0	0	1
0	0	1	3	0	0	0	1
0	0	0	3	0	0	0	0
0	0	0	3	0	0	0	0
0	0	0	3	0	0	0	0
0	0	0	4	0	0	0	0
0	0	0	3	0	0	0	0
0	0	0	3	0	0	0	0
0	0	0	3	0	0	0	0
0	0	0	2	0	0	0	0
0	0	0	3	0	1	0	1

0	0	0	3	0	0	0	1
0	0	0	3	0	1	0	1
0	0	0	3	0	0	0	0
0	0	0	2	0	0	0	0
0	0	0	3	0	0	0	0
0	0	0	4	0	0	0	0
0	0	0	5	0	0	0	1
0	0	0	2	0	0	0	1
0	0	0	3	0	0	0	0
0	0	0	4	0	0	0	0
0	0	0	3	0	0	0	0
0	0	0	3	0	0	0	0
0	0	0	2	0	0	0	0
0	0	0	3	0	0	0	0
0	0	0	3	0	0	0	0
0	0	0	2	0	0	0	0
0	0	0	2	0	0	0	0
0	0	0	3	0	0	0	0
0	0	0	2	0	0	0	0
0	0	1	5	0	0	0	1
0	0	1	3	0	0	0	1
0	0	0	3	0	1	0	1
0	0	0	3	0	0	0	0
0	0	0	3	0	1	0	0
0	0	0	3	0	0	0	0
0	0	0	5	0	1	0	0
0	0	0	3	0	0	0	1
0	0	0	3	0	0	0	0
0	0	0	3	0	0	0	0
0	0	0	3	0	0	0	0
0	0	0	2	0	1	0	0
0	0	0	3	0	0	0	0
0	0	0	3	0	0	0	0
0	0	0	3	0	0	0	0
0	0	0	2	0	0	0	0
0	0	0	2	0	1	0	0
0	0	0	1	0	0	0	0
0	0	0	3	0	0	0	0
0	0	0	1	0	0	0	0
0	0	0	3	0	0	0	0
0	0	0	2	0	0	0	0
0	0	0	3	0	0	0	0
0	0	0	3	0	0	0	0
0	0	0	3	0	0	0	0
0	0	0	1	0	0	0	0
0	0	0	5	0	0	0	0
0	0	0	3	0	0	0	1
0	0	0	2	0	0	0	0

0	0	0	3	0	0	0	0
0	0	0	2	0	0	0	0
0	0	0	3	0	0	0	0
0	0	0	3	0	0	0	0
0	0	0	3	0	0	0	0
0	0	0	3	0	0	0	0
0	0	0	3	0	0	0	0
0	0	0	4	0	0	0	0
0	0	0	2	0	0	0	0
0	0	0	4	0	0	0	0
0	0	0	3	0	1	0	0
0	0	0	3	0	0	0	0
0	0	0	5	1	0	0	1
0	0	0	1	0	0	0	0
0	0	0	3	0	0	0	0
0	0	1	3	1	1	0	0
0	0	0	2	0	0	0	0
0	0	0	3	0	0	0	0
0	0	0	3	0	0	0	0
0	0	0	3	0	0	0	0
0	0	0	3	0	0	0	0
0	0	0	3	0	0	0	0
0	0	0	2	0	0	0	0
0	0	0	2	0	0	0	0
0	0	0	3	0	0	0	0
0	0	0	2	0	0	0	0
0	0	0	2	0	0	0	0
0	0	0	3	0	0	0	0
0	0	0	2	0	0	0	0
0	0	0	2	0	0	0	0
0	0	0	1	0	0	0	0
0	0	0	2	0	0	0	0
0	0	0	3	0	0	0	0
0	0	0	3	0	1	0	0
0	0	0	2	0	0	0	0
0	0	0	3	0	0	0	0
0	0	0	2	0	0	0	0
0	0	0	3	0	0	0	0
0	0	0	2	0	1	0	0
0	0	0	2	0	0	0	0
0	0	0	3	0	0	0	0
0	0	0	3	0	0	0	0
0	0	0	3	0	1	0	1
0	0	0	1	0	0	0	0
0	0	0	1	0	0	0	0
0	0	0	3	0	0	0	0
0	0	0	2	0	0	0	0
0	0	0	5	0	0	0	1

0	0	0	3	0	0	0	0
0	0	0	3	0	0	0	0
0	1	0	3	0	0	0	0
0	0	0	3	0	0	0	0
0	0	0	5	0	0	0	1
0	0	0	3	0	1	0	0
0	0	0	2	0	0	0	0
0	0	0	3	0	0	0	0
0	0	0	2	0	0	0	0
0	0	0	2	0	0	0	0
0	0	0	3	0	0	0	0
0	0	0	5	0	0	0	0
0	1	0	3	0	0	0	1
0	0	0	3	0	0	0	0
0	0	0	3	0	0	0	0
0	0	0	2	0	0	0	0
0	0	0	1	0	0	0	0
0	0	0	3	0	0	0	0
0	0	0	3	0	0	0	0
0	0	0	3	0	0	0	0
0	0	0	3	0	1	0	0
0	0	0	3	1	1	0	0
0	0	0	3	0	0	0	1
0	0	0	2	0	0	0	0
0	0	0	3	0	0	0	0
0	0	1	3	1	1	0	1
0	0	0	3	0	0	0	1
0	0	0	3	0	0	0	0
0	0	0	2	0	1	0	0
0	0	0	3	0	0	0	0
0	0	0	3	0	1	0	0
0	0	0	3	0	0	0	0
0	0	0	3	0	0	0	0
0	0	0	2	0	0	0	0
0	0	0	3	0	0	0	1
0	0	0	3	0	0	0	0
0	0	0	2	0	0	0	0
0	0	0	2	0	1	0	0
0	0	0	3	0	0	0	0
0	0	0	3	0	0	0	0
0	0	0	3	0	0	0	0
0	0	0	3	0	0	0	0
0	0	0	3	0	0	0	0
0	0	0	3	0	0	0	0
0	0	0	3	0	0	0	0
0	0	0	3	0	0	0	0
0	0	0	3	0	0	0	0
0	0	0	3	0	0	0	0

0	0	0	2	0	0	0	0
0	0	0	3	1	1	0	0
0	0	0	2	0	1	0	0
0	0	0	3	0	0	0	0
0	0	0	3	0	1	0	0
0	0	0	3	1	0	0	0
0	0	0	3	1	0	0	0
0	0	0	2	0	1	0	0
0	0	0	2	0	0	0	0
0	0	0	2	0	0	0	0
0	0	0	3	0	1	0	0
0	0	0	1	0	1	0	0
0	0	0	3	0	1	0	0
0	0	0	3	0	0	0	0
0	0	0	3	0	0	0	0
0	0	0	2	0	0	0	0
0	0	0	3	0	1	0	0
0	0	0	3	0	0	0	0
0	0	0	3	0	0	0	0
0	0	0	2	0	1	0	0
0	0	0	3	0	0	0	0
0	0	0	3	0	0	0	0
0	0	0	2	0	0	0	0
0	0	0	2	0	0	0	0
0	0	0	3	0	1	0	0
0	0	0	2	0	0	0	0
0	0	0	2	0	0	0	0
0	0	0	2	0	0	0	0
0	0	0	2	0	0	0	0
0	0	0	3	0	0	0	0
0	0	0	3	0	0	0	0
0	0	0	2	0	1	0	0
0	0	0	2	0	0	0	0
0	0	0	3	0	0	0	0
0	0	0	2	1	1	0	0
0	0	0	2	0	1	0	0
0	0	0	2	0	1	0	0
0	0	0	3	0	0	0	0
0	0	0	3	0	0	0	0
0	0	0	2	0	0	0	0
0	0	0	3	1	1	0	0
0	0	0	2	0	0	0	0
0	0	0	2	0	0	0	0
0	0	0	3	0	0	0	0
0	0	0	3	0	0	0	0
0	0	0	2	0	0	0	0
0	0	0	3	0	0	0	0
0	0	0	3	1	1	0	0

0	0	0	3	0	1	0	0
0	0	0	2	0	0	0	0
0	0	0	2	1	1	0	0
0	0	0	3	0	0	0	0
0	0	0	2	0	0	0	0
0	0	0	3	0	0	0	0
0	0	0	2	0	0	0	0
0	0	0	2	0	0	0	0
0	0	0	3	0	0	0	0
0	0	0	1	0	0	0	0
0	0	0	3	0	0	0	0
0	0	0	3	0	0	0	0
0	0	0	3	0	0	0	0
0	0	0	1	1	0	0	0
0	0	0	3	0	0	0	0
0	0	0	3	0	1	0	0
0	0	0	3	0	0	0	0
0	0	0	1	0	0	0	0
0	0	0	3	0	0	0	0
0	0	0	2	0	0	0	0
0	0	0	2	0	1	0	0
0	0	0	3	0	0	0	0
0	0	0	5	0	0	0	0
0	0	0	3	0	0	0	0
0	0	0	1	0	0	0	0
0	0	0	3	0	0	0	0
0	0	0	3	0	0	0	0
0	0	0	2	0	0	0	0
0	0	0	1	0	0	0	0
0	0	0	3	0	0	0	0
0	0	0	3	0	0	0	0
0	0	0	3	0	0	0	0
0	0	0	2	1	1	0	0
0	0	0	3	0	0	0	0
0	0	0	3	0	0	0	0
0	0	0	3	0	0	0	0
0	0	0	2	0	1	0	0
0	0	0	1	0	0	0	0
0	0	0	2	0	0	0	0
0	0	0	2	0	0	0	0
0	0	0	2	0	0	0	0
0	0	0	3	0	0	0	0
0	0	0	1	1	0	0	0
0	0	0	1	0	0	0	0
0	0	0	3	0	0	0	0
0	0	0	2	1	1	0	0
0	0	0	1	0	0	0	0
0	0	0	2	1	0	0	0

0	0	0	3	0	0	0	0
0	0	0	3	0	0	0	0
0	0	0	3	0	1	0	0
0	0	0	3	0	0	0	0
0	0	0	1	0	0	0	0
0	0	0	2	0	0	0	0
0	0	0	2	0	0	0	0
0	0	0	2	0	0	0	0
0	0	0	2	1	1	0	0
0	0	0	3	0	0	0	0
0	0	0	3	0	0	0	0
0	0	0	1	0	0	0	0
0	0	0	3	0	0	0	0
0	0	0	1	0	0	0	0
0	0	0	2	0	0	0	0
0	0	0	2	0	0	0	0
0	0	0	3	0	0	0	0
0	0	0	1	0	0	0	0
0	0	0	3	0	0	0	0
0	0	0	2	0	0	0	0
0	0	0	2	0	0	0	0
0	0	0	2	0	1	0	0
0	0	0	1	0	0	0	0
0	0	0	2	0	0	0	0
0	0	0	1	0	0	0	0
0	0	0	2	0	1	0	0
0	0	0	3	0	0	0	0
0	0	0	2	0	0	0	0
0	0	0	1	0	0	0	0
0	0	0	3	0	0	0	0
0	0	0	1	0	0	0	0
0	0	0	2	0	0	0	0
0	0	0	1	0	0	0	0
0	0	0	2	0	0	0	0
0	0	0	2	0	0	0	0
0	0	0	3	0	0	0	0
0	0	0	3	0	0	0	0
0	0	0	3	0	0	0	0
0	0	0	3	0	1	0	0
0	0	0	2	0	0	0	0
0	0	0	1	0	0	0	0
0	0	0	3	0	0	0	0
0	0	0	2	0	0	0	0
0	0	0	1	0	0	0	0
0	0	0	3	0	0	0	0
0	0	0	3	0	0	0	0
0	0	0	3	0	0	0	0
0	0	0	3	1	0	0	0

0	0	0	2	0	1	0	0
0	0	0	2	0	0	0	0
0	0	0	2	1	1	0	0
0	0	0	2	0	0	0	0
0	0	0	2	0	0	0	0
0	0	0	3	0	0	0	0
0	0	0	1	0	0	0	0
0	0	0	1	0	0	0	0
0	0	0	2	0	1	0	0
0	0	0	2	0	0	0	0
0	0	0	2	0	0	0	0
0	0	0	3	0	0	0	0
0	0	0	1	0	0	0	0
0	0	0	1	0	0	0	0
0	0	0	2	0	0	0	0
0	0	0	3	0	0	0	0
0	0	0	3	0	0	0	0
0	0	0	3	1	1	0	0
0	0	0	2	0	0	0	0
0	0	0	2	0	0	0	0
0	0	0	3	0	0	0	0
0	0	0	2	0	0	0	0
0	0	0	3	0	0	0	0
0	0	0	3	0	0	0	0
0	0	1	3	0	0	0	0
0	0	0	2	0	0	0	0
0	0	0	3	0	0	0	0
0	0	0	3	0	0	0	0
0	0	0	3	0	1	0	0
0	0	0	2	0	0	0	0
0	0	0	2	0	0	0	0
0	0	0	1	0	0	0	0
0	0	0	3	0	0	0	0
0	0	1	6	0	1	0	0
0	0	0	2	0	0	0	0
0	0	0	2	0	0	0	0
0	0	0	2	0	0	0	0
0	0	0	4	0	1	0	0
0	0	0	2	0	1	0	0
0	0	0	2	0	0	0	0
0	0	0	2	0	0	0	0
0	0	0	3	1	1	1	0
0	0	0	2	0	0	0	0
0	0	0	2	0	0	0	0
0	0	0	3	0	0	0	0
0	0	0	3	0	0	0	0
0	0	0	3	0	0	0	0
0	0	0	1	0	0	0	0

0	0	0	2	0	0	0	0
0	0	0	3	0	0	0	0
0	0	0	3	0	0	0	0
0	0	0	2	0	0	0	0
0	0	0	3	0	0	0	0
0	0	1	3	0	0	0	0
0	0	0	3	0	0	0	0
0	0	0	3	0	0	0	0
0	0	0	3	0	0	0	0
0	0	0	3	0	0	0	0
0	0	1	2	0	0	0	0
0	0	0	2	0	0	0	0
0	0	0	2	0	0	0	0
0	0	0	3	0	0	0	0
0	0	0	3	0	0	0	0
0	0	0	3	0	0	0	0
0	0	0	3	0	0	0	0
0	0	0	6	0	0	0	0
0	0	1	5	0	0	0	0
0	0	0	2	0	0	0	0
0	0	0	2	0	0	0	0
0	0	0	3	0	0	0	0
0	0	0	3	0	0	0	0
0	0	0	2	0	0	0	0
0	0	0	2	0	0	0	0
0	0	0	3	0	0	0	1
0	0	0	3	0	0	0	0
0	0	0	6	0	0	0	0
0	0	0	3	0	0	0	0
0	0	0	3	0	0	0	0
0	0	1	3	0	0	0	1
0	0	0	3	0	0	0	0
0	0	0	3	0	0	0	0
0	0	0	3	0	1	0	0
0	0	0	3	0	0	0	0
0	0	0	3	0	0	0	0
0	0	0	3	0	0	0	1
0	0	1	2	0	0	0	0
0	0	0	3	0	0	0	0
0	0	0	1	0	0	0	0
0	0	0	3	0	0	0	1
0	0	0	3	0	0	0	0
0	0	0	1	0	0	0	0
0	0	0	2	0	0	0	0
0	0	0	2	0	0	0	0
0	0	0	3	0	0	0	0
0	0	0	3	0	0	0	0
0	0	0	2	0	0	0	0

0	0	0	1	0	0	0	0
0	0	0	2	0	0	0	0
0	0	0	6	0	0	0	0
0	0	0	3	0	0	0	0
0	0	0	5	0	0	0	0
0	0	0	5	0	0	0	0
0	0	0	3	0	0	0	0
0	0	0	3	0	0	0	0
0	0	0	3	0	0	0	0
0	0	0	3	0	0	0	0
0	0	0	3	0	0	0	0
0	0	0	3	0	0	0	0
0	0	0	3	0	0	0	0
0	0	0	5	0	0	0	0
0	0	0	5	0	0	0	0
0	0	0	3	0	0	0	0
0	0	0	3	0	0	0	0
0	0	0	2	0	0	0	0
0	0	0	3	0	0	0	0
0	0	0	3	0	0	0	0
0	0	0	3	0	0	0	0
0	0	0	3	0	0	0	0
0	0	0	2	0	0	0	1
0	0	0	2	0	0	0	0
0	0	0	2	0	0	0	0
0	0	0	3	0	0	0	0
0	0	0	3	0	0	0	0
0	0	1	3	0	0	0	0
0	0	0	3	0	0	0	0
0	0	0	4	0	0	0	0
0	0	0	1	0	0	0	0
0	0	0	3	0	0	0	0
0	0	0	3	0	0	0	1
