# Supplementary material for: Comparison of influencing factors on outcomes of single and multiple road traffic injuries: A regional study in Shanghai, China (2011-2014)
Source: PLoS One. 2017 May 11;12(5):e0176907. doi: 10.1371/journal.pone.0176907 (PMC5426634; doi:10.1371/journal.pone.0176907)
Supplement: S2 Dataset — (PDF) [file pone.0176907.s002.pdf]

ID	Gender	Age	Marital status	Admission pathway	Prehospital time	Outcome
1	1	4	3	3	1	3
2	2	5	3	2	3	3
3	2	4	3	2	3	4
4	1	3	2	3	3	4
5	1	2	2	3	3	3
6	1	6	3	3	3	4
7	1	6	3	3	2	4
8	1	7	2	3	3	4
9	1	4	3	3	1	4
10	1	6	3	3	3	4
11	1	6	2	2	3	3
12	2	6	3	3	2	4
13	2	5	3	3	3	3
14	2	6	2	3	3	3
15	1	3	3	3	2	3
16	2	6	2	3	3	3
17	1	4	3	3	3	3
18	1	5	2	2	3	3
19	1	6	3	3	3	3
20	2	5	3	2	2	4
21	2	2	2	3	2	3
22	2	5	3	3	1	4
23	2	5	3	3	2	4
24	1	4	2	3	3	3
25	1	7	3	3	3	4
26	2	5	2	2	3	4
27	1	5	3	3	1	4
28	2	3	2	3	3	3
29	1	5	3	3	2	4
30	1	6	3	3	3	1
31	1	4	3	3	1	3
32	2	5	3	1	3	3
33	2	4	3	3	1	4
34	1	6	3	3	2	4
35	2	4	3	3	2	4
36	1	5	3	3	3	4
37	1	6	2	3	3	4
38	1	3	2	3	2	1
39	1	6	3	3	1	4
40	1	6	3	3	3	3
41	1	7	3	3	2	3
42	1	2	2	3	3	3
43	2	6	3	3	2	4
44	1	6	3	2	3	4
45	1	7	3	3	2	1
46	1	5	3	3	3	3
47	2	7	3	3	2	4

48	2	7	3	3	1	4
49	1	7	3	3	3	4
50	2	7	3	3	2	3
51	2	6	3	3	2	3
52	1	7	3	3	2	4
53	1	7	3	3	1	4
54	1	6	3	3	2	4
55	1	5	1	3	3	3
56	1	6	3	3	2	4
57	1	5	2	3	2	3
58	1	4	3	3	2	4
59	1	5	3	3	2	4
60	1	5	3	3	3	3
61	1	5	3	3	2	4
62	1	4	3	3	3	3
63	2	5	3	3	3	4
64	2	5	3	3	2	3
65	2	6	3	3	3	4
66	2	6	3	3	2	4
67	1	3	2	2	3	3
68	1	5	3	3	2	4
69	1	7	3	3	2	4
70	1	6	3	2	3	3
71	1	3	3	3	3	4
72	2	4	3	2	3	3
73	1	7	3	3	1	4
74	2	3	2	2	3	4
75	1	6	3	3	3	3
76	1	6	3	3	3	3
77	1	6	3	3	3	3
78	1	5	3	3	3	3
79	1	4	3	3	3	3
80	2	7	3	3	2	1
81	1	7	3	3	3	3
82	1	6	3	3	2	4
83	2	4	3	3	3	3
84	1	5	3	2	3	3
85	2	7	3	3	3	3
86	1	6	3	2	3	4
87	1	6	3	3	2	3
88	1	5	3	3	3	4
89	2	7	3	3	1	3
90	2	7	2	3	2	3
91	1	7	2	3	2	3
92	1	5	3	2	3	4
93	1	7	3	3	3	1
94	1	4	2	3	3	4
95	2	6	3	3	3	3

96	1	7	3	3	3	4
97	1	7	3	3	3	3
98	1	7	3	2	3	4
99	2	5	3	3	3	4
100	1	7	3	3	2	4
101	2	7	3	2	3	4
102	1	3	2	2	2	4
103	1	5	3	3	3	4
104	1	6	3	2	3	4
105	1	4	3	3	1	4
106	2	6	2	2	3	4
107	2	7	3	3	3	4
108	1	5	3	3	3	3
109	1	2	2	3	3	3
110	1	6	3	3	3	4
111	2	5	3	2	3	3
112	1	3	3	3	2	3
113	1	3	2	3	3	3
114	2	5	3	3	3	3
115	1	3	3	3	3	4
116	1	6	3	2	3	4
117	2	6	3	2	3	3
118	1	6	3	3	2	3
119	2	5	2	3	2	4
120	1	3	3	3	2	3
121	1	3	3	3	3	3
122	1	7	3	3	3	3
123	1	5	3	3	1	3
124	1	4	3	3	2	4
125	1	6	3	3	2	1
126	2	7	3	3	2	4
127	2	2	2	3	1	4
128	1	7	3	3	3	3
129	1	3	3	3	2	4
130	1	2	2	2	3	4
131	2	6	2	3	2	3
132	2	5	2	3	3	4
133	1	6	3	3	1	4
134	1	4	3	3	2	4
135	1	7	3	3	2	4
136	2	3	3	3	2	3
137	2	2	2	3	2	4
138	2	3	2	3	3	4
139	1	5	3	3	1	4
140	1	5	3	3	2	4
141	2	4	3	3	3	4
142	2	6	3	3	1	4
143	1	6	3	3	2	3

144	2	7	3	3	3	3
145	1	4	2	3	3	3
146	1	3	3	2	2	3
147	2	3	3	3	2	3
148	1	5	3	3	1	4
149	1	7	3	3	2	3
150	2	3	2	3	3	3
151	1	7	3	3	2	1
152	1	4	3	3	3	3
153	2	6	3	3	2	3
154	2	4	3	3	2	4
155	2	5	3	3	3	4
156	1	5	3	3	3	3
157	1	3	3	2	3	4
158	1	3	3	3	2	3
159	1	2	2	3	2	3
160	1	7	3	3	2	3
161	1	5	3	3	2	4
162	2	5	3	3	2	3
163	1	3	2	3	2	4
164	1	5	2	3	1	4
165	1	4	3	2	3	4
166	2	5	3	3	2	4
167	2	6	3	3	2	4
168	1	7	3	3	3	4
169	1	7	2	3	3	3
170	1	6	3	3	2	1
171	1	2	2	3	1	4
172	2	5	3	3	3	4
173	2	5	3	3	2	4
174	1	7	3	3	3	3
175	1	6	3	3	3	4
176	1	4	2	3	3	3
177	1	3	2	3	1	3
178	2	4	3	3	3	4
179	1	6	3	3	3	3
180	1	6	3	3	3	3
181	1	5	3	3	3	3
182	1	4	3	3	3	3
183	2	5	3	2	3	4
184	2	6	3	3	3	4
185	2	4	3	3	2	4
186	2	5	3	1	3	4
187	1	5	3	2	3	4
188	1	7	2	3	3	1
189	2	6	3	3	1	3
190	2	7	3	2	2	4
191	2	6	3	3	3	3

192	1	7	3	2	3	3
193	1	5	3	3	3	4
194	1	6	3	2	2	4
195	1	6	2	3	2	4
196	1	4	3	3	2	4
197	2	7	3	3	2	3
198	1	7	3	3	3	4
199	1	6	3	3	3	4
200	1	5	3	3	3	4
201	1	6	3	3	1	4
202	1	3	2	3	2	4
203	1	7	3	2	3	4
204	1	7	3	3	2	3
205	1	4	3	3	3	4
206	2	6	3	3	1	3
207	1	5	3	3	1	3
208	1	3	3	3	1	3
209	1	4	3	3	2	4
210	1	5	3	3	2	4
211	2	4	3	3	3	4
212	2	4	3	3	3	1
213	2	4	3	3	3	3
214	2	5	3	3	3	4
215	2	7	3	3	3	3
216	2	5	3	3	2	4
217	1	7	3	3	3	4
218	2	7	3	3	3	3
219	1	6	3	3	3	3
220	1	6	3	3	3	2
221	2	4	3	3	2	4
222	1	5	3	3	3	4
223	2	7	3	3	2	4
224	1	5	3	3	2	4
225	2	4	3	3	2	3
226	1	5	3	3	3	4
227	1	5	3	3	3	3
228	1	6	3	3	2	3
229	1	5	2	3	3	4
230	1	7	3	1	3	3
231	1	5	3	3	2	4
232	1	7	3	3	1	4
233	1	6	3	2	3	4
234	2	6	3	3	2	3
235	1	7	2	3	3	3
236	2	7	3	3	3	4
237	1	7	3	3	2	3
238	1	3	2	3	2	3
239	1	6	3	2	3	4

240	1	7	3	3	3	4
241	1	5	3	2	2	3
242	2	6	3	1	3	3
243	2	4	2	3	3	4
244	1	4	2	3	1	4
245	1	1	2	2	2	3
246	1	7	3	3	2	4
247	1	5	3	1	3	3
248	1	7	3	2	3	1
249	1	5	3	3	2	4
250	1	6	3	3	3	3
251	1	4	3	3	3	3
252	2	6	3	3	3	3
253	1	4	3	2	3	3
254	1	6	3	2	3	4
255	2	6	3	3	2	3
256	1	5	3	3	3	4
257	1	5	2	3	3	4
258	2	6	3	2	3	4
259	1	3	3	1	3	4
260	2	3	2	3	3	3
261	1	6	3	3	2	4
262	1	6	3	3	1	3
263	1	6	3	3	3	2
264	1	4	3	3	2	4
265	2	7	3	3	2	3
266	1	5	2	3	3	3
267	2	7	2	3	1	1
268	1	3	3	3	2	3
269	1	7	2	3	3	1
270	1	6	3	3	2	4
271	1	6	3	3	1	4
272	1	6	3	3	3	3
273	1	5	3	3	2	3
274	1	1	2	3	3	3
275	2	4	3	3	2	4
276	1	4	3	3	2	2
277	1	4	3	3	2	4
278	1	6	3	3	3	4
279	2	7	3	3	3	4
280	2	5	2	3	4	3
281	1	5	3	3	4	3
282	1	3	3	3	4	3
283	1	6	3	1	4	4
284	2	7	3	3	4	3
285	1	2	3	1	4	4
286	2	4	3	1	4	3
287	1	5	3	3	4	3

288	1	5	3	1	4	4
289	1	6	3	1	4	4
290	2	3	3	1	4	3
291	1	2	2	3	4	3
292	1	7	2	2	4	3
293	1	2	2	1	4	4
294	1	5	3	1	4	3
295	2	4	3	1	4	4
296	1	1	2	1	4	4
297	1	3	2	1	4	4
298	2	5	3	1	4	3
299	1	1	2	1	4	3
300	2	6	3	3	4	4
301	2	2	2	1	4	4
302	2	6	2	1	4	3
303	2	2	2	1	4	3
304	1	7	3	1	4	4
305	1	7	3	1	4	2
306	1	3	3	2	4	3
307	1	6	3	3	4	4
308	1	1	2	1	4	3
309	2	6	3	1	4	3
310	2	6	3	1	4	4
311	1	5	3	1	4	3
312	1	6	3	3	4	4
313	1	7	3	2	4	3
314	2	7	3	2	4	3
315	1	3	3	1	4	2
316	1	7	2	3	4	3
317	2	4	3	3	4	3
318	1	2	3	1	4	4
319	1	4	3	1	4	3
320	1	6	2	3	4	4
321	2	7	2	1	4	3
322	2	7	3	1	4	3
323	1	4	2	1	4	4
324	1	3	3	2	4	4
325	2	6	3	2	4	3
326	2	3	3	1	4	3
327	1	6	3	1	4	3
328	1	1	2	1	4	3
329	2	5	3	1	4	4
330	1	6	3	1	4	4
331	1	4	3	1	4	3
332	2	5	2	1	4	4
333	2	7	3	1	4	3
334	2	2	2	1	4	3
335	2	5	3	1	4	4

336	1	5	3	1	4	2
337	2	2	2	3	4	1
338	1	2	2	1	4	4
339	2	4	3	1	3	3
340	1	4	3	2	3	4
341	1	5	3	3	1	4
342	1	6	3	3	3	3
343	2	7	3	3	3	1
344	1	7	3	2	3	3
345	1	4	3	2	3	3
346	1	5	3	2	3	3
347	1	6	3	2	3	4
348	2	6	3	3	1	4
349	1	2	2	2	3	3
350	1	3	2	3	3	4
351	2	4	3	2	3	3
352	2	3	2	3	3	3
353	1	7	3	3	3	1
354	1	7	3	1	3	2
355	1	6	3	1	3	4
356	1	6	3	2	3	3
357	2	3	2	3	3	4
358	1	5	3	3	2	4
359	1	4	3	3	3	3
360	1	3	2	2	3	4
361	1	6	3	3	3	4
362	2	3	2	3	3	3
363	1	3	3	3	3	4
364	1	6	3	2	3	4
365	1	5	3	3	3	3
366	2	5	3	3	3	4
367	1	6	3	3	3	4
368	1	3	3	2	3	4
369	1	7	3	3	1	4
370	2	5	3	3	3	4
371	2	7	3	2	3	4
372	1	5	3	3	1	1
373	1	6	3	3	3	4
374	1	7	3	3	2	1
375	1	5	3	3	3	4
376	2	3	3	1	3	4
377	2	2	2	3	3	3
378	1	2	2	3	3	4
379	1	6	3	3	1	3
380	1	3	3	3	3	3
381	2	5	3	3	3	4
382	1	4	3	1	3	4
383	2	6	3	3	3	4

384	1	5	3	1	4	3
385	1	5	3	1	4	3
386	2	2	2	2	4	3
387	1	5	3	1	4	4
388	1	6	3	2	4	4
389	2	6	3	2	4	4
390	1	5	3	1	4	3
391	1	6	3	2	4	3
392	2	6	3	1	4	4
393	1	2	2	1	4	4
394	1	4	3	1	4	1
395	2	3	3	2	4	3
396	1	7	3	1	4	4
397	1	4	3	1	4	4
398	1	6	3	1	4	3
399	2	4	3	1	4	4
400	1	4	3	1	4	4
401	1	5	3	1	4	3
402	2	6	3	2	4	4
403	1	5	3	1	4	4
404	1	7	3	2	4	4
405	1	6	3	1	4	3
406	1	6	3	2	4	3
407	1	4	3	1	4	3
408	1	7	3	1	4	3
409	1	6	3	2	4	4
410	1	2	3	1	4	3
411	1	4	3	1	4	4
412	1	5	3	1	4	4
413	2	3	2	1	4	4
414	1	2	2	1	4	3
415	1	5	3	2	4	3
416	1	6	3	1	4	3
417	2	5	3	2	4	4
418	1	6	3	1	4	4
419	1	6	3	1	4	3
420	1	5	3	1	4	4
421	1	5	3	1	4	2
422	1	4	3	1	4	4
423	1	4	3	2	4	4
424	2	6	3	1	4	3
425	1	3	3	1	4	3
426	1	5	3	2	4	2
427	1	6	3	1	4	3
428	2	4	3	1	4	3
429	2	5	3	3	4	4
430	2	3	2	2	4	3
431	1	1	2	1	4	3

432	2	5	3	1	4	4
433	1	6	3	1	4	4
434	1	4	3	1	4	3
435	1	4	3	1	4	4
436	1	5	3	1	4	4
437	1	5	3	1	4	4
438	1	5	3	1	4	2
439	2	4	3	1	4	4
440	2	6	3	1	4	4
441	1	2	2	1	4	3
442	1	5	3	1	4	3
443	2	6	3	2	4	4
444	1	4	3	1	4	3
445	1	4	3	1	4	3
446	2	4	3	1	4	4
447	1	6	3	2	4	2
448	1	2	2	2	4	4
449	1	3	2	1	3	3
450	1	7	3	2	3	4
451	1	6	3	3	3	3
452	1	6	3	3	1	1
453	1	7	3	2	3	3
454	1	6	3	3	3	3
455	2	6	3	3	3	4
456	2	4	3	3	1	4
457	1	7	3	2	3	2
458	2	5	3	1	3	4
459	2	6	3	3	3	4
460	1	5	3	3	2	3
461	1	7	3	3	2	4
462	1	6	3	1	3	3
463	1	5	3	3	1	3
464	1	3	3	3	3	4
465	1	3	2	2	3	4
466	2	2	2	1	3	4
467	1	4	3	3	3	4
468	1	6	3	2	3	4
469	1	5	3	2	3	4
470	1	6	3	3	3	4
471	2	5	3	3	3	4
472	2	5	3	3	2	4
473	2	5	3	1	3	4
474	1	3	2	1	3	3
475	1	3	3	2	2	4
476	1	5	3	2	3	4
477	1	5	3	3	1	4
478	2	7	3	3	3	4
479	1	5	3	3	2	3

480	1	5	3	3	3	4
481	2	7	3	3	3	4
482	2	6	3	2	3	4
483	1	7	3	3	2	3
484	2	5	3	1	3	4
485	2	7	3	3	2	4
486	1	3	3	1	3	4
487	2	5	3	2	3	3
488	2	6	3	3	3	3
489	1	4	3	3	2	4
490	1	5	3	3	1	3
491	1	6	3	3	3	4
492	1	7	3	2	3	4
493	1	6	3	3	2	3
494	1	7	3	3	3	4
495	1	5	3	3	3	4
496	1	5	3	3	2	3
497	2	5	3	3	1	4
498	1	7	3	1	3	4
499	1	1	2	1	3	4
500	1	4	3	3	3	4
501	1	2	2	2	3	4
502	2	5	3	3	3	4
503	1	5	3	1	4	3
504	1	4	3	1	4	2
505	1	5	3	1	4	4
506	1	3	3	1	4	3
507	1	7	3	1	4	3
508	1	5	3	1	4	2
509	1	6	3	2	4	3
510	2	3	3	1	4	3
511	2	6	3	1	4	3
512	1	6	3	1	4	3
513	1	5	3	1	4	4
514	1	5	3	2	4	4
515	1	6	3	1	4	3
516	1	5	3	1	4	3
517	1	3	3	2	4	3
518	1	6	3	1	4	4
519	1	7	3	2	4	1
520	1	5	3	2	4	3
521	1	2	2	1	4	4
522	1	5	3	1	4	4
523	1	7	3	1	4	3
524	1	6	3	1	4	3
525	1	6	3	1	4	3
526	1	1	2	1	4	4
527	2	6	3	1	4	3

528	2	6	3	2	4	2
529	2	7	3	1	4	3
530	1	5	3	1	4	3
531	2	7	3	1	4	3
532	2	5	3	1	4	3
533	2	6	3	1	4	3
534	1	6	3	1	4	3
535	2	5	3	1	4	3
536	1	6	3	1	4	4
537	1	4	3	1	4	3
538	1	4	3	1	4	3
539	1	5	3	1	4	3
540	1	4	3	1	4	3
541	2	7	3	2	4	3
542	1	6	3	1	4	3
543	1	6	3	1	4	3
544	1	6	3	1	4	4
545	1	5	3	1	4	3
546	1	5	3	1	4	3
547	1	5	3	1	4	3
548	1	2	2	1	4	4
549	1	7	3	1	4	3
550	2	4	3	1	4	3
551	2	5	3	1	4	4
552	2	2	2	2	4	4
553	1	7	3	2	4	4
554	1	3	3	2	4	4
555	1	6	3	1	4	4
556	1	6	3	2	4	3
557	2	6	3	1	4	4
558	1	1	2	1	4	4
559	1	7	3	1	4	4
560	1	5	3	2	4	3
561	1	5	3	1	4	3
562	1	6	3	1	4	4
563	1	6	3	1	4	3
564	2	5	3	1	4	3
565	1	7	3	2	4	4
566	1	6	3	1	4	3
567	1	3	3	1	4	3
568	1	3	3	2	4	4
569	1	3	3	1	4	3
570	1	6	3	2	4	4
571	1	7	3	1	4	3
572	1	4	3	1	4	4
573	1	5	3	2	4	3
574	1	5	3	2	4	4
575	2	4	3	2	4	4

576	1	5	3	2	4	4
577	1	5	3	1	4	4
578	2	5	3	1	4	4
579	2	6	3	2	4	3
580	1	3	3	1	4	4
581	2	6	3	1	4	3
582	1	7	3	1	4	2
583	2	1	2	1	4	3
584	1	6	3	2	4	2
585	1	6	3	1	4	4
586	1	5	3	1	4	4
587	2	6	3	2	4	4
588	1	7	3	2	4	4
589	1	4	3	2	4	4
590	1	5	3	1	4	3
591	1	5	3	1	4	3
592	2	5	3	2	4	2
593	1	4	3	1	4	4
594	1	6	3	3	4	4
595	1	6	3	3	3	4
596	2	6	3	2	3	4
597	1	3	3	3	1	4
598	1	2	2	1	3	4
599	1	4	3	3	3	4
600	1	4	3	3	2	3
601	1	4	3	3	3	3
602	2	5	3	2	3	3
603	1	2	2	3	3	4
604	1	6	3	3	3	4
605	2	4	3	3	3	4
606	2	6	3	3	2	4
607	1	7	3	1	3	4
608	2	6	3	3	3	4
609	2	4	3	3	3	3
610	1	6	3	3	3	4
611	1	3	3	3	2	4
612	1	5	3	3	2	3
613	2	7	3	3	3	4
614	1	7	3	3	3	4
615	2	7	3	3	3	3
616	1	4	3	3	3	4
617	1	5	3	2	3	4
618	2	2	2	3	3	4
619	1	6	3	3	3	3
620	2	6	3	2	3	4
621	1	4	3	3	3	4
622	2	7	2	3	3	3
623	1	6	3	3	3	4

624	2	3	3	3	4	4
625	1	7	3	2	4	3
626	1	5	3	3	4	4
627	1	7	3	1	4	3
628	2	3	3	2	4	4
629	1	3	2	1	4	3
630	1	5	3	3	4	4
631	1	3	3	2	4	4
632	1	6	3	2	4	4
633	2	6	3	3	4	3
634	1	5	3	3	4	4
635	2	5	3	2	4	4
636	1	3	3	2	3	4
637	1	5	3	3	2	3
638	1	6	3	3	3	3
639	1	3	3	3	2	4
640	1	4	3	2	3	4
641	1	2	2	2	3	4
642	2	6	3	2	3	4
643	2	5	3	2	3	4
644	1	4	3	2	3	3
645	2	4	3	3	3	4
646	2	7	3	3	3	4
647	1	5	3	3	3	4
648	2	6	3	3	3	4
649	1	1	2	1	3	4
650	1	4	3	3	2	3
651	1	4	3	2	3	4
652	1	3	3	3	3	4
653	2	4	3	3	3	3
654	2	5	3	3	2	4
655	2	3	3	3	3	3
656	1	6	3	3	3	4
657	1	2	3	3	3	3
658	2	5	3	2	3	3
659	2	2	2	3	3	4
660	1	4	3	3	3	4
661	1	5	3	3	3	3
662	1	4	3	3	1	4
663	2	6	3	3	3	3
664	2	4	3	3	3	3
665	2	7	3	2	3	3
666	2	2	2	2	1	4
667	1	3	3	3	3	4
668	1	3	3	3	1	4
669	1	6	3	3	3	4
670	1	6	3	3	3	4
671	2	4	3	3	3	4

672	1	6	3	2	3	4
673	1	6	3	2	3	4
674	2	3	2	3	3	3
675	1	4	3	2	3	4
676	1	6	3	2	3	4
677	1	6	3	3	3	4
678	2	4	3	2	3	3
679	2	5	3	3	1	4
680	2	2	2	3	3	4
681	1	7	3	3	3	4
682	1	2	2	3	3	4
683	1	5	3	3	3	4
684	1	5	3	3	2	4
685	2	4	3	3	3	3
686	1	3	2	2	3	4
687	1	3	3	3	3	4
688	1	5	3	3	3	4
689	1	5	3	3	3	4
690	1	3	3	3	3	4
691	1	6	3	3	1	4
692	1	7	3	2	3	4
693	1	6	3	3	3	4
694	2	6	3	2	2	4
695	1	4	3	3	2	4
696	1	6	3	3	2	4
697	2	1	2	1	3	3
698	1	4	3	3	3	4
699	1	3	3	1	3	3
700	1	6	3	3	3	4
701	2	7	3	1	3	3
702	1	5	3	3	2	4
703	1	2	2	3	2	4
704	2	4	3	3	2	4
705	2	4	3	3	2	4
706	1	5	3	3	3	4
707	1	6	1	3	2	3
708	1	5	3	3	3	3
709	1	6	3	1	3	4
710	1	4	3	3	3	4
711	1	5	3	3	2	4
712	1	4	3	3	3	4
713	2	1	2	3	1	3
714	1	4	3	3	3	4
715	2	3	3	3	1	4
716	2	4	3	2	2	3
717	1	6	3	3	2	4
718	1	3	3	3	2	4
719	1	5	3	3	2	4

720	2	2	3	3	3	4
721	2	3	3	3	2	4
722	1	2	2	3	1	4
723	2	6	3	2	3	4
724	1	7	3	2	3	4
725	1	2	2	3	3	4
726	2	5	3	3	1	4
727	2	5	3	3	3	4
728	1	6	3	3	3	4
729	2	7	3	2	2	4
730	1	4	3	3	2	4
731	1	4	3	3	3	3
732	1	6	3	3	2	4
733	2	7	3	1	3	4
734	1	6	3	3	3	4
735	1	6	3	3	2	3
736	1	3	3	3	2	4
737	2	7	3	3	3	4
738	1	6	3	3	3	4
739	2	2	3	3	1	4
740	1	1	2	1	3	4
741	1	5	3	3	3	4
742	2	6	3	3	2	4
743	1	3	3	3	1	3
744	2	5	3	2	3	4
745	2	5	3	3	3	4
746	1	3	3	3	3	4
747	1	4	3	3	3	3
748	1	3	3	3	1	4
749	2	7	3	2	3	3
750	1	5	3	3	3	4
751	2	2	2	2	3	4
752	1	7	3	2	3	4
753	1	2	2	1	4	4
754	1	6	3	1	4	3
755	1	3	3	1	4	4
756	1	4	3	1	4	4
757	2	6	3	2	4	4
758	1	7	3	1	4	3
759	1	7	3	3	4	4
760	1	1	2	1	4	4
761	1	7	3	3	4	3
762	2	7	3	2	4	3
763	1	5	3	1	4	3
764	1	5	3	3	4	3
765	1	5	3	2	4	3
766	2	6	3	1	4	4
767	1	6	3	1	4	4

768	2	7	3	1	4	4
769	1	5	3	1	4	4
770	2	4	3	1	4	4
771	1	2	2	1	4	4
772	1	5	3	1	4	4
773	1	5	3	3	4	3
774	2	3	3	1	4	3
775	1	5	3	1	4	4
776	1	3	3	2	4	4
777	1	3	3	2	4	4
778	1	5	3	1	4	4
779	1	6	3	3	4	4
780	1	5	3	1	4	4
781	2	7	3	1	4	4
782	1	3	3	1	4	4
783	1	6	3	1	4	3
784	1	2	2	1	4	4
785	1	5	3	1	4	3
786	1	2	2	3	4	4
787	1	4	3	1	4	4
788	1	6	3	1	4	3
789	1	4	2	1	4	4
790	1	6	3	3	4	3
791	1	5	3	1	4	3
792	2	3	3	1	4	4
793	1	2	2	1	4	4
794	1	5	3	1	4	4
795	1	6	3	2	4	3
796	2	2	2	2	4	4
797	2	7	3	1	3	4
798	1	5	3	3	4	3
799	2	3	3	1	4	4
800	1	6	3	3	3	4
801	1	3	3	2	3	4
802	1	6	3	3	3	4
803	1	7	3	3	3	4
804	1	5	3	1	3	4
805	2	5	3	2	3	4
806	2	3	3	1	3	3
807	1	2	2	3	3	4
808	1	7	3	2	3	4
809	1	5	3	3	3	3
810	1	3	2	1	3	3
811	2	5	3	2	3	3
812	1	5	3	2	3	3
813	2	4	3	1	3	3
814	1	3	3	3	3	3
815	1	4	3	1	2	4

816	1	3	2	3	3	4
817	1	6	3	2	3	4
818	2	5	3	1	3	4
819	1	5	3	1	3	3
820	1	5	3	2	3	4
821	2	4	3	2	3	4
822	1	6	3	2	3	3
823	2	5	3	1	3	4
824	1	6	3	3	3	4
825	2	5	3	2	3	4
826	1	7	3	2	3	3
827	1	6	3	1	3	3
828	1	7	3	3	3	3
829	1	7	3	1	3	4
830	1	7	3	2	3	3
831	1	5	3	1	3	4
832	1	2	2	3	3	4
833	2	7	3	3	3	3
834	1	4	3	2	3	3
835	2	5	3	3	3	4
836	1	5	3	2	3	4
837	2	6	3	1	4	4
838	2	4	3	1	4	3
839	1	3	2	1	4	3
840	1	5	3	1	4	4
841	1	6	3	1	4	4
842	1	5	3	1	4	3
843	1	3	3	2	4	4
844	1	6	3	1	4	4
845	1	6	3	2	4	4
846	2	2	2	1	4	4
847	1	3	2	1	4	4
848	2	3	3	1	4	3
849	1	3	3	1	4	3
850	2	5	3	3	4	4
851	1	5	3	2	4	3
852	1	3	3	1	4	3
853	1	5	3	1	4	4
854	2	5	3	1	4	3
855	1	4	3	2	4	4
856	2	1	2	1	4	3
857	1	3	3	1	4	3
858	2	6	3	2	4	4
859	1	5	3	1	4	2
860	1	4	3	1	4	3
861	1	5	3	1	4	3
862	1	6	3	1	4	3
863	1	3	2	1	4	3

864	1	5	3	1	4	3
865	2	4	3	1	4	3
866	2	5	3	2	4	4
867	1	2	2	1	4	3
868	2	6	3	1	4	3
869	1	3	3	1	4	3
870	1	6	3	2	4	4
871	1	5	3	2	4	3
872	2	5	3	1	4	4
873	1	6	3	1	4	3
874	1	2	2	1	4	4
875	1	5	3	1	4	3
876	1	6	3	1	4	4
877	1	1	2	1	4	4
878	2	7	3	1	4	2
879	2	6	3	2	4	4
880	1	3	3	1	4	3
881	1	4	3	1	4	3
882	1	2	2	1	4	3
883	2	6	3	1	4	4
884	2	7	3	1	4	4
885	2	5	3	2	4	4
886	2	3	3	1	4	3
887	2	4	3	1	4	4
888	1	3	3	1	4	4
889	1	3	3	2	4	4
890	2	5	3	1	4	3
891	2	5	3	1	4	3
892	1	7	3	2	4	4
893	1	5	3	2	4	4
894	1	6	3	2	4	4
895	2	3	3	3	3	4
896	1	7	3	3	2	3
897	1	5	3	3	3	3
898	1	4	3	2	3	4
899	2	7	3	2	3	3
900	2	6	3	3	3	3
901	1	6	3	1	3	4
902	1	2	2	2	3	4
903	1	6	3	3	3	3
904	1	5	3	2	3	3
905	2	5	3	3	3	3
906	1	6	3	2	3	4
907	2	5	3	2	3	3
908	1	4	3	2	2	3
909	1	6	3	2	3	4
910	1	3	2	3	3	3
911	1	4	3	2	3	3

912	1	4	3	2	3	4
913	1	3	3	2	3	4
914	2	6	3	2	3	4
915	1	6	3	3	3	3
916	2	4	3	3	3	3
917	1	4	3	2	3	4
918	1	6	3	2	2	4
919	1	5	3	1	3	4
920	2	6	3	3	3	3
921	2	6	3	3	3	4
922	1	5	3	3	3	3
923	1	2	2	3	3	4
924	1	6	3	1	3	3
925	2	7	3	2	3	3
926	1	6	3	2	3	4
927	1	6	3	3	3	4
928	1	4	3	3	3	4
929	2	5	3	2	3	4
930	2	5	3	3	3	3
931	1	4	3	2	3	4
932	1	5	3	1	4	3
933	2	1	2	1	4	3
934	1	7	3	2	4	3
935	1	3	2	1	4	3
936	1	4	3	2	4	4
937	1	7	3	1	4	2
938	1	6	3	2	4	3
939	1	4	3	1	4	4
940	1	4	3	1	4	4
941	1	7	3	1	4	3
942	1	6	3	1	4	4
943	1	6	3	2	4	3
944	2	5	3	1	4	4
945	1	2	2	2	4	4
946	1	6	3	2	4	4
947	1	2	2	1	4	4
948	1	5	3	2	4	4
949	1	6	3	1	4	3
950	1	4	3	2	4	2
951	1	4	3	2	4	4
952	2	6	3	1	4	2
953	1	3	3	2	4	3
954	1	6	3	2	4	3
955	1	5	3	1	4	4
956	1	2	3	1	4	4
957	1	7	3	1	4	3
958	2	6	3	3	4	3
959	1	4	3	1	4	3

960	1	4	3	1	4	2
961	1	4	3	1	4	4
962	1	5	3	1	4	3
963	2	6	3	2	4	4
964	1	5	3	1	4	3
965	1	3	3	2	4	3
966	1	4	3	1	4	4
967	1	7	3	2	4	4
968	2	5	3	2	4	4
969	1	4	3	2	4	4
970	1	6	3	2	4	4
971	2	6	3	2	4	4
972	1	6	3	2	4	3
973	1	5	3	2	4	1

LOS	Number of body regions	Head	Thorax	Abdomen	Upper extremity
3	4	1	1	0	1
3	2	1	1	0	0
3	3	0	1	0	1
4	2	1	0	0	0
2	2	1	0	0	1
5	3	1	1	0	0
2	2	1	0	0	1
4	2	1	0	0	0
5	4	1	1	1	0
4	4	1	1	0	1
4	3	1	1	0	1
4	3	1	0	0	1
4	3	1	1	1	0
4	3	1	1	0	1
1	2	1	1	0	0
3	2	1	0	0	1
4	3	1	0	0	1
4	2	1	1	0	0
4	2	1	1	0	0
5	3	1	1	0	0
3	2	1	0	0	0
4	2	0	0	0	1
4	3	1	1	0	0
2	3	1	1	0	1
3	2	1	0	0	0
5	3	0	1	1	1
3	2	1	1	0	0
3	3	1	0	0	1
3	3	1	1	0	0
4	2	1	0	0	0
2	3	1	1	1	0
5	6	0	1	1	1
4	2	1	1	0	0
5	4	1	1	0	1
4	3	1	0	0	1
4	3	1	0	0	0
4	3	1	0	0	0
1	2	0	1	1	0
4	3	1	0	0	1
2	3	0	1	0	0
3	2	1	1	0	0
5	3	1	1	0	0
4	3	1	1	0	1
3	5	1	1	1	1
1	2	1	0	0	1
4	3	1	0	0	1
4	3	1	0	0	1

4	3	1	0	0	1
4	3	1	1	0	0
4	2	1	1	0	0
3	3	1	1	0	0
3	4	0	1	1	1
4	5	1	1	0	0
5	5	1	1	0	1
5	5	1	1	0	1
4	3	1	1	0	0
2	3	1	1	0	1
4	3	1	1	0	1
3	3	1	1	0	1
4	4	1	1	0	0
5	4	1	1	1	0
3	3	1	0	0	1
5	3	1	1	0	0
5	4	1	1	0	0
3	5	1	1	0	1
4	3	0	0	0	1
2	3	1	1	1	0
4	3	1	1	1	0
4	3	1	0	0	1
4	3	1	1	0	0
4	2	1	0	0	0
2	3	0	1	0	0
3	3	1	1	0	0
5	3	1	1	0	0
3	4	1	1	0	1
2	3	1	1	0	0
4	3	1	1	1	0
2	4	1	1	0	1
2	3	0	1	1	0
1	5	0	1	1	1
5	3	1	1	0	0
4	2	1	1	0	0
4	3	1	0	0	0
4	3	1	1	0	0
4	3	1	1	0	1
4	2	1	1	0	0
4	4	1	1	0	1
2	2	1	0	0	1
4	3	1	1	0	1
2	2	1	0	0	1
4	3	1	0	0	0
4	2	1	1	0	0
4	4	1	1	0	1
4	3	1	0	0	1
4	3	1	1	0	1

3	2	1	0	0	1
5	3	1	1	1	0
4	3	1	1	0	1
4	4	1	1	0	0
4	3	1	1	0	1
5	3	0	1	1	1
3	3	1	1	0	0
4	3	1	1	0	1
4	3	1	1	0	1
5	3	1	0	0	1
1	2	1	0	0	1
5	3	1	0	0	1
3	3	1	1	0	1
3	2	1	0	0	1
3	2	0	1	0	1
5	4	1	1	1	1
3	3	1	0	0	1
4	4	1	1	1	0
5	5	1	1	0	1
4	4	1	1	0	1
4	2	1	1	0	0
3	2	1	1	0	0
4	4	1	1	0	1
4	3	1	1	0	1
2	4	1	1	0	1
5	2	0	0	0	1
3	2	1	0	0	1
5	2	0	1	0	0
5	3	0	0	0	0
1	2	1	0	0	1
4	2	0	0	0	1
5	2	1	0	0	0
4	2	1	0	0	1
4	2	0	0	0	1
5	2	1	0	0	1
4	2	1	0	0	0
4	2	0	1	0	0
4	2	0	0	0	0
4	2	0	0	0	1
4	2	0	0	0	1
2	4	1	1	1	0
4	2	1	0	0	0
3	3	1	1	0	1
4	2	0	1	0	0
4	2	1	0	0	1
4	2	1	0	0	0
4	2	0	0	0	1
2	2	1	0	0	0

4	2	1	1	0	0
2	2	1	1	0	0
4	2	1	1	0	0
3	2	1	0	0	1
5	2	1	0	0	1
1	2	1	0	0	1
3	2	1	0	0	0
1	2	1	1	0	0
2	2	1	1	0	0
4	2	1	0	0	0
3	2	1	0	0	0
4	2	1	0	0	0
2	2	0	1	1	0
3	2	1	0	0	1
3	2	1	0	1	0
3	2	1	1	0	0
5	2	0	0	0	1
3	2	1	0	0	0
3	2	1	0	0	0
3	2	1	0	0	0
2	2	0	1	1	0
2	2	0	0	0	1
4	2	0	0	0	0
4	2	1	0	0	1
3	3	1	0	0	1
2	2	1	0	0	0
4	2	1	1	0	0
4	3	1	1	0	1
3	2	1	0	0	1
4	2	1	1	0	0
4	2	1	1	0	0
3	2	0	1	0	1
5	4	0	1	1	0
1	2	0	0	0	1
4	2	0	0	0	1
3	2	0	1	0	0
3	2	0	1	0	0
3	2	1	0	0	1
2	2	1	1	0	0
4	2	0	0	0	1
2	2	0	0	0	1
3	2	0	0	0	1
4	2	0	0	0	1
4	2	0	1	0	1
5	3	1	1	1	0
4	2	0	0	0	0
3	2	0	0	0	1
3	2	1	1	0	0

4	2	0	1	0	0
3	2	1	0	0	1
4	2	0	1	0	0
4	2	0	1	0	1
4	2	0	1	0	1
4	2	0	1	0	1
4	2	1	0	0	0
4	2	0	0	0	1
3	2	0	1	0	1
3	2	0	1	0	1
3	2	0	1	0	1
4	2	1	1	0	0
4	2	1	1	0	0
4	2	0	1	0	1
4	2	1	0	0	0
4	2	1	0	0	0
3	2	0	0	1	0
4	2	0	0	0	1
5	2	0	1	0	1
3	2	1	0	0	0
2	2	1	1	0	0
3	2	1	0	0	0
3	2	1	1	0	0
4	2	1	0	0	0
4	2	1	1	0	0
3	2	1	0	0	0
2	2	1	0	0	0
1	2	1	1	0	0
3	3	1	0	0	1
4	3	0	1	0	0
3	2	0	1	0	1
2	2	1	0	0	1
3	2	0	1	0	1
2	2	0	1	0	1
2	2	0	0	0	1
4	2	1	0	0	0
1	3	1	0	0	1
3	3	0	1	1	0
4	2	1	0	0	1
4	2	0	1	1	0
5	2	1	1	0	0
5	2	0	1	1	0
3	2	1	1	0	0
4	2	1	0	0	1
4	2	1	1	0	0
3	2	1	0	0	1
2	2	1	0	0	1
4	2	0	1	0	1

4	2	1	1	0	0
5	2	1	1	0	0
4	2	1	1	0	0
4	3	1	0	0	0
4	2	1	1	0	0
3	2	1	1	0	0
3	3	1	1	0	1
4	2	0	1	0	1
1	2	1	0	0	0
3	2	0	1	0	1
4	2	1	1	0	0
4	2	1	1	0	0
3	2	1	1	0	0
4	2	1	0	0	0
4	2	0	0	1	1
3	2	0	1	0	0
3	2	0	1	0	1
3	2	0	1	0	1
3	3	1	1	0	0
4	2	0	1	0	1
4	2	1	0	0	0
5	2	1	1	0	0
3	2	0	1	0	1
1	2	0	0	0	1
4	2	1	0	0	0
5	2	1	1	0	0
3	2	0	1	0	1
1	3	1	1	1	0
4	4	1	1	1	0
2	2	1	0	0	0
4	3	0	1	1	0
4	2	0	0	0	0
4	4	0	0	1	1
3	2	0	0	0	0
3	2	1	0	0	1
3	2	0	0	0	0
1	2	1	0	0	0
3	2	1	1	0	0
4	2	0	1	0	0
4	2	0	0	0	1
3	4	1	0	0	1
3	4	0	1	1	0
1	3	1	0	0	1
5	2	0	1	0	0
4	4	1	1	0	1
5	3	0	1	0	1
3	3	1	1	0	0
3	3	1	1	0	1

4	3	0	1	0	1
5	5	1	1	0	1
2	2	1	0	0	1
3	2	1	0	0	1
4	2	1	1	0	0
4	3	1	1	0	0
3	2	1	0	0	1
2	3	1	1	0	1
2	3	1	1	0	0
4	5	0	0	1	1
5	3	1	1	0	0
2	3	1	1	0	0
3	3	0	1	0	1
5	3	1	0	0	1
4	2	1	1	0	0
4	3	1	0	0	1
5	3	0	1	0	0
4	4	1	1	0	1
5	3	0	1	1	1
3	3	1	1	0	0
3	3	1	0	0	0
5	4	1	1	0	0
3	2	1	1	0	0
4	3	1	0	0	0
5	2	1	0	0	0
5	2	1	0	1	0
2	2	1	1	0	0
3	2	0	1	1	0
3	3	1	1	1	0
2	2	1	0	0	0
5	2	1	1	0	0
3	2	1	0	0	1
3	3	0	1	1	0
4	2	0	0	0	0
4	2	1	1	0	0
4	3	1	0	0	0
4	2	1	1	0	0
1	3	1	1	0	0
2	2	1	0	0	0
4	2	1	1	0	0
5	2	1	1	0	0
5	2	1	0	0	0
2	2	0	1	0	1
4	2	1	0	0	1
5	2	1	0	0	1
2	2	1	1	0	0
4	2	1	1	0	0
3	3	1	0	0	0

4	5	1	1	1	0
1	3	1	1	0	0
3	2	1	0	0	0
4	3	0	1	1	0
4	4	1	1	0	1
5	3	0	1	1	1
3	3	1	1	0	0
1	2	1	1	0	0
3	4	1	1	0	1
4	3	1	0	0	0
4	4	1	1	0	1
5	3	1	1	0	0
3	2	1	0	0	0
3	2	1	0	0	1
3	3	1	0	0	1
2	2	1	1	0	0
1	3	1	0	0	1
3	3	1	0	0	1
2	2	1	0	0	0
4	2	1	1	0	0
2	3	1	1	1	0
2	3	1	1	1	0
4	2	1	0	0	1
3	2	1	0	0	0
2	2	1	0	0	1
3	2	1	0	0	0
2	3	1	0	0	0
3	2	1	0	1	0
4	2	1	1	0	0
4	2	0	1	0	0
3	2	0	0	0	1
3	2	1	0	0	1
3	2	0	0	0	1
3	2	0	0	0	1
2	2	1	0	0	1
4	2	0	0	0	1
1	2	1	1	0	0
3	2	1	0	0	0
4	2	1	1	0	0
1	2	0	0	0	1
4	2	1	0	0	0
2	2	1	0	0	0
3	2	0	0	0	1
3	2	1	0	0	0
2	2	1	0	0	0
3	2	1	0	0	0
4	2	0	0	0	0
2	2	0	0	0	1

4	2	1	0	0	1
3	4	1	1	1	0
3	3	1	0	0	1
4	3	1	1	0	0
5	3	1	1	0	1
4	5	1	1	1	1
4	4	1	0	0	0
5	6	0	1	1	1
3	3	1	0	0	1
4	5	1	1	0	0
1	3	0	1	1	0
2	3	1	1	0	0
4	4	1	1	1	0
4	4	1	1	1	0
4	5	1	1	1	0
4	4	1	1	0	0
5	6	1	1	1	1
3	4	0	1	1	1
2	3	1	0	0	0
3	3	0	1	0	1
3	3	0	0	1	1
4	3	1	1	1	0
4	4	1	1	0	1
4	2	1	0	0	1
4	3	0	1	1	0
3	3	0	1	0	1
5	3	0	1	0	1
2	2	1	1	0	0
1	2	1	0	0	0
3	3	0	1	0	0
3	2	0	0	0	1
2	2	0	1	0	0
3	2	1	0	0	1
2	2	0	0	0	0
4	2	0	1	0	0
4	2	1	1	0	0
2	2	0	0	0	1
1	2	1	1	0	0
1	2	0	1	0	1
4	2	0	1	0	1
3	2	1	0	0	0
2	2	1	0	0	0
2	2	0	0	0	1
3	2	1	1	0	0
3	3	1	0	0	0
3	2	0	0	0	1
3	2	1	1	0	0
2	2	1	0	0	0

2	3	1	0	0	1
3	2	0	1	0	1
3	2	1	0	0	0
5	2	1	1	0	0
2	2	0	0	0	0
3	2	1	0	0	0
3	2	0	0	0	0
2	2	1	0	0	1
4	2	0	1	0	1
1	3	1	0	1	0
3	2	1	0	1	0
2	2	0	0	0	0
4	2	0	0	1	0
5	2	1	0	0	0
2	2	0	0	1	0
1	2	1	0	0	1
3	2	0	0	0	0
3	5	1	1	1	1
3	3	1	1	1	0
4	3	1	1	0	0
1	5	1	1	1	1
5	4	1	1	0	1
3	5	1	1	0	1
2	2	1	0	0	0
3	3	1	1	0	0
2	4	1	1	1	0
3	3	1	0	0	1
3	4	0	1	1	1
2	3	1	1	0	1
3	4	1	1	0	1
4	3	0	1	1	1
2	2	1	1	0	0
3	3	1	0	0	1
3	2	1	0	0	0
3	3	1	0	1	1
3	3	1	0	0	1
3	3	1	1	0	1
3	3	1	1	0	1
4	4	1	0	0	1
3	3	1	0	0	0
3	3	1	0	0	1
3	2	1	0	0	1
4	2	1	0	0	0
3	2	0	0	0	0
3	2	1	1	0	0
3	2	1	0	0	0
3	2	1	0	0	1
2	2	1	1	0	0

2	2	0	0	0	1
3	2	0	0	0	1
3	2	1	0	0	0
1	2	0	1	0	0
3	2	1	0	0	1
4	2	0	0	0	1
2	2	1	0	0	1
4	4	1	1	0	0
3	2	0	0	0	0
4	2	1	0	0	0
1	3	0	0	0	1
3	2	0	1	0	1
3	2	0	1	0	0
2	2	1	0	0	0
3	2	1	1	0	0
2	2	0	1	0	1
4	2	1	0	0	0
3	2	1	1	0	0
3	2	1	0	0	0
3	2	1	0	0	0
3	3	0	0	0	1
2	2	1	0	0	0
3	2	1	0	0	0
4	3	1	1	0	0
2	2	1	1	0	0
5	3	0	0	1	0
3	2	0	0	1	0
4	4	1	0	1	0
2	4	1	1	1	0
3	4	1	1	0	0
5	3	1	1	1	0
3	3	0	1	1	0
4	4	0	1	1	0
4	5	1	0	1	0
4	5	1	1	1	1
4	6	1	1	1	1
4	3	0	1	1	0
4	5	1	1	1	0
3	2	1	1	0	0
3	2	1	1	0	0
4	5	1	1	1	1
2	5	1	1	1	1
4	3	0	0	0	0
4	5	1	1	0	1
4	4	1	1	0	1
4	4	1	1	0	1
4	2	1	1	0	0
3	5	1	1	0	1

4	3	1	1	1	0
4	5	1	1	1	1
4	3	0	1	1	0
4	2	1	1	0	0
3	3	1	0	0	1
3	3	0	1	0	1
4	3	1	1	0	0
4	3	0	1	0	0
2	3	1	1	0	0
4	4	1	1	0	0
4	3	1	1	0	1
4	3	0	0	1	0
3	3	1	1	0	0
4	3	1	1	0	1
3	3	1	0	0	0
4	3	1	0	0	1
3	4	1	1	0	1
4	2	1	0	0	0
2	3	1	0	0	0
2	3	1	1	0	1
3	2	0	0	0	0
4	2	1	0	0	0
3	3	1	0	1	0
2	4	1	1	1	0
4	2	1	0	0	0
2	3	1	0	0	0
4	3	1	1	0	0
3	3	1	0	0	1
4	2	1	1	0	0
4	3	0	1	0	1
3	2	1	0	0	0
3	2	1	0	0	0
2	2	1	0	0	0
5	2	0	1	1	0
3	2	0	1	0	0
4	2	0	1	1	0
3	2	1	0	0	0
4	2	1	1	0	0
4	2	1	1	0	0
4	2	0	1	0	0
2	2	1	0	0	1
3	2	0	1	0	0
2	2	0	1	0	0
3	2	0	1	0	1
2	2	0	0	0	0
2	3	1	0	0	1
3	2	0	0	0	1
2	2	0	0	0	0

2	2	0	1	0	1
4	2	1	0	0	0
2	2	0	1	0	0
4	2	1	1	0	0
5	3	0	0	1	0
5	2	1	0	1	0
3	2	0	1	0	1
3	2	0	0	1	1
3	2	0	1	1	0
4	2	0	1	0	0
3	2	0	1	0	1
3	2	1	0	0	0
3	2	0	1	0	1
2	2	0	1	0	0
2	3	1	1	0	0
2	2	0	0	0	0
1	3	1	0	1	0
2	2	0	0	0	1
1	2	0	0	0	1
3	2	1	0	0	1
3	2	0	0	0	1
4	3	1	1	1	0
3	2	0	1	0	0
4	2	1	0	0	0
3	3	1	0	0	1
2	3	1	1	0	0
2	3	1	0	0	1
3	2	1	0	0	0
2	2	0	0	0	1
3	2	1	0	0	0
1	3	0	1	0	1
2	2	1	0	0	0
3	3	0	1	0	1
2	3	1	0	0	1
3	2	1	0	0	1
2	2	1	0	0	0
2	2	0	0	0	1
3	2	1	0	0	1
2	2	1	0	0	1
3	2	1	0	0	0
2	2	0	1	0	0
3	4	0	1	1	0
3	3	0	1	1	1
2	5	1	0	0	1
3	2	1	0	0	0
3	2	1	0	0	1
2	4	1	1	0	1
3	2	0	1	0	0

3	2	1	1	0	0
4	6	1	1	1	1
4	3	1	0	0	1
5	2	1	0	0	0
3	2	1	0	0	0
4	3	1	1	0	0
2	2	0	1	0	1
4	2	0	1	0	0
2	2	0	0	0	1
1	2	0	0	0	1
3	2	0	1	0	1
2	2	1	0	0	0
5	2	1	1	0	0
5	2	1	0	0	0
4	6	1	1	1	1
5	3	1	0	0	0
4	4	1	1	0	1
5	3	0	1	0	0
5	5	1	1	1	0
3	3	1	1	1	0
2	3	1	1	0	1
4	4	0	1	0	1
4	2	1	1	0	0
5	4	1	0	0	1
5	5	1	1	0	0
5	3	1	0	0	1
4	6	1	1	0	1
5	3	1	0	0	1
4	3	1	0	0	1
5	5	1	0	1	1
5	2	0	0	0	1
4	4	1	1	0	0
5	3	1	1	0	1
4	3	1	1	1	0
5	3	0	0	0	1
5	3	1	0	0	0
5	2	1	0	0	0
4	5	0	1	1	1
5	3	1	1	0	0
5	3	0	1	0	0
4	5	0	1	1	0
5	6	1	1	1	1
5	3	0	1	1	0
5	2	1	0	0	1
4	4	1	1	0	1
5	5	0	1	1	0
5	3	1	1	0	1
5	4	0	1	1	0

5	2	0	1	0	1
3	4	1	1	0	0
2	2	1	1	0	0
5	5	0	1	1	0
5	3	1	1	0	0
5	3	1	1	0	0
5	3	1	1	0	0
5	3	0	0	1	0
5	4	0	0	0	1
4	3	1	1	0	1
4	4	1	1	1	0
5	4	1	1	0	0
5	3	1	1	1	0
4	2	0	1	0	0
5	3	1	1	0	0
5	3	1	0	0	0
5	4	0	1	0	0
5	2	0	1	0	0
3	3	1	1	0	0
5	3	1	1	0	1
5	5	1	1	1	1
5	4	1	1	1	0
5	3	1	1	0	1
5	4	1	1	0	1
5	4	0	1	0	1
5	4	0	1	1	1
5	3	1	1	0	1
5	2	1	0	0	0
5	4	1	1	0	0
4	4	1	1	1	1
4	5	1	1	0	0
3	2	1	0	0	1
5	3	1	0	0	1
5	2	0	0	1	0
4	2	1	0	0	0
5	2	1	1	0	0
3	2	0	1	0	0
4	2	1	0	0	0
4	3	0	0	0	1
5	2	0	1	1	0
5	2	0	0	1	0
5	2	0	0	0	0
5	2	1	0	0	0
5	3	1	0	0	1
5	2	0	0	0	1
4	2	0	1	0	0
4	2	1	0	0	0
5	2	0	0	0	1

5	4	0	1	0	0
5	2	0	0	0	0
2	2	1	0	0	1
5	2	1	0	0	0
4	2	1	0	0	0
4	2	1	0	0	0
5	2	0	0	0	1
5	2	0	0	0	1
3	2	1	0	0	0
4	2	0	0	0	1
4	2	1	0	0	0
3	2	0	1	1	0
4	2	1	0	0	0
4	2	0	0	0	1
2	2	1	0	0	0
4	2	0	1	0	0
5	2	0	0	1	0
5	3	0	0	0	1
5	3	0	1	0	1
5	2	0	0	0	1
3	2	1	0	0	1
5	2	0	0	0	0
4	4	1	1	0	0
4	2	1	0	0	0
3	3	1	1	1	0
5	2	1	0	0	1
5	2	1	1	0	0
5	3	0	1	1	1
5	6	1	1	1	0
5	6	1	1	1	1
5	5	1	1	1	0
5	2	0	0	0	0
4	2	0	1	0	0
5	4	1	0	1	0
5	2	0	1	0	0
5	3	0	1	1	0
5	3	1	0	0	0
5	4	0	0	1	1
4	4	1	1	1	0
4	3	1	1	1	0
3	4	1	0	1	1
5	3	1	1	1	0
5	3	1	1	0	0
2	2	1	0	0	1
2	3	1	1	1	0
5	5	1	1	0	1
5	4	1	1	0	1
4	3	0	1	0	1

5	6	1	1	1	1
5	3	0	1	0	0
5	4	0	1	0	1
3	3	1	1	0	0
5	3	1	0	1	0
5	4	0	1	1	0
5	4	1	1	1	0
5	3	0	0	1	0
5	4	0	0	1	0
5	3	0	1	1	0
5	3	0	1	0	1
5	3	1	0	0	1
5	3	1	0	0	0
5	3	1	1	0	1
4	4	1	1	0	1
5	2	1	0	0	0
4	2	0	0	0	1
5	2	0	0	0	1
3	3	1	0	0	1
5	3	0	0	1	0
5	3	1	0	0	0
5	3	0	0	0	0
5	2	1	0	0	0
5	2	0	0	0	0
4	2	1	0	0	0
5	3	0	0	1	0
4	3	1	0	0	0
5	2	0	1	0	0
5	2	0	0	0	0
4	2	1	0	0	0
3	3	1	1	1	0
5	2	1	0	0	0
3	2	1	0	0	1
5	4	1	0	1	1
5	2	1	0	0	0
4	2	0	0	0	1
3	2	1	0	0	1
5	4	0	1	0	1
3	3	1	1	0	0
3	2	0	0	0	1
3	2	1	1	0	0
3	2	1	0	0	0
2	2	0	0	0	1
4	3	1	1	0	1
3	2	1	0	0	0
3	3	0	1	0	1
4	4	1	0	0	1
4	2	1	1	0	0

2	3	1	1	0	0
2	2	1	0	0	0
2	2	1	0	0	0
4	4	1	1	1	0
2	2	1	1	0	0
2	2	1	0	0	0
4	3	1	1	0	1
3	2	0	1	0	0
2	2	1	0	0	0
4	2	1	1	0	0
4	2	1	0	0	1
3	4	1	1	0	0
4	2	0	1	0	1
3	2	1	0	0	1
4	3	0	1	0	1
2	2	0	1	0	0
1	2	1	0	0	1
1	2	1	0	0	1
4	3	1	1	0	0
5	2	0	1	1	0
5	3	1	1	0	1
5	5	1	1	0	1
4	5	1	1	1	0
5	2	0	0	1	0
5	4	1	1	0	1
4	3	1	1	0	1
3	2	0	1	0	0
5	3	0	0	1	0
4	2	1	0	0	1
3	3	0	0	1	0
3	2	1	0	0	0
5	3	1	1	0	0
4	2	1	1	0	0
3	6	1	1	1	1
4	4	0	1	0	1
4	5	1	1	1	1
3	4	1	1	0	1
4	3	1	1	0	0
4	4	1	1	0	1
2	2	1	0	0	0
3	3	0	0	0	0
4	3	1	0	0	1
2	2	1	0	0	0
1	2	0	0	0	1
2	3	1	1	0	0
5	2	1	1	0	0
3	2	1	0	0	0
3	2	1	1	0	0

3	4	1	0	0	1
4	4	1	1	0	1
3	2	1	0	0	0
2	4	1	1	0	1
2	3	0	1	0	1
4	2	0	1	0	0
3	2	1	0	0	0
4	2	1	0	1	0
2	2	0	1	0	0
2	2	1	0	0	0
4	2	1	0	0	0
2	2	0	1	0	0
2	2	1	0	0	0
4	4	1	1	0	1
1	2	1	1	0	0
3	3	1	0	0	1
2	3	1	1	0	0
2	2	0	0	0	0
3	3	0	1	1	0
3	2	0	1	0	0
4	4	0	1	0	1
3	4	0	1	0	1
4	2	1	0	0	0
2	3	1	0	0	1
2	2	1	0	0	0
4	4	1	1	0	1
4	2	1	0	0	0
4	3	0	0	1	1
4	2	1	0	0	0
4	2	1	1	0	0
4	4	1	1	0	1
5	3	1	1	0	1
4	3	1	1	0	1
3	2	1	0	0	0
2	2	1	1	0	0
3	2	1	0	0	0
4	3	1	1	0	0
1	5	1	0	1	0
4	3	1	0	0	1
4	6	1	1	1	1
4	3	1	1	0	1
5	4	1	1	0	1
5	3	0	1	0	1
5	3	0	1	1	1
2	3	1	1	0	1
2	2	1	1	0	0
4	3	1	0	0	1
4	3	1	1	0	1

5	2	1	0	0	0
3	2	1	0	0	0
3	3	1	0	0	1
4	3	1	1	0	1
2	3	1	0	0	1
3	2	1	1	0	0
2	2	1	0	0	0
3	2	1	0	0	0
3	3	1	1	0	1
3	2	0	0	0	1
2	2	1	0	0	0
4	3	1	0	0	0
4	2	1	0	0	0
2	2	0	0	0	1
2	2	1	0	0	0
3	3	1	0	0	1
4	2	0	0	0	1
4	2	1	0	0	1
4	3	1	0	0	1
3	2	1	0	0	1
4	2	1	1	0	0
3	3	1	0	0	1
5	5	1	1	1	0
4	4	1	1	0	1
4	4	1	1	0	1
3	3	1	1	0	0
3	3	1	1	0	1
4	3	0	1	0	1
5	2	0	1	0	0
3	3	1	0	1	0
4	4	1	1	0	0
4	4	1	0	0	1
3	3	1	1	0	1
4	4	1	1	0	1
2	2	1	1	0	0
4	4	0	0	1	1
2	4	0	1	0	1
4	3	1	1	0	1
2	3	1	0	0	1
5	4	0	1	1	0
5	3	1	1	0	1
1	3	1	0	0	1
3	2	1	0	0	0
1	3	1	1	0	0
2	3	1	0	0	0
5	4	1	1	1	1
2	4	1	1	0	1
3	3	1	0	0	0

1	4	1	1	1	0
5	4	1	0	0	1
3	2	1	0	0	0
2	2	1	0	0	0
2	2	0	1	0	0
3	2	1	0	0	1
4	2	1	0	0	0
2	2	1	0	0	0
3	2	0	1	0	1
4	2	1	0	0	0
3	2	1	0	0	1
3	2	1	1	0	0
2	3	0	1	0	0
4	7	1	1	1	1

Lower extremity	Spine	Pelvis	FJI	SSTI	Destruction	CNSI	PC
1	0	0	1	1	0	0	1
0	0	0	1	1	0	1	0
1	0	0	1	1	0	0	0
1	0	0	1	1	0	0	0
0	0	0	1	1	0	0	0
1	0	0	1	1	0	1	1
0	0	0	1	1	0	1	0
0	0	1	1	1	0	0	0
0	0	1	1	0	0	1	1
1	0	0	1	0	0	1	1
0	0	0	1	1	0	1	0
1	0	0	1	1	0	1	0
0	0	0	0	1	0	0	0
0	0	0	1	1	0	0	1
0	0	0	0	1	0	0	0
0	0	0	1	1	0	0	0
0	1	0	1	1	0	1	0
0	0	0	1	1	0	1	1
0	0	0	1	1	0	1	0
1	0	0	1	0	0	1	1
1	0	0	0	1	0	1	0
1	0	0	1	1	0	0	0
0	0	1	1	1	0	0	1
0	0	0	1	1	0	0	1
1	0	0	1	1	0	1	0
0	0	0	1	1	0	0	1
0	0	0	1	1	0	1	0
1	0	0	0	1	0	1	0
1	0	0	1	1	0	1	0
1	0	0	1	1	0	1	0
0	0	0	1	1	0	1	1
1	1	1	1	1	0	0	1
0	0	0	1	1	0	1	1
1	0	0	1	1	0	1	1
0	0	1	1	1	0	0	0
1	0	1	1	1	0	1	0
1	1	0	1	1	0	0	0
0	0	0	1	1	0	0	1
1	0	0	1	1	0	0	0
1	0	1	1	1	0	0	1
0	0	0	0	1	0	1	0
0	1	0	1	1	0	0	1
0	0	0	1	1	0	0	1
0	1	0	1	1	0	1	0
0	0	0	1	1	0	1	0
0	1	0	1	1	0	1	0
1	0	0	1	1	0	1	0

0	0	1	1	1	0	0	0
0	1	0	1	1	0	1	1
0	0	0	1	1	0	0	1
0	1	0	1	1	0	0	1
1	0	0	1	0	0	0	1
1	1	1	1	1	0	1	1
1	0	1	1	1	0	1	1
1	1	0	1	1	0	1	1
1	0	0	1	1	0	0	0
0	0	0	1	1	0	0	0
0	0	0	1	1	0	1	1
0	0	0	1	0	0	1	1
1	1	0	1	1	0	0	1
1	0	0	1	1	0	0	1
1	0	0	1	1	0	1	0
0	0	1	1	1	0	1	1
0	1	1	1	1	0	1	1
1	0	1	1	1	0	0	0
1	1	0	1	0	0	0	0
0	0	0	0	0	0	1	1
0	0	0	1	1	0	0	1
1	0	0	1	1	0	1	0
0	1	0	1	1	0	1	1
0	1	0	1	1	0	0	0
1	1	0	1	0	0	0	1
0	1	0	1	1	0	1	1
1	0	0	1	0	0	1	1
0	1	0	1	1	0	1	1
1	0	0	1	1	0	1	1
0	0	0	1	1	0	1	0
1	0	0	1	1	0	1	1
1	0	0	1	1	0	0	0
1	0	1	1	1	1	0	0
1	0	0	1	1	0	1	1
0	0	0	1	1	0	1	0
1	1	0	1	1	0	0	0
0	1	0	1	1	0	1	0
0	0	0	1	1	0	1	0
0	0	0	1	1	0	1	0
0	1	0	1	1	0	1	1
0	0	0	1	1	0	0	0
0	0	0	1	1	0	0	1
0	0	0	1	1	0	1	0
0	1	1	1	1	0	0	0
0	0	0	1	1	0	1	1
0	0	1	1	1	0	0	1
1	0	0	1	1	0	0	0
0	0	0	1	1	0	1	0

0	0	0	1	1	0	1	0
0	0	0	1	1	0	1	1
0	0	0	1	1	0	0	1
1	0	1	1	0	0	0	1
0	0	0	1	0	0	1	1
0	0	0	1	0	0	0	0
0	1	0	1	0	0	1	0
0	0	0	1	1	0	0	1
0	0	0	1	0	0	1	0
1	0	0	1	1	0	0	0
0	0	0	1	1	0	1	0
0	1	0	1	1	0	0	0
0	0	0	1	1	0	0	0
0	0	0	1	1	0	1	0
0	0	0	1	0	0	0	0
0	0	0	1	1	0	1	1
1	0	0	1	0	0	1	0
1	0	0	1	1	0	1	1
0	1	1	1	1	0	0	1
0	1	0	1	1	0	0	1
0	0	0	1	1	0	1	1
0	0	0	1	1	0	0	1
0	0	1	1	1	0	1	1
0	0	0	1	1	0	1	1
1	0	0	0	1	0	1	0
1	0	0	1	0	0	0	0
0	0	0	0	1	0	0	0
1	0	0	1	0	0	0	0
1	1	1	1	1	0	0	0
0	0	0	1	1	0	1	0
0	0	1	1	0	0	0	0
1	0	0	1	1	0	1	0
0	0	0	1	1	0	1	0
1	0	0	1	1	0	0	0
0	0	0	1	1	0	0	0
0	1	0	1	1	0	0	0
1	0	0	1	1	0	0	0
0	1	1	1	0	0	0	0
0	0	1	1	0	0	0	0
1	0	0	1	0	0	0	0
1	0	0	0	1	0	0	0
0	0	1	1	1	0	0	0
0	0	0	1	1	0	0	1
1	0	0	1	1	0	0	1
0	0	0	1	1	0	1	0
1	0	0	1	1	0	1	0
1	0	0	1	1	0	0	0
0	1	0	1	0	0	0	0

0	0	0	1	0	0	1	1
0	0	0	1	1	0	0	0
0	0	0	1	1	0	1	0
0	0	0	0	1	0	1	0
0	0	0	1	1	0	1	0
0	0	0	1	1	0	1	0
0	1	0	1	0	0	1	0
0	0	0	1	1	0	1	1
0	0	0	0	1	0	1	1
0	0	1	1	1	0	1	0
1	0	0	1	0	0	1	0
1	0	0	1	1	0	0	0
0	0	0	1	0	0	0	0
0	0	0	1	1	0	1	0
0	0	0	0	1	0	0	0
0	0	0	0	1	0	1	1
1	0	0	1	1	0	0	0
0	0	1	1	1	0	0	0
0	1	0	1	1	0	1	0
1	0	0	1	1	0	0	0
0	0	0	1	0	0	0	1
1	0	0	1	1	0	0	0
1	1	0	1	0	0	0	0
0	0	0	0	1	0	1	0
1	0	0	1	1	0	0	0
1	0	0	1	1	0	1	0
0	0	0	1	1	0	1	1
0	0	0	1	0	0	1	1
0	0	0	1	1	0	0	0
0	0	0	1	1	0	1	0
0	0	0	1	1	0	1	0
0	0	0	1	0	0	0	0
1	0	1	1	1	0	0	1
1	0	0	1	1	0	0	0
0	0	1	1	0	0	0	0
0	1	0	1	0	0	0	1
0	1	0	1	0	0	0	1
0	0	0	1	0	0	1	0
0	0	0	1	1	0	0	0
1	0	0	1	1	0	0	0
1	0	0	1	1	0	0	0
1	0	0	0	1	0	0	0
0	0	1	1	1	0	0	0
0	0	0	1	0	0	0	1
0	0	0	1	1	0	1	0
1	1	0	1	0	0	0	0
0	1	0	1	1	0	0	0
0	0	0	1	1	0	0	0

1	0	0	1	0	0	0	0
0	0	0	1	1	0	0	0
1	0	0	1	0	0	0	1
0	0	0	1	0	0	0	1
0	0	0	1	0	0	0	1
0	0	0	1	0	0	0	0
0	0	1	1	1	0	1	0
1	0	0	1	0	0	0	0
0	0	0	1	1	0	0	0
0	0	0	1	1	0	0	0
0	0	0	0	1	0	0	0
0	0	0	1	1	0	0	0
0	0	0	1	1	0	1	1
0	0	0	1	0	0	0	1
0	1	0	1	1	0	0	0
1	0	0	1	0	0	1	0
0	1	0	1	0	0	0	0
1	0	0	1	0	0	0	0
0	0	0	1	0	0	0	0
0	1	0	0	1	0	1	0
0	0	0	1	1	0	1	1
1	0	0	1	1	0	1	0
0	0	0	1	1	0	0	0
1	0	0	1	1	0	1	0
0	0	0	1	1	0	1	1
1	0	0	1	1	0	1	0
0	1	0	1	0	0	1	0
0	0	0	0	1	0	0	0
1	0	0	0	1	0	1	0
1	0	1	1	0	0	0	0
0	0	0	1	0	0	0	0
0	0	0	1	1	0	0	0
0	0	0	1	0	0	0	0
0	0	0	1	0	0	0	1
1	0	0	1	0	0	0	0
0	1	0	1	1	0	1	0
1	0	0	0	1	0	1	0
0	0	1	1	0	0	0	0
0	0	0	1	0	0	1	0
0	0	0	1	0	0	0	0
0	0	0	1	1	0	1	1
0	0	0	1	0	0	0	1
0	0	0	0	1	0	1	1
0	0	0	1	1	0	0	0
0	0	0	1	0	0	1	1
0	0	0	0	1	0	1	0
0	0	0	1	1	0	0	0
0	0	0	1	0	0	0	0

0	0	0	1	1	0	1	1
0	0	0	1	1	0	1	1
0	0	0	1	1	0	0	0
1	1	0	1	1	0	0	0
0	0	0	1	1	0	1	1
0	0	0	1	1	0	1	1
0	0	0	1	1	0	0	1
0	0	0	1	0	0	0	1
0	1	0	1	1	0	1	0
0	0	0	1	0	0	0	0
0	0	0	1	1	0	1	0
0	0	0	1	1	0	1	1
0	0	0	1	1	0	0	0
0	1	0	1	0	0	1	0
0	0	0	1	0	0	0	0
1	0	0	1	0	0	0	1
0	0	0	1	0	0	0	0
0	0	0	1	0	0	0	0
1	0	0	1	1	0	0	0
0	0	0	1	0	0	0	0
0	0	1	1	1	0	1	0
0	0	0	1	1	0	1	0
0	0	0	1	0	0	0	0
1	0	0	1	0	0	0	0
1	0	0	1	1	0	0	0
0	0	0	1	1	0	1	1
0	0	0	1	0	0	0	0
0	0	0	0	1	0	1	0
0	1	0	1	1	0	1	1
1	0	0	1	1	0	1	0
0	1	0	1	0	0	0	1
1	0	1	1	0	0	0	0
1	1	0	1	0	0	0	0
1	0	1	1	0	0	0	0
0	0	0	0	1	0	1	0
1	1	0	1	0	0	0	0
0	1	0	1	0	0	0	0
0	0	0	1	1	0	1	0
0	1	0	1	0	0	0	1
1	0	0	1	0	0	0	0
1	0	1	1	1	0	0	0
0	1	1	1	1	0	0	1
1	0	0	1	1	0	0	0
0	1	0	1	1	0	0	0
1	0	0	1	1	0	1	0
1	0	0	1	1	0	0	0
0	1	0	1	1	0	1	0
0	0	0	1	1	0	1	1

0	1	0	1	1	0	0	1
1	0	1	1	1	0	1	1
0	0	0	0	1	0	1	0
0	0	0	1	1	0	1	0
0	0	0	1	1	0	1	0
1	0	0	1	1	0	0	1
0	0	0	1	1	0	1	0
0	0	0	1	1	0	0	0
1	0	0	1	1	0	0	0
1	1	1	1	1	0	0	0
0	1	0	1	1	0	1	1
1	0	0	1	1	0	1	1
1	0	0	1	1	0	0	0
1	0	0	1	1	0	0	0
0	0	0	1	1	0	1	1
1	0	0	1	1	0	1	0
0	1	1	1	0	0	0	0
1	0	0	1	0	0	1	0
0	0	0	1	1	0	0	1
1	0	0	1	1	0	0	0
0	1	1	1	1	0	1	0
1	0	1	1	0	0	1	1
0	0	0	1	1	0	1	0
1	0	1	1	1	0	1	0
0	1	0	1	1	0	1	0
0	0	0	1	1	0	0	0
0	0	0	0	1	0	1	0
0	0	0	1	0	0	0	0
0	0	0	0	1	0	1	0
0	1	0	1	0	0	1	0
0	0	0	0	0	0	1	1
0	0	0	1	0	0	1	0
0	1	0	1	1	0	0	0
1	0	1	1	1	0	0	0
0	0	0	1	1	0	1	1
1	0	1	1	1	0	1	0
0	0	0	1	0	0	1	1
1	0	0	1	1	0	0	0
1	0	0	0	1	0	1	0
0	0	0	1	1	0	0	1
0	0	0	1	1	0	1	0
1	0	0	1	1	0	0	0
0	0	0	1	0	0	0	0
0	0	0	1	0	0	1	0
0	0	0	1	1	0	0	0
0	0	0	1	1	0	1	0
0	0	0	0	1	0	1	1
1	0	1	1	1	0	0	0

1	0	1	1	1	0	1	0
0	0	1	1	1	0	1	1
1	0	0	1	0	0	1	0
1	0	0	1	1	0	0	1
0	1	0	1	1	0	1	1
0	0	0	1	1	0	0	1
1	0	0	1	1	0	1	1
0	0	0	1	1	0	1	1
1	0	0	0	1	0	1	1
0	1	1	1	1	0	1	0
1	0	0	1	1	0	1	1
0	1	0	1	1	0	0	1
1	0	0	1	1	0	1	0
0	0	0	1	1	0	1	0
1	0	0	1	1	0	0	0
0	0	0	1	1	0	1	0
1	0	0	1	1	0	0	0
1	0	0	1	1	0	1	0
0	1	0	1	1	0	1	0
0	0	0	1	1	0	1	0
0	0	0	1	1	0	0	0
0	0	0	0	1	0	1	0
0	0	0	0	1	0	1	0
1	0	0	1	1	0	1	0
0	0	0	1	1	0	1	0
1	0	0	1	1	0	0	0
1	1	0	1	1	0	0	0
0	0	0	1	1	0	1	0
0	0	0	1	1	0	0	0
0	1	0	0	0	0	1	1
1	0	0	1	1	0	0	0
0	0	0	1	1	0	1	0
1	0	0	1	0	0	0	0
1	0	0	1	0	0	0	0
0	0	0	1	1	0	0	0
1	0	0	1	0	0	0	0
0	0	0	1	1	0	1	0
1	0	0	1	1	0	0	0
0	0	0	1	1	0	1	0
1	0	0	1	1	0	0	0
0	1	0	1	1	0	1	0
1	0	0	0	1	0	1	0
1	0	0	1	1	0	0	0
0	1	0	1	1	0	1	0
0	1	0	1	1	0	1	0
0	1	0	1	0	0	0	0
1	1	0	1	1	0	1	0
1	0	0	1	0	0	0	0

0	0	0	1	1	0	1	0
1	0	0	1	1	0	0	1
1	0	0	1	0	0	1	0
1	0	0	1	1	0	0	1
0	0	0	1	1	0	1	1
0	0	1	1	1	0	1	1
1	1	1	1	0	0	0	0
1	1	1	1	0	0	0	1
1	0	0	1	1	0	1	0
1	1	1	1	1	0	1	1
1	0	0	1	1	0	0	1
0	1	0	1	1	0	1	1
0	1	0	1	1	0	1	1
0	1	0	1	1	0	1	1
0	1	1	1	1	0	1	0
0	1	1	1	1	0	1	0
1	0	1	1	1	1	0	1
0	1	0	1	0	0	0	0
1	1	0	1	1	0	0	0
0	1	0	1	0	0	0	1
1	0	0	1	1	0	0	0
0	0	0	1	0	0	1	1
1	0	0	1	1	0	1	0
0	0	0	1	0	0	1	0
0	1	0	1	0	0	0	0
0	1	0	1	0	0	0	0
1	0	0	1	1	0	0	0
0	0	0	1	0	0	1	0
0	1	0	0	1	0	1	0
1	1	0	1	1	0	0	0
1	0	0	1	1	1	0	0
0	1	0	1	0	0	0	1
0	0	0	1	1	0	1	0
0	1	1	1	0	0	0	0
0	1	0	1	0	0	0	1
0	0	0	1	1	0	0	0
0	1	0	1	0	0	0	0
0	0	0	1	0	0	1	1
0	0	0	1	1	0	0	0
0	0	0	1	0	0	0	0
0	1	0	1	1	0	1	0
0	1	0	1	0	0	1	0
1	0	0	1	0	0	0	0
0	0	0	1	1	0	1	1
0	1	1	1	0	0	1	0
0	0	1	1	0	0	0	0
0	0	0	1	1	0	0	0
1	0	0	1	1	0	1	0

0	1	0	0	1	0	1	0
0	0	0	1	0	0	0	0
1	0	0	1	1	0	0	0
0	0	0	1	0	0	0	0
1	1	0	1	1	0	0	0
0	1	0	1	1	0	1	0
1	0	1	1	1	0	0	0
0	0	0	1	1	0	0	0
0	0	0	1	0	0	0	0
0	1	0	1	1	0	1	0
0	0	0	0	1	0	1	0
1	1	0	1	0	0	0	0
1	0	0	1	1	0	0	0
0	1	0	1	1	0	1	0
0	1	0	1	0	0	0	0
0	0	0	1	1	0	0	0
1	0	1	1	1	0	0	0
1	0	0	1	1	0	0	0
0	0	0	1	1	0	1	1
0	0	1	1	1	0	1	0
1	0	0	1	1	0	1	1
1	0	0	1	1	0	1	1
1	0	1	1	1	0	0	1
1	0	0	1	1	0	1	0
0	1	0	1	1	0	1	1
0	1	0	1	1	0	1	1
0	1	0	1	1	0	0	0
0	1	0	1	1	0	0	1
0	0	0	1	1	0	1	1
1	0	0	1	1	0	0	0
0	0	0	1	0	0	0	1
0	0	0	1	0	0	1	0
1	0	0	1	0	0	0	0
1	0	0	1	1	0	0	0
0	0	0	1	1	0	0	0
0	1	0	1	1	0	0	0
0	0	0	1	1	0	1	0
0	0	0	1	1	0	1	0
1	0	1	1	1	0	0	0
1	0	1	1	1	0	1	0
1	0	0	1	1	0	0	0
0	0	0	1	1	0	1	0
1	0	0	1	1	0	1	0
0	1	1	1	0	0	0	0
0	0	0	1	1	0	0	0
1	0	0	1	1	0	1	0
0	0	0	1	1	0	1	0
0	0	0	1	1	0	1	0

0	1	0	1	0	0	0	0
1	0	0	1	0	0	0	0
0	1	0	1	1	0	0	0
1	0	0	1	0	0	0	0
0	0	0	1	0	0	0	0
1	0	0	1	0	0	0	0
0	0	0	1	0	0	0	0
0	1	1	1	1	0	1	1
1	1	0	1	0	0	0	0
1	0	0	1	1	0	0	0
1	0	1	1	1	0	0	0
0	0	0	1	0	0	0	0
1	0	0	1	1	0	0	0
1	0	0	1	1	0	0	0
0	0	0	1	1	0	1	0
0	0	0	1	0	0	0	0
1	0	0	1	1	0	0	0
0	0	0	0	1	0	1	0
0	1	0	1	1	0	0	0
1	0	0	1	1	0	0	0
1	1	0	1	1	0	0	0
0	1	0	1	1	0	1	0
1	0	0	1	0	0	0	0
0	1	0	1	1	0	1	1
0	0	0	1	1	0	1	1
1	0	1	1	1	0	0	0
1	0	0	1	1	0	0	0
1	1	0	1	1	0	1	0
0	1	0	1	1	0	1	1
0	1	1	1	1	0	1	1
0	0	0	1	1	0	0	1
0	1	0	1	1	0	0	0
0	1	1	1	1	0	0	1
1	1	1	1	1	0	0	0
0	1	0	1	1	0	0	0
1	0	1	1	0	0	1	1
0	1	0	1	1	0	0	1
1	0	1	1	1	0	1	1
0	0	0	1	1	0	1	1
0	0	0	1	1	0	1	1
1	0	0	1	1	0	1	1
0	1	0	1	1	0	0	1
1	1	1	1	1	0	0	0
1	1	0	1	1	0	1	1
0	1	0	1	1	0	1	1
0	1	0	1	1	0	1	1
0	0	0	1	1	0	1	1
1	0	1	1	1	0	1	1

0	0	0	1	1	0	0	0
1	0	0	1	0	0	1	1
1	0	0	1	0	0	0	1
0	0	0	1	1	0	1	1
1	0	0	1	1	0	1	0
0	1	0	1	0	0	0	1
1	0	0	1	0	0	1	0
0	1	1	1	0	0	0	1
0	1	0	1	1	0	0	0
1	0	1	1	1	0	1	1
0	0	0	1	1	0	1	1
0	1	1	1	1	0	0	0
0	1	0	1	1	0	1	1
0	0	0	1	1	0	1	0
1	0	1	1	1	0	1	0
1	0	0	1	1	0	1	0
0	1	0	1	0	0	1	0
0	1	0	1	1	0	1	0
1	0	1	1	1	0	1	0
0	0	0	1	1	0	0	1
0	1	1	1	0	0	0	0
0	1	0	1	1	0	1	0
0	1	0	1	0	0	1	0
0	1	0	1	0	0	0	1
0	0	1	1	1	0	1	0
1	1	0	1	1	0	1	0
1	0	0	1	1	0	1	0
0	1	0	1	1	0	0	0
0	0	0	1	1	0	1	0
1	0	0	1	0	0	0	0
1	0	0	1	1	0	0	0
1	0	0	1	0	0	1	0
0	1	0	1	1	0	1	0
0	0	0	1	1	0	0	1
0	1	0	1	0	0	0	1
0	0	0	1	0	0	0	0
1	0	0	1	0	0	1	0
0	0	0	1	1	0	1	1
0	0	0	1	1	0	1	0
0	1	0	1	0	0	1	0
0	0	0	1	0	0	0	0
0	1	0	1	0	0	0	1
0	1	0	1	0	0	1	1
0	0	0	1	0	0	0	0
1	1	0	1	0	0	0	0
0	1	0	1	1	0	0	0
1	0	0	1	1	0	0	0
0	1	1	1	0	0	0	0

0	0	0	1	0	0	0	1
0	1	0	1	0	0	1	0
1	0	0	1	0	0	0	0
0	0	0	1	1	0	1	0
1	0	1	0	1	0	1	0
0	0	0	0	1	0	0	0
0	0	0	1	1	0	0	0
0	0	0	0	1	0	0	0
0	0	0	0	1	0	0	0
1	0	0	1	1	0	0	0
0	0	0	1	0	0	0	0
0	1	0	1	1	0	0	0
0	0	0	1	0	0	0	0
1	0	0	1	0	0	0	0
0	1	0	1	1	0	1	1
1	1	0	1	0	0	1	0
0	1	0	1	0	0	1	0
0	1	0	1	0	0	1	0
0	1	0	0	1	0	0	0
0	0	0	1	0	0	0	0
1	0	0	1	1	0	0	0
0	0	0	1	1	0	0	0
1	0	0	1	0	0	0	0
1	0	0	1	1	0	0	0
1	0	0	1	1	0	0	0
1	0	0	1	1	0	0	1
1	0	0	1	1	0	0	0
1	0	0	1	1	0	0	0
1	0	0	1	0	0	0	0
0	0	1	1	1	0	0	0
1	0	0	1	1	0	0	0
1	0	0	1	1	0	0	0
1	0	0	1	1	0	0	1
1	0	0	1	1	0	0	0
0	0	0	1	1	0	0	0
1	0	0	1	1	0	1	0
1	0	0	1	0	0	0	0
0	0	0	1	1	0	0	0
0	0	0	1	0	0	0	0
1	0	0	1	1	0	0	0
1	0	0	1	0	0	0	0
0	1	1	1	1	0	0	1
0	0	0	1	1	0	0	0
1	1	1	1	1	0	0	0
1	0	0	1	1	0	0	0
0	0	0	1	0	0	0	0
1	0	0	1	1	0	0	0
1	0	0	1	0	0	0	0

0	0	0	1	1	0	0	0
0	1	1	1	1	0	0	1
1	0	0	1	1	0	0	0
1	0	0	1	0	0	0	0
0	0	1	1	1	0	0	0
0	1	0	1	1	0	1	0
0	0	0	1	0	0	0	1
1	0	0	1	1	0	0	0
0	1	0	1	1	0	0	0
1	0	0	1	0	0	0	0
0	0	0	1	0	0	0	0
1	0	0	1	0	0	0	0
0	0	0	1	0	0	1	1
1	0	0	1	1	0	0	0
1	1	0	1	1	0	0	1
1	1	0	1	1	1	0	0
1	0	0	1	1	0	1	0
1	0	1	1	1	0	0	1
0	1	1	1	1	0	0	1
0	0	0	1	1	0	0	0
0	0	0	1	1	0	0	1
0	1	1	1	1	0	0	0
0	0	0	1	1	0	0	1
1	0	1	1	1	0	0	0
1	1	1	1	0	0	0	1
1	0	0	1	1	0	0	0
1	1	1	1	1	0	1	1
0	1	0	1	1	0	0	0
1	0	0	1	1	0	0	0
0	1	1	1	1	0	0	0
1	0	0	1	1	0	0	0
0	1	1	1	1	0	0	1
0	0	0	1	1	0	1	0
0	0	0	0	1	0	0	1
1	1	0	1	0	0	0	0
1	0	1	1	1	0	1	0
1	0	0	1	0	0	1	0
1	0	1	1	1	0	0	1
0	1	0	1	1	0	0	1
1	1	0	1	1	0	0	0
1	1	1	1	1	0	0	1
1	0	1	1	1	0	0	1
1	0	0	1	1	0	0	1
0	0	0	1	1	0	0	0
1	0	0	1	1	0	1	1
1	1	1	1	1	0	0	1
0	0	0	1	1	0	0	1
1	0	1	1	0	0	0	1

0	0	0	1	1	0	0	1
1	0	1	1	1	0	0	1
0	0	0	1	0	0	0	1
1	1	1	1	1	0	0	0
1	0	0	1	1	0	1	1
1	0	0	1	1	0	0	1
1	0	0	1	1	0	0	0
1	1	0	1	1	0	0	0
1	1	1	1	1	0	0	0
0	0	0	1	1	0	1	1
1	0	0	1	1	0	1	1
1	1	0	1	1	0	0	0
0	0	0	1	0	0	1	1
0	0	1	1	1	0	0	1
1	0	0	1	1	0	0	1
1	0	1	1	1	1	1	0
1	1	1	1	1	0	0	1
1	0	0	1	1	0	0	0
1	0	0	1	1	0	0	1
0	0	0	1	0	0	0	1
0	1	0	1	1	0	1	1
1	0	0	1	1	0	0	1
0	0	0	1	1	0	0	0
1	0	0	1	1	0	0	0
1	0	1	1	1	0	0	1
1	0	0	1	1	0	0	0
0	0	0	1	1	0	1	1
1	0	0	1	1	0	0	0
1	1	0	1	1	0	1	1
0	0	0	1	1	0	0	1
1	1	1	1	1	1	0	0
0	0	0	1	1	0	0	0
1	0	0	1	1	0	1	0
0	0	1	1	1	0	0	0
1	0	0	1	1	0	1	0
0	0	0	1	1	0	1	1
1	0	0	0	1	0	0	0
1	0	0	1	1	0	0	0
1	0	1	1	1	0	0	0
0	0	0	1	0	0	0	0
0	0	1	1	0	0	0	0
1	0	1	0	1	1	0	0
1	0	0	1	1	0	0	0
1	0	0	1	1	0	0	0
1	0	0	1	1	1	0	0
1	0	0	1	0	0	0	0
1	0	0	1	1	0	1	0
1	0	0	1	1	0	0	0

1	1	1	1	1	0	0	0
1	1	0	1	1	0	0	0
0	0	0	0	1	0	1	0
1	0	0	0	1	1	1	0
1	0	0	1	1	0	0	0
1	0	0	1	1	0	0	0
0	1	0	1	0	1	0	0
1	0	0	1	1	0	0	0
1	0	0	1	1	0	0	0
1	0	0	0	1	1	0	0
1	0	0	1	1	0	0	0
0	0	0	1	0	0	0	0
1	0	0	1	1	0	0	0
1	0	0	1	1	0	0	0
1	0	0	1	1	0	0	0
1	0	0	1	0	1	0	1
1	0	0	0	1	0	0	0
1	0	1	1	1	0	0	0
1	0	0	1	1	0	0	0
1	0	0	0	1	1	0	0
0	0	0	1	1	0	0	0
1	1	0	1	1	0	0	0
1	0	1	1	1	0	0	0
1	0	0	0	1	0	0	0
0	0	0	1	1	0	0	0
0	0	0	1	1	1	0	0
0	0	0	1	0	0	0	0
0	0	0	1	1	0	0	1
1	1	1	1	1	1	0	1
0	1	1	1	1	0	1	1
0	1	1	1	1	0	1	1
1	0	1	1	1	0	0	0
1	0	0	1	1	0	0	0
1	0	1	1	1	1	0	0
1	0	0	1	0	0	0	1
0	0	1	1	1	0	0	1
1	0	1	1	1	0	1	0
1	0	1	1	1	0	0	0
1	0	0	1	1	0	1	1
0	0	0	0	1	0	0	1
1	0	0	0	1	0	0	0
0	0	0	1	1	0	0	1
1	0	0	1	1	0	1	0
0	0	0	1	1	0	0	0
0	0	0	1	1	0	1	1
1	1	0	1	1	0	1	1
1	0	0	1	1	0	0	1
0	1	0	1	0	0	0	0

1	1	0	1	1	0	0	0
1	1	0	1	1	0	0	1
1	1	0	1	1	1	0	1
1	0	0	1	1	0	1	0
1	0	0	0	1	0	0	0
1	0	1	1	1	0	0	1
1	0	0	1	1	0	0	0
0	1	1	1	1	0	0	0
1	1	1	1	1	0	0	0
0	1	0	1	1	0	0	1
1	0	0	1	0	0	0	0
1	0	0	1	1	0	0	0
1	0	1	1	1	0	0	0
0	0	0	1	1	0	1	1
1	0	0	1	1	1	0	1
1	0	0	0	0	1	1	0
1	0	0	1	1	0	0	0
1	0	0	1	1	0	0	0
1	0	0	1	1	0	0	0
1	0	1	1	1	1	0	0
1	1	0	1	1	0	0	0
1	1	1	1	1	0	0	0
1	0	0	1	1	0	1	0
1	0	1	1	1	0	0	0
1	0	0	1	1	0	0	0
1	0	1	1	1	0	0	0
1	0	1	1	1	0	0	0
1	0	0	1	1	0	0	0
1	0	1	1	1	0	0	0
0	1	0	1	1	0	0	0
0	0	0	1	1	0	0	1
1	0	0	1	1	0	0	0
0	0	0	1	1	0	1	0
1	0	0	1	1	0	1	1
1	0	0	1	1	0	1	0
1	0	0	1	0	0	0	0
0	0	0	1	1	0	1	0
1	0	1	1	0	0	0	1
0	1	0	1	1	0	1	0
1	0	0	1	1	0	0	0
0	0	0	1	1	0	1	0
0	1	0	1	0	0	1	0
1	0	0	1	1	0	0	0
0	0	0	1	0	0	1	1
0	1	0	1	1	0	0	0
0	1	0	1	0	0	0	0
1	1	0	1	1	0	1	0
0	0	0	1	1	0	1	1

0	1	0	1	1	0	0	1
0	1	0	1	1	0	0	0
1	0	0	1	1	0	0	0
0	1	0	1	0	0	1	0
0	0	0	1	1	0	0	1
0	1	0	1	1	0	1	0
0	0	0	1	1	0	1	0
0	1	0	1	0	0	0	1
0	1	0	0	1	0	1	0
0	0	0	1	1	0	1	0
0	0	0	1	1	0	1	0
0	1	1	0	1	0	1	0
0	0	0	1	1	0	0	0
0	0	0	1	1	0	1	0
1	0	0	1	0	0	0	0
0	1	0	1	0	0	0	1
0	0	0	1	1	0	0	0
0	0	0	0	1	0	1	0
1	0	0	1	1	0	0	1
0	0	0	1	0	0	0	0
0	0	0	1	0	0	1	0
1	1	0	1	1	0	1	0
1	0	1	1	1	0	1	1
0	0	1	1	1	0	0	0
1	0	0	1	1	0	1	1
0	0	0	1	1	0	1	1
0	1	0	1	0	0	0	0
1	0	1	1	1	0	0	0
0	0	0	1	1	0	1	0
1	1	0	1	1	0	0	0
0	1	0	1	1	0	1	0
0	1	0	1	1	0	1	1
0	0	0	1	0	0	1	0
0	1	1	1	1	0	1	1
1	1	0	1	0	0	1	1
0	1	0	1	1	0	1	1
0	1	0	1	1	0	1	1
0	1	0	1	0	0	1	1
1	0	0	1	1	0	1	1
0	1	0	1	1	0	1	0
1	1	1	1	0	0	1	0
0	1	0	1	1	0	1	0
0	1	0	1	1	0	1	0
0	1	0	1	0	0	1	0
0	1	0	1	1	1	1	0
0	0	0	1	1	0	1	1
0	1	0	1	1	0	1	0
0	0	0	1	1	0	1	1

1	1	0	1	1	0	1	0
0	0	1	1	1	0	1	0
0	1	0	1	1	0	1	0
0	1	0	1	1	0	1	0
0	0	1	1	1	0	0	0
0	1	0	1	0	0	1	1
0	1	0	0	0	0	1	0
0	0	0	1	1	0	1	0
0	1	0	1	1	0	0	0
0	1	0	0	1	0	1	0
1	0	0	1	0	0	0	0
0	1	0	1	0	0	1	0
0	1	0	0	1	0	1	0
0	0	1	0	1	0	0	0
0	0	0	1	1	0	1	1
0	1	0	1	1	0	0	0
0	1	0	1	1	0	1	0
1	1	0	1	0	0	0	0
0	1	0	1	0	0	0	0
0	1	0	1	0	0	0	0
1	1	0	1	1	0	0	1
1	1	0	1	0	0	0	0
1	0	0	1	1	0	1	0
0	1	0	1	0	0	1	0
0	1	0	1	0	0	1	0
0	1	0	1	1	0	0	1
1	0	0	1	1	0	0	0
0	0	1	1	0	0	0	0
1	0	0	0	1	0	0	0
0	0	0	1	1	0	1	1
0	0	1	1	1	0	1	1
0	0	0	1	1	0	1	1
0	0	0	1	1	0	1	0
1	0	0	1	1	0	1	0
0	0	0	1	1	0	0	1
1	0	0	1	1	0	1	0
1	0	0	1	1	0	1	0
1	1	1	1	1	0	1	0
1	0	0	1	1	0	0	0
0	1	1	1	1	0	0	1
0	0	0	1	1	0	1	1
0	0	1	1	1	0	1	1
0	0	1	1	0	0	0	1
0	0	0	1	0	0	0	0
0	0	0	1	0	0	1	0
0	0	0	1	1	0	1	0
1	0	0	1	1	0	1	0
0	0	0	1	1	0	0	1

0	1	0	1	1	0	1	0
0	0	1	1	1	0	1	0
1	0	0	1	1	0	1	0
0	0	0	1	1	0	1	0
0	1	0	1	1	0	0	0
0	0	0	0	1	0	1	0
0	1	0	1	0	0	0	0
0	1	0	1	0	0	1	0
0	0	0	1	1	0	0	0
1	0	0	1	1	0	0	0
1	0	0	1	1	0	1	0
1	0	1	1	1	0	1	0
0	1	0	1	1	0	0	0
1	0	0	1	1	0	0	0
0	1	0	1	1	0	0	0
1	0	0	0	1	0	1	0
1	0	0	0	1	0	0	0
0	0	0	1	0	0	1	0
1	0	0	1	1	0	0	0
0	0	0	1	1	0	1	0
0	0	0	1	1	0	1	1
1	0	0	1	0	0	1	0
0	1	1	1	1	0	1	1
1	0	0	1	1	0	1	1
1	0	0	1	1	0	0	1
1	0	0	1	1	0	0	1
0	0	0	1	1	0	1	0
1	0	0	1	1	0	0	1
0	1	0	1	0	0	0	1
0	1	0	1	1	0	0	0
0	1	1	1	1	0	1	1
1	1	0	1	1	0	1	0
0	0	0	1	1	0	1	1
0	0	1	1	1	0	1	1
0	0	0	1	1	0	0	0
1	0	1	1	0	0	0	0
1	1	0	1	0	0	0	0
0	0	0	1	1	0	1	0
1	0	0	1	1	0	0	0
1	0	1	1	1	0	0	0
0	0	0	1	0	0	1	0
1	0	0	1	1	0	0	0
0	1	0	1	1	0	1	0
0	1	0	1	0	0	1	0
1	1	0	1	0	0	1	0
0	0	0	1	1	0	0	1
0	0	1	0	1	0	0	0
1	1	0	1	1	0	1	0

0	1	0	1	1	0	0	0
1	0	1	1	1	0	0	0
0	1	0	1	1	0	1	0
0	1	0	1	0	0	0	0
0	1	0	1	0	0	0	1
0	0	0	1	1	0	1	0
0	1	0	1	1	0	0	0
1	0	0	1	0	0	1	0
0	0	0	1	1	0	0	0
1	0	0	1	0	0	1	0
0	0	0	1	0	0	0	0
0	0	0	1	1	0	0	0
1	1	0	1	1	0	0	0
1	1	1	1	1	0	0	0

TH	TOI	HS	DC	Apnea	CA	CH	Paraplegia	AF
0	0	0	1	0	0	0	0	0
0	0	0	0	0	0	0	0	0
1	0	0	0	0	0	0	0	0
0	0	0	0	0	0	0	0	0
0	0	0	1	0	0	0	0	0
0	0	0	1	0	0	0	0	0
0	0	0	0	0	0	0	0	0
0	0	0	1	0	0	0	0	0
0	1	0	0	0	0	0	0	0
0	0	0	0	0	0	0	0	0
0	0	0	0	0	0	0	0	0
0	0	0	1	0	0	0	0	0
0	1	0	0	0	0	0	0	0
0	0	0	0	0	0	0	0	0
0	0	0	1	0	0	0	0	0
0	0	0	0	0	0	0	0	0
0	0	0	0	0	0	0	0	0
0	0	0	0	0	0	0	0	0
0	0	0	1	0	0	0	0	0
0	0	0	1	0	0	0	0	0
0	0	0	1	0	0	0	0	0
0	0	0	1	0	0	0	0	0
0	0	0	0	0	0	0	0	0
0	0	0	1	0	0	0	0	0
0	0	0	1	0	0	0	0	0
1	1	0	0	0	0	0	0	0
0	0	0	0	0	0	0	0	0
0	0	0	1	0	0	0	0	0
0	0	0	1	0	0	0	0	0
0	0	0	1	0	0	0	0	0
0	1	0	0	0	0	0	0	0
0	1	0	0	0	0	0	0	0
0	0	0	1	0	0	0	0	0
0	0	0	1	0	0	0	0	0
0	0	0	1	0	0	0	0	0
0	0	1	1	0	0	0	0	0
0	0	0	0	0	0	0	0	0
0	1	1	0	0	0	0	0	0
0	0	0	0	0	0	0	0	0
0	0	1	0	0	0	0	0	0
0	0	0	0	0	0	0	1	0
0	0	1	0	0	0	0	0	0
1	0	0	0	0	0	0	0	0
1	0	0	1	0	0	0	0	0
0	1	1	1	0	0	0	0	0
0	0	0	0	0	0	0	0	0
0	0	0	0	0	0	0	0	0

0	0	0	0	0	0	0	0	0
1	0	0	1	0	0	0	0	0
0	0	0	0	0	0	0	0	0
0	0	0	1	0	0	0	0	0
1	1	0	0	0	0	0	0	0
0	0	1	0	0	0	0	0	0
0	0	0	1	0	0	0	0	0
0	0	0	1	0	0	0	0	0
0	0	0	0	0	0	0	0	0
0	0	0	1	0	0	0	0	0
1	0	0	0	0	0	0	0	0
0	0	0	1	0	0	0	0	0
0	0	0	0	0	0	0	0	0
0	1	1	0	0	0	0	0	0
0	0	0	0	0	0	0	0	0
1	0	0	0	0	0	0	0	0
1	0	0	1	0	0	0	0	0
0	0	0	0	0	0	0	0	0
0	0	0	0	0	0	0	0	0
0	1	0	0	0	0	0	0	0
0	1	1	0	0	0	0	0	0
0	0	0	0	0	0	0	0	0
0	0	0	1	0	0	0	0	0
0	0	0	1	0	0	0	0	0
0	0	0	0	0	0	0	0	0
0	0	0	1	0	0	0	0	0
0	0	0	1	0	0	0	0	0
0	0	0	1	0	0	0	0	0
0	0	0	1	0	0	0	0	0
0	1	0	1	0	0	0	0	0
0	0	0	1	0	0	0	0	0
0	1	0	1	0	0	0	0	0
0	1	1	0	0	0	0	0	0
0	0	1	1	0	0	0	0	0
0	0	0	1	0	0	0	0	0
0	0	0	1	0	0	0	0	0
0	0	0	1	0	0	0	0	0
0	0	0	1	0	0	0	0	0
0	0	0	1	0	0	0	0	0
1	0	0	0	0	0	0	0	0
0	0	0	0	0	0	0	0	0
0	0	0	0	0	0	0	0	0
0	0	0	0	0	0	0	0	0
0	1	1	1	0	0	0	0	0
0	0	0	0	0	0	0	0	0
1	0	0	0	0	0	0	0	0
0	0	0	1	0	0	0	0	0
0	0	0	1	0	0	0	0	0

0	0	0	0	0	0	0	0	0
0	1	0	1	0	0	0	0	0
0	0	0	0	0	0	0	0	0
1	0	0	1	0	0	0	0	0
1	0	0	0	0	0	0	0	0
0	1	1	0	0	0	0	0	0
1	0	0	1	0	0	0	0	0
1	0	0	0	0	0	0	0	0
1	0	0	1	0	0	0	0	0
0	0	0	0	0	0	0	0	0
0	0	0	0	0	0	0	0	0
0	0	0	0	0	0	0	0	0
0	0	0	1	0	0	0	0	0
0	0	0	1	0	0	0	0	0
0	0	0	0	0	0	0	0	0
0	1	0	1	0	0	0	0	0
0	0	0	0	0	0	0	0	0
0	0	0	1	0	0	0	0	0
0	0	0	1	0	0	0	0	0
1	0	0	0	0	0	0	0	0
0	0	0	0	0	0	0	0	0
0	0	0	0	0	0	0	0	0
1	0	0	0	0	0	0	0	0
0	0	0	0	0	0	0	0	0
0	0	0	1	0	0	0	0	0
0	0	0	0	0	0	0	0	0
0	0	0	0	0	0	0	0	0
0	0	0	0	0	0	0	0	0
0	0	0	0	0	0	0	0	0
0	0	0	1	0	0	0	0	0
0	0	0	0	0	0	0	0	0
0	0	0	1	0	0	0	0	0
0	0	0	0	0	0	0	0	0
0	0	0	0	0	0	0	0	0
0	0	0	1	0	0	0	0	0
0	0	0	0	0	0	0	0	0
0	0	0	0	0	0	0	0	0
0	0	0	0	0	0	0	0	0
0	0	0	0	0	0	0	0	0
0	0	0	0	0	0	0	0	0
0	0	0	0	0	0	0	0	0
0	0	0	0	0	0	0	0	0
0	0	0	0	0	0	0	0	0
1	0	0	0	0	0	0	0	0
0	0	0	0	0	0	0	0	0
0	0	0	0	0	0	0	0	0
0	0	0	0	0	0	0	0	0
0	0	0	0	0	0	0	0	0

0	0	0	1	0	0	0	0	0
0	0	0	0	0	0	0	0	0
1	0	0	1	0	0	0	0	0
0	0	0	0	0	0	0	0	0
0	0	0	1	0	0	0	0	0
0	0	0	1	0	0	0	0	0
0	0	0	1	0	0	0	0	0
0	0	0	1	0	0	0	0	0
0	0	0	0	0	0	0	0	0
0	0	0	1	0	0	0	0	0
0	0	0	0	0	0	0	0	0
0	0	0	1	0	0	0	0	0
0	1	0	1	0	0	0	0	0
0	0	0	1	0	0	0	0	0
0	1	0	0	0	0	0	0	0
0	0	0	0	0	0	0	0	0
0	0	0	0	0	0	0	0	0
0	0	0	0	0	0	0	0	0
0	0	0	1	0	0	0	0	0
0	0	0	1	0	0	0	0	0
0	0	0	0	0	0	0	0	0
0	0	0	0	0	0	0	0	0
0	0	0	0	0	0	0	0	0
0	0	0	1	0	0	0	0	0
0	0	0	0	0	0	0	0	0
0	0	0	1	0	0	0	0	0
0	0	0	1	0	0	0	0	0
0	0	0	0	0	0	0	0	0
0	0	0	0	0	0	0	0	0
0	1	0	0	0	0	0	0	0
0	0	0	1	0	0	0	0	0
0	0	0	0	0	0	0	0	0
1	0	0	1	0	0	0	0	0
0	0	0	0	0	0	0	0	0
0	0	0	0	0	0	0	0	0
1	0	0	0	0	0	0	0	0
0	0	0	1	0	0	0	0	0
0	0	0	1	0	0	0	0	0
0	0	0	1	0	0	0	0	0
0	0	0	0	0	0	0	0	0
0	0	0	0	0	0	0	0	0
0	0	0	0	0	0	0	0	0
0	0	0	0	0	0	0	0	0
1	0	0	0	0	0	0	0	0
0	0	0	1	0	0	0	0	0
0	0	0	0	0	0	0	0	0
0	0	0	0	0	0	0	0	0
0	0	0	0	0	0	0	0	0

0	0	0	0	0	0	0	0	0
0	0	0	0	0	0	0	0	0
1	0	0	0	0	0	0	0	0
0	0	0	0	0	0	0	0	0
0	0	0	0	0	0	0	0	0
1	0	0	0	0	0	0	0	0
0	0	0	0	0	0	0	0	0
0	0	0	0	0	0	0	0	0
0	0	0	0	0	0	0	0	0
0	0	0	0	0	0	0	0	0
0	0	0	0	0	0	0	0	0
0	0	0	0	0	0	0	0	0
0	0	0	1	0	0	0	0	0
1	0	0	1	0	0	0	0	0
0	0	0	1	0	0	0	0	0
0	0	0	1	0	0	0	0	0
0	1	0	0	0	0	0	0	0
0	0	0	0	0	0	0	0	0
1	0	0	0	0	0	0	0	0
0	0	0	1	0	0	0	0	0
0	0	0	1	0	0	0	0	0
0	0	0	1	0	0	0	0	0
0	0	0	1	0	0	0	0	0
0	0	0	0	0	0	0	0	0
0	0	0	1	0	0	0	0	0
0	0	0	1	0	0	0	0	0
0	0	0	1	0	0	0	0	0
0	0	0	0	0	0	0	0	0
0	0	0	1	0	0	0	0	0
0	0	0	0	0	0	0	0	0
0	0	0	0	0	0	0	0	0
0	0	0	0	0	0	0	0	0
0	0	0	0	0	0	0	0	0
0	0	0	0	0	0	0	0	0
0	0	0	0	0	0	0	0	0
0	0	0	1	0	0	0	0	0
0	0	0	1	0	0	0	0	0
0	1	0	0	0	0	0	0	0
0	0	0	0	0	0	0	0	0
0	1	1	0	0	0	0	0	0
0	0	0	1	0	0	0	0	0
0	1	0	0	0	0	0	0	0
0	0	0	1	0	0	0	0	0
0	0	0	0	0	0	0	0	0
1	0	0	0	0	0	0	0	0
0	0	0	0	0	0	0	0	0
0	0	0	1	0	0	0	0	0
0	0	0	0	0	0	0	0	0

0	0	0	0	0	0	0	0	0
0	0	0	1	0	0	0	0	0
0	0	0	1	0	0	0	0	0
0	0	0	0	0	0	0	0	0
0	0	0	0	0	0	0	0	0
0	0	0	1	0	0	0	0	0
1	0	0	0	0	0	0	0	0
1	0	0	0	0	0	0	0	0
0	0	0	1	0	0	0	0	0
0	0	0	0	0	0	0	0	0
0	0	0	0	0	0	0	0	0
1	0	0	0	0	0	0	0	0
0	0	0	1	0	0	0	0	0
0	0	0	0	0	0	0	1	0
0	0	0	0	0	0	0	0	0
1	0	0	0	0	0	0	0	0
1	0	0	0	0	0	0	0	0
0	0	0	0	0	0	0	0	0
0	0	0	0	0	0	0	0	0
0	0	0	0	0	0	0	0	0
0	0	0	1	0	0	0	0	0
0	0	0	1	0	0	0	0	0
0	0	0	0	0	0	0	0	0
0	0	0	0	0	0	0	0	0
0	0	0	0	0	0	0	0	0
0	0	0	0	0	0	0	0	0
0	0	0	0	0	0	0	0	0
0	1	1	1	1	0	0	0	0
0	1	0	0	0	0	0	0	0
0	0	0	1	0	0	0	0	0
0	1	0	0	0	0	0	0	0
0	0	0	0	0	0	0	0	0
0	1	0	0	0	0	0	0	0
0	0	0	0	0	0	0	0	0
0	0	0	1	0	0	0	0	0
0	0	0	0	0	0	0	0	0
0	0	0	0	0	0	0	0	0
0	0	0	0	0	0	0	0	0
0	0	0	0	0	0	0	0	0
0	0	0	0	0	0	0	0	0
0	0	0	0	0	0	0	0	0
0	1	0	0	0	0	0	0	0
0	0	0	0	0	0	0	0	0
1	0	0	0	0	0	0	0	0
1	0	0	0	0	0	0	0	0
1	0	0	0	0	0	0	0	0
0	0	0	1	0	0	0	0	0
0	0	0	1	0	0	0	0	0

1	0	0	0	0	0	0	0	0
1	0	0	0	0	0	0	0	0
0	0	0	1	0	0	0	0	0
0	0	0	1	0	0	0	0	0
0	0	0	1	0	0	0	0	0
0	0	0	1	0	0	0	0	0
0	0	0	1	0	0	0	0	0
0	0	0	0	0	0	0	0	0
0	0	0	0	0	0	0	0	0
0	1	1	0	0	0	0	0	0
0	0	0	1	0	0	0	0	0
0	0	0	0	0	0	0	0	0
0	0	0	0	0	0	0	0	0
0	0	0	1	0	0	0	0	0
0	0	0	0	0	0	0	0	0
0	0	0	1	0	0	0	0	0
1	0	0	0	0	0	0	0	0
0	0	0	0	0	0	0	0	0
0	0	0	0	0	0	0	0	0
0	0	0	1	0	0	0	0	0
0	0	0	1	0	0	0	0	0
0	0	0	1	0	0	0	0	0
0	0	0	1	0	0	0	0	0
0	0	0	1	0	0	0	0	0
0	0	0	1	0	0	0	0	0
0	0	0	0	0	0	0	0	0
0	0	0	0	0	0	0	0	0
0	1	0	0	0	0	0	0	0
0	0	0	1	0	0	0	0	0
0	0	0	0	0	0	0	0	0
0	0	0	1	0	0	0	0	0
0	0	0	1	0	0	0	0	0
0	0	0	0	0	0	0	0	0
0	0	0	0	0	0	0	0	0
0	0	0	1	0	0	0	0	0
0	0	0	1	0	0	0	0	0
1	0	0	1	0	0	0	0	0
0	0	0	0	0	0	0	0	0
0	0	0	1	0	0	0	0	0
0	0	0	0	0	0	0	0	0
1	0	0	1	0	0	0	0	0
0	0	0	0	0	0	0	0	0
0	0	0	0	0	0	0	0	0
0	0	0	1	0	0	0	0	0
0	0	0	0	0	0	0	0	0
0	0	0	0	0	0	0	0	0
0	0	0	1	0	0	0	0	0
0	0	0	0	0	0	0	0	0

1	0	1	0	0	0	0	0	0
0	0	0	1	1	1	0	0	0
0	0	0	1	0	0	0	0	0
1	1	1	1	0	0	0	0	0
0	0	0	1	0	0	0	1	0
0	1	1	0	0	0	0	0	0
0	0	1	1	0	0	0	0	0
1	0	0	1	0	1	0	0	0
0	0	0	1	0	0	0	0	0
0	0	0	1	0	0	0	1	0
1	0	0	1	0	0	0	0	0
0	0	0	0	0	0	0	0	0
0	0	0	1	0	0	0	0	0
0	0	0	1	0	0	0	0	0
0	0	0	0	0	0	0	0	0
0	0	0	1	0	0	0	0	0
0	0	0	1	0	0	0	0	0
0	0	0	0	1	1	0	0	0
0	0	0	0	1	1	0	1	0
0	0	0	0	0	0	0	0	0
0	1	0	0	0	0	0	0	0
0	0	0	1	0	0	0	0	0
0	0	0	0	0	0	0	0	0
0	0	0	1	0	0	1	0	0
0	0	0	1	0	0	0	0	0
0	0	0	0	0	0	0	0	0
0	0	0	0	0	0	0	0	0
0	1	0	1	0	0	0	0	0
0	0	0	0	0	0	0	0	0
0	0	0	0	0	0	0	1	0
0	0	0	0	0	0	0	0	0
0	0	0	0	0	0	0	0	0
0	0	0	0	0	0	0	0	0
0	0	0	0	0	0	0	0	0
0	0	0	0	0	0	0	0	0
0	0	0	0	0	0	0	0	0
0	0	0	1	0	0	0	0	0
0	0	0	0	0	0	0	0	0
0	0	0	1	0	0	0	0	0
0	0	0	0	0	0	0	0	0
0	0	0	0	0	0	0	1	0
0	0	0	0	0	0	0	0	0
0	0	0	0	0	0	0	0	0
0	0	0	1	0	0	0	0	0
0	0	0	0	0	0	0	0	0
0	0	0	1	0	0	0	0	0
0	0	0	0	0	0	0	1	0
0	0	0	0	0	0	0	0	0

0	0	0	1	0	0	0	0	0
0	1	1	0	1	0	0	0	0
0	0	0	1	0	0	0	0	0
1	0	0	1	0	0	0	0	0
0	0	0	1	0	0	0	0	0
1	1	1	1	0	0	0	0	0
0	0	0	1	0	0	0	0	0
1	1	0	0	0	0	0	0	0
0	0	0	1	0	0	0	0	0
0	0	1	1	0	0	0	1	1
1	1	1	0	0	0	0	0	1
1	0	0	1	0	0	0	0	0
1	1	0	0	0	0	0	1	0
0	0	0	1	0	0	0	0	0
1	1	1	1	0	0	0	0	0
0	0	0	1	0	0	0	0	0
0	0	0	1	0	0	0	0	0
1	1	0	0	0	0	0	0	0
0	0	0	0	0	0	0	0	0
0	0	0	1	0	0	0	0	0
0	1	0	1	0	0	0	0	0
1	1	0	1	0	0	0	0	0
1	0	0	1	0	0	0	0	1
0	0	0	0	0	0	0	0	0
0	1	0	0	0	0	0	0	0
0	0	0	0	0	0	0	0	0
0	0	0	0	0	0	0	0	0
0	0	0	0	0	0	0	0	0
0	0	0	0	0	0	0	1	0
0	0	0	1	0	0	0	0	0
0	0	0	0	0	0	0	0	0
1	0	0	0	0	0	0	0	0
0	0	0	0	0	0	0	0	0
0	0	0	0	0	0	0	0	0
1	0	0	0	0	0	0	0	0
1	0	0	1	0	0	0	0	1
0	0	0	1	0	0	0	0	0
1	0	0	1	0	1	0	0	0
0	0	0	0	0	0	0	0	0
1	0	0	0	0	0	0	0	0
0	0	0	1	0	0	0	0	0
0	0	0	1	0	0	0	0	0
0	0	0	0	0	0	0	0	0
0	0	0	0	0	0	0	0	0
0	0	0	1	0	0	0	0	0
0	0	0	0	0	0	0	0	0
0	0	0	0	0	0	0	0	0
0	0	0	1	0	0	0	0	0

0	0	0	0	0	0	0	0	0
0	0	0	0	0	0	0	0	0
0	0	0	0	0	0	0	0	0
0	0	0	0	0	0	0	0	0
0	0	0	0	0	0	0	0	0
0	0	0	0	0	0	0	1	0
0	1	1	0	0	0	0	0	0
0	0	0	1	0	0	0	0	0
0	0	0	0	0	0	0	0	0
0	1	0	1	0	0	0	0	0
0	0	0	1	0	0	0	0	0
0	0	0	0	0	0	0	0	0
0	0	0	1	0	0	0	0	0
0	0	0	1	0	0	0	0	0
0	1	0	0	0	0	0	0	0
0	0	0	1	0	0	0	0	0
0	0	0	0	0	0	0	0	0
1	1	1	0	0	0	0	0	0
1	0	0	1	0	0	0	0	1
1	0	0	1	0	0	0	0	0
0	0	0	1	0	0	1	0	0
0	0	0	1	0	0	0	0	0
0	0	0	1	0	0	0	0	0
0	0	0	0	0	0	0	0	0
0	0	0	1	0	0	0	0	0
0	0	0	1	0	0	0	0	0
0	0	0	0	0	0	0	1	0
0	1	1	0	0	0	0	0	0
1	0	0	1	0	0	0	0	0
0	0	0	1	0	0	0	0	0
1	1	0	0	0	0	0	0	0
0	0	0	1	0	0	0	0	0
0	0	0	0	0	0	0	0	0
0	0	0	0	0	0	0	0	0
0	0	0	0	0	0	0	0	0
0	0	0	0	0	0	0	0	0
0	0	0	1	0	0	0	0	0
0	0	0	1	0	0	0	0	0
0	0	0	1	0	0	0	0	0
0	0	0	1	0	0	0	0	0
0	0	0	1	0	0	0	0	0
0	0	0	1	0	0	0	0	0
0	0	0	1	1	1	0	0	0
0	0	0	0	0	0	0	0	0
0	0	0	0	0	0	0	0	0
0	0	0	1	0	0	0	0	0
0	0	0	1	0	0	0	0	0
0	0	0	1	0	0	0	0	0

0	0	0	0	0	0	0	0	0
0	0	0	0	0	0	0	0	0
0	0	0	0	0	0	0	0	0
0	0	0	0	0	0	0	0	0
0	0	0	0	0	0	0	0	0
0	0	0	0	0	0	0	0	0
0	0	0	0	0	0	0	0	0
0	0	0	1	0	0	0	1	0
0	0	0	0	0	0	0	0	0
0	0	0	0	0	0	0	0	0
0	0	0	0	0	0	0	0	0
0	0	0	0	0	0	0	0	0
1	0	0	0	0	0	0	0	0
0	0	0	0	0	0	0	0	0
0	0	0	0	0	0	0	0	0
0	0	0	0	0	0	0	0	0
0	0	0	0	0	0	0	0	0
0	0	0	0	0	0	0	0	0
0	0	0	1	0	0	0	0	0
0	0	0	0	0	0	0	0	0
0	0	0	0	0	0	0	0	0
0	0	0	0	0	0	0	0	0
0	0	0	0	0	0	0	0	0
0	0	0	0	0	0	0	1	0
1	0	1	1	0	0	0	0	0
0	1	1	0	0	0	0	0	0
0	1	0	0	0	0	0	0	0
0	1	0	1	0	0	0	0	0
1	1	1	0	0	0	0	0	0
0	0	0	1	0	0	0	1	0
0	1	1	0	0	0	0	0	0
0	1	1	0	0	0	0	1	0
0	1	0	0	0	0	0	0	0
0	0	0	1	0	0	0	0	0
1	1	0	0	0	0	0	0	0
0	1	0	0	0	0	0	0	0
1	1	1	0	0	0	0	0	1
1	1	1	1	0	0	0	0	0
0	0	0	0	0	0	0	0	0
0	0	1	0	0	0	0	0	0
0	1	0	0	0	0	0	0	0
0	1	0	1	0	0	0	1	0
0	0	0	0	0	0	0	0	0
0	0	0	0	0	0	0	1	0
1	0	0	1	0	0	0	0	0
1	0	0	1	0	0	0	0	0
0	0	0	1	0	0	0	0	0
1	1	1	1	0	0	0	0	0

1	1	0	0	0	0	0	0	0
1	1	0	1	0	0	0	0	0
0	1	0	0	0	0	0	0	1
0	0	0	1	0	0	0	0	0
0	0	0	1	0	0	0	0	0
0	0	0	0	0	0	0	1	0
0	0	0	1	0	0	0	1	0
0	0	0	0	0	0	0	0	0
1	0	0	1	0	0	0	0	0
0	0	0	0	0	0	0	0	0
0	0	0	1	0	0	0	0	0
0	1	1	0	0	0	0	0	0
0	0	0	1	0	0	0	1	0
0	0	0	1	0	0	0	0	0
0	0	1	0	0	0	0	0	0
0	0	0	1	0	0	0	0	0
0	0	0	0	0	0	0	1	0
0	0	0	0	0	0	0	1	0
0	0	0	1	0	0	0	0	0
1	0	1	1	1	1	0	0	0
0	0	0	0	0	0	0	1	0
0	0	0	0	0	0	0	1	0
0	1	0	0	0	0	0	0	0
0	1	0	0	0	0	0	0	0
0	0	0	1	0	0	0	0	0
0	0	0	0	0	0	0	1	0
0	0	0	1	0	0	0	0	0
0	0	0	0	0	0	0	0	0
0	0	0	1	0	0	0	0	0
0	0	0	0	0	0	0	0	0
0	0	0	0	0	0	0	0	0
0	0	0	1	0	0	0	0	0
0	0	0	0	0	0	0	0	0
0	1	1	0	1	1	0	0	0
1	0	0	1	0	0	0	0	0
1	1	1	0	0	0	0	0	0
0	0	0	0	0	0	0	0	0
0	0	0	0	0	0	0	0	0
0	0	0	1	0	0	0	0	0
1	0	0	0	0	0	0	1	0
0	0	0	1	0	0	0	0	0
0	0	0	0	0	0	0	1	0
0	0	0	0	0	0	0	0	0
1	0	0	0	0	0	0	0	0
0	0	0	0	0	0	0	0	0
0	0	0	0	0	0	0	0	0
0	0	0	0	0	0	0	0	0
0	0	0	0	0	0	0	0	0

0	0	0	0	0	0	0	0	0
0	0	0	1	0	0	0	0	1
0	0	0	0	0	0	0	0	0
0	0	0	1	0	0	0	0	0
0	0	0	0	0	0	0	0	1
0	1	0	0	0	0	0	0	0
0	0	0	0	0	0	0	0	0
0	1	0	0	0	0	0	0	0
0	1	0	0	0	0	0	0	0
0	0	0	0	0	0	0	0	0
0	0	0	0	0	0	0	0	0
0	0	0	0	0	0	0	0	0
0	0	0	0	0	0	0	0	0
0	0	0	0	0	0	0	0	1
0	0	0	0	0	0	0	0	0
0	0	0	0	0	0	0	1	0
0	1	0	0	0	0	0	0	0
0	0	0	0	0	0	0	0	0
0	0	0	0	0	0	0	0	0
0	0	0	1	0	0	0	0	0
0	0	0	0	0	0	0	0	0
1	0	0	0	0	0	0	0	0
0	0	0	0	0	0	0	0	0
0	0	0	0	0	0	0	0	0
0	0	1	0	0	0	0	0	0
1	0	0	0	0	0	0	0	0
0	0	0	0	0	0	0	0	0
0	0	0	0	0	0	0	0	0
0	0	0	0	0	0	0	0	0
0	0	0	1	0	0	0	0	0
0	0	0	0	0	0	0	0	0
0	0	0	0	0	0	0	0	0
0	0	0	0	0	0	0	0	0
0	0	0	1	0	0	0	0	0
0	0	0	0	0	0	0	0	0
0	0	0	0	0	0	0	0	0
0	0	0	0	0	0	0	0	0
0	0	0	0	0	0	0	0	0
0	0	0	0	0	0	0	0	0
0	0	0	0	0	0	0	0	0
0	0	0	0	0	0	0	0	0
0	0	0	0	0	0	0	0	0
0	0	0	0	0	0	0	0	0
0	0	0	0	0	0	0	0	0
0	0	0	0	0	0	0	0	0
0	0	0	0	0	0	0	0	0
0	0	0	0	0	0	0	0	0
0	0	0	0	0	0	0	0	0

0	0	0	0	0	0	0	0	0
0	0	1	1	0	0	0	0	0
0	0	0	0	0	0	0	0	0
0	0	0	1	0	0	0	1	0
0	0	0	1	0	0	0	0	0
0	1	0	1	0	0	0	0	0
0	0	0	0	0	0	0	0	0
1	0	0	0	0	0	0	0	0
0	0	0	0	0	0	0	0	0
0	0	0	0	0	0	0	0	0
0	0	0	0	0	0	0	0	0
0	0	0	0	0	0	0	0	0
1	0	0	1	0	0	0	0	0
0	0	0	0	0	0	0	0	0
0	1	0	0	0	0	0	0	0
0	0	0	0	0	0	0	0	0
0	0	0	0	0	0	0	0	0
0	0	0	0	0	0	0	0	0
0	0	1	0	0	0	0	0	0
0	0	0	0	0	0	0	0	0
1	0	0	0	0	0	0	0	0
0	0	1	0	0	0	0	0	0
0	0	0	0	0	0	0	0	0
0	0	0	0	0	0	0	0	0
0	0	1	0	0	0	0	0	0
0	0	0	0	0	0	0	0	0
0	0	0	0	0	0	0	0	0
0	0	0	0	0	0	0	0	0
0	0	0	0	0	0	0	0	0
0	0	0	0	0	0	0	0	0
0	0	0	0	0	0	0	0	0
0	0	0	1	0	0	0	0	0
0	0	0	0	0	0	0	0	0
0	1	0	0	0	0	0	0	0
0	0	0	0	0	0	0	0	0
0	0	0	1	0	0	0	0	0
0	0	0	0	0	0	0	0	0
1	0	0	1	0	0	0	0	0
1	0	0	1	0	0	0	0	0
0	0	0	0	0	0	0	0	0
0	0	0	0	0	0	0	0	0
0	0	1	0	0	0	0	0	0
0	0	1	0	0	0	0	0	0
0	0	0	0	0	0	0	0	0
1	0	0	1	0	0	0	0	0
0	0	1	0	0	0	0	0	0
0	0	0	0	0	0	0	0	0
1	0	1	1	0	0	0	0	0

1	0	0	0	0	0	0	0	0
0	0	0	0	0	0	0	0	0
0	0	0	1	0	0	0	0	0
0	0	0	0	0	0	0	0	0
0	0	0	0	0	0	0	0	0
0	0	0	0	0	0	0	0	0
1	0	1	1	0	0	0	0	0
0	0	0	0	0	0	0	0	0
0	0	0	0	0	0	0	0	0
0	0	0	1	0	0	0	0	0
0	0	0	1	0	0	0	0	0
0	0	0	0	0	0	0	0	0
1	0	0	0	0	0	0	0	0
0	0	0	0	0	0	0	0	0
0	0	0	1	0	0	0	0	0
0	0	0	0	0	0	0	0	0
0	0	0	0	0	0	0	0	0
1	0	0	0	0	0	0	0	0
0	0	0	0	0	0	0	0	0
1	0	0	1	0	0	0	0	0
1	0	0	1	0	0	0	0	0
1	0	0	0	0	0	0	0	0
1	0	0	0	0	0	0	0	0
1	0	0	0	0	0	0	0	0
0	0	0	0	0	0	0	0	0
0	0	1	0	0	0	0	0	0
0	0	0	1	0	0	0	0	0
0	0	0	0	0	0	0	0	0
1	0	0	0	0	0	0	0	0
1	0	0	0	0	0	0	0	0
0	0	1	0	0	0	0	0	0
0	0	0	0	0	0	0	0	0
0	0	0	0	0	0	0	0	0
0	0	0	0	0	0	0	0	0
0	0	0	0	0	0	0	0	0
1	0	0	1	0	0	0	0	0
1	0	0	0	0	0	0	0	0
0	0	0	0	0	0	0	0	0
0	0	0	0	0	0	0	0	0
1	1	0	0	0	0	0	0	0
0	0	0	0	0	0	0	0	0
0	0	1	0	0	0	0	0	0
0	0	0	0	0	0	0	0	0
0	0	0	0	0	0	0	0	0
0	0	1	0	0	0	0	0	0
0	0	0	0	0	0	0	0	0
0	0	0	0	0	0	0	0	0
0	0	0	0	0	0	0	0	0

0	0	1	0	0	0	0	0	0
0	0	0	1	0	0	0	0	0
0	0	0	0	0	0	0	0	0
0	0	0	0	0	0	0	0	0
0	0	0	0	0	0	0	0	0
0	0	0	1	0	0	0	0	0
0	0	1	0	0	0	0	0	0
0	0	0	0	0	0	0	0	0
0	0	0	0	0	0	0	0	0
0	0	0	1	0	0	0	0	0
0	0	0	0	0	0	0	0	0
0	0	0	0	0	0	0	0	0
0	0	0	0	0	0	0	0	0
0	0	1	0	0	0	0	0	0
0	0	0	0	0	0	0	0	0
0	0	0	0	0	0	0	0	0
0	0	0	0	0	0	0	0	0
0	0	1	1	0	0	0	0	0
0	0	0	0	0	0	0	0	0
0	0	1	0	0	0	0	0	0
0	0	0	0	0	0	0	0	0
0	0	0	0	0	0	0	0	0
0	0	1	1	0	0	0	0	0
0	0	0	0	0	0	0	0	0
0	0	0	1	0	0	0	0	0
0	0	0	0	0	0	0	0	0
0	0	0	0	0	0	0	0	0
1	1	0	0	0	0	0	0	0
0	0	1	0	0	0	0	0	0
0	0	1	0	0	0	0	0	0
0	0	1	0	0	0	0	0	0
0	0	1	0	0	0	0	0	0
0	0	0	0	0	0	0	0	0
0	0	0	0	0	0	0	0	0
0	0	0	0	0	0	0	0	0
0	0	0	0	0	0	0	0	0
0	0	1	0	0	0	0	1	0
0	0	0	0	0	0	0	0	0
0	0	0	1	0	0	0	0	0
0	0	0	0	0	0	0	0	0
0	0	0	0	0	0	0	0	0
0	1	0	0	0	0	0	0	0
0	0	0	0	0	0	0	0	0
0	0	0	0	0	0	0	0	0
1	0	0	0	0	0	0	0	0
0	0	0	0	0	0	0	0	0
0	0	0	0	0	0	0	0	0
0	0	0	0	0	0	0	0	0

0	0	1	0	0	0	0	0	0
0	0	0	0	0	0	0	1	0
1	0	0	0	0	0	0	1	0
0	0	0	1	0	0	0	0	0
0	0	0	0	0	0	0	0	0
0	0	0	0	0	0	0	0	0
0	0	0	0	0	0	0	0	1
0	0	0	0	0	0	0	0	0
0	0	1	0	0	0	0	0	0
1	1	0	0	0	0	0	0	0
0	0	0	1	0	0	0	0	0
0	0	0	0	0	0	0	0	0
0	0	0	1	0	0	0	0	0
0	0	1	0	0	0	0	0	0
0	0	0	1	0	0	0	0	0
0	0	0	0	0	0	0	0	0
0	0	0	0	0	0	0	0	0
0	0	0	0	0	0	0	0	0
0	0	0	0	0	0	0	0	0
0	0	0	0	0	0	0	0	0
0	0	0	0	0	0	0	0	0
0	0	0	0	0	0	0	0	0
0	0	0	0	0	0	0	0	0
0	0	0	0	0	0	0	0	1
0	0	0	0	0	0	0	0	0
0	0	0	0	0	0	0	0	0
0	0	0	0	0	0	0	0	0
0	0	0	1	0	0	0	0	0
0	0	0	0	0	0	0	0	0
0	0	0	1	0	0	0	0	0
0	0	0	0	0	0	0	0	0
0	0	0	1	0	0	0	0	0
0	0	0	0	0	0	0	0	0
0	0	0	1	0	0	0	0	0
0	0	0	1	0	0	0	0	0
0	0	0	0	0	0	0	0	0
0	0	0	1	0	0	0	0	0
1	0	1	0	0	0	0	0	0
1	0	0	1	0	0	0	1	0
0	0	0	0	0	0	0	0	0
0	0	0	1	0	0	0	0	0
0	0	0	1	0	0	0	0	0
0	0	0	0	0	0	0	0	0
0	0	0	1	0	0	0	0	0
0	0	0	0	0	0	0	0	0
0	0	0	0	0	0	0	0	0
0	0	1	1	0	0	0	1	0
0	0	0	1	0	0	0	0	0

0	0	0	0	0	0	0	0	0
0	0	0	1	0	0	0	0	0
0	0	0	1	0	0	0	0	0
0	1	0	1	0	0	0	0	0
0	0	0	0	0	0	0	0	0
0	0	0	1	0	0	0	1	0
0	0	0	1	0	0	0	0	0
0	0	0	1	0	0	0	0	0
0	0	0	1	0	0	0	0	0
0	0	0	1	0	0	0	0	0
0	0	0	1	0	0	0	0	0
0	0	0	1	0	0	0	0	0
0	0	0	0	0	0	0	0	0
0	0	0	1	0	0	0	0	0
0	0	1	1	0	0	0	0	0
0	0	0	0	0	0	0	1	0
0	0	0	0	0	0	0	0	0
0	0	0	1	0	0	0	0	0
0	0	0	0	0	0	0	0	0
0	1	0	0	0	0	0	0	0
0	0	0	1	0	0	0	0	0
0	0	0	0	0	0	0	0	0
1	1	0	1	0	0	0	0	0
0	1	0	0	0	0	0	0	0
1	0	0	1	0	0	0	0	0
0	0	0	0	0	0	0	0	0
0	0	0	0	0	0	0	1	0
0	1	0	0	0	0	0	0	0
0	0	0	1	0	0	0	0	0
0	1	0	0	0	0	0	0	0
0	0	0	1	0	0	0	0	0
1	0	0	1	0	0	0	0	0
0	0	0	1	0	0	0	0	0
0	1	1	1	0	0	0	0	0
0	0	0	0	0	0	0	0	0
1	1	0	0	0	0	0	0	0
1	0	0	1	0	0	0	0	0
1	0	0	1	0	0	0	0	0
0	0	0	1	0	0	0	0	0
0	0	0	0	0	0	0	0	0
0	0	0	1	0	0	0	1	0
0	0	0	0	0	0	0	1	0
0	0	0	1	0	0	0	1	0
0	0	0	0	0	0	0	0	0
0	0	0	1	0	0	0	0	0
0	0	0	1	0	0	0	0	0
0	0	0	1	0	0	0	0	0
0	0	0	1	0	0	0	0	0

0	0	0	1	0	0	0	1	0
0	0	0	1	0	0	0	0	0
0	0	0	1	0	0	0	0	0
0	0	0	1	0	0	0	1	0
1	0	0	1	0	0	0	0	0
0	0	0	1	0	0	0	1	0
0	0	0	1	0	0	0	0	0
0	1	0	1	0	0	0	0	0
0	0	0	0	0	0	0	0	0
0	0	0	0	0	0	0	0	0
0	0	0	0	0	0	0	0	0
1	0	0	0	0	0	0	0	0
0	0	0	1	0	0	0	1	0
1	0	0	0	0	0	0	0	0
0	0	0	1	0	0	0	0	0
0	0	0	0	0	0	0	0	0
0	0	0	1	0	0	0	0	0
0	0	0	0	0	0	0	0	0
1	1	0	0	0	0	0	1	0
0	0	0	0	0	0	0	0	0
0	0	0	1	0	0	0	1	0
0	0	0	1	0	0	0	0	0
0	0	0	1	0	0	0	0	0
0	0	0	1	0	0	0	1	0
0	0	0	1	0	0	0	0	0
0	0	0	0	0	0	0	0	0
0	0	0	1	0	0	0	0	0
0	1	0	0	0	0	0	0	0
0	0	0	1	0	0	0	0	0
1	0	1	1	0	0	0	0	0
1	0	1	1	0	0	0	0	0
0	0	0	1	0	0	0	0	0
0	0	0	0	0	0	0	0	0
0	0	0	1	0	0	0	0	0
0	0	1	1	0	0	0	0	0
0	0	0	0	0	0	0	0	0
0	0	0	1	0	0	0	0	0
0	1	1	1	0	0	0	1	0
0	0	0	1	0	0	0	0	0
0	1	1	0	0	0	0	0	0
0	0	0	1	0	0	0	0	0
1	0	0	1	0	0	0	0	0
0	0	0	0	0	0	0	0	0
0	1	0	1	0	0	0	0	0
0	0	0	1	0	0	0	0	0
0	0	0	1	0	0	0	0	0
0	0	0	0	0	0	0	0	0
0	0	0	1	0	0	0	0	0

0	0	0	1	0	0	0	0	0
0	0	0	1	0	0	0	0	0
0	0	0	1	0	0	0	0	0
1	0	0	1	0	0	0	0	0
0	0	0	1	0	0	0	0	0
0	0	0	1	0	0	0	0	0
0	0	0	0	0	0	0	0	0
0	0	0	1	0	0	0	1	0
1	0	0	0	0	0	0	0	0
0	0	0	0	0	0	0	0	0
0	0	0	1	0	0	0	0	0
0	0	0	1	0	0	0	0	0
0	0	0	0	0	0	0	1	0
0	0	0	0	0	0	0	0	0
0	0	0	0	0	0	0	1	0
0	0	0	1	0	0	0	0	0
0	0	0	0	0	0	0	0	0
0	0	0	0	0	0	0	0	0
0	0	0	0	0	0	0	0	0
0	0	0	0	0	0	0	0	0
0	0	0	1	0	0	0	0	0
0	0	0	1	0	0	0	0	0
0	1	0	1	0	0	0	0	0
1	0	0	1	0	0	0	0	0
1	0	0	0	0	0	0	0	0
0	0	0	1	0	0	0	0	0
0	0	0	1	0	0	0	0	0
1	0	0	0	0	0	0	0	0
1	0	1	1	0	0	0	1	0
0	1	0	0	0	0	0	0	0
0	0	1	0	0	0	0	0	0
0	0	0	1	0	0	0	0	0
0	0	0	1	0	0	0	0	0
1	0	1	1	0	0	0	0	0
1	0	0	0	0	0	0	0	0
0	1	0	0	0	0	0	0	0
0	0	0	0	0	0	0	0	0
0	0	0	1	0	0	0	0	0
0	0	0	1	0	0	0	0	0
0	1	1	0	0	0	0	0	0
0	0	0	1	0	0	0	0	0
0	0	0	1	0	0	0	0	0
0	0	0	1	0	0	0	0	1
0	0	0	1	0	0	0	0	0
0	0	0	1	0	0	0	0	0
1	0	0	0	0	0	0	0	0
0	0	0	0	0	0	0	0	0
0	0	0	1	0	0	0	0	0

0	0	0	0	0	0	0	0	0
0	0	0	1	0	0	0	0	0
0	0	0	1	0	0	0	0	0
0	0	0	1	0	0	0	1	0
0	0	0	1	0	0	0	0	0
0	0	0	1	0	0	0	0	0
0	0	0	1	0	0	0	1	0
0	0	0	1	0	0	0	0	0
1	0	0	0	0	0	0	0	0
0	0	0	0	0	0	0	0	0
0	0	0	0	0	0	0	0	0
0	0	0	1	0	0	0	0	0
0	0	0	0	0	0	0	0	0
0	1	1	0	0	0	0	0	0

WED	ABI	ARDS	ARF	MODS	BI	ISS	Diabetes	Hypertension
0	0	0	0	0	0	3	0	0
0	0	0	0	0	0	3	0	0
0	0	0	0	0	0	3	0	0
0	0	0	0	0	0	2	0	0
0	0	0	0	0	0	2	0	0
0	0	0	0	0	0	4	0	0
0	0	0	0	0	1	1	0	0
0	0	0	0	0	0	2	0	1
0	0	0	0	0	0	4	0	0
0	0	0	0	0	0	3	0	0
0	0	0	0	0	0	2	1	0
0	0	0	0	0	0	2	0	0
0	0	0	0	0	0	2	0	0
0	0	0	0	0	0	2	0	0
0	0	0	0	0	0	1	0	0
0	0	0	0	0	0	3	0	0
0	0	0	0	0	0	2	0	0
0	0	0	0	0	0	4	0	0
0	0	0	0	0	0	2	0	0
0	0	0	0	0	0	4	1	0
0	0	0	0	0	0	2	0	0
0	0	0	0	0	0	3	0	0
0	0	0	0	0	0	3	0	0
0	0	0	0	0	0	3	0	0
0	0	0	0	0	0	3	0	1
0	0	0	0	0	0	3	0	0
0	0	0	0	0	0	3	0	0
0	0	0	0	0	0	1	0	0
0	0	0	0	0	0	2	0	0
0	0	0	1	0	1	2	0	1
0	0	0	0	0	0	3	0	0
0	0	0	0	0	1	3	1	0
0	0	0	0	0	0	4	0	0
0	0	0	0	0	0	4	0	0
0	0	0	0	0	0	2	0	0
0	0	0	0	0	0	3	0	1
0	0	0	0	0	1	2	0	0
0	0	0	0	0	0	4	0	0
0	0	0	0	0	0	1	0	0
0	0	0	0	0	0	3	0	0
0	0	0	0	0	1	4	1	1
0	0	0	0	0	0	3	0	0
0	0	0	0	0	0	2	0	0
0	0	0	0	0	0	4	0	0
0	0	0	0	0	0	2	0	0
0	0	0	0	0	0	3	0	0
0	0	0	0	0	0	3	0	0

0	0	0	0	0	0	2	0	1
0	0	0	0	0	0	4	0	0
0	0	0	0	0	0	3	0	0
0	0	0	0	0	0	2	0	0
0	0	0	0	0	0	4	0	0
0	0	0	0	0	0	4	0	0
0	0	0	0	0	0	4	0	0
0	0	0	0	0	1	4	0	0
0	0	0	0	0	0	1	0	0
0	0	0	0	0	0	1	0	0
0	0	0	0	0	0	3	0	0
0	0	0	0	0	0	2	0	0
0	0	0	0	0	0	3	0	1
0	0	0	0	0	0	4	0	0
0	0	0	0	0	0	2	0	0
0	0	0	0	0	0	3	0	0
0	0	0	0	0	1	4	0	0
0	0	0	0	0	0	1	1	0
0	0	0	0	0	0	3	0	1
0	0	0	0	0	0	3	0	0
0	0	0	0	0	0	4	0	0
0	0	0	0	0	0	4	0	0
0	0	0	0	0	0	4	0	0
0	0	0	0	0	0	3	0	0
0	0	0	0	0	0	3	0	0
0	0	0	0	0	0	3	0	0
0	0	0	0	0	0	3	0	0
0	0	0	0	0	0	4	0	0
0	0	0	0	0	0	3	0	0
0	0	0	0	0	0	4	1	1
0	0	0	0	0	0	3	0	0
0	0	0	0	0	0	2	0	0
0	0	0	0	0	0	2	0	0
0	0	0	0	0	1	3	0	0
0	0	0	0	0	0	2	0	0
0	0	0	0	0	0	3	0	0
0	0	0	0	0	0	4	0	0
0	0	0	0	0	0	1	0	0
0	0	0	0	0	1	2	0	0
0	0	0	0	0	0	4	0	1
0	0	0	0	0	0	2	0	0
0	0	0	0	0	0	2	0	0
0	0	0	0	0	0	2	0	0
0	0	0	0	0	1	4	0	0
0	0	0	0	0	0	3	0	0
0	0	0	0	1	0	3	1	0
0	0	0	0	0	0	3	0	0
0	0	0	0	0	0	2	0	0

0	0	0	0	0	0	2	0	0
0	0	0	0	0	0	3	0	0
0	0	0	0	0	0	2	0	0
0	0	0	0	0	0	3	0	0
0	0	0	0	0	0	3	0	0
0	0	0	0	0	0	3	0	0
0	0	0	0	0	0	3	0	0
0	0	0	0	0	0	3	0	0
0	0	0	0	0	0	3	0	0
0	0	0	0	0	0	3	0	0
0	0	0	0	0	0	2	0	0
0	0	0	0	0	1	2	1	1
0	0	0	0	0	0	2	0	0
0	0	0	0	0	0	2	0	0
0	0	0	0	0	0	1	0	0
0	0	0	0	0	0	4	0	0
0	0	0	0	0	1	3	0	0
0	0	0	0	0	0	3	0	0
0	0	0	0	0	1	3	0	0
0	0	0	0	0	0	2	0	0
0	0	0	0	0	0	4	0	1
0	0	0	0	0	0	2	0	0
0	0	0	0	0	0	4	0	0
0	0	0	0	0	0	3	0	0
0	0	0	0	0	0	2	0	0
0	0	0	0	0	0	1	0	0
0	0	0	0	0	0	1	0	1
0	0	0	0	0	0	2	0	0
0	0	0	0	0	0	3	0	0
0	0	0	0	0	0	2	0	0
0	0	0	0	0	0	1	1	1
0	0	0	0	0	0	2	0	0
0	0	0	0	0	0	2	0	0
0	0	0	0	0	0	1	0	0
0	0	0	0	0	0	3	0	0
0	0	0	0	0	0	2	0	0
0	0	0	0	0	0	2	0	0
0	0	0	0	0	0	3	1	1
0	0	0	0	0	0	2	0	0
0	0	0	0	0	0	2	0	1
0	0	0	0	0	0	1	0	0
0	0	0	0	0	0	2	0	0
0	0	0	0	0	0	2	0	0
0	0	0	0	0	0	2	0	0
0	0	0	0	0	0	2	1	0
0	0	0	0	0	0	2	0	0
0	0	0	0	0	0	2	0	1
0	0	0	0	0	0	2	0	1

0	0	0	0	0	0	3	0	0
0	0	0	0	0	0	1	0	0
0	0	0	0	0	0	4	0	0
0	0	0	0	0	0	2	0	0
0	0	0	0	0	0	3	0	0
0	0	0	0	0	0	1	0	0
0	0	0	0	0	0	4	0	0
0	0	0	0	0	0	3	0	0
0	0	0	0	0	0	2	0	0
0	0	0	0	0	0	2	0	0
0	0	0	0	0	0	2	0	0
0	0	0	0	0	0	1	0	0
0	0	0	0	0	0	4	0	0
0	0	0	0	0	0	2	0	0
0	0	0	0	0	0	4	0	0
0	0	0	0	0	0	2	0	0
0	0	0	0	0	0	2	0	0
0	0	0	0	0	0	1	0	0
0	0	0	0	0	0	2	0	0
0	0	0	0	0	0	2	0	0
0	0	0	0	0	0	2	1	0
0	0	0	0	0	0	1	0	0
0	0	0	0	0	0	2	0	0
0	0	0	0	0	0	1	0	0
0	0	0	0	0	0	2	0	0
0	0	0	0	0	0	2	0	0
0	0	0	0	0	0	3	0	0
0	0	0	0	0	0	1	0	0
0	0	0	0	0	0	1	0	0
0	0	0	0	0	0	2	0	0
0	0	0	0	0	0	1	0	1
0	0	0	0	0	0	1	0	0
0	0	0	0	0	0	3	0	0
0	0	0	0	0	0	2	0	0
0	0	0	0	0	0	2	0	0
0	0	0	0	0	0	3	0	0
0	0	0	0	0	0	3	0	0
0	0	0	0	0	0	3	0	0
0	0	0	0	0	0	1	0	0
0	0	0	0	0	0	2	0	0
0	0	0	0	0	0	1	0	0
0	0	0	0	0	0	1	0	0
0	0	0	0	0	0	3	0	0
0	0	0	0	0	0	2	0	1
0	0	0	0	1	0	4	0	1
0	0	0	0	0	0	2	1	1
0	0	0	0	0	0	2	0	0
0	0	0	0	0	0	1	0	0

0	0	0	0	0	0	3	0	0
0	0	0	0	0	0	1	0	0
0	0	0	0	0	0	3	0	0
0	0	0	0	0	0	2	0	0
0	0	0	0	0	0	2	0	0
0	0	0	0	0	0	2	0	0
0	0	0	0	0	0	2	0	1
0	0	0	0	0	0	1	0	0
0	0	0	0	0	0	1	0	0
0	0	0	0	0	0	1	1	1
0	0	0	0	0	0	1	0	0
0	0	0	0	0	0	1	0	0
0	0	0	0	0	0	3	0	1
0	0	0	0	0	0	2	0	0
0	0	0	0	0	0	2	0	0
0	0	0	0	0	0	2	0	0
0	0	0	0	0	0	2	0	0
0	0	0	0	0	0	1	0	0
0	0	0	0	0	0	2	0	0
0	0	0	0	0	0	1	0	0
0	0	0	0	0	1	2	0	0
0	0	0	0	0	0	2	0	0
0	0	0	0	0	0	1	0	1
0	0	0	0	0	0	1	0	0
0	0	0	0	0	1	3	0	0
0	0	0	0	0	0	2	0	1
0	0	0	0	0	0	2	0	0
0	0	0	0	0	0	1	0	0
0	0	0	0	0	0	1	0	0
0	0	0	0	0	0	2	0	0
0	0	0	0	0	0	1	0	0
0	0	0	0	0	0	2	0	0
0	0	0	0	0	0	1	0	0
0	0	0	0	0	0	2	0	0
0	0	0	0	0	0	1	0	0
0	0	0	0	0	0	3	0	0
0	0	0	0	0	0	1	0	0
0	0	0	0	0	0	4	0	0
0	0	0	0	0	0	1	0	0
0	0	0	0	0	0	4	0	0
0	0	0	0	0	1	3	0	0
0	0	0	0	0	0	4	0	1
0	0	0	0	0	0	3	0	0
0	0	0	0	0	0	2	1	1
0	0	0	0	0	0	3	0	0
0	0	0	0	0	0	1	1	1
0	0	0	0	0	0	2	0	0
0	0	0	0	0	0	1	0	0

0	0	0	0	0	0	2	0	1
0	0	0	0	0	0	3	0	0
0	0	0	0	0	0	2	0	0
0	0	0	0	0	0	2	0	0
0	0	0	0	0	0	4	0	0
0	0	0	0	0	0	3	0	0
0	0	0	0	0	0	2	0	1
0	0	0	0	0	0	2	1	1
0	0	0	0	0	0	3	0	0
0	0	0	0	0	0	1	0	0
0	0	0	0	0	0	1	1	1
0	0	0	0	0	0	3	0	0
0	0	0	0	0	0	2	0	0
0	0	0	0	0	0	4	0	0
0	0	1	0	0	1	3	0	1
0	0	0	0	0	0	2	0	0
0	0	0	0	0	0	2	0	0
0	0	0	0	0	0	1	1	1
0	0	0	0	0	0	1	0	0
0	0	0	0	0	0	1	0	0
0	0	0	0	0	0	2	0	0
0	0	0	0	0	1	4	0	0
0	0	0	0	0	0	1	0	0
0	0	0	0	0	0	2	1	1
0	0	0	0	0	0	1	0	0
0	0	0	1	0	1	3	0	0
0	0	0	0	0	0	1	0	0
0	0	0	0	0	0	4	0	0
0	0	0	0	0	0	4	0	0
0	0	0	0	1	1	4	0	0
0	0	0	0	0	1	4	1	1
0	0	0	0	0	0	2	0	0
0	0	0	0	0	1	3	0	0
0	0	0	0	0	0	2	0	0
0	0	0	0	0	0	1	0	0
0	0	0	0	0	0	2	0	0
0	0	0	0	0	0	2	0	0
0	0	0	0	0	0	3	0	1
0	0	0	0	0	0	3	1	1
0	0	0	0	0	0	1	0	0
0	0	0	0	0	0	2	0	0
0	0	0	0	0	0	3	0	0
0	0	0	0	0	0	2	0	0
0	0	0	0	0	0	3	0	0
0	0	0	0	0	0	2	0	0
0	0	0	0	0	0	3	0	0
0	0	0	0	0	0	3	0	0
0	0	0	0	0	0	3	0	0

0	0	0	0	0	0	3	1	1
0	0	0	0	0	1	4	0	0
0	0	0	0	0	0	1	0	0
0	0	0	0	0	0	2	0	0
0	0	0	0	0	0	2	0	0
0	0	0	0	0	0	4	0	0
0	0	0	0	0	0	2	0	1
0	0	0	0	0	0	1	0	0
0	0	0	0	0	0	1	0	0
0	0	0	0	0	0	3	0	0
0	0	0	0	0	0	4	0	0
0	0	0	0	0	0	3	0	0
0	0	0	0	0	0	1	0	0
0	0	0	0	0	0	2	0	0
0	0	0	0	0	1	3	0	0
0	0	0	0	0	0	4	0	0
0	0	0	0	0	0	4	0	0
0	0	0	0	0	1	4	0	0
0	0	0	0	0	0	3	0	0
0	0	0	0	0	0	2	0	0
0	0	0	0	0	0	3	0	0
0	0	0	0	0	1	3	0	0
0	0	0	0	0	0	2	0	0
0	0	0	0	0	0	3	0	0
0	0	0	0	0	0	4	0	0
0	0	0	0	0	1	2	0	0
0	0	0	0	0	0	3	0	1
0	0	0	0	0	0	4	0	0
0	0	0	0	0	0	1	1	0
0	0	0	0	0	0	2	0	0
0	0	0	0	0	1	4	0	0
0	0	0	0	0	0	1	0	0
0	0	0	0	0	0	2	0	0
0	0	0	0	0	0	2	0	0
0	0	0	0	0	1	2	1	1
0	0	0	0	0	0	2	0	0
0	0	0	0	0	0	2	0	0
0	0	0	0	0	0	1	0	0
0	0	0	0	0	0	1	0	0
0	0	0	0	0	0	2	0	0
0	0	0	0	0	1	4	0	0
0	0	0	0	0	1	3	0	0
0	0	0	0	0	0	1	1	0
0	0	0	0	0	0	1	0	0
0	0	0	0	0	0	1	0	0
0	0	0	0	0	0	1	0	1
0	0	0	0	0	1	4	0	0
0	0	0	0	0	0	2	0	0

0	0	0	0	0	0	4	0	0
0	0	0	0	0	0	4	0	0
0	0	0	0	0	1	2	0	0
0	0	0	0	0	1	4	0	0
0	0	0	0	0	0	4	0	0
0	0	0	1	0	1	4	0	0
0	0	0	0	0	0	4	0	0
0	0	0	1	0	0	3	0	0
0	0	0	0	0	0	3	0	1
0	0	0	0	0	1	4	0	0
0	0	0	0	0	0	3	0	0
0	0	0	0	0	1	3	0	0
0	0	0	0	0	0	3	0	0
0	0	0	0	0	0	3	0	0
0	0	0	0	0	0	2	0	0
0	0	0	0	0	0	2	0	0
0	0	0	0	0	0	2	0	0
0	0	0	0	1	0	2	0	1
1	0	0	0	0	1	4	0	0
0	0	0	0	0	0	2	0	0
0	0	0	0	0	0	1	1	1
0	0	0	0	0	0	1	0	0
0	0	0	0	0	0	2	0	0
0	0	0	0	0	0	2	0	0
0	0	0	0	0	0	1	0	0
0	0	0	0	0	0	1	1	0
0	0	0	0	0	0	2	0	0
0	0	0	0	0	0	2	0	0
0	0	0	0	0	0	2	0	0
0	0	0	1	0	0	4	0	0
0	0	0	0	0	0	2	0	0
0	0	0	0	0	0	1	0	0
0	0	0	0	0	0	1	0	0
0	0	0	0	0	0	2	0	0
0	0	0	0	0	0	1	0	0
0	0	0	0	0	0	1	1	1
0	0	0	0	1	1	4	0	0
0	0	0	0	0	0	2	0	0
0	0	0	1	0	1	2	0	1
0	0	0	0	0	0	1	0	0
0	0	0	0	0	0	3	0	0
0	0	0	0	0	0	2	0	0
0	0	0	0	0	0	1	0	0
0	0	0	0	0	0	4	0	0
0	0	0	0	0	0	3	0	0
0	0	0	0	0	0	2	0	0
0	0	0	0	0	0	4	0	0
0	0	0	0	0	0	1	0	0

0	0	0	0	0	0	4	0	0
1	0	0	0	0	0	4	1	1
0	0	0	0	0	0	3	0	0
0	0	0	0	0	0	3	0	0
0	0	0	0	0	0	3	1	0
1	0	0	0	0	1	4	0	0
0	0	0	0	0	0	3	1	1
0	0	0	0	0	1	4	0	0
0	0	0	0	0	0	3	0	0
1	0	0	0	0	0	4	0	0
0	0	0	0	1	1	4	0	0
1	0	0	0	0	0	3	0	0
0	0	0	0	1	1	4	0	0
0	0	0	0	0	0	4	0	0
0	0	0	0	0	1	4	0	0
0	0	0	0	0	1	3	0	0
0	0	0	0	0	0	4	0	0
0	0	0	0	0	0	3	0	1
0	0	0	0	0	0	2	0	0
0	0	0	0	0	0	3	0	0
0	0	0	0	0	0	3	0	0
0	0	0	0	0	1	4	0	1
1	0	0	1	0	0	3	0	1
0	0	0	0	0	0	3	0	0
0	0	0	0	0	0	2	1	1
0	0	0	0	0	0	2	0	0
0	0	0	0	0	0	2	0	0
0	0	0	0	0	0	2	0	0
0	0	0	0	0	0	3	0	0
0	0	0	0	0	0	2	0	0
0	0	0	0	0	0	4	0	0
0	0	0	0	0	0	3	0	0
0	0	0	0	0	0	2	0	0
0	0	0	0	0	0	3	0	0
0	0	0	0	0	0	3	0	0
0	0	0	0	0	1	4	0	1
0	0	0	0	0	0	2	0	0
1	0	0	0	1	1	2	0	0
0	0	0	0	0	0	2	0	0
0	0	0	0	0	0	2	0	0
0	0	0	0	0	0	2	1	0
0	0	0	0	0	0	2	0	0
0	0	0	0	0	0	1	0	0
0	0	0	0	0	0	2	0	0
0	0	0	0	0	0	2	0	0
0	0	0	0	0	0	1	0	0
0	0	0	0	0	0	1	0	0
0	0	0	0	0	0	4	0	0

0	0	0	0	0	0	2	0	1
0	0	0	0	0	0	2	0	0
0	0	0	0	0	0	1	0	0
0	0	0	0	0	0	1	0	0
0	0	0	0	0	0	2	0	0
0	0	0	0	0	0	3	0	0
1	0	0	0	1	0	2	0	0
0	0	0	0	0	0	1	0	0
0	0	0	0	0	0	1	0	0
0	0	0	1	1	1	4	0	0
0	0	0	0	0	1	4	0	0
0	0	0	0	0	0	3	0	0
0	0	0	0	0	0	3	0	0
0	0	0	0	0	0	2	0	0
0	0	0	0	0	0	2	0	0
0	0	0	0	0	0	2	0	0
0	0	0	0	0	0	2	1	0
0	0	0	0	0	0	4	0	0
0	0	0	0	0	1	3	1	0
0	0	0	0	0	0	4	0	0
0	0	0	0	0	0	4	0	0
0	0	0	0	0	0	3	0	0
0	0	0	0	0	1	3	0	1
0	0	0	0	0	0	3	0	0
0	0	0	0	0	0	4	0	0
0	0	0	0	0	1	4	1	1
0	0	0	0	0	0	4	0	0
1	1	0	0	1	1	4	0	0
0	0	0	0	0	1	3	0	0
0	0	0	0	0	0	2	0	0
1	0	0	0	0	0	3	0	0
0	0	0	0	0	0	2	0	0
0	0	0	0	0	0	2	0	0
0	0	0	0	0	0	2	0	0
0	0	0	0	0	0	1	0	0
0	0	0	0	0	0	2	0	0
0	0	0	0	0	1	2	0	0
0	0	0	0	0	0	2	0	0
0	0	0	0	0	0	2	0	0
0	0	0	0	0	0	2	1	0
0	0	0	0	0	0	1	0	1
0	0	0	0	0	1	4	0	0
0	0	0	0	1	1	4	0	0
0	0	0	0	0	0	3	0	0
0	0	0	0	0	0	2	0	0
0	0	0	0	0	0	3	0	0
0	0	0	0	0	0	3	0	0
0	0	0	0	0	0	2	0	0

0	0	0	0	0	0	2	1	1
0	0	0	0	0	0	1	0	0
0	0	0	0	0	0	2	0	0
0	0	0	0	0	0	1	0	0
0	0	0	0	0	0	2	0	0
0	0	0	0	0	0	2	1	1
0	0	0	0	0	0	2	0	0
0	0	0	0	0	1	2	0	0
0	0	0	0	0	0	2	0	0
0	0	0	0	0	0	2	0	0
0	0	0	0	0	0	2	0	0
0	0	0	0	0	0	1	0	0
0	0	0	0	0	0	2	0	1
0	0	0	0	0	0	2	0	0
0	0	0	0	0	1	2	0	0
0	0	0	0	0	0	1	1	0
0	0	0	0	0	0	1	0	0
0	0	0	0	0	0	2	0	0
0	0	0	0	0	0	2	0	0
0	0	0	0	0	0	1	0	0
0	0	0	0	0	0	3	0	0
0	0	0	0	0	0	3	0	0
0	0	0	0	0	0	4	0	1
0	0	0	0	0	1	4	0	0
0	0	0	0	1	1	4	0	0
0	0	0	0	0	1	4	0	0
1	0	0	0	0	1	4	0	0
0	0	0	0	0	1	4	0	0
1	1	0	0	1	1	4	0	1
0	0	0	0	0	1	4	0	0
0	0	0	1	0	1	4	0	0
0	0	0	0	0	0	4	0	0
1	0	0	0	0	0	4	0	0
0	0	0	0	0	0	3	1	1
0	0	0	0	0	0	4	0	0
0	0	0	1	1	1	4	0	0
1	1	0	0	1	1	4	0	0
0	0	0	0	0	0	4	0	0
0	0	0	0	0	0	3	0	0
1	1	1	0	1	1	3	1	0
0	0	0	0	0	1	4	0	0
0	0	0	0	0	0	4	0	0
0	0	0	0	0	0	3	0	0
0	0	0	0	0	0	4	0	0
1	0	0	0	0	1	4	0	0
0	0	0	0	0	0	3	0	0
0	0	0	0	0	1	4	0	0
1	0	0	0	0	1	4	0	0

1	0	1	0	1	1	3	0	0
1	0	0	0	1	1	4	0	1
0	0	0	0	0	1	4	0	0
0	0	0	0	0	0	4	0	0
0	0	0	0	0	1	4	0	0
0	0	0	0	0	0	4	0	0
0	0	0	0	0	0	4	0	0
0	0	0	0	0	1	4	0	0
0	0	0	0	0	0	3	0	0
0	0	0	0	0	1	3	0	0
0	0	0	0	0	1	3	0	0
0	0	0	0	0	1	3	0	0
0	0	0	0	0	1	4	0	0
0	0	0	0	0	0	3	0	1
0	0	0	1	0	0	3	0	0
0	0	0	0	0	1	2	0	0
0	0	0	0	0	0	4	0	0
0	0	0	0	0	0	4	0	0
0	0	0	0	0	0	2	0	0
0	0	1	0	0	1	3	0	0
0	0	0	0	0	0	3	0	0
0	0	0	0	0	1	4	0	1
1	0	0	0	0	1	3	0	0
0	0	0	0	0	0	3	0	0
0	0	0	0	0	0	2	0	0
0	0	0	0	0	0	3	0	0
0	0	0	0	0	0	2	0	0
0	0	0	0	0	0	2	0	0
0	0	0	0	0	0	2	0	0
0	0	0	0	0	0	2	1	0
0	0	0	0	0	0	1	0	0
0	0	0	0	0	0	4	0	0
0	0	0	0	0	0	3	0	0
0	0	0	0	1	0	4	0	0
0	0	0	0	0	0	4	0	0
0	0	0	0	1	1	4	0	1
0	0	0	0	0	0	2	0	0
0	0	0	0	0	0	3	0	1
0	0	0	0	0	1	3	0	0
0	0	0	0	0	1	4	0	0
0	0	0	0	0	0	2	0	0
0	0	0	0	0	0	4	0	0
0	0	0	0	0	0	3	0	0
1	0	0	0	0	0	2	0	1
0	0	0	0	0	0	2	0	0
0	0	0	0	0	0	2	0	0
0	0	0	0	0	0	2	0	0
0	0	0	0	0	0	2	0	0

0	0	0	0	0	0	2	0	0
0	0	0	0	1	1	4	0	0
0	0	0	0	0	0	2	0	0
0	0	0	0	0	1	2	0	0
0	0	0	0	0	1	2	0	0
1	0	0	0	0	1	1	0	0
0	0	0	0	0	0	1	0	1
0	0	0	0	0	0	4	0	0
0	0	0	0	0	0	1	0	0
0	0	0	0	0	0	1	0	0
0	0	0	0	0	0	1	0	0
0	0	0	0	0	0	2	0	0
0	0	0	0	0	0	1	1	0
0	0	0	0	0	1	1	0	0
0	0	0	0	0	0	3	0	0
0	0	0	0	0	0	3	0	0
0	0	0	0	0	0	3	0	0
0	0	0	0	0	0	2	0	0
0	0	0	0	0	0	2	1	0
0	0	0	0	0	0	2	0	0
0	0	0	0	0	0	3	0	1
0	0	0	0	0	0	2	0	0
0	0	0	0	0	0	2	0	0
0	0	0	0	0	0	2	0	0
0	0	0	0	0	0	3	0	0
0	0	0	0	0	0	4	0	0
0	0	0	0	0	0	2	0	0
0	0	0	0	0	0	2	0	0
0	0	0	0	0	0	2	0	0
0	0	0	0	0	0	1	0	0
0	0	0	0	0	0	2	0	0
0	0	0	0	0	0	1	0	1
0	0	0	0	0	0	3	0	0
0	0	0	0	0	0	2	0	0
0	0	0	0	0	0	2	0	1
0	0	0	0	0	0	1	0	0
0	0	0	0	0	0	1	0	0
0	0	0	0	0	0	1	0	0
0	0	0	0	0	0	3	0	0
0	0	0	0	0	0	2	0	0
0	0	0	0	0	0	2	0	0
0	0	0	0	0	0	3	1	0
0	0	0	0	0	0	1	0	0
0	0	0	0	0	0	2	0	0
0	0	0	0	0	0	3	1	1
0	0	0	0	0	0	1	0	0
0	0	0	0	0	0	2	0	1
0	0	0	0	0	0	3	0	1

0	0	0	0	0	0	2	0	0
0	0	0	0	0	0	4	0	0
0	0	0	0	0	0	3	0	0
0	0	0	0	0	0	4	0	0
0	0	0	0	0	0	1	0	0
0	0	0	0	0	0	2	0	0
0	0	0	0	0	0	2	0	0
0	0	0	0	0	0	3	0	0
0	0	0	0	0	0	2	1	0
0	0	0	0	0	0	1	0	0
0	0	0	0	0	0	1	0	1
0	0	0	0	0	0	1	0	1
0	0	0	0	0	0	3	0	0
0	0	0	0	0	0	3	0	1
0	0	0	0	0	0	4	0	0
0	0	0	0	0	0	4	0	0
0	0	0	0	0	0	3	0	0
0	0	0	0	0	0	3	0	0
0	0	0	0	0	0	4	0	0
0	0	0	0	0	0	4	0	0
0	0	0	0	0	0	2	0	0
0	0	0	0	0	0	3	0	0
0	0	0	0	0	0	3	0	1
0	0	0	0	0	0	2	0	0
0	0	0	0	0	0	3	1	0
0	0	0	0	0	0	3	0	0
0	0	0	0	0	0	4	0	0
0	0	0	0	0	0	4	0	0
0	0	0	0	0	0	2	0	0
0	0	0	0	0	0	4	0	0
0	0	0	0	0	0	2	0	0
0	0	0	0	0	0	4	0	0
0	0	0	0	0	0	2	0	0
0	0	0	0	0	0	4	0	0
0	0	0	0	0	0	3	0	0
0	0	0	0	0	0	2	0	0
0	0	0	0	0	0	3	0	0
0	0	0	0	0	0	4	0	0
0	0	0	0	0	0	4	0	0
0	0	0	0	0	0	3	0	0
0	0	0	0	0	0	4	0	0
0	0	0	0	0	0	4	0	0
0	0	0	0	0	0	4	0	0
0	0	0	0	0	0	3	0	0
0	0	0	0	0	0	4	0	0
0	0	0	0	0	0	4	0	0
0	0	0	0	0	0	2	0	0
0	0	0	0	0	0	3	0	0

0	0	1	0	0	0	2	0	0
0	0	0	0	0	0	3	0	0
0	0	0	0	0	0	3	0	0
0	0	0	0	0	0	3	0	0
0	0	0	0	0	0	4	1	1
0	0	0	0	0	0	3	0	0
0	0	0	0	0	0	4	0	0
0	0	0	0	0	0	3	0	0
0	0	0	0	0	0	3	0	0
0	0	0	0	0	0	3	0	0
0	0	0	0	0	0	3	0	0
0	0	0	0	0	0	3	0	0
0	0	0	0	0	0	3	1	0
0	0	0	0	0	0	4	0	0
0	0	0	0	0	0	3	0	0
0	0	0	0	0	0	2	0	0
0	0	0	0	0	0	4	0	0
0	0	0	0	0	0	3	0	0
0	0	0	0	0	0	2	0	0
0	0	0	0	0	0	3	0	0
0	0	0	0	0	0	4	0	0
0	0	0	0	0	0	3	0	0
0	0	0	0	0	0	2	0	0
0	0	0	0	0	0	3	0	0
0	0	0	0	0	0	3	1	1
0	0	0	0	0	0	1	0	0
0	0	0	0	0	0	3	0	0
0	0	0	0	0	0	2	0	0
0	0	0	0	0	0	4	0	0
0	0	0	0	0	0	2	0	0
0	0	0	0	0	0	4	0	0
0	0	0	0	0	0	2	0	0
0	0	0	0	0	0	3	1	0
0	0	0	0	0	0	2	0	0
0	0	0	0	0	0	2	0	0
0	0	0	0	0	0	3	0	0
0	0	0	0	0	0	2	0	0
0	0	0	0	0	0	2	0	0
0	0	0	0	0	0	2	0	0
0	0	0	0	0	0	4	0	0
0	0	0	0	0	0	4	0	0
0	0	0	0	0	0	2	0	0
0	0	0	0	0	0	2	0	0
0	0	0	0	0	0	1	0	0
0	0	0	0	0	0	2	0	0
0	0	0	0	0	0	1	0	1
0	0	0	0	0	0	2	0	0
0	0	0	0	0	0	2	0	0

0	0	0	0	0	0	3	0	0
0	0	0	0	0	0	2	0	0
0	0	0	0	0	0	1	0	0
0	0	0	0	0	0	2	0	0
0	0	0	0	0	0	2	0	0
0	0	0	0	0	0	2	0	0
0	0	0	0	0	0	3	0	0
0	0	0	0	0	0	1	0	0
0	0	0	0	0	0	1	0	0
0	0	0	0	0	0	2	1	1
0	0	0	0	0	0	3	0	0
0	0	0	0	0	0	4	0	0
0	0	0	0	0	0	1	0	0
0	0	0	0	0	0	2	0	0
0	0	0	0	0	0	1	0	0
0	0	0	0	0	0	3	0	1
0	0	0	0	0	0	1	0	0
0	0	0	0	0	0	1	0	0
0	0	0	0	0	0	2	0	0
0	0	0	0	0	0	2	0	0
0	0	0	0	0	0	1	0	0
0	0	0	0	0	0	2	0	0
0	0	0	0	0	0	3	0	0
0	0	0	0	0	0	1	0	0
0	0	0	0	0	0	1	0	0
0	0	0	0	0	0	2	0	0
0	0	0	0	0	0	4	0	0
0	0	0	0	0	0	4	0	0
0	0	0	0	0	0	4	0	0
0	0	0	0	0	0	4	0	0
0	0	0	0	0	0	4	0	0
0	0	0	0	0	0	2	0	0
0	0	0	0	0	0	2	0	0
0	0	0	0	0	0	4	0	0
0	0	0	0	0	0	4	0	0
0	0	0	1	0	0	4	0	0
0	0	0	0	0	0	4	0	0
0	0	0	0	0	0	4	0	0
0	0	0	0	0	0	3	0	0
0	0	0	0	0	0	4	0	0
0	0	0	0	0	0	1	0	0
0	0	0	0	0	0	4	0	0
0	0	0	0	0	0	4	0	1
0	0	0	0	0	0	3	1	0
0	0	0	0	0	0	3	0	0
0	0	0	0	0	0	4	0	1
0	0	0	0	0	0	3	0	1
0	0	0	0	0	0	2	0	0

0	0	0	0	0	0	3	0	0
0	0	0	0	0	0	4	0	0
0	0	0	0	0	0	4	0	0
0	0	0	0	0	0	2	0	0
0	0	0	0	0	0	4	0	0
0	0	0	0	0	0	3	0	0
0	1	1	0	1	0	3	0	0
0	0	0	0	0	0	3	0	0
0	0	0	0	0	0	3	0	0
0	0	0	0	0	0	4	0	0
0	0	0	0	0	0	2	0	0
0	0	0	0	0	0	3	0	1
0	0	0	0	0	0	4	0	0
0	0	0	0	0	0	3	0	0
0	0	0	0	0	0	3	0	0
0	0	0	0	0	0	3	0	0
0	0	0	0	0	0	1	0	0
0	0	0	0	0	0	3	1	0
0	0	0	0	0	0	1	0	0
0	0	0	0	0	1	4	0	0
0	0	0	0	0	0	3	0	0
0	0	0	0	0	0	3	0	0
0	0	0	0	0	0	1	1	0
0	0	0	0	0	0	2	0	0
0	0	0	0	0	0	3	0	0
0	0	0	0	0	0	2	0	0
0	0	0	0	0	0	2	0	0
0	0	0	0	0	0	2	0	0
0	0	0	0	0	0	2	0	0
0	0	0	0	0	0	2	0	1
0	0	0	0	0	0	4	0	0
0	0	0	0	0	0	2	0	0
0	0	0	0	0	0	2	0	0
0	0	0	0	0	0	4	0	0
0	0	0	0	0	0	2	0	0
0	0	0	0	0	0	1	0	0
0	0	0	0	0	0	2	0	0
0	0	0	0	0	1	3	0	0
0	0	0	0	0	0	4	0	0
0	0	0	0	0	0	1	0	0
0	0	0	0	0	0	2	0	0
0	0	0	0	0	0	3	0	1
0	0	0	0	0	0	1	0	0
0	0	0	0	0	0	3	0	0
0	0	0	0	0	0	2	0	0
0	0	0	0	0	0	2	0	0
0	0	0	0	0	0	4	0	0
0	0	0	0	0	0	3	0	0

0	0	0	0	0	0	3	0	0
0	0	0	0	0	0	2	0	0
0	0	0	0	0	0	1	0	0
0	0	0	0	0	0	4	0	0
0	0	0	0	0	0	3	0	0
0	0	0	0	0	0	3	0	0
0	0	0	0	0	0	3	0	0
0	0	0	0	0	0	3	0	0
0	0	0	0	0	0	2	0	0
0	0	0	0	0	0	2	0	0
0	0	0	0	0	0	2	0	1
0	0	0	0	0	0	3	0	0
0	0	0	0	0	0	1	0	0
0	0	0	0	0	0	2	0	0
0	0	0	0	0	0	2	0	0
0	0	0	0	0	0	4	0	0
0	0	0	0	0	0	2	0	0
0	0	0	0	0	0	1	0	0
0	0	0	0	0	0	3	0	0
0	0	0	0	0	0	1	0	0
0	0	0	0	0	0	2	0	0
0	0	0	0	0	1	4	1	0
0	0	0	0	0	0	3	0	0
0	0	0	0	0	0	2	0	0
0	0	0	1	0	1	3	0	0
0	0	0	0	0	0	3	0	0
0	0	0	0	0	0	4	0	0
0	0	0	0	0	0	4	0	0
0	0	0	0	0	1	3	0	0
0	0	0	0	0	0	3	0	0
0	0	0	0	0	0	3	0	0
0	0	0	0	0	0	4	0	0
0	0	0	0	0	0	4	0	0
0	0	0	0	1	1	4	0	0
0	0	0	0	0	0	4	0	0
0	0	0	0	0	0	4	0	0
0	0	0	0	0	0	3	0	0
0	0	0	0	0	0	3	0	0
0	0	0	0	0	1	4	0	0
0	0	0	0	0	0	2	0	0
0	0	0	0	0	0	4	0	0
1	0	0	0	0	0	4	0	0
0	0	0	0	0	0	3	0	1
0	0	0	0	0	0	2	0	0
0	0	0	0	0	0	3	0	0
0	0	0	0	0	1	4	0	0
1	1	0	0	0	1	4	0	0
0	0	0	0	0	0	3	0	0

1	0	0	0	0	1	4	0	0
0	0	0	0	0	0	4	0	0
0	0	0	0	0	0	2	0	0
0	0	0	0	0	0	4	0	0
1	0	0	0	0	1	2	0	0
0	0	0	0	0	0	4	0	0
0	0	0	0	0	0	2	0	1
0	0	0	0	0	1	4	0	0
0	0	0	0	0	0	2	0	0
0	0	0	0	0	0	3	0	0
0	0	0	0	0	0	1	0	0
0	0	0	0	0	0	4	0	0
0	0	0	0	0	0	3	0	1
0	0	0	0	0	0	4	0	0
0	0	0	0	0	0	3	0	0
0	0	0	0	0	0	2	0	0
0	0	0	0	0	0	2	0	0
0	0	0	0	0	0	2	0	0
0	0	0	0	0	0	4	0	0
0	0	0	0	0	0	2	0	0
0	0	0	0	0	0	4	0	0
0	0	0	0	0	0	2	0	0
0	0	0	0	0	0	2	0	0
0	0	0	0	0	0	3	0	0
0	0	0	0	0	0	2	0	0
0	0	0	0	0	1	4	0	0
0	0	0	0	0	0	2	0	0
0	0	0	0	0	0	4	0	0
0	0	0	0	0	0	1	0	0
0	0	0	0	0	0	2	0	1
0	0	0	0	0	1	3	0	0
0	0	0	0	0	0	4	0	0
0	0	0	0	0	1	3	1	1
0	0	0	0	0	0	3	0	0
0	0	0	0	0	1	3	0	0
0	0	0	0	0	0	3	0	0
0	0	0	0	0	0	3	0	0
0	0	0	0	0	0	4	0	0
0	0	0	0	0	0	3	0	0
0	0	0	0	0	1	4	0	0
0	0	0	0	0	0	3	0	0
0	0	0	0	0	1	3	0	0
0	0	0	0	0	0	3	0	0
0	0	0	0	0	1	3	0	1
0	0	0	0	0	0	2	0	0
0	0	0	0	0	0	2	0	0
0	0	0	0	0	0	3	0	0
0	0	0	0	0	1	3	0	0

0	0	0	0	0	1	3	0	0
0	0	0	0	0	0	3	0	0
0	0	0	0	0	0	3	0	0
0	0	0	0	0	0	3	0	0
0	0	0	0	0	0	2	0	0
0	0	0	0	0	0	2	0	0
0	0	0	0	0	0	2	0	0
0	0	0	0	0	0	4	0	0
0	0	0	0	0	0	2	0	0
0	0	0	0	0	0	1	0	0
0	0	0	0	0	0	2	0	0
0	0	0	0	0	0	1	0	0
0	0	0	0	0	1	2	0	0
0	0	0	0	0	0	1	1	1
0	0	0	0	0	0	3	1	0
0	0	0	0	0	0	1	0	0
0	0	0	0	0	0	1	0	0
0	0	0	0	0	0	2	0	0
0	0	0	0	0	0	4	0	0
0	0	0	0	0	0	1	0	0
0	0	0	0	0	0	4	1	0
0	0	0	0	0	0	4	0	0
0	0	0	0	0	0	4	0	1
0	0	0	0	0	1	3	0	0
0	0	0	0	0	0	4	0	1
1	0	0	0	1	1	4	0	0
0	0	0	0	0	0	2	0	0
0	0	0	0	0	0	3	0	0
0	0	0	0	0	1	4	0	1
0	0	0	0	0	0	4	0	0
0	0	0	0	0	1	4	1	1
0	0	0	0	0	0	4	0	0
1	0	0	0	0	1	3	0	0
0	0	0	1	0	1	3	0	0
0	0	0	0	0	0	2	0	0
0	0	0	0	0	0	4	0	0
0	0	0	0	0	0	3	0	0
1	0	0	0	0	0	2	0	0
0	0	0	0	0	0	2	0	0
0	0	0	0	0	0	2	1	0
0	0	0	0	0	0	4	0	0
0	0	0	0	0	0	2	0	0
0	0	0	0	0	1	2	0	0
0	0	0	0	0	0	2	0	0
0	0	0	0	0	0	3	0	0
0	0	0	0	0	0	2	1	0
0	0	0	0	0	0	2	0	0
0	0	0	0	0	0	2	0	0

0	0	0	0	0	0	2	0	0
0	0	0	0	0	0	1	0	0
0	0	0	0	0	0	4	0	0
0	0	0	0	0	0	3	0	0
0	0	0	0	0	0	3	0	0
0	0	0	0	0	0	2	0	0
0	0	0	0	0	0	4	0	0
0	0	0	0	0	0	2	0	0
0	0	0	0	0	0	2	0	0
0	0	0	0	0	0	1	0	0
0	0	0	0	0	0	1	0	1
0	0	0	0	0	0	1	0	0
0	0	0	0	0	0	2	0	0
0	0	0	0	1	0	4	0	0

**Coma**
0
1
0
0
0
0
0
0
1
0
0
0
0
0
0
0
0
1
0
1
0
0
0
0
0
0
0
0
0
1
0
0
1
1
0
0
0
0
0
0
0
0
0
0
1
0
0

0
0
0
0
0
0
1
1
0
0
0
0
0
0
0
0
1
0
0
0
0
0
0
0
0
0
0
0
0
1
0
0
0
1
0
0
1
0
1
0
0
0
0
0
0
0
0
0

0
0
0
0
0
0
0
0
0
0
0
0
0
0
0
0
0
0
0
0
0
0
0
0
0
0
0
0
0
1
0
0
0
0
0
0
0
0
0
0
0
0
0
0
0
0
0
0

0
0
1
0
1
0
0
0
0
0
0
0
0
0
0
0
0
0
0
0
0
0
0
0
0
0
0
0
0
0
1
0
0
0
0
0
0
0
0
0
0
0
0
0
0
0
0
0

0
0
0
0
0
0
0
0
0
0
0
0
0
0
0
0
0
0
0
0
0
0
0
0
1
0
0
0
0
0
0
0
0
0
0
0
0
0
0
0
1
0
0
0
0
0
0
0

0
0
1
0
0
0
0
0
1
0
0
0
0
0
0
0
0
0
0
0
0
0
0
0
0
0
0
1
0
1
0
0
0
0
0
0
0
0
0
0
0
0
0
0
0
0
0
0

0
1
0
0
0
1
1
0
0
0
1
0
0
0
0
0
0
0
0
0
0
1
0
1
0
0
0
0
0
0
0
0
0
0
1
0
0
0
0
0
0
0
0
0
0
0
1
0

0
1
1
0
0
0
1
0
1
0
0
0
0
0
0
0
0
0
0
0
0
0
0
0
0
0
0
0
0
0
0
0
0
0
0
0
0
0
0
0
0
0
0
0
0
0
0
0

0
0
0
0
0
0
0
0
0
0
0
0
0
0
0
0
0
0
0
0
0
0
0
0
0
0
0
0
0
0
0
0
0
0
0
0
0
0
0
0
0
0
0
0
0
0
0
0

0
0
0
0
0
0
0
0
0
0
1
0
0
0
0
0
0
0
0
0
1
1
0
0
0
0
0
0
0
0
0
0
0
0
0
0
0
1
0
0
0
0
1
0
0
0
0
0

0
0
0
0
0
0
0
1
0
0
0
0
0
0
0
0
0
0
0
0
0
0
0
0
0
0
0
0
0
1
0
0
0
0
0
0
0
0
0
0
0
0
0
0
0
0
0
0

0
0
0
0
1
0
0
0
0
0
0
0
0
1
0
0
0
0
0
0
0
0
0
0
0
0
0
0
0
0
0
0
0
0
0
0
0
0
0
0
0
0
0
0
0
0
0
0

0
0
0
0
0
0
0
0
0
0
0
0
0
0
0
0
0
0
0
0
0
0
0
0
0
0
0
0
0
0
0
0
0
0
0
0
0
0
0
0
0
0
0
0
0
0
0
0

0
0
0
0
0
0
0
0
0
0
0
0
0
0
0
0
1
0
0
0
0
0
0
0
0
0
0
0
0
0
0
0
0
0
1
0
0
0
0
0
0
0
0
0
0
0
0
0

0
0
0
0
1
0
0
0
0
0
0
0
0
0
0
0
0
0
0
0
0
0
0
0
0
0
0
0
0
0
0
0
0
0
0
1
0
0
0
0
0
0
0
0
0
0
0
0

0
0
0
0
0
0
0
0
0
0
0
0
0
0
0
0
0
0
0
0
0
0
1
0
0
0
1
0
0
1
0
0
0
0
0
1
0
0
1
0
0
0
0
0
1
1
0
0

0
0
0
0
0
0
0
0
0
0
0
0
0
0
0
0
0
0
0
0
0
0
0
0
0
0
0
0
0
0
0
0
0
0
0
0
1
0
0
0
0
0
0
0
0
0
0
1

0

0

0

0

0

0

0

0

0

1

1

0

0

0

0

0

0

1

0

0

0

0

0

0

0

0

0

0

0

0

1

1

1

0

0

0

0

0

1

0

0

0

0

0

0

1

0

0

0
0
0
0
0
0
0
1
0
0
0
0
0
0
0
0
0
0
0
0
0
0
0
0
0
1
0
0
0
0
0
0
0
0
0
0
0
0
0
0
0
0
0
0
0
0
0
0

1
0
0
0
0
0
0
0
0
0
1
0
0
0
0
0
0
0
0
0
0
0
0
1
0
0
1
0
0
0
0
0
0
0
0
0
0
0
0
0
1
0
0
0
0
0
0
0

0
0
1
0
0
0
0
0
0
0
0
0
0
0
